# Biphasic electrochemical peptide synthesis†

**DOI:** 10.1039/d1sc03023j

**Published:** 2021-09-02

**Authors:** Shingo Nagahara, Yohei Okada, Yoshikazu Kitano, Kazuhiro Chiba

**Affiliations:** Department of Applied Biological Science, Tokyo University of Agriculture and Technology 3-5-8- Saiwai-cho Fuchu Tokyo 183-8509 Japan chiba@cc.tuat.ac.jp

## Abstract

The large amount of waste derived from coupling reagents is a serious drawback of peptide synthesis from a green chemistry viewpoint. To overcome this issue, we report an electrochemical peptide synthesis in a biphasic system. Anodic oxidation of triphenylphosphine (Ph_3_P) generates a phosphine radical cation, which serves as the coupling reagent to activate carboxylic acids, and produces triphenylphosphine oxide (Ph_3_P=O) as a stoichiometric byproduct. In combination with a soluble tag-assisted liquid-phase peptide synthesis, the selective recovery of desired peptides and Ph_3_P=O was achieved. Given that methods to reduce Ph_3_P=O to Ph_3_P have been reported, Ph_3_P=O could be a recyclable byproduct unlike byproducts from typical coupling reagents. Moreover, a commercial peptide active pharmaceutical ingredient (API), leuprorelin, was successfully synthesized without the use of traditional coupling reagents.

## Abstract 2

The large amount of waste derived from coupling reagents is a serious drawback of peptide synthesis from a green chemistry viewpoint.

## Graphical abstract

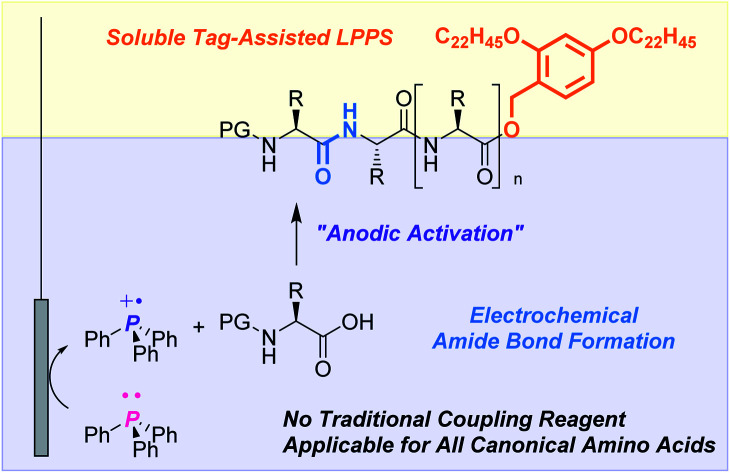

## Introduction

**Scheme 1:**
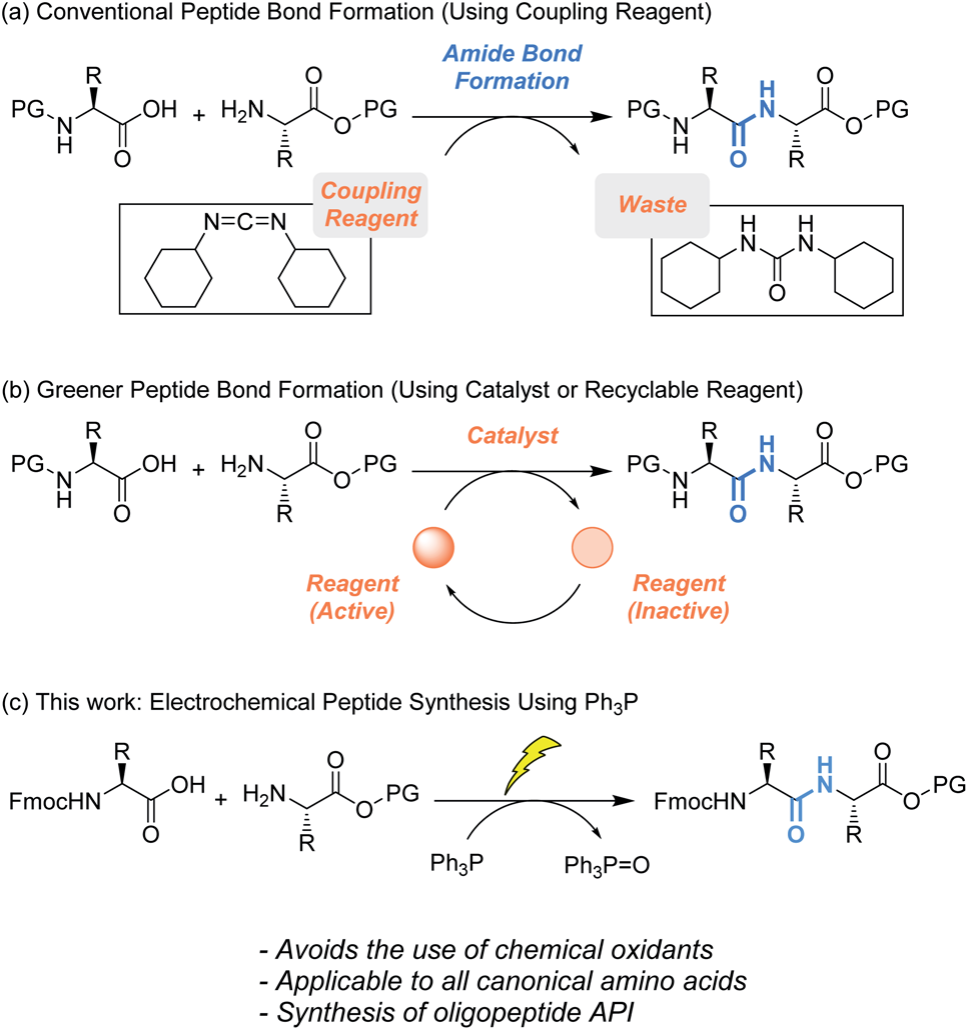
(a) Conventional and (b) greener peptide bond formation. (c) Electrochemical peptide bond formation.

Recently, peptides have been recognized as candidates for “medium” molecular medicines,^1*a*^ which refers to pharmaceutical compounds whose molecular weights are roughly in the 1000 to 5000 range. This class of medicines has more specificity and fewer side effects than conventional small molecular medicines.^1*b*^ In 2018, over 60 peptides were approved as drugs in the United States, Europe and Japan. In addition, over 150 peptides are in active clinical development, and more than 260 have been tested in human clinical trials.^2^ Even as peptides are gaining such a great deal of attention, the long-standing problem of the large amount of waste produced has not been resolved.^1^ One contributor to waste generation is the use of coupling reagents that enable efficient peptide bond formation.^3^ Generally, peptides are synthesized through repetitive deprotection and peptide bond formation reactions. To form peptide bonds between carboxylic acids and amines, stoichiometric amounts of coupling reagents are required (Scheme 1a), which generate stoichiometric byproducts, leading to accumulation of chemical waste.^4^ In this regard, the development of greener peptide synthetic processes is identified as a key for green chemistry.^1*a*,*e*,*g*^

To address this issue, catalytic peptide synthesis has emerged (Scheme 1b, above arrow). Since Yamamoto reported boronic acid catalyzed amide bond formation,^5*a*^ various organoboron catalysts have been developed,^5*b*–*g*^ some of which have proven to be effective for oligopeptide synthesis. The mechanism of the reaction has been well studied, which has aided researchers in designing more sophisticated catalysts. In addition, recent studies revealed that several metals act as Lewis acid catalysts. These catalysts form active complexes with *in situ* generated esters, and promote peptide bond formation while avoiding epimerization.^5*h*,*i*^

We envisioned that development of an efficient peptide synthetic method using potentially recyclable reagents would pave the way to an alternative solution (Scheme 1b, below arrow). For this strategy, the use of phosphine (R_3_P) would be a promising approach. Oxidative activation of R_3_P generates an electrophilic phosphine cation, which serves as the coupling reagent to activate carboxylic acids and facilitate amide bond formation.^6^ In 2019, the Arora group reported efficient oligopeptide synthesis using Bu_3_P activated by a diselenide catalyst. Although phosphine oxide (R_3_P=O) is produced as a stoichiometric byproduct, its reduction back to R_3_P has been achieved in various ways.^7^ Therefore, R_3_P=O can be a recyclable byproduct, unlike byproducts from typical peptide coupling reagents. Among R_3_P, triphenylphosphine (Ph_3_P) would be most suitable because Ph_3_P is easy to handle and reduction of triphenylphosphine oxide (Ph_3_P=O) to Ph_3_P proceeds more efficiently than the case for alkyl phosphines. Moreover, Sevov reported an electrochemical reduction method applicable on a large scale,^7*k*^ and Favre-Réguillon has also succeeded in the conversion of Ph_3_P=O to Ph_3_P on a 100 g scale by the combination of Ti(O*i*Pr)_4_ and hydrosiloxane.^7*i*^ Therefore, development of peptide synthesis utilizing Ph_3_P and recovery of Ph_3_P=O would demonstrate the potential of Ph_3_P as a recyclable coupling reagent.

**Fig. 1:**
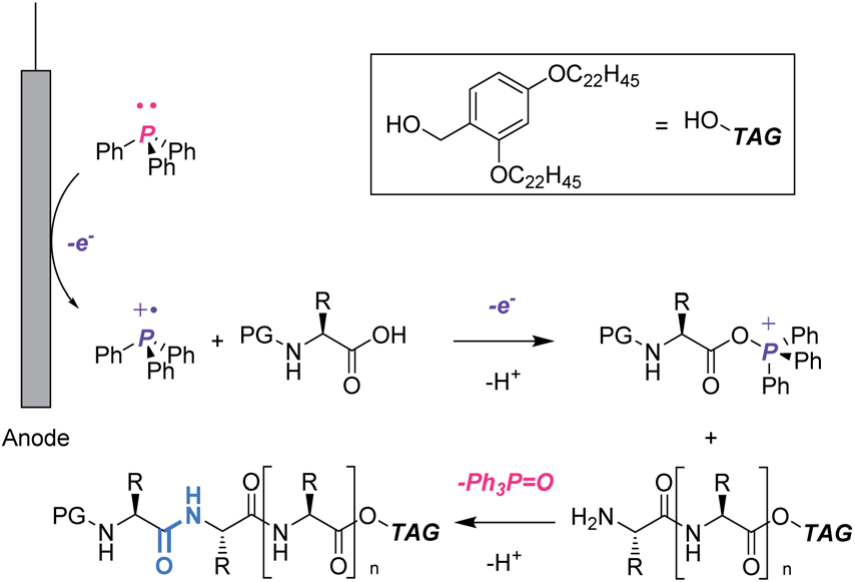
Electrochemical peptide synthesis utilizing soluble tag-assisted method.

In this context, we aimed to develop a peptide synthesis method using Ph_3_P by performing electrochemical amide bond formation as reported by Frontana-Uribe.^8^ In this electrochemical method, because electrons themselves act as an oxidant to generate a phosphine cation to facilitate amide bond formation, waste from chemical oxidants can be avoided.^9^ To facilitate recovery of Ph_3_P=O, soluble tags as the carboxylic acid protecting groups could be combined with the electrochemical method.^10^ Soluble tags are benzyl alcohols bearing long alkyl chains, so peptides protected with soluble tags dissolve in THF, CH_2_Cl_2_ and *c*-Hex, but precipitate in polar solvents like MeCN. Hence, peptide synthesis is conducted in the liquid phase, and purification can be accomplished simply by filtration, which realizes facile separation of peptides and Ph_3_P=O. In this paper, we describe biphasic electrochemical peptide bond formation, leading to the synthesis of a commercial peptide active pharmaceutical ingredient (API), leuprorelin, without the use of traditional coupling reagents and recovery of Ph_3_P=O from the reaction mixture (Fig. 1).

## Results and discussion

First, we optimized the reaction between Fmoc-Asp(O^*t*^Bu)-OH (**1a**) and H-Asp(O^*t*^Bu)-O**TAG** (**2a**) as models using Ph_3_P (Table 1). In CH_2_Cl_2_, the reaction proceeded efficiently to give the dipeptide (**3aa**) in 92% yield (entry 1). On the other hand, the yield was significantly decreased to 19% in THF and most starting materials were recovered even when 4.8 F mol^−1^ was passed (entry 2). Since THF is more coordinating than CH_2_Cl_2_, it is likely that THF coordinates to Ph_3_P˙^+^ to lower its electrophilicity, which leads to low efficiency of carboxylic acid activation.^11^ A biphasic condition (MeCN/*c*-Hex) was also found to be effective,^12^ affording **3aa** in excellent yield (entry 3, Fig. S1 and Scheme S1†). Hydrophobic **2a** is localized in the upper *c*-Hex, while polar **1a** and other reagents are selectively dissolved in the lower MeCN, where the electrochemical reaction takes place. The peptide bond formation between the activated **1a** and **2a** is expected to occur at the surface of the reversed micelle. Since **3aa** is also localized in *c*-Hex, it is noteworthy that its separation from the supporting electrolyte is possible by simple phase separation. Furthermore, in the biphasic condition, since *c*-Hex is not conductive, undesired oxidation of **2a** and/or overoxidation of **3aa** could be suppressed. Avoiding the use of hazardous CH_2_Cl_2_ is preferred from the green chemistry viewpoint, and therefore, further investigations were carried out using the biphasic condition.

**Table 1:** Optimization and comparison studies for electrochemical peptide bond formation^a^

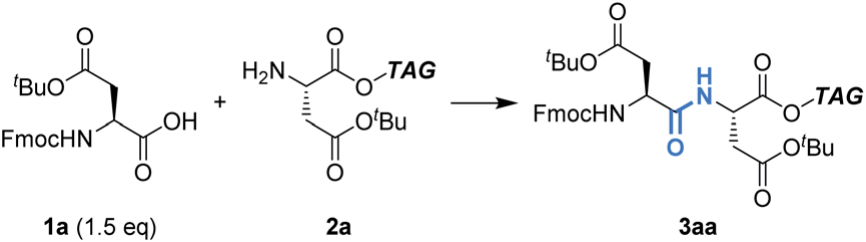		
Entry^b^	Electrolyte solution	Yield^c^ (%)
1	Bu_4_NClO_4_/CH_2_Cl_2_	92
2	Bu_4_NClO_4_/THF	19
3	Bu_4_NClO_4_/*c*-Hex/MeCN	95
4	LiClO_4_/*c*-Hex/MeCN	86
5	NaClO_4_/*c*-Hex/MeCN	—
6	KClO_4_/*c*-Hex/MeCN	55

a Conditions: Ph_3_P (2.0 eq.), 2,6-lutidine (3.0 eq.), supporting electrolyte (0.05 M), platinum electrodes, 2.0 mA, 4.8 F mol^−1^, rt, undivided cell.

b Carried out at 0.20 mmol scale (**2a**).

c Determined by NMR analysis.

When LiClO_4_ or KClO_4_ was used instead of Bu_4_NClO_4_, the yields decreased somewhat (entries 4 and 6), while the use of NaClO_4_ caused severe gelation of the reaction mixture (entry 5). These observations suggest that tetrabutylammonium ions are more suitable for the reaction than alkali metal cations, presumably due to weaker interactions with the carboxylate anion of **1a**. Moreover, ^31^P NMR indicated that the transformation of Ph_3_P into Ph_3_P=O was selective. After further investigation of the procedure, we succeeded in the recovery of both Bu_4_NClO_4_ (>99%) and Ph_3_P=O (91%) with high purity (>95%) without column purification (detailed procedure and purity assessment are in ESI†).

Having confirmed the potential of Ph_3_P as a recyclable coupling reagent, we explored the feasibility of electrochemical peptide bond formation with other Fmoc-protected amino acids (**1b–1u**) instead of **1a**, using **2a** as a reaction partner (Table 2). Although heating was needed in some cases to avoid gelation of the reaction mixture, all the Fmoc-protected canonical amino acids were demonstrated to be amenable to the reaction. It should be noted that not only amino acids with redox inactive alkyl side chains but also those with redox active moieties, such as Cys and Met, gave the desired products in excellent yields. When Fmoc-His(Trt)-OH was used (**3pa**), however, partial epimerization occurred, likely due to the basicity of the imidazole moiety.^13^ Boc is an electron-withdrawing alternative to Trt for the protection of imidazole, which may decrease its basicity. Using Boc, we succeeded in preventing the epimerization and the desired product (**3qa**) was obtained in excellent yield as a pure stereoisomer. Slightly excessive amounts of reagents and electricity were needed for the reaction of Fmoc-Pro-OH (**3ua**), presumably due to steric hindrance.

**Table 2:** Scope of Fmoc-protected amino acids

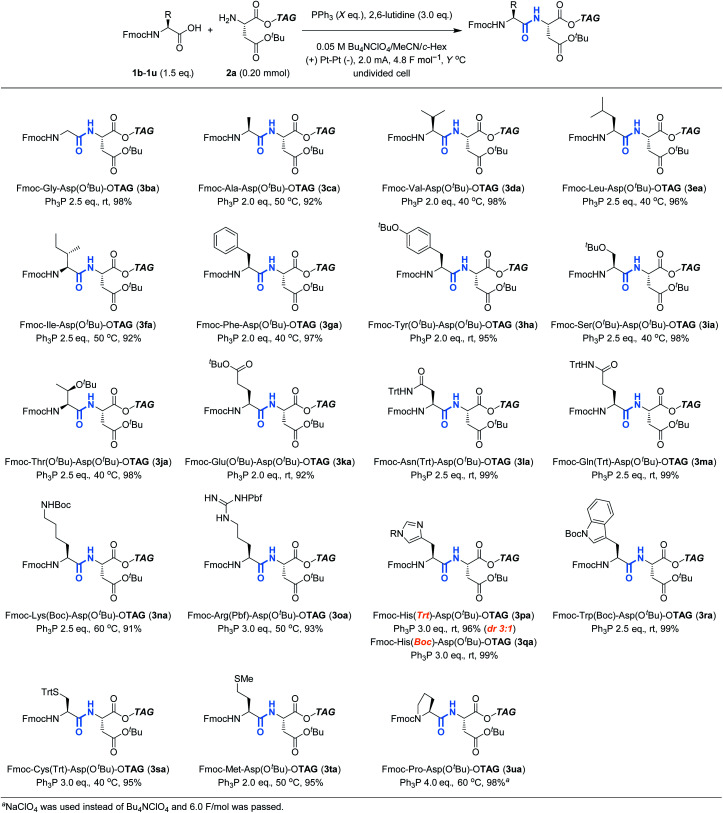

We next turned our attention to explore the compatibility of this method with other tag-protected amino acids (**2b–2u**) instead of **2a**, using **1a** as a reaction partner (Table 3). Although heating was also needed in some cases to avoid gelation of the reaction mixture, all the tag-protected canonical amino acids were demonstrated to be amenable to the reaction. In this case, no significant epimerization was observed for the reaction of H-His(Trt)-O**TAG**, and the desired product (**3ap**) was obtained in excellent yield as a pure stereoisomer. The reaction of H-Pro-O**TAG** (**3au**) was less efficient than others, requiring slightly higher amounts of reagents and electricity. It should be noted that the protecting groups commonly employed in conventional peptide synthesis were proven to be compatible with the reaction, and therefore, commercially available building blocks could be used directly in this method.

**Table 3:** Scope of tag-protected amino acids

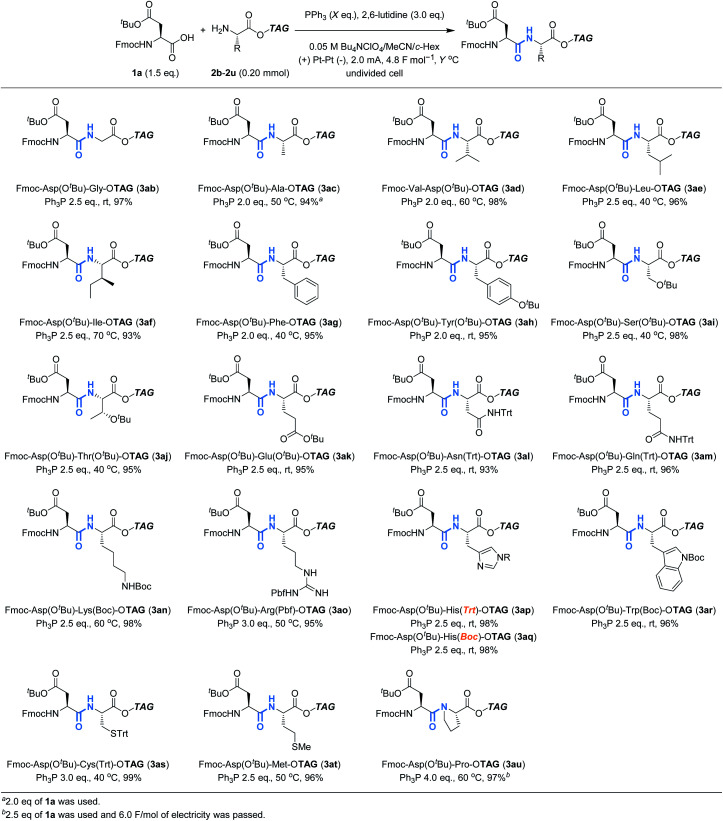

**Scheme 2:**
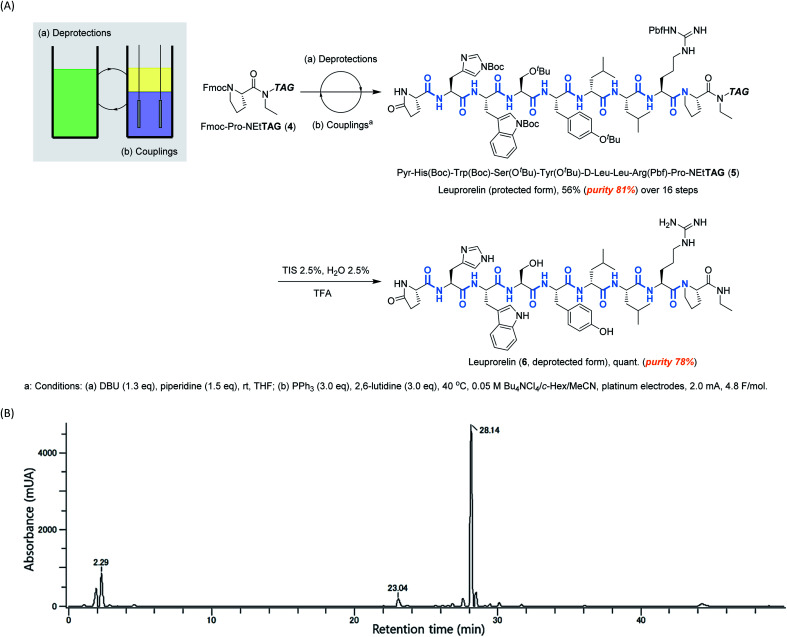
(A) Biphasic electrochemical synthesis of leuprorelin, and (B) HPLC analysis of leuprorelin (protected form, R. T. = 28.14) (**5**).

Having confirmed that electrochemical peptide bond formation can be applied to all canonical amino acids, we applied this method to the synthesis of a commercial peptide active pharmaceutical ingredient (API), Leuprorelin, as a model (Scheme 2A, see ESI† for experimental details).^14^ Starting from Fmoc-Pro-NEt**TAG** (**4**),^10*d*^ all 8 peptide bonds were electrochemically formed. Deprotections of Fmoc were carried out under typical conditions using DBU and piperidine. After repetitive deprotections and couplings, the protected form of leuprorelin (**5**) was successfully obtained in 45% yield over 16 steps (56% by weight, and its purity was estimated to be 81% by HPLC, Scheme 2B and Fig. S2†). The average yield in each step was >95%. Finally, acidic global deprotection, which is commonly used in conventional peptide synthesis, afforded leuprorelin (**6**) quantitatively (Fig. S3†). This result demonstrates that electrochemical peptide synthesis has the potential to be an alternative choice for conventional SPPS and/or LPPS.

## Conclusions

We succeeded in developing a biphasic electrochemical peptide bond formation reaction using Ph_3_P. Electrochemically generated Ph_3_P˙^+^ promoted efficient peptide bond formation with no significant epimerization. Protecting groups commonly employed in conventional peptide synthesis were proven to be compatible with the reaction, and therefore, commercially available building blocks could be used directly in this method. In combination with a soluble tag-assisted LPPS, the synthesis of a commercial peptide API, leuprorelin, was achieved without the use of traditional coupling reagents. Although using Ph_3_P as a coupling reagent under electrochemical conditions results in the generation of Ph_3_P=O, the biphasic system enabled selective recovery of the desired peptides and Ph_3_P=O by simple phase separation. Given that methods to reduce Ph_3_PO to Ph_3_P have been reported, Ph_3_P is a potentially recyclable coupling reagent. Therefore, this soluble tag-assisted electrochemical method can be used to address sustainability challenges in peptide synthesis. Aiming for further improvement, we are exploring novel methods to reduce Ph_3_P=O to Ph_3_P.

## Data availability

The datasets supporting this article have been uploaded as part of the ESI.†

## Author contributions

K. C. conceived and directed the project. S. N. designed and performed the experiments. S. N., Y. O., Y. K., and K. C. discussed results. S. N. and Y. O. wrote the manuscript.

## Conflicts of interest

There are no conflicts to declare.

## Supplementary Material

SC-012-D1SC03023J-s001

## References

[cit1] Bryan M. C., Dunn P. J., Entwistle D., Gallou F., Koenig S. G., Hayler J. D., Hickey M. R., Hughes S., Kopach M. E., Moine G., Richardson P., Roschangar F., Steven A., Weiberth F. J. (2018). Green Chem..

[cit2] Lau J. L., Dunn M. K. (2018). Bioorg. Med. Chem..

[cit3] El-Faham A., Albericio F. (2011). Chem. Rev..

[cit4] Trost B. M. (1991). Science.

[cit5] Ishihara K., Ohara S., Yamamoto H. (1996). J. Org. Chem..

[cit6] Barstow L. E., Hruby V. J. (1971). J. Org. Chem..

[cit7] Hérault D., Nguyen D. H., Nuel D., Buono G. (2015). Chem. Soc. Rev..

[cit8] Palma A., Cardenas J., Frontana-Uribe B. A. (2009). Green Chem..

[cit9] Kärkäs M. D. (2018). Chem. Soc. Rev..

[cit10] Okada Y., Takasawa R., Kubo D., Iwanaga N., Fujita S., Suzuki K., Suzuki H., Kamiya H., Chiba K. (2019). Org. Process Res. Dev..

[cit11] Shida N., Imada Y., Nagahara S., Okada Y., Chiba K. (2019). Commun. Chem..

[cit12] Okada Y., Kamimura K., Chiba K. (2012). Tetrahedron.

[cit13] Jones J. H., Ramage W. I., Witty M. J. (1980). Int. J. Pept. Protein Res..

[cit14] Fujino M., Fukuda T., Shinagawa S., Kobayashi S., Yamazaki I., Nakayama R. (1974). Biochem. Biophys. Res. Commun..

