# Supplementary material for: Biphasic electrochemical peptide synthesis
Source: Chem Sci. 2021 Sep 2;12(39):12911–7. doi: 10.1039/d1sc03023j (PMC8513919; doi:10.1039/d1sc03023j)
Supplement: SC-012-D1SC03023J-s001 [file SC-012-D1SC03023J-s001.pdf]

Electronic Supplementary Material (ESI) for Chemical Science.
This journal is © The Royal Society of Chemistry 2021

Supporting Information

Biphasic Electrochemical Peptide Synthesis

Shingo Nagahara,[a] Yohei Okada,[a] Yoshikazu Kitano,[a] and Kazuhiro Chiba*[a]

[a] Department of Applied Biological Science, Tokyo University of Agriculture and Technology, 3-5-8 Saiwai-cho, Fuchu, Tokyo 183-8509, Japan
*E-mail: chiba@cc.tuat.ac.jp

Table of Contents

1. Additional Figure……………………………………………………………S2

2. Experimental Section…………………………………………………………..S3

3. ^{1}H and ^{13}C NMR Spectra of Compounds……………………………………….S57

1. Additional Figures

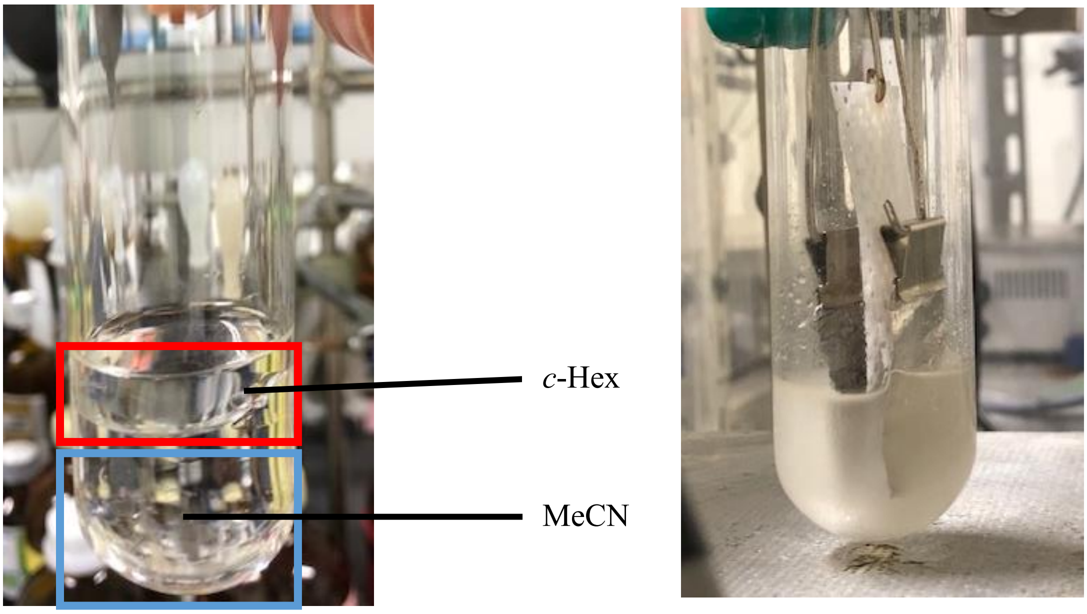

Figure S1. Biphasic Reaction System; Before electrolysis (left), and during electrolysis (right).

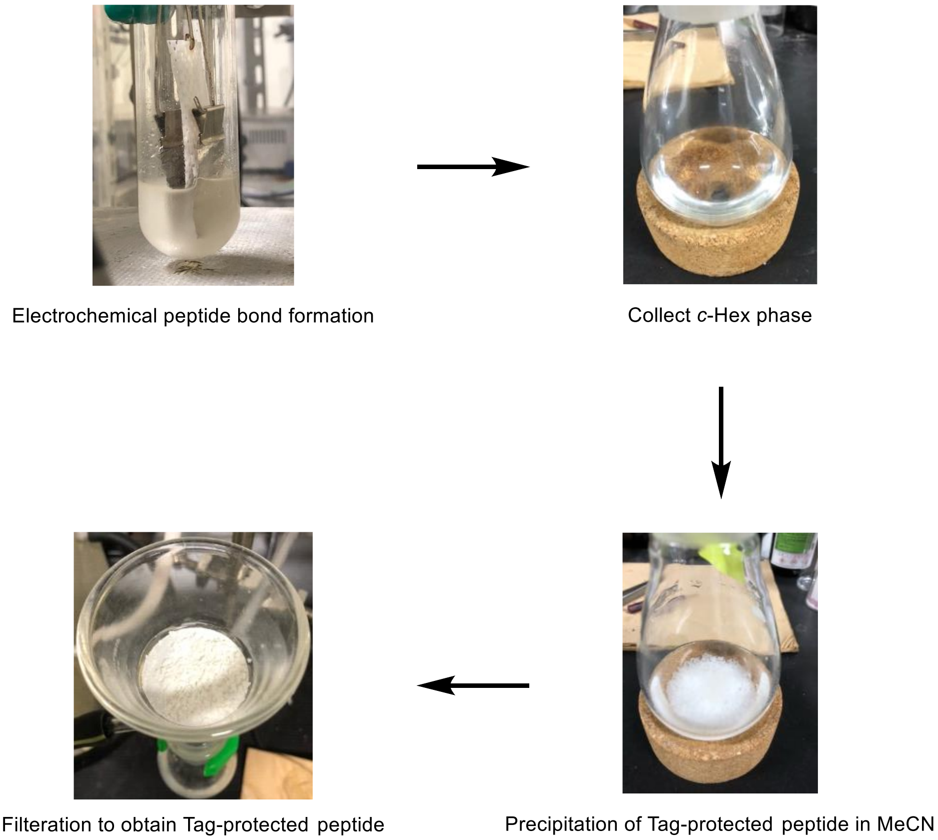

Scheme S1. The Process of Biphasic Electrochemical Peptide Synthesis.

2. Experimental Section

2.1 General Information

All reagents and solvents were purchased from commercial sources and used further purification. Reactions were monitored by thin-layer chromatography carried out on silica gel plates, with detection by UV absorption (254 nm) and by heating the plates after dipping them in a solution of 12 molybdol (VI) phosphoric acid n-hydrate in 95% ethanol or ninhydrin in 97% *n*-butanol solution (2.9 % AcOH). Silica gel (particle size 40- 50 nm) was used for column chromatography. ^{1}H NMR spectra were collected on a 600 or 400 MHz NMR spectrometer using the deuterated solvent as an internal deuterium reference. Chemical shift data are given in δ units calibrated with residual protic solvent. The multiplicity of a signal is indicated as follows: s, singlet; d, doublet; triplet; q, quartet; quin, quintet, m, multiplet. ^{13}C NMR spectra were collected on a 150 or 100MHz spectrometer with proton decoupling using the deuterated solvent as an internal carbon reference. Chemical shift data are given in δ units calibrated with residual solvent.

2.2 General Procedure for Synthesis of Tag-Protected Amino Acids

To a solution of tag (0.50 mmol) in DCM (10 mL), Fmoc-protected amino acid (0.75 mmol), DMAP (0.10 mmol) and DIPCI (0.75 mmol) were added. The resulting mixture was stirred for 15 min at r.t. After completion, MeCN was added to reaction solution, and concentrated under vacuum. To remaining solids, MeCN was added and filtered to give soluble tag protected amino acid as white solid quantitatively.

2.3 General Procedure for Deprotection of Fmoc Group

To a solution of Fmoc protected tagged amino acid (0.50 mmol) in THF (10 mL),

piperidine (74.2 μL, 0.75 mmol) and DBU (1% v/v) were added. The reaction mixture was stirred for 10 min at r.t. After completion, 6 M HCl aq. was added to the solution to neutralize (pH 7.0), and then MeCN was added followed by concentration under vacuum. To remaining solid, MeCN was added and filtered to give *N*-deprotected amino acid quantitatively as white solid.

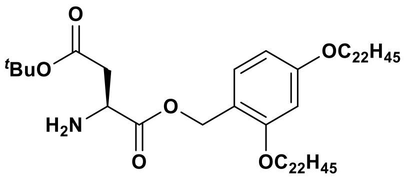

H_2N-Asp(O^{t}Bu)-Tag (**2a**). ^{1}H NMR ($CDCl_3$, 400 MHz) δ 7.19 (1H, d, *J* = 7.6 Hz), 6.45-6.40 (2H, m), 5.16 (1H, d, *J* = 11.7 Hz), 5.12 (1H, d, *J* = 11.7 Hz), 3.93 (4H, t, *J* = 6.9 Hz), 3.76 (1H, dd, *J* = 7.6 Hz, 4.8 Hz), 2.72 (1H, dd, *J* = 16.5 Hz, 4.8 Hz), 2.60 (1H, dd, *J* = 16.5 Hz, 7.6 Hz), 1.81-1.68 (6H, m), 1.46-1.21 (85H, m), 0.88 (6H, t, *J* = 6.9 Hz); ^{13}C NMR ($CDCl_3$, 100 MHz) δ 174.5, 170.4, 160.8, 158.4, 131.2, 116.2, 104.5, 99.6, 81.1, 68.1, 62.6, 51.4, 40.1, 31.9, 29.7, 29.6, 29.6, 29.4, 29.4, 29.3, 29.2, 28.0, 26.1, 22.7, 14.1; HRMS (ESI-MS) $[M+Na]^+$ calcd for $C_{59}H_{109}NO_6$ 950.8147, found 950.8136.

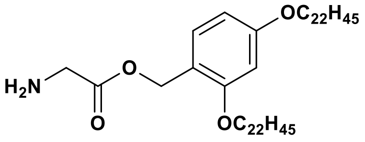

H_2N-Gly-Tag (**2b**). ^{1}H NMR ($CDCl_3$, 400 MHz) δ 7.20 (1H, d, *J* = 8.2 Hz), 6.46-6.40 (2H, m), 5.15 (2H, s), 3.98-3.90 (4H, m), 3.51-3.33 (2H, s), 1.81-1.71 (4H, m), 1.63-1.59 (2H, s), 1.49-1.20 (76H, m), 0.88 (6H, t, *J* = 6.9 Hz); ^{13}C NMR ($CDCl_3$, 100 MHz) δ 160.9, 158.5, 131.4, 116.2, 104.6, 99.8, 68.1, 62.4, 44.1, 31.9, 29.7, 29.6, 29.4, 29.3, 29.2, 26.1, 22.7, 14.1; HRMS

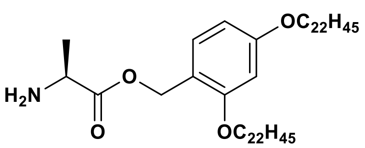

(ESI-MS) [M+Na]$^+$ calcd for $C_{53}H_{99}NO_4$ 836.7466, found 836.7483.

H$_2$N-Ala-Tag (**2c**). ^{1}H NMR (CDCl$_3$, 400 MHz) δ 7.19 (1H, d, J = 8.2 Hz), 6.46-6.40 (2H, m), 5.14 (1H, d, J = 11.9 Hz), 5.10 (1H, d, J = 11.9 Hz), 3.94 (4H, t, J = 6.4 Hz), 3.54 (1H, q, J = 6.9 Hz), 1.82-1.71 (4H, m), 1.68 (2H, s), 1.47-1.21 (79H, m), 0.88 (6H, t, J = 6.9 Hz); ^{13}C NMR (CDCl$_3$, 100 MHz) δ 176.7, 160.9, 158.5, 131.2, 116.4, 104.5, 99.7, 68.1, 62.4, 50.2, 31.9, 29.7, 29.6, 29.4, 29.3, 29.2, 26.1, 22.7, 20.6, 14.1; HRMS (ESI-MS) [M+Na]$^+$ calcd for $C_{54}H_{101}NO_4$ 850.7617, found 850.7603.

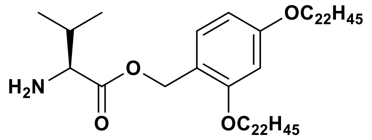

H$_2$N-Val-Tag (**2d**). ^{1}H NMR (CDCl$_3$, 400 MHz) δ 7.20 (1H, d, J = 8.7 Hz), 6.46-6.40 (2H, m), 5.14 (1H, d, J = 11.9 Hz), 5.10 (1H, d, J = 11.9 Hz), 3.94 (4H, t, J = 6.4 Hz), 3.29 (1H, d, J = 4.6 Hz), 2.08-1.98 (1H, m), 1.84-1.70 (4H, m), 1.65-1.58 (2H, m), 1.51-1.18 (76H, m), 0.95 (3H, d, J = 6.9 Hz), 0.92-0.84 (9H, m); ^{13}C NMR(CDCl$_3$, 100 MHz) δ 175.7, 160.9, 158.6, 131.4, 116.4, 104.5, 99.7, 68.1, 62.2, 60.0, 32.1, 31.9, 29.7, 29.6, 29.4, 29.4, 29.3, 29.2, 26.1, 22.7, 19.3, 17.2, 14.1; HRMS (ESI-MS) [M+Na]$^+$ calcd for $C_{56}H_{105}NO_4$ 878.7936, found 878.7946.

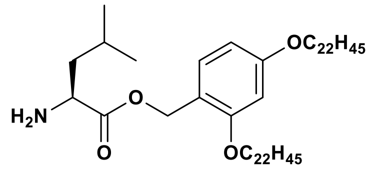

H$_2$N-Leu-Tag (**2e**). ^{1}H NMR (CDCl$_3$, 400 MHz) δ 7.19 (1H, d, J = 8.7 Hz), 6.46-6.40 (2H, m), 5.11 (2H. s), 3.94 (4H, t, J = 6.4 Hz), 3.46 (1H, dd, J = 8.7 Hz, 5.5 Hz), 1.81-1.71

(4H, m), 1.62-1.49 (4H, m), 1.49-1.19 (77H, m), 0.94-0.84 (12H, m); ^{13}C NMR ($CDCl_3$, 100 MHz) δ 176.8, 160.8, 158.5, 131.2, 116.4, 104.5, 99.7, 68.1, 62.3, 53.0, 44.0, 31.9, 29.7, 29.6, 29.4, 29.4, 29.3, 29.2, 26.1, 24.7, 23.0, 22.7, 21.9, 14.1; HRMS (ESI-MS) $[M+Na]^+$ calcd for $C_{57}H_{107}NO_4$ 892.8092, found 892.8092..

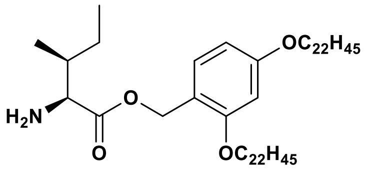

H_2N-Ile-Tag (**2f**). ^{1}H NMR ($CDCl_3$, 400 MHz) δ 7.20 (1H, d, J = 8.7 Hz), 6.46-6.40 (2H, m), 5.13 (1H, d, J = 11.5 Hz), 5.10 (1H, d, J = 11.5 Hz), 3.94 (4H, t, J = 6.4 Hz), 3.34 (1H, d, J = 5.0 Hz), 1.82-1.68 (5H, m), 1.61 (2H, s), 1.51-1.20 (77H, m), 1.2-1.07 (1H, m), 0.94-0.82 (12H, m); ^{13}C NMR ($CDCl_3$, 100 MHz) δ 175.8, 160.8, 158.5, 131.4, 116.4, 104.5, 99.7, 68.1, 62.1, 59.2, 39.1, 31.9, 29.7, 29.6, 29.4, 29.3, 29.2, 26.1, 24.6, 22.7, 15.7, 14.1, 11.7; HRMS (ESI-MS) $[M+Na]^+$ calcd for $C_{57}H_{107}NO_4$ 892.8092, found 892.8113.

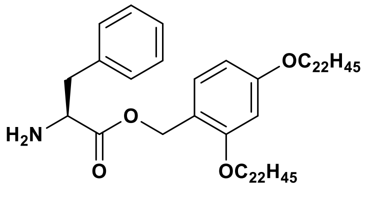

H_2N-Phe-Tag (**2g**). ^{1}H NMR ($CDCl_3$, 400 MHz) δ 7.29-7.11 (6H, m), 6.47-6.39 (2H, m), 5.14 (2H, s), 3.95 (4H, t, J = 6.4 Hz), 3.73 (1H, dd, J = 7.8 Hz, 5.0 Hz), 3.08 (1H, dd, J = 13.7 Hz, 5.0 Hz), 2.87 (1H, dd, J = 13.7 Hz, 7.8 Hz), 1.82-1.72 (4H, m), 1.55 (2H, s), 1.50-1.20 (76H, m), 0.88 (6H, t, J = 6.9 Hz); ^{13}C NMR ($CDCl_3$, 100 MHz) δ 175.2, 160.9, 158.6, 137.3, 131.5, 129.4, 128.5, 126.7, 116.2, 104.5, 99.7, 68.1, 62.4, 55.8, 40.9, 31.9, 29.7, 29.6, 29.6, 29.4, 29.4, 29.3, 29.2, 26.1, 22.7, 14.1; HRMS (ESI-MS) $[M+Na]^+$

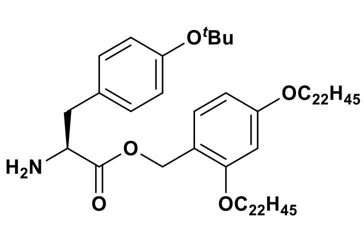

calcd for $C_{60}H_{105}NO_4$ 926.7936, found 926.7952.

H_2N-Tyr(O^{t}Bu)-Tag (**2h**). ^{1}H NMR ($CDCl_3$, 400 MHz) δ 7.16 (1H, d, *J* = 8.2 Hz), 7.03 (2H, d, *J* = 8.7 Hz), 6.87 (2H, d, *J* = 8.7 Hz), 6.46-6.39 (2H. m), 5.13 (2H, s), 3.99-3.90 (4H, m), 3.70 (1H, dd, *J* = 7.7 Hz, 5.0 Hz), 3.03 (1H, dd, *J* = 13.7 Hz, 5.0 Hz), 2.83 (1H, dd, *J* = 13.7 Hz, 7.3 Hz), 1.82-1.71 (4H, m), 1.58 (2H, s), 1.48-1.20 (85H, m), 0.88 (6H, t, *J* = 7.1 Hz); ^{13}C NMR ($CDCl_3$, 100 MHz) δ 17517, 160.9, 158.6, 154.2, 132.0, 131.4, 129.8, 124.1, 116.3, 104.5, 99.7, 78.3, 68.1, 62.4, 55.8, 40.2, 31.9, 29.7, 29.4, 29.4, 29.3, 29.2, 28.9, 26.1, 22.7, 14.1; HRMS (ESI-MS) $[M+Na]^+$ calcd for $C_{64}H_{113}NO_5$ 998.8511, found 998.8529.

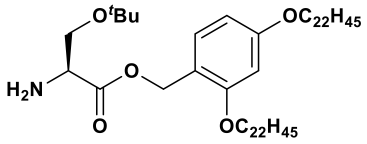

H_2N-Ser(O^{t}Bu)-Tag (**2i**). ^{1}H NMR ($CDCl_3$, 400 MHz) δ 7.21 (1H, d, *J* = 8.3 Hz), 6.45-6.40 (2H, m), 5.18 (1H, d, *J* = 12.4 Hz), 5.13 (1H, d, *J* = 12.4 Hz), 3.94 (4H, t, *J* = 6.9 Hz), 3.64-3.54 (3H, m), 1.80-1.71 (6H, m), 1.47-1.22 (76H, m), 1.13 (9H, s), 0.88 (6H, t, *J* = 6.9 Hz); ^{13}C NMR ($CDCl_3$, 100 MHz) δ 174.2, 160.7, 158.4, 131.0, 116.5, 104.5, 99.6, 73.0, 68.1, 63.7, 62.2, 55.3, 31.9, 29.7, 29.6, 29.6, 29.4, 29.4, 29.3, 29.2, 27.4, 26.1, 22.7, 14.1; HRMS (ESI-MS) $[M+Na]^+$ calcd for $C_{64}H_{109}NO_5$ 922.8198, found 922.8188.

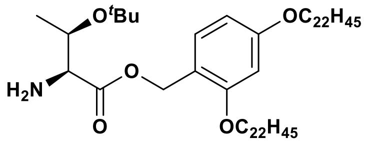

H_2N-Thr(O^{t}Bu)-Tag (**2j**). ^{1}H NMR ($CDCl_3$, 400 MHz) δ 7.21 (1H, d, *J* = 8.3 Hz), 6.45-6.41 (2H, m), 5.20 (1H, d, *J* = 11.7 Hz), 5.01 (1H, d, *J* = 11.7 Hz), 4.01 (1H, td, *J* = 6.2 Hz, 3.4 H), 3.97-3.91 (4H, m), 3.26(1H, d, *J* = 2.8 Hz), 1.80-1.74 (4H, m), 1.63 (2H, s), 1.47-1.20 (79H, m), 1.12 (9H, s), 0.88 (6H, t, *J* = 6.9 Hz); ^{13}C NMR ($CDCl_3$, 100 MHz) δ 174.9, 160.9, 158.5, 131.5, 116.3, 104.5, 99.7, 73.6, 68.5, 68.1, 62.3, 60.7, 31.9, 29.7, 29.6, 29.6, 29.4, 29.4, 29.3, 29.2, 28.5, 26.1, 22.7, 21.0, 14.1; HRMS (ESI-MS) $[M+Na]^+$ calcd for $C_{59}H_{111}NO_5$ 936.8354, found 936.8374.

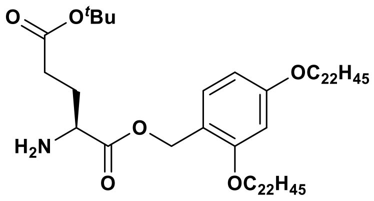

H_2N-Glu(O^{t}Bu)-Tag (**2k**). ^{1}H NMR ($CDCl_3$, 400 MHz) δ 7.19 (1H, d, *J* = 8.7 Hz), 6.45-6.40 (2H, m), 5.13 (2H, s), 3.94 (4H, t, *J* = 6.4 Hz), 3.45 (1H, dd, *J* = 8.4 Hz, 4.8 Hz), 2.33 (2H, t, *J* = 7.3 Hz), 1.80-1.91 (1H, m), 1.87-1.71 (5H, m), 1.60 (2H, s), 1.48-1.18 (85H, m), 0.88 (6H, t, *J* = 6.9 Hz); ^{13}C NMR ($CDCl_3$, 100 MHz) δ 175.7, 172.4, 160.9, 158.5, 131.2, 116.3, 104.5, 99.7, 80.3, 68.1, 62.4, 54.0, 31.9, 31.9, 30.0, 29.7, 29.4, 29.3, 29.2, 28.1, 26.1, 22.7, 14.1; HRMS (ESI-MS) $[M+Na]^+$ calcd for $C_{60}H_{111}NO_6$ 964.8304, found 964.8309.

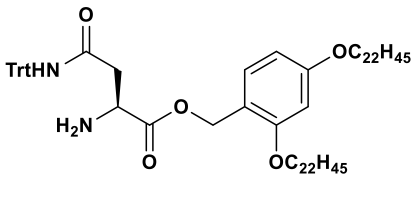

H_2N-Asn(Trt)-Tag (**2l**). ^{1}H NMR ($CDCl_3$, 400 MHz) δ 8.72 (1H, s), 7.29-7.17 (15H, m), 7.15 (1H, d, *J* = 8.2 Hz), 6.43-6.37 (2H, m), 5.16 (1H, d, *J* = 11.9 Hz), 5.11 (1H, d, *J* =

11.9 Hz), 3.92 (4H, t, J = 6.4 Hz), 3.83 (1H, dd, J = 9.6 Hz, 2.8 Hz), 2.68 (1H, dd, J = 15.6 Hz, 3.2 Hz), 2.51 (1H, dd, J = 16.0 Hz, 9.6 Hz), 1.81-1.70 (4H, m), 1.65 (2H, s), 1.48-1.21 (76H, m), 0.88 (6H, t, J = 6.9 Hz); ^{13}C NMR ($CDCl_3$, 100 MHz) δ 174.2, 169.3, 160.9, 158.4, 144.9, 131.2, 128.7, 127.9, 126.9, 115.9, 104.6, 99.7, 70.4, 68.2, 62.8, 51.7, 40.7, 31.9, 29.7, 29.6, 29.4, 29.4, 29.3, 29.2, 26.1, 22.7, 14.1; HRMS (ESI-MS) $[M+Na]^+$ calcd for $C_{74}H_{116}N_2O_5$ 1135.8776, found 1135.8770.

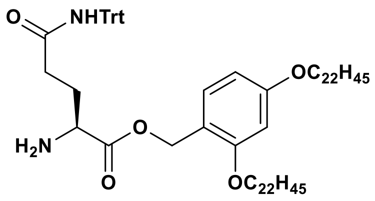

H_2N-Gln(Trt)-Tag (**2m**). ^{1}H NMR ($CDCl_3$, 400 MHz) δ 7.29-7.14 (16H, m), 7.05 (1H, s), 6.42-6.36 (2H, m), 5.14 (1H, d, J = 11.9 Hz), 5.09 (1H, d, J = 11.9 Hz), 3.94-3.84 (4H, m), 3.42 (1H, dd, J = 8.2 Hz, 5.0 Hz), 2.46-2.31 (2H, m), 2.11-2.01 (1H, m), 1.89-1.69 (5H, m), 1.64 (2H, s), 1.50-1.19 (76H, m), 0.88 (6H, t, J = 6.9 Hz); ^{13}C NMR ($CDCl_3$, 100 MHz) δ 175.5, 171.3, 160.9, 158.4, 144.7, 131.3, 128.7, 127.9, 126.9, 116.2, 104.6, 99.8, 70.4, 68.2, 68.1, 62.5, 53.9, 33.9, 31.9, 30.1, 29.7, 29.7, 29.6, 29.4, 29.4, 29.3, 29.2, 26.1, 26.0, 22.7, 14.1; HRMS (ESI-MS) $[M+Na]^+$ calcd for $C_{75}H_{118}N_2O_5$ 1149.8933, found 1149.8945.

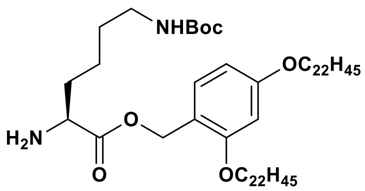

H_2N-Lys(Boc)-Tag (**3n**). ^{1}H NMR ($CDCl_3$, 400 MHz) δ 7.19 (1H, d, J = 9.2 Hz), 6.45-6.41 (2H, m), 5.12 (2H, s), 4.51 (1H, s), 3.94 (4H, t, J = 6.4 Hz), 3.42 (1H, dd, J = 7.7 Hz,

5.0 Hz), 3.08 (2H, m), 1.81-1.71 (5H, m), 1.58 (2H, s), 1.51-1.21 (90H, m), 0.88 (6H, t, J = 6.9 Hz); ^{13}C NMR ($CDCl_3$, 100 MHz) δ 176.1, 160.9, 158.5, 155.9, 131.2, 116.4, 104.6, 99.7, 68.1, 62.3, 54.5, 40.4, 34.4, 31.9, 29.7, 29.6, 29.6, 29.4, 29.4, 29.3, 29.2, 28.4, 26.1, 22.9, 22.7, 14.1; HRMS (ESI-MS) $[M+Na]^+$ calcd for $C_{62}H_{116}N_2O_6$ 1007.8726, found 1007.8723.

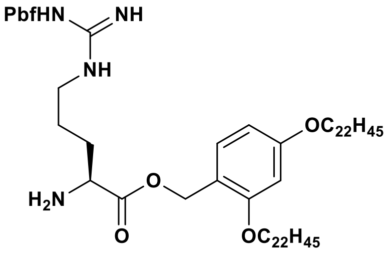

H_2N-Arg(Pbf)-Tag (**3o**). ^{1}H NMR ($CDCl_3$, 600 MHz) δ 7.17 (1H, d, J = 8.3 Hz), 6.44-6.35 (3H, m), 6.31 (2H, s), 5.12 (1H, d, J = 11.7 Hz), 5.09 (1H, d, J = 11.7 Hz), 3.93 (4H, t, J = 6.5 Hz), 3.47-3.43 (1H, m), 3.20-3.06 (2H, m), 2.93 (2H, s), 2.56 (3H, s), 2.50 (3H, s), 2.08 (3H, s), 1.81-1.51 (9H, m), 1.47-1.22 (83H, s), 0.88 (6H, t, J = 7.2 Hz); ^{13}C NMR ($CDCl_3$, 150 MHz) δ 175.5, 160.9, 158.6, 158.4, 156.3, 138.3, 133.1, 132.3, 131.3, 124.5, 117.4, 116.1, 104.6, 99.7, 86.3, 68.2, 62.5, 53.9, 43.2, 40.7, 31.9, 29.7, 29.7, 29.7, 29.6, 29.5, 29.4, 29.3, 29.2, 28.6, 26.1, 26.0, 25.5, 22.7, 19.3, 17.9, 14.1, 12.5; HRMS (ESI-MS) $[M+Na]^+$ calcd for $C_{70}H_{124}N_4O_7S$ 1187.9083, found 1187.9077.

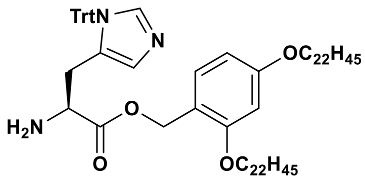

H_2N-His(Trt)-Tag (**3p**). ^{1}H NMR ($CDCl_3$, 400 MHz) δ 7.35 (1H, d, J = 1.4 Hz), 7.34-7.28 (9H, m), 7.14-7.09 (6H, m), 7.07 (1H, d, J = 8.2 Hz), 6.57 (1H, d, J = 1.4 Hz), 6.40 (1H, d, J = 2.3 Hz), 6.33 (1H, dd, J = 8.2 Hz, 2.3 Hz), 5.13 (1H, d, J = 11.9 Hz), 4.97 (1H, d, J = 11.9 Hz), 3.95-3.85 (4H, m), 3.77-3.71 (1H, m), 3.00 (1H, dd, J = 14.7 Hz, 4.2 Hz),

2.84 (1H, dd, J = 14.7 Hz, 7.3 Hz), 1.81-1.70 (6H, m), 1.48-1.20 (76H, m), 0.88 (6H, t, J = 6.9 Hz); ^{13}C NMR (CDCl$_3$, 100 MHz) δ175.1, 160.6, 158.3, 142.4, 138.6, 137.4, 130.9, 129.8, 128.0, 128.0, 119.5, 116.4, 104.5, 99.7, 75.2, 68.1, 68.0, 62.2, 54.9, 33.3, 31.9, 29.7, 29.6, 29.4, 29.3, 29.2, 29.2, 26.1, 26.0, 25.6, 22.7, 14.1; HRMS (ESI-MS) $[M+H]^+$ calcd for $C_{76}H_{117}N_3O_4$ 1136.9117, found 1136.9132.

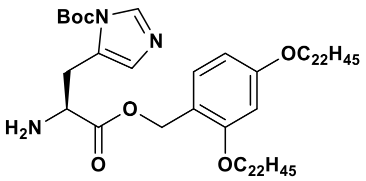

H$_2$N-His(Boc)-Tag (**3q**). ^{1}H NMR (CDCl$_3$, 600 MHz) δ 8.00-7.95 (1H, s), 7.18 (1H, d, J = 8.3 Hz), 7.13 (1H, s), 6.44-6.40 (2H, m), 5.16 (1H, d, J = 12.4 Hz), 5.13 (1H, d, J = 12.4 Hz), 3.94 (4H, t, J = 6.4 Hz), 3.84 (1H, dd, J = 8.3 Hz, 4.8 Hz), 3.04 (1H, dd, J = 14.4 Hz, 4.9 Hz), 2.81 (1H, J = 14.4 Hz, 8.3 Hz), 1.80-1.73 (4H, m), 1.70 (2H, s), 1.60 (9H, m), 1.47-1.20 (76H, m), 0.88 (6H, t, J = 6.9 Hz); ^{13}C NMR (CDCl$_3$, 151 MHz) δ 175.1, 160.8, 158.4, 146.9, 139.7, 136.8, 131.1, 116.3, 114.5, 104.5, 99.7, 85.4, 68.1, 68.1, 62.4, 54.2, 33.1, 31.9, 29.7, 29.7, 29.6, 29.6, 29.4, 29.4, 29.3, 29.2, 27.9, 26.1, 22.7, 14.1; HRMS (ESI-MS) $[M+H]^+$ calcd for $C_{62}H_{111}N_3O_6$ 994.8546, found 994.8528.

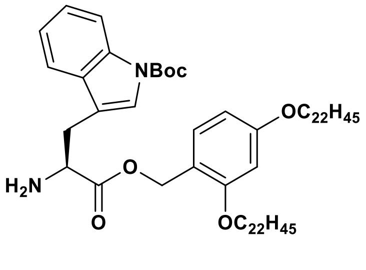

H$_2$N-Trp(Boc)-Tag (**3r**). ^{1}H NMR (CDCl$_3$, 400 MHz) δ 8.12 (1H, d, J = 6.9 Hz), 7.54 (1H, d, J = 7.8 Hz), 7.46 (1H, s), 7.30 (1H, m), 7.21 (1H, m), 7.11 (1H, d, J = 8.2 Hz), 6.45-6.38 (2H, m), 5.16 (1H, d, J = 11.9 Hz), 5.11 (1H, d, J = 11.9 Hz), 3.94 (4H, t, J = 6.4 Hz), 3.83 (1H, dd, J = 7.8 Hz, 4.6 Hz), 3.21 (1H, dd, J = 14.2 Hz, 4.6 Hz), 2.95 (1H,

dd, *J* = 14.6 Hz, 8.2 Hz), 1.81-1.70 (4H, m), 1.65 (9H, s), 1.64-1.58 (2H, s), 1.51-1.20 (76H, m), 0.88 (6H, t, *J* = 6.9 Hz); ^{13}C NMR (CDCl$_3$, 100 MHz) δ 175.1, 160.9, 158.5, 149.6, 135.5, 131.2, 130.5, 127.1, 124.4, 124.1, 122.5, 119.0, 116.2, 115.3, 104.5, 99.7, 83.5, 68.1, 68.1, 62.6, 54.5, 31.9, 30.5, 29.7, 29.6, 29.4, 29.4, 29.3, 29.2, 28.2, 26.1, 22.7, 14.1; HRMS (ESI-MS) [M+Na]$^+$ calcd for $C_{67}H_{114}N_2O_6$ 1065.8569, found 1065.8563.

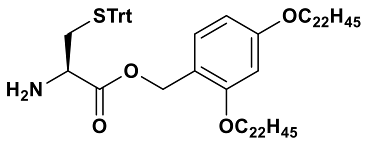

H$_2$N-Cys(Trt)-Tag (**3s**). ^{1}H NMR (CDCl$_3$, 400 MHz) δ 7.42-7.36 (6H, m), 7.27-7.09 (10H, m), 6.43-6.38 (2H, m), 5.07 (2H, m), 3.94 (2H, t, *J* = 6.4 Hz), 3.87 (2H, t, *J* = 6.4 Hz), 3.20 (1H, dd, *J* = 8.2 Hz, 4.6 Hz), 2.56 (1H, dd, *J* = 12.4 Hz, 4.6 Hz), 2.46 (1H, dd, *J* = 12.4 Hz, 7.8 Hz), 1.82-1.66 (4H, m), 1.60-1.18 (78 H, m), 0.88 (6H, t, *J* = 6.9 Hz); ^{13}C NMR (CDCl$_3$, 100 MHz) δ 173.8, 160.8, 158.3, 144.6, 131.0, 129.6, 129.6, 127.9, 127.8, 126.7, 126.6, 116.3, 104.5, 99.6, 68.1, 66.8, 62.4, 54.0, 37.0, 31.9, 29.7, 29.4, 29.4, 29.3, 29.2, 26.1, 26.0, 22.7, 14.1; HRMS (ESI-MS) [M+Na]$^+$ calcd for $C_{73}H_{115}NO_4S$ 1124.8439, found 1124.8461..

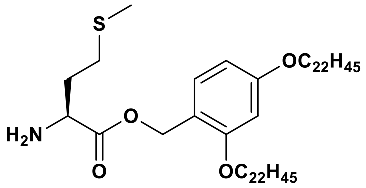

H$_2$N-Met-Tag (**3t**). ^{1}H NMR (CDCl$_3$, 400 MHz) δ 7.20 (1H, d, *J* = 8.2 Hz), 6.45-6.39 (2H, m), 5.16 (1H, d, *J* = 11.5 Hz), 5.11 (1H, d, *J* = 11.5 Hz), 3.94 (4H, t, *J* = 6.4 Hz), 3.60 (1H, dd, *J* = 8.2 Hz, 5.0 Hz), 2.58 (2H, t, *J* = 7.3 Hz), 2.1-1.98 (4H, m), 1.94-1.70 (7H, m), 1.48-1.20 (76H, m), 0.88 (6H, t, *J* = 6.6 Hz); ^{13}C NMR (CDCl$_3$, 100 MHz) δ 175.5, 160.9, 158.6, 131.4, 116.2, 104.5, 99.7, 68.1, 62.6, 53.5, 33.8, 31.9, 30.4, 29.7, 29.6, 29.4,

29.4, 29.3, 29.2, 26.1, 22.7, 15.4, 14.1; HRMS (ESI-MS) $[M+Na]^+$ calcd for $C_{56}H_{105}NO_4S$ 910.7657, found 910.7672.

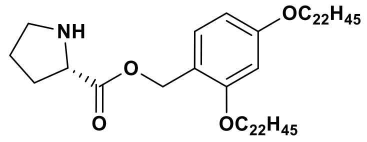

H_2N-Pro-Tag (**3u**). ^{1}H NMR ($CDCl_3$, 400 MHz) δ 7.19 (1H, d, J = 8.2 Hz), 6.45-6.41 (2H, m), 5.15 (1H, d, J = 11.9 Hz), 5.11 (1H, d, J = 11.9 Hz), 3.94 (4H, t, J = 6.4 Hz), 3.77 (1H, dd, J = 8.8 Hz, 6.0 Hz), 3.12-3.04 (1H, m), 2.93-2.85 (1H, m), 2.16-2.05 (1H, m), 1.90-1.70 (8H, m), 1.48-1.21 (76H, m), 0.88 (6H, t, J = 6.7 Hz); ^{13}C NMR ($CDCl_3$, 100 MHz) δ 175.5, 160.9, 158.5, 131.2, 116.4, 104.5, 99.7, 68.1, 62.5, 59.8, 47.0, 31.9, 30.3, 30.0, 29.9, 29.7, 29.6, 29.5, 29.4, 29.3, 29.2, 26.1, 25.4, 22.71, 14.1; HRMS (ESI-MS) $[M+Na]^+$ calcd for $C_{56}H_{103}NO_4$ 876.7779, found 876.7798.

2.4 General Procedure for Electrochemical Peptide Bond Formation

Solvent was purged with Ar gas for 2 min. To a purged solvent, 2,6-lutidine (69.9 μL, 0.60 mmol), electrolyte (0.40 mmol), Ph_3P (0.40-0.80 mmol), Fmoc-protected amino acid (0.30-0.50 mmol), and *N*-deprotected tagged amino acid (0.20 mmol) were added. The solution was electrolyzed at constant current (0.67 mA/cm^2) under Ar at rt-60 °C. After 4.8-6.0 F/mol of electricity based on *N*-deprotected tagged amino acid was passed, the reaction mixture was extracted with *c*-Hex (20 mL x 2), and then the solvent was replaced with MeCN. The resulting precipitants were collected by filtration and washed with MeCN to give the peptides.

2.5 Recovery of Bu_4NClO_4 and Ph_3PO

After the following the procedure **2.4**, filtrate and MeCN layer was combined followed by treatment with activated carbon. The activated carbon was removed with filtration and the resulting solution was concentrated. To the residue, toluene-*c*-Hex (30:1) 20 mL was added to afford Bu_4NClO_4 as a precipitate (quant.). After the collection of Bu_4NClO_4 by filtration, DBU (20 μL) and piperidine (20 μL) was added to the filtrate to deprotect Fmoc group, and stirred for 10 min at room temperature followed by addition of 1 M HCl (10 mL). Ph_3P=O was extracted with toluene (15 mL) x 2, and combined organic phase was washed with 1 M HCl (20 mL) and brine (20 mL). The organic phase was dried over anhydrous Na_2SO_4, filtered and concentrated. Crystallization with (*c*-Hex: EtOAc = 30:1) gave Ph_3P=O (101.5 mg, 91%).

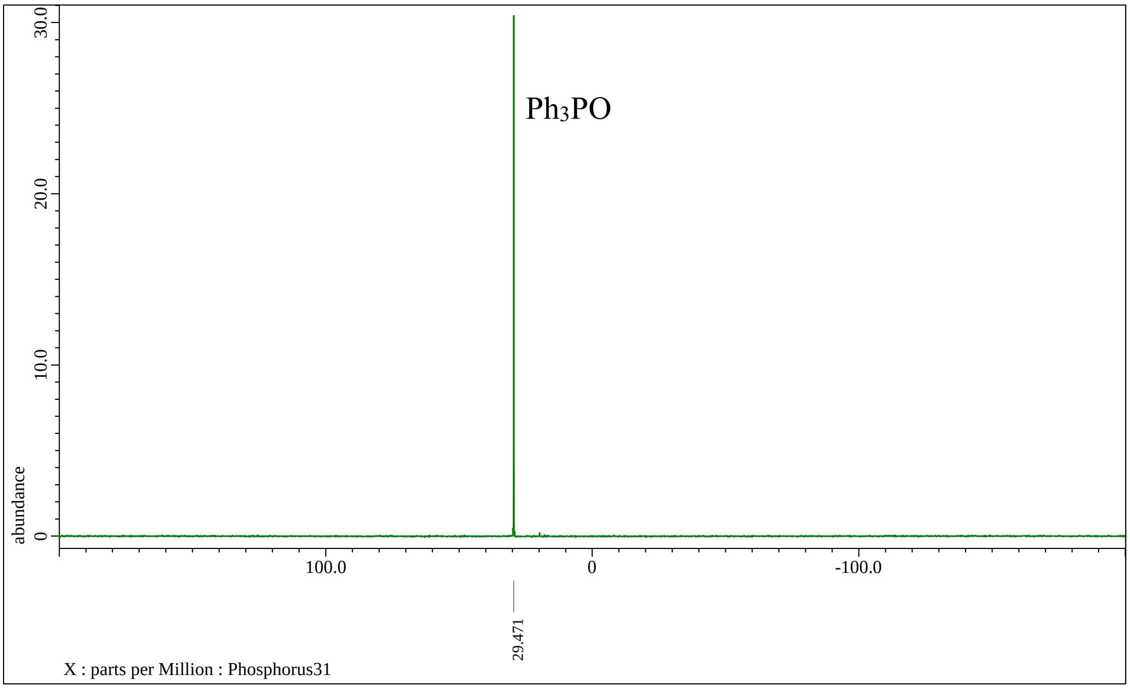

^{31}P NMR analysis of reaction mixture after electrolysis under the condition of Entry 3.

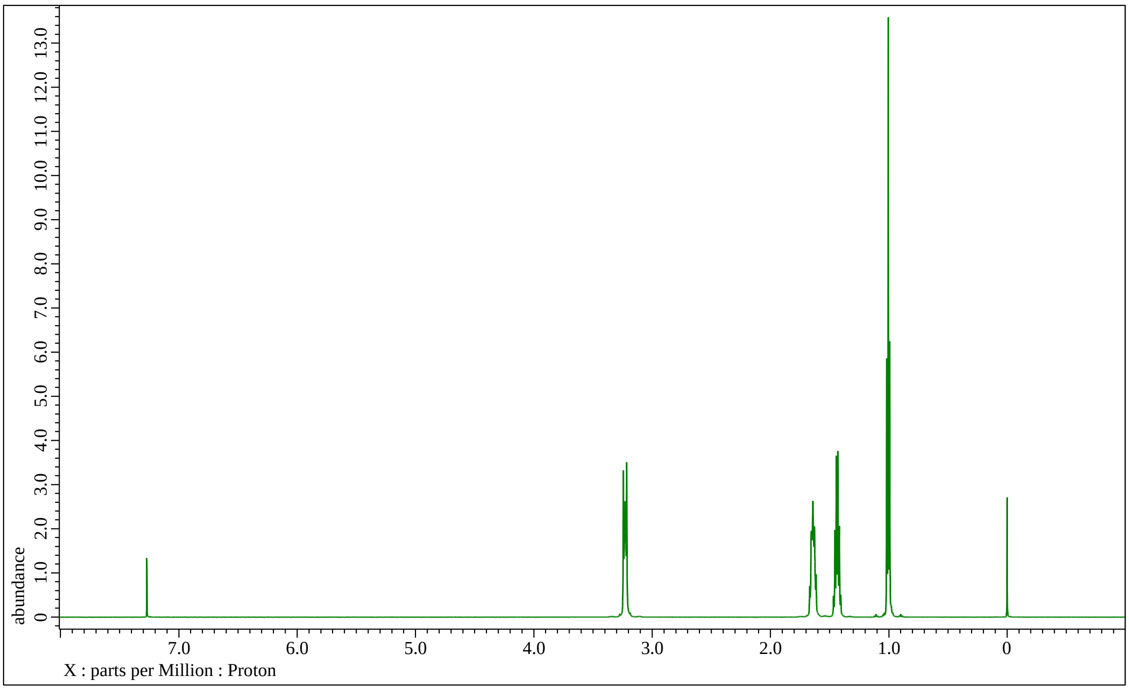

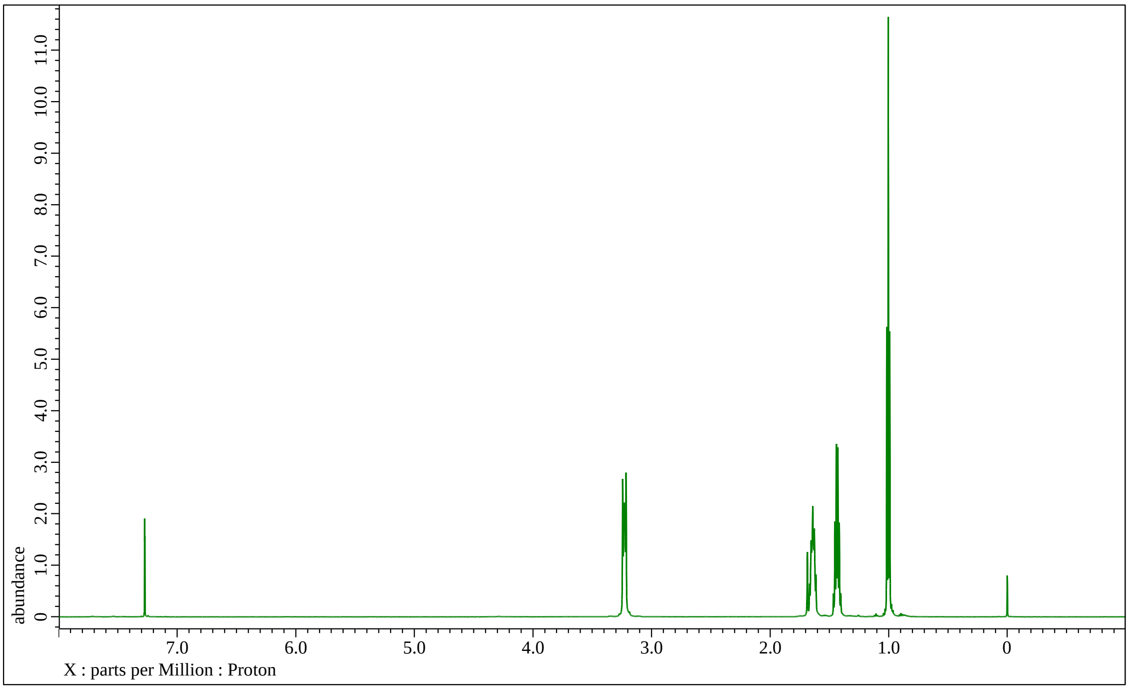

Comparison between Bu_4NClO_4 from commercial source (above) and recovered (below).

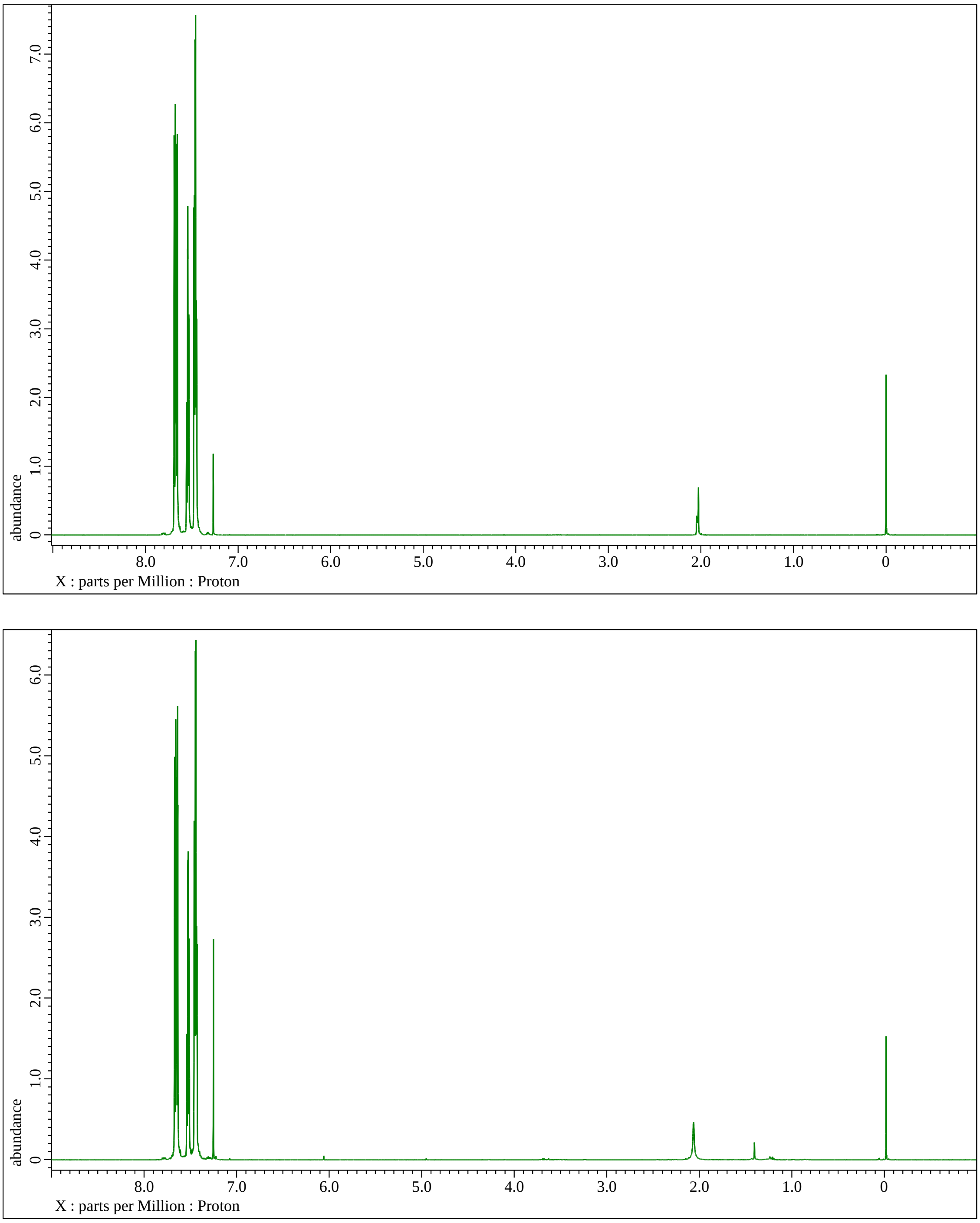

Comparison between Ph_3PO from commercial source (above) and recovered (below).

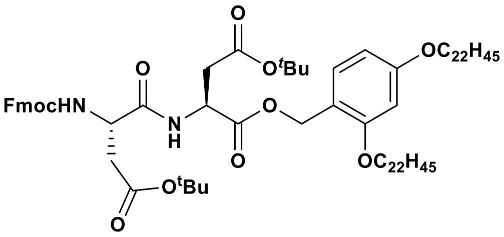

Fmoc -Asp(O^{t}Bu)-Asp(O^{t}Bu)-Tag (**3aa**). Following the general procedure, 2,6-lutidine (69.9 μl, 0.60 mmol), Bu_4NClO_4 (136.8 mg, 0.40 mmol), Ph_3P (104.9 mg, 0.40 mmol), Fmoc-Asp(O^{t}Bu)-OH (123.4 mg, 0.30 mmol), and H_2N-Asp(O^{t}Bu)-Tag (185.7 mg, 0.20 mmol) were added to Ar-purged MeCN (8.0 mL)-*c*-hex (4.0 mL). The solution was electrolyzed at constant current (0.67 mA/cm^2) under Ar at room temparature. After 4.8 F/mol of electricity based on H_2N-Asp(O^{t}Bu)-Tag was passed, the reaction mixture was extracted with *c*-hex (20 mL x 2), and then the solvent was replaced with MeCN. The resulting precipitants were collected by filtration and washed with MeCN to give the product in 95% yield (251.1 mg) as white solid.

^{1}H NMR ($CDCl_3$, 600 MHz) δ 7.76 (2H, d, *J* = 7.6 Hz), 7.63-7.56 (2H, m), 7.45-7.35 (3H, m), 7.31 (2H, t, *J* = 7.6 Hz), 7.14 (1H, d, *J* = 8.3 Hz), 6.43-6.34 (2H, m), 5.95 (1H, d, *J* = 8.3 Hz), 5.19-5.11 (2H, m), 4.82-4.76 (1H, m), 4.62-4.54 (1H, m), 4.42-4.32 (2H, m), 4.22 (1H, t, *J* = 7.6 Hz), 3.95- 3.84 (4H, m), 2.90 (2H, m), 2.72 (1H, dd, *J* = 16.5, 4.1 Hz), 2.63 (1H, dd, *J* = 17.2, 6.2 Hz), 1.80-1.70 (4H, m), 1.48-1.20 (94H, m), 0.88 (6H, t, *J* = 6.9 Hz); ^{13}C NMR ($CDCl_3$, 150 MHz) δ 171.1, 170.4, 170.3, 169.6, 160.8, 158.3, 155.9, 143.9, 143.7, 141.3, 130.9, 127.7, 127.2, 127.1 125.1, 120.0, 116.0, 104.5, 99.6, 81.8, 81.7, 68.1, 68.1, 67.3, 62.9, 51.1, 49.1, 47.1, 37.6, 37.2, 31.9, 29.7, 29.7, 29.6, 29.6, 29.4, 29.4, 29.3, 29.1, 28.0, 27.9, 26.1, 26.0, 22.7, 14.1; HRMS (ESI-MS) $[M+Na]^+$ calcd for $C_{82}H_{132}N_2O_{11}$ 1343.9723, found 1392.9703.

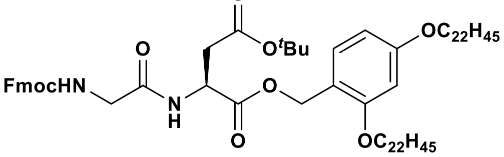

Fmoc-Gly-Asp(O^{t}Bu)-Tag (**3ba**). Following the general procedure, 2,6-lutidine (69.9 μl, 0.60 mmol), Bu_4NClO_4 (136.8 mg, 0.40 mmol), Ph_3P (131.1 mg, 0.50 mmol), Fmoc-Gly-OH (89.2 mg,

0.30 mmol), and H_2N-Asp(O^{t}Bu)-Tag (185.7 mg, 0.20 mmol) were added to Ar-purged MeCN (8.0 mL)-*c*-hex (4.0 mL). The solution was electrolyzed at constant current (0.67 mA/cm^2) under Ar at room temparature. After 4.8 F/mol of electricity based on H_2N-Asp(O^{t}Bu)-Tag was passed, the reaction mixture was extracted with *c*-hex (20 mL x 2), and then the solvent was replaced with MeCN. The resulting precipitants were collected by filtration and washed with MeCN to give the product in 98% yield (237.0 mg) as white solid.

^{1}H NMR ($CDCl_3$, 600 MHz) δ 7.76 (2H, d, *J* = 7.6 Hz), 7.66-7.53 (2H, m), 7.40 (2H, t, *J* = 7.6 Hz), 7.31 (2H, t, *J* = 7.6 Hz), 7.16 (1H, d, *J* = 8.3 Hz), 6.86 (1H, d, *J* = 6.9 Hz), 6.45-6.38 (2H, m), 5.55-5.35 (1H, m), 5.21 (1H, d, *J* = 11.7 Hz), 5.14 (1H, d, *J* = 11.7 Hz), 4.86-4.79 (1H, m), 4.39 (2H, d, *J* = 6.9 Hz), 4.22 (1H, t, *J* = 7.6 Hz), 4.01-3.87 (6H, m), 2.93 (1H, dd, *J* = 17.2 Hz, 4.8 Hz), 2.75 (1H, dd, *J* = 17.2 Hz, 4.1 Hz), 1.80-1.72 (4H, m), 1.48-1.21 (85H, m), 0.88 (6H, t, *J* = 6.9 Hz); ^{13}C NMR($CDCl_3$, 150 MHz) δ 170.5, 169.9, 168.4, 160.9, 158.4, 156.4, 143.8, 143.8, 141.3, 131.1, 127.7, 127.1, 125.1, 125.1, 120.0, 115.8, 104.6, 99.7, 81.8, 68.2, 68.1, 67.3, 63.1, 48.8, 47.1, 44.3, 37.3, 31.9, 29.7, 29.7, 29.6, 29.4, 29.4, 29.3, 29.1, 27.9, 26.1, 26.0, 22.7, 14.1; HRMS (ESI-MS) $[M+Na]^+$ calcd for $C_{76}H_{122}N_2O_9$ 1229.9043, found 1229.9046.

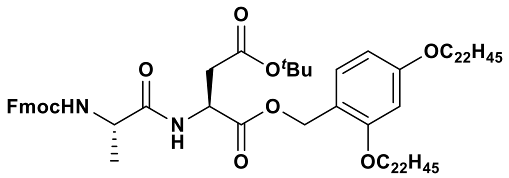

Fmoc-Ala-Asp(O^{t}Bu)-Tag (**3ca**). Following the general procedure, 2,6-lutidine (69.9 μl, 0.60 mmol), Bu_4NClO_4 (136.8 mg, 0.40 mmol), Ph_3P (104.9 mg, 0.40 mmol), Fmoc-Ala-OH (93.4 mg, 0.30 mmol), and H_2N-Asp(O^{t}Bu)-Tag (185.7 mg, 0.20 mmol) were added to Ar-purged MeCN (8.0 mL)-*c*-hex (4.0 mL). The solution was electrolyzed at constant current (0.67 mA/cm^2) under Ar at 50 °C. After 4.8 F/mol of electricity based on H_2N-Asp(O^{t}Bu)-Tag was passed, the reaction mixture was extracted with *c*-hex (20 mL x 2), and then the solvent was replaced with MeCN. The resulting precipitants were collected by filtration and washed with MeCN to give the product

in 92% yield (225.9 mg) as white solid.

^{1}H NMR (CDCl3, 600 MHz) δ 7.76 (2H, d, *J* = 7.6 Hz), 7.63-7.53 (2H, m), 7.40 (2H, t, *J* = 7.6 Hz), 7.31 (2H, t, *J* = 7.6 Hz), 7.16 (1H, d, *J* = 8.3 Hz), 6.78 (1H, d, *J* = 8.3 Hz), 6.44-6.36 (2H, m), 5.44 (1H, d, *J* = 6.2 Hz), 5.20 (1H, d, *J* = 11.7 Hz), 5.14 (1H, d, *J* = 11.7 Hz), 4.86-4.72 (1H, m), 4.36 (2H, d, *J* = 6.9 Hz), 4.26 (1H, t, 7.3 Hz), 4.21 (2H, t, *J* = 7.2 Hz), 3.92 (4H, t, *J* = 4.8Hz), 2.95 (1H, dd , *J* = 16.5 Hz, 4.1 Hz), 2.72 (1H, dd, *J* = 16.5 Hz, 4.1 Hz), 1.80-1.72 (4H, m), 1.47-1.21 (88H, m), 0.88 (6H, t, *J* = 7.3 Hz); ^{13}C NMR($CDCl_3$, 150 MHz) δ 172.0, 170.6, 170.1, 160.9, 158.4, 155.8, 144.0, 143.9, 141.4, 131.2, 127.8, 127.2, 125.2, 120.1, 116.0, 104.6, 100.0, 81.9, 68.2, 68.2, 67.1, 63.1, 50.5, 48.9, 47.2, 37.3, 32.0, 29.8, 29.8, 29.7, 29.5, 29.5, 29.3, 29.2, 28.1, 22.8, 19.2, 14.2; HRMS (ESI-MS) $[M+Na]^+$ calcd for $C_{77}H_{124}N_2O_9$ 1243.9199, found 1243.9199.

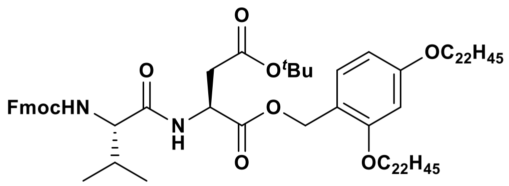

Fmoc-Val-Asp(O^{t}Bu)-Tag (**3da**). Following the general procedure, 2,6-lutidine (69.9 μl, 0.60 mmol), Bu_4NClO_4 (136.8 mg, 0.40 mmol), Ph_3P (104.9 mg, 0.40 mmol), Fmoc-Val-OH (101.8 mg, 0.30 mmol), and H_2N-Asp(O^{t}Bu)-Tag (185.7 mg, 0.20 mmol) were added to Ar-purged MeCN (8.0 mL)-*c*-hex (4.0 mL). The solution was electrolyzed at constant current (0.67 mA/cm^2) under Ar at 40 °C. After 4.8 F/mol of electricity based on H_2N-Asp(O^{t}Bu)-Tag was passed, the reaction mixture was extracted with *c*-hex (20 mL x 2), and then the solvent was replaced with MeCN. The resulting precipitants were collected by filtration and washed with MeCN to give the product in 98% yield (245.7 mg) as white solid.

^{1}H NMR ($CDCl_3$, 600 MHz) δ 7.76 (2H, d, *J* = 7.6 Hz), 7.64-7.56 (2H, m), 7.43-7.37 (2H,m), 7.34-7.28 (2H, m), 7.15 (1H, d, *J* = 8.3 Hz), 6.75 (1H, d, *J* = 8.9 Hz), 6.45-6.35 (2H, m), 5.45 (1H, d, 8.9 Hz), 5.18 (1H, d, 12.4 Hz), 5.14 (1H, d, 12.4 Hz), 4.89-4.80 (1H, m), 4.40 (1H, dd, *J* = 11.0, 7.6 Hz), 4.33 (1H, dd, *J* = 11.0 Hz, 7.6 Hz), 4.22 (1H, t, *J* = 7.6 Hz), 4.06 (1H, dd, *J* = 8.3, 5.5 Hz), 3.92 (4H, t, *J* = 6.5 Hz), 2.97 (1H, dd, *J* = 17.2 Hz, 4.8 Hz), 2.70 (1H, dd, *J* = 17.2 Hz, 4.1

Hz), 2.15-2.05 (1H. m), 1.81-1.70 (4H, m), 1.45-1.21 (85H, m), 1.01-0.85 (12H, m); ^{13}C NMR(CDCl$_3$, 150 MHz) δ 170.7, 170.5, 170.2, 160.9, 158.4, 156.2, 143.9, 143.8, 141.3, 131.2, 127.7, 127.1, 125.2, 125.1, 120.0, 115.8, 104.5, 99.6, 81.9, 68.1, 68.1, 67.1, 63.1, 60.0, 48.6, 47.2, 37.2, 31.9, 31.7, 29.7, 29.7, 29.6, 29.6, 29.4, 29.4, 29.3, 29.1, 28.0, 26.1, 22.7, 19.0, 17.6, 14.1; HRMS (ESI-MS) [M+Na]$^+$ calcd for $C_{79}H_{128}N_2O_9$ 1271.9512, found 1271.9531.

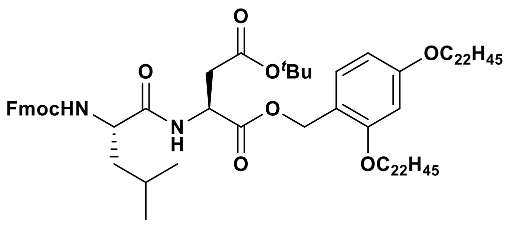

Fmoc-Leu-Asp(O^{t}Bu)-Tag (**3ea**). Following the general procedure, 2,6-lutidine (69.9 μl, 0.60 mmol), Bu_4NClO_4 (136.8 mg, 0.40 mmol), Ph_3P (131.1 mg, 0.50 mmol), Fmoc-Val-OH (106.0 mg, 0.30 mmol), and H_2N-Asp(O^{t}Bu)-Tag (185.7 mg, 0.20 mmol) were added to Ar-purged MeCN (8.0 mL)-*c*-hex (4.0 mL). The solution was electrolyzed at constant current (0.67 mA/cm^2) under Ar at 50 °C. After 4.8 F/mol of electricity based on H_2N-Asp(O^{t}Bu)-Tag was passed, the reaction mixture was extracted with *c*-hex (20 mL x 2), and then the solvent was replaced with MeCN. The resulting precipitants were collected by filtration and washed with MeCN to give the product in 96% yield (242.7 mg) as white solid.

^{1}H NMR (CDCl$_3$, 600 MHz) δ 7.76 (2H, d, *J* = 7.6 Hz), 7.59 (2H, t, *J* = 6.9 Hz), 7.40 (2H, td, *J* = 7.6 Hz, 2.8 Hz), 7.31 (2H, t, *J* = 7.6 Hz), 7.15 (1H, d, *J* = 8.3 Hz), 6.77 (1H, d, J = 8.3 Hz), 6.44-6.33 (2H, m), 5.24 (1H, d, *J* = 8.3 Hz), 5.18 (1H, d, *J* = 11.7 Hz), 5.15 (1H, d, *J* = 11.7 Hz), 4.86-4.75 (1H, m), 4.44-4.30 (2H, m), 4.25-4.17 (2H, t, *J* = 6.9 Hz), 3.91 (4H, m), 2.96 (1H, dd, *J* = 16.5 Hz, 4.1 Hz), 2.71 (1H, dd, *J* = 16.8, 4.1 Hz), 1.80-1.71 (4H, m), 1.71-1.58 (2H, m), 1.54-1.47 (1H, m), 1.45-1.19 (85H, m), 0.91 (6H, t, *J* = 6.2 Hz), 0.88 (6H, t, *J* = 6.9 Hz); ^{13}C NMR(CDCl$_3$, 150 MHz) δ 171.8, 170.5, 170.0, 160.8, 158.3, 156.0, 143.9, 143.8, 141.3, 131.1, 127.7, 127.1, 125.1, 120.0, 119.9, 115.9, 104.5, 99.6, 81.8, 68.1, 68.1, 67.0, 63.0, 53.4, 48.7, 47.2, 42.1, 37.3, 31.9, 29.7, 29.7, 29.6, 29.4, 29.4, 29.3, 29.1, 28.0, 26.1, 26.0, 24.6, 22.9, 22.7, 22.0,

14.1; HRMS (ESI-MS) $[M+Na]^+$ calcd for $C_{80}H_{130}N_2O_9$ 1285.9669, found 1285.9691.

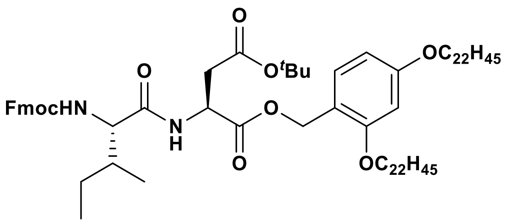

Fmoc-Ile-Asp(O^{t}Bu)-Tag (**3fa**). Following the general procedure, 2,6-lutidine (69.9 μl, 0.60 mmol), Bu_4NClO_4 (136.8 mg, 0.40 mmol), Ph_3P (131.1 mg, 0.50 mmol), Fmoc-Ile-OH (106.0 mg, 0.30 mmol), and H_2N-Asp(O^{t}Bu)-Tag (185.7 mg, 0.20 mmol) were added to Ar-purged MeCN (8.0 mL)-*c*-hex (4.0 mL). The solution was electrolyzed at constant current (0.67 mA/cm^2) under Ar at 50 °C. After 4.8 F/mol of electricity based on H_2N-Asp(O^{t}Bu)-Tag was passed, the reaction mixture was extracted with *c*-hex (20 mL x 2), and then the solvent was replaced with MeCN. The resulting precipitants were collected by filtration and washed with MeCN to give the product in 92% yield (233.6 mg) as white solid.

^{1}H NMR ($CDCl_3$, 600 MHz) δ 7.76 (2H, d, *J* = 7.6 Hz), 7.60 (2H, d, *J* = 7.6 Hz), 7.40 (2H, m), 7.31 (2H, t, *J* = 7.6 Hz), 7.15 (1H, d, *J* = 8.3 Hz), 6.73 (1H, d, J = 8.3 Hz), 6.45-6.33 (2H, m), 5.50 (1H, d, *J* = 8.9 Hz), 5.18 (1H, d, *J* = 11.7 Hz), 5.14 (1H, d, *J* = 11.7 Hz), 4.86-4.75 (1H, m), 4.39 (1H, dd, *J* = 10.7 Hz, 7.6 Hz), 4.35 (1H, dd, *J* = 10.7 Hz, 7.6 Hz), 4.21 (2H, t, 7.6 Hz), 4.14-4.03 (1H, m), 3.91 (4H, t, *J* = 6.9 Hz), 2.95 (2H, dd, *J* = 16.5 Hz, 4.1 Hz), 2.71 (1H, dd, *J* = 17.1 Hz, 4.1 Hz), 1.89-1.81 (1H, m), 1.81-1.70 (4H, m), 1.71-1.58 (2H, m), 1.51-1.22 (85H, m), 1.22 (2H, m), 0.97-0.83 (12H, m); ^{13}C NMR($CDCl_3$, 150 MHz) δ 170.7, 170.6, 170.2, 160.9, 158.5, 156.1, 144.0, 143.9, 141.4, 131.2, 127.8, 127.2, 125.2, 120.0, 115.9, 104.6, 99.7, 81.9, 68.2, 68.2, 63.1, 59.5, 48.7, 47.3, 38.2, 37.3, 32.0, 29.8, 29.8, 29.7, 29.5, 29.5, 29.3, 29.2, 28.1, 26.1, 25.0, 22.8, 15.2, 14.2, 11.6; HRMS (ESI-MS) $[M+Na]^+$ calcd for $C_{80}H_{130}N_2O_9$ 1285.9669, found 1285.9680.

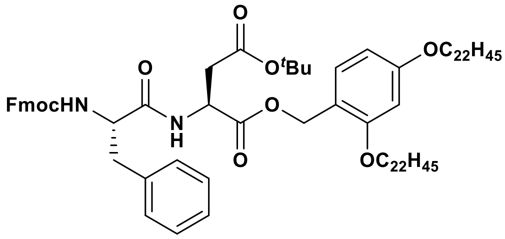

Fmoc-Phe-Asp(O^{t}Bu)-Tag (**3ga**). Following the general procedure, 2,6-lutidine (69.9 μl, 0.60 mmol), Bu_4NClO_4 (136.8 mg, 0.40 mmol), Ph_3P (104.9 mg, 0.40 mmol), Fmoc-Phe-OH (116.2 mg, 0.30 mmol), and H_2N-Asp(O^{t}Bu)-Tag (185.7 mg, 0.20 mmol) were added to Ar-purged MeCN (8.0 mL)-*c*-hex (4.0 mL). The solution was electrolyzed at constant current (0.67 mA/cm^2) under Ar at 40 °C. After 4.8 F/mol of electricity based on H_2N-Asp(O^{t}Bu)-Tag was passed, the reaction mixture was extracted with *c*-hex (20 mL x 2), and then the solvent was replaced with MeCN. The resulting precipitants were collected by filtration and washed with MeCN to give the product in 97% yield (251.0 mg) as white solid.

^{1}H NMR ($CDCl_3$, 600 MHz) δ 7.76 (2H, d, *J* = 7.6 Hz), 7.53 (1H, d, *J* = 7.6 Hz), 7.51 (1H, d, *J* = 7.6 Hz), 7.39 (2H, t, *J* = 7.6 Hz), 7.30 (2H, t, *J* = 7.6 Hz), 7.26-7.19 (4H, m), 7.16 (2H, d, *J* = 6.9 Hz), 6.84 (1H, d, *J* = 6.9 Hz), 6.42-6.35 (2H, m), 5.33 (1H, d, *J* = 7.6 Hz), 5.19 (1H, d, *J* = 11.7 Hz), 5.15 (1H, d, *J* = 11.7 Hz), 4.82-4.72 (1H, m), 4.52-4.43 (1H, m), 4.38 (1H, dd, *J* = 10.9, 7.6 Hz), 4.30-4.22 (1H, m), 4.17 (1H, t, *J* = 6.9 Hz), 3.96-3.81 (4H, m), 3.24-2.98 (2H, m), 2.90 (1H, dd, *J* = 16.5 Hz, 3.4 Hz), 2.70 (1H, dd, *J* = 16.5 Hz, 4.1 Hz), 1.80-1.68 (4H, m), 1.45-1.20 (85H, m), 0.88 (6H, t, *J* = 6.9 Hz); ^{13}C NMR($CDCl_3$, 150 MHz) δ 170.4, 170.4, 169.9, 160.9, 158.3, 155.7, 143.8, 143.8, 141.3, 131.1, 129.4, 128.6, 127.7, 127.1, 125.2, 125.1, 120.0, 115.9, 104.6, 99.6, 81.7, 68.2, 68.1, 67.1, 63.0, 55.7, 48.9, 47.1, 38.5, 37.3, 31.9, 29.7, 29.7, 29.6, 29.4, 29.4, 29.3, 29.2, 28.0, 26.0, 22.7, 14.1; HRMS (ESI-MS) [M+Na]$^+$ calcd for $C_{83}H_{128}N_2O_9$ 1319.9512, found 1319.9533.

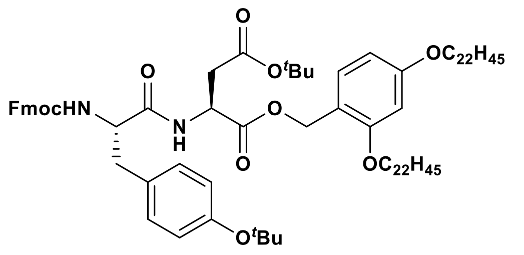

Fmoc-Tyr(O^{t}Bu)-Asp(O^{t}Bu)-Tag (**3ha**). Following the general procedure, 2,6-lutidine (69.9 µl, 0.60 mmol), Bu_4NClO_4 (136.8 mg, 0.40 mmol), Ph_3P (104.9 mg, 0.40 mmol), Fmoc-Tyr(O^{t}Bu)-OH (137.9 mg, 0.30 mmol), and H_2N-Asp(O^{t}Bu)-Tag (185.7 mg, 0.20 mmol) were added to Ar-purged MeCN (8.0 mL)-*c*-hex (4.0 mL). The solution was electrolyzed at constant current (0.67 mA/cm^2) under Ar at room temperature. After 4.8 F/mol of electricity based on H_2N-Asp(O^{t}Bu)-Tag was passed, the reaction mixture was extracted with *c*-hex (20 mL x 2), and then the solvent was replaced with MeCN. The resulting precipitants were collected by filtration and washed with MeCN to give the product in 95% yield (261.0 mg) as white solid.

^{1}H NMR ($CDCl_3$, 600 MHz) δ 7.75 (2H, d, *J* = 7.6 Hz), 7.58-7.49 (2H, m), 7.39 (2H, t, *J* = 7.6 Hz), 7.33-7.27 (2H, m), 7.16 (1H, d, *J* = 7.6 Hz), 7.06 (2H, d, *J* = 7.6 Hz), 6.87 (2H, d, *J* = 7.6 Hz), 6.80 (1H, d, *J* = 7.6 Hz), 6.44-6.34 (2H, m), 5.33 (1H, d, *J* = 7.6 Hz), 5.18 (1H, d, *J* = 11.7 Hz), 5.15 (1H, d, *J* = 11.7 Hz), 4.82-4.71 (1H, m), 4.48-4.40 (1H, m), 4.39-4.32 (1H, m), 4.31-4.24 (1H, m), 4.17 (1H, t, *J* = 6.9 Hz), 3.94-3.83 (4H, m), 3.12-3.02 (1H,m), 3.02-2.94 (1H, m), 2.90 (1H, dd, *J* = 16.5, 4.1 Hz), 2.70 (1H, dd, *J* = 16.5, 4.1 Hz), 1.80-1.68 (4H, m), 1.45-1.21 (94H, m), 0.88 (6H, t, *J* = 6.9 Hz); ^{13}C NMR($CDCl_3$, 150 MHz) δ 170.5, 170.4, 169.8, 160.8, 158.3, 155.7, 154.4, 143.8, 143.8, 141.3, 131.1, 129.8, 127.7, 127.1, 125.1, 125.1, 124.3, 120.0, 115.9, 104.6, 99.6, 81.7, 78.3, 68.2, 68.1, 67.1, 63.0, 55.9, 48.9, 47.1, 38.0, 37.3, 31.9, 29.7, 29.7, 29.6, 29.4, 29.4, 29.3, 29.2, 28.8, 27.9, 26.1, 26.0, 22.7, 14.1; HRMS (ESI-MS) $[M+Na]^+$ calcd for $C_{87}H_{136}N_2O_{10}$ 1392.0087, found 1392.0077.

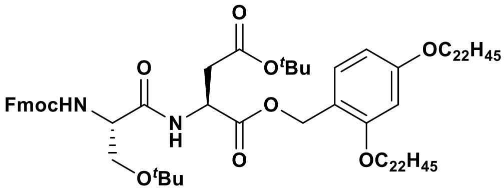

Fmoc-Ser(O^{t}Bu)-Asp(O^{t}Bu)-Tag (**3ia**). Following the general procedure, 2,6-lutidine (69.9 μl, 0.60 mmol), Bu_4NClO_4 (136.8 mg, 0.40 mmol), Ph_3P (131.1 mg, 0.50 mmol), Fmoc-Ser(O^{t}Bu)-OH (153.4 mg, 0.40 mmol), and H_2N-Asp(O^{t}Bu)-Tag (185.7 mg, 0.20 mmol) were added to Ar-purged MeCN (8.0 mL)-*c*-hex (4.0 mL). The solution was electrolyzed at constant current (0.67 mA/cm^2) under Ar at 40 ºC. After 4.8 F/mol of electricity based on H_2N-Asp(O^{t}Bu)-Tag was passed, the reaction mixture was extracted with *c*-hex (20 mL x 2), and then the solvent was replaced with MeCN. The resulting precipitants were collected by filtration and washed with MeCN to give the product in 98% yield (253.1 mg) as white solid.

^{1}H NMR ($CDCl_3$, 600 MHz) δ 7.81 (1H, d, *J* = 6.2 Hz), 7.76 (2H, d, *J* = 7.6 Hz), 7.64-7.53 (2H, m), 7.40 (2H, t, *J* = 7.6 Hz), 7.31 (2H, t, *J* = 7.6 Hz), 7.16 (1H, d, *J* = 7.6 Hz), 6.44-6.35 (2H, m), 5.79 (1H, d, *J* = 5.5 Hz), 5.20 (1H, d, *J* = 11.7 Hz), 5.14 (1H, d, *J* = 11.7 Hz), 4.85-4.79 (1H, m), 4.41-4.33 (2H, m), 4.29-4.18 (2H, m), 3.95-3.86 (4H, m), 3.84-3.76 (1H, m), 3.41 (1H, t, *J* = 8.6 Hz), 2.91 (1H, dd, *J* = 16.5 Hz, 4.8 Hz), 2.74 (1H, dd, *J* = 17.2 Hz, 4.8 Hz), 1.80-1.71 (4H, m), 1.46-1.18 (94H, m), 0.88 (6H, t, *J* = 6.9 Hz); ^{13}C NMR($CDCl_3$, 150 MHz) δ 170.4, 170.2, 169.6, 160.8, 158.3, 156.0, 144.0, 143.8, 141.3, 130.9, 127.7, 127.1, 125.2, 125.2, 120.0, 116.0, 104.6, 99.6, 81.5, 74.4, 68.1, 68.1, 67.2, 62.8, 61.7, 54.2, 49.1, 47.1, 37.5, 31.9, 29.7, 29.7, 29.6, 29.4, 29.4, 29.3, 29.2, 28.0, 27.3, 26.1, 22.7, 14.1; HRMS (ESI-MS) [M+Na]$^+$ calcd for $C_{81}H_{132}N_2O_{10}$ 1315.9774, found 1315.9796.

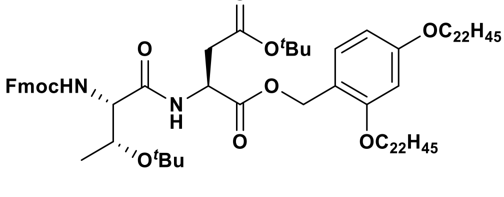

Fmoc-Thr(O^{t}Bu)-Asp(O^{t}Bu)-Tag (**3ja**). Following the general procedure, 2,6-lutidine (69.9 μl, 0.60 mmol), Bu_4NClO_4 (136.8 mg, 0.40 mmol), Ph_3P (131.1 mg, 0.50 mmol), Fmoc-Thr(O^{t}Bu)-OH (159.0 mg, 0.40 mmol), and H_2N-Asp(O^{t}Bu)-Tag (185.7 mg, 0.20 mmol) were added to Ar-purged MeCN (8.0 mL)-*c*-hex (4.0 mL). The solution was electrolyzed at constant current (0.67 mA/cm^2) under Ar at 40 ºC. After 4.8 F/mol of electricity based on H_2N-Asp(O^{t}Bu)-Tag was passed, the reaction mixture was extracted with *c*-hex (20 mL x 2), and then the solvent was replaced with MeCN. The resulting precipitants were collected by filtration and washed with MeCN to give the product in 93% yield (243.3 mg) as white solid.

^{1}H NMR ($CDCl_3$, 600 MHz) δ 8.12 (1H, d, *J* = 7.6 Hz), 7.76 (1H, d, *J* = 7.6 Hz), 7.61 (1H, d, *J* = 7.6 Hz), 7.43-7.36 (2H, m), 7.31 (2H, t, *J* = 7.6 Hz), 7.19 (1H, d, *J* = 8.9 Hz), 6.44-6.37 (2H, m), 6.01 (1H, d, *J* = 5.5 Hz), 5.21 (1H, d, *J* = 12.4 Hz), 5.17 (1H, d, *J* = 12.4 Hz), 4.88-4.80 (1H, m), 4.42-4.32 (2H, m), 4.25-4.11 (3H, m), 3.95-3.87 (4H, m), 2.92 (1H, dd, *J* = 17.2 Hz, 4.8 Hz), 2.74 (1H, dd, *J* = 17.2 Hz, 4.1 Hz), 1.80-1.72 (4H, m), 1.47-1.20 (94H, m), 1.07 (3H, d, *J* = 6.2 Hz), 0.88 (6H, t, *J* = 6.9 Hz); ^{13}C NMR($CDCl_3$, 150 MHz) δ 170.5, 169.6, 169.3, 160.7, 158.3, 156.0, 144.0, 143.7, 143.7, 141.3, 141.3, 131.0, 127.7, 127.1, 125.2, 120.0, 120.0, 116.1, 104.6, 99.5, 81.4, 75.5, 68.1, 67.0, 66.6, 62.9, 58.5, 49.0, 47.2, 37.6, 31.9, 29.7, 29.6, 29.6, 29.4, 29.4, 29.4, 29.3, 29.2, 28.1, 28.0, 26.1, 22.7, 16.5, 14.1; HRMS (ESI-MS) $[M+Na]^+$ calcd for $C_{82}H_{134}N_2O_{10}$ 1329.9931, found 1329.9909.

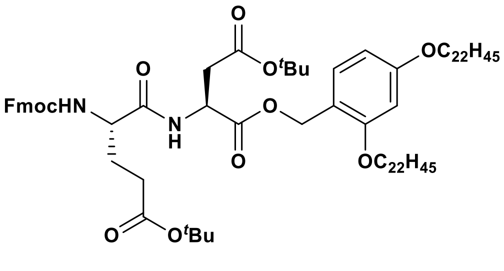

Fmoc-Glu(O^{t}Bu)-Asp(O^{t}Bu)-Tag (**3ka**). Following the general procedure, 2,6-lutidine (69.9 μl, 0.60 mmol), Bu_4NClO_4 (136.8 mg, 0.40 mmol), Ph_3P (104.9 mg, 0.40 mmol), Fmoc-Glu(O^{t}Bu)-OH (133.1 mg, 0.30 mmol), and H_2N-Asp(O^{t}Bu)-Tag (185.7 mg, 0.20 mmol) were added to Ar-purged MeCN (8.0 mL)-*c*-hex (4.0 mL). The solution was electrolyzed at constant current (0.67

mA/cm^2) under Ar at room temperature. After 4.8 F/mol of electricity based on H_2N-Asp(O^{t}Bu)-Tag was passed, the reaction mixture was extracted with *c*-hex (20 mL x 2), and then the solvent was replaced with MeCN. The resulting precipitants were collected by filtration and washed with MeCN to give the product in 92% yield (245.3 mg) as white solid.

^{1}H NMR ($CDCl_3$, 600 MHz) δ 7.76 (2H, d, J = 7.6 Hz), 7.60 (2H, m), 7.39 (2H, t, J = 7.6 Hz), 7.31 (2H, t, J = 7.6 Hz), 7.19-7.09 (2H, m), 6.44-6.35 (2H, m), 5.72 (1H, d, J = 7.6 Hz), 5.18 (1H, d, J = 12.4 Hz), 5.15 (1H, d, J = 12.4 Hz), 4.86-4.80 (1H, m), 4.39-4.31 (2H, m), 4.28 (1H, dd, J = 13.1 Hz, 7.6 Hz), 4.21 (1H, t, J = 7.6 Hz), 3.95-3.86 (4H, m), 2.94 (1H, dd, J = 17.2 Hz, 4.8 Hz), 2.72 (1H, dd, J = 17.2, 4.8 Hz), 2.44-2.33 (2H, m), 2.13-2.05 (1H, m), 1.96-1.86 (1H, m), 1.80-1.71 (4H, m), 1.49-1.21 (94H, m), 0.88 (6H, t, J = 6.9 Hz); ^{13}C NMR($CDCl_3$, 150 MHz) δ 172.7, 171.0, 170.5, 169.7, 160.8, 158.3, 156.0, 144.0, 143.8, 141.3, 131.0, 127.7, 127.1, 125.2, 125.2, 120.0, 115.9, 104.6, 99.6, 81.8, 80.9, 68.2, 68.1, 67.1, 63.0, 54.1, 48.8, 47.1, 37.3, 31.9, 31.5, 29.7, 29.7, 29.6, 29.4, 29.4, 29.3, 29.1, 28.6, 28.1, 28.0, 26.1, 26.0, 22.7, 14.1; HRMS (ESI-MS) $[M+Na]^+$ calcd for $C_{83}H_{134}N_2O_{11}$ 1357.9880, found 1357.9876.

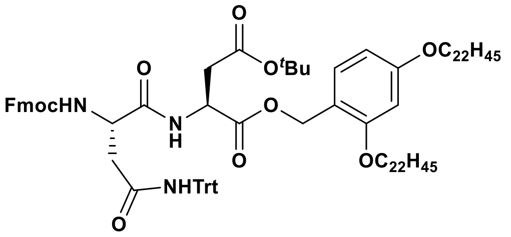

Fmoc-Asn(Trt)-Asp(O^{t}Bu)-Tag (**3la**). Following the general procedure, 2,6-lutidine (69.9 μl, 0.60 mmol), Bu_4NClO_4 (136.8 mg, 0.40 mmol), Ph_3P (131.1 mg, 0.50 mmol), Fmoc-Asn(Trt)-OH (179.0 mg, 0.30 mmol), and H_2N-Asp(O^{t}Bu)-Tag (185.7 mg, 0.20 mmol) were added to Ar-purged MeCN (8.0 mL)-*c*-hex (4.0 mL). The solution was electrolyzed at constant current (0.67 mA/cm^2) under Ar at room temperature. After 4.8 F/mol of electricity based on H_2N-Asp(O^{t}Bu)-Tag was passed, the reaction mixture was extracted with *c*-hex (20 mL x 2), and then the solvent was replaced with MeCN. The resulting precipitants were collected by filtration and washed with MeCN to give the product in 99% yield (300.5 mg) as white solid.

^{1}H NMR ($CDCl_3$, 600 MHz) δ 7.75 (2H, d, *J* = 6.9 Hz), 7.60-7.50 (3H, m), 7.38 (2H, t, *J* = 7.6 Hz), 7.32-7.26 (7H, m), 7.26-7.11 (11H, m), 6.89 (1H, s), 6.43-6.34 (3H, m), 5.13 (2H, s), 4.85-4.78 (1H, m), 4.60 (1H, s), 4.38-4.27 (2H, m), 4.18 (1H, t, *J* = 7.2 Hz), 3.93-3.84 (4H, m), 3.09 (1H, d, *J* = 14.8 Hz), 2.81 (1H, dd, *J* = 16.5 Hz, 4.8 Hz), 2.64 (1H, dd, *J* = 15.8Hz, 4.8 Hz), 2.55 (1H, dd, *J* = 17.2 Hz, 4.8 Hz), 1.78-1.71 (4H, m), 1.45-1.22 (85H, m), 0.88 (6H, t, *J* = 6.9 Hz); ^{13}C NMR($CDCl_3$, 150 MHz) δ 170.7, 170.5, 170.4, 169.5, 160.7, 158.2, 156.3, 144.3, 143.9, 143.7, 141.2, 130.8, 128.7, 128.0, 127.7, 127.1, 125.3, 119.9, 116.0, 104.5, 99.6, 81.7, 70.9, 68.1, 68.1, 67.4, 62.8, 51.6, 49.0, 47.1, 38.1, 37.3, 31.9, 29.7, 29.7, 29.6, 29.6, 29.4, 29.4, 29.3, 29.1, 27.9, 26.1, 26.0, 22.7, 14.1; HRMS (ESI-MS) $[M+Na]^+$ calcd for $C_{97}H_{139}N_3O_{10}$ 1529.0353, found 1529.0349.

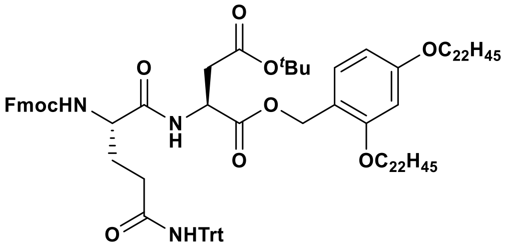

Fmoc-Gln(Trt)-Asp(O*t*Bu)-Tag (**3ma**). Following the general procedure, 2,6-lutidine (69.9 μl, 0.60 mmol), Bu_4NClO_4 (136.8 mg, 0.40 mmol), Ph_3P (131.1 mg, 0.50 mmol), Fmoc-Gln(Trt)-OH (183.2 mg, 0.30 mmol), and H_2N-Asp(O*t*Bu)-Tag (185.7 mg, 0.20 mmol) were added to Ar-purged MeCN (8.0 mL)-*c*-hex (4.0 mL). The solution was electrolyzed at constant current (0.67 mA/cm^2) under Ar at room temperature. After 4.8 F/mol of electricity based on H_2N-Asp(O*t*Bu)-Tag was passed, the reaction mixture was extracted with *c*-hex (20 mL x 2), and then the solvent was replaced with MeCN. The resulting precipitants were collected by filtration and washed with MeCN to give the product in 99% yield (301.2mg) as white solid.

^{1}H NMR ($CDCl_3$, 600 MHz) δ 7.75 (2H, d, *J* = 7.6 Hz), 7.58 (2H, d, *J* = 6.9 Hz), 7.41-7.34 (2H, m), 7.31-7.26 (8H, m), 7.26-7.19 (9H, m), 7.17 (1H, d, *J* = 7.6 Hz), 7.08 (1H, d, *J* = 8.3 Hz), 7.02 (1H, s), 6.40 (1H, d, *J* = 1.4 Hz), 6.35 (1H, d, *J* = 8.3 Hz), 5.83 (1H, d, *J* = 6.9 Hz), 5.10 (1H, d, *J* = 11.7 Hz), 5.07 (1H, d, *J* = 11.7 Hz), 4.85-4.78 (1H, m), 4.38-4.28(2H, m), 4.20 (1H, t, *J* = 7.2

Hz), 4.18-4.111 (1H, m), 3.94-3.86 (4H, m), 2.82 (1H, dd, *J* = 17.2 Hz, 4.8 Hz), 2.68 (1H, dd, *J* = 17.2 Hz, 4.8 Hz), 2.50-2.38 (2H, m), 2.13-1.97 (2H, m), 1.80-1.69 (4H, m), 1.46-1.22 (85H, m), 0.88 (6H, t, *J* = 6.9 Hz); ^{13}C NMR($CDCl_3$, 150 MHz) δ 171.7, 171.1, 170.6, 169.7, 160.8, 158.3, 156.0, 144.6, 143.9, 143.8, 141.3, 141.3, 131.1, 128.8, 127.9, 127.7, 127.1, 127.0, 125.2, 119.9, 115.8, 104.5, 99.6, 81.8, 70.7, 68.2, 68.1, 67.1, 63.0, 54.0, 48.9, 47.2, 37.3, 33.0, 31.9, 29.7, 29.6, 29.4, 29.4, 29.3, 29.1, 27.9, 26.1, 26.0, 22.7, 14.1; HRMS (ESI-MS) $[M+Na]^+$ calcd for $C_{98}H_{141}N_3O_{10}$ 1543.0509, found 1543.0519.

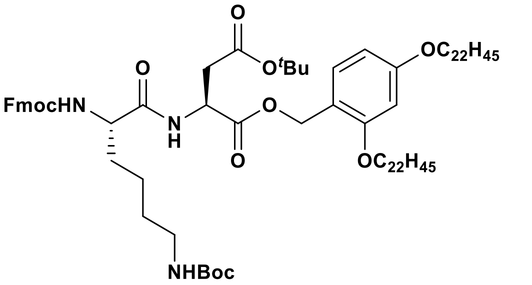

Fmoc-Lys(Boc)-Asp(O^{t}Bu)-Tag (**3na**). Following the general procedure, 2,6-lutidine (69.9 μl, 0.60 mmol), Bu_4NClO_4 (136.8 mg, 0.40 mmol), Ph_3P (131.1 mg, 0.50 mmol), Fmoc-Lys(Boc)-OH (140.6 mg, 0.30 mmol), and H_2N-Asp(O^{t}Bu)-Tag (185.7 mg, 0.20 mmol) were added to Ar-purged MeCN (8.0 mL)-*c*-hex (4.0 mL). The solution was electrolyzed at constant current (0.67 mA/cm^2) under Ar at 60 ºC. After 4.8 F/mol of electricity based on H_2N-Asp(O^{t}Bu)-Tag was passed, the reaction mixture was extracted with *c*-hex (20 mL x 2), and then the solvent was replaced with MeCN. The resulting precipitants were collected by filtration and washed with MeCN to give the product in 91% yield (252.0 mg) as white solid.

^{1}H NMR ($CDCl_3$, 600 MHz) δ 7.76 (2H, d, *J* = 7.6 Hz), 7.63-7.51 (2H, m), 7.40 (2H, t, *J* = 7.6 Hz), 7.31 (2H, t, *J* = 7.6 Hz), 7.16 (1H, d, *J* = 8.3 Hz), 6.81 (1H, d, *J* = 8.3 Hz), 6.45-6.34 (2H, m), 5.51 (1H, d, *J* = 6.9 Hz), 5.16 (2H, s), 4.86-4.79 (1H, m), 4.72 (1H, s), 4.37 (2H, d, *J* = 6.9 Hz), 4.26-4.14 (2H, m), 3.97-3.85 (4H, m), 3.18-3.00 (2H, m), 2.95 (1H, dd, *J* = 17.2 Hz, 4.8 Hz), 2.70 (1H, dd, *J* = 17.2 Hz, 4.1 Hz), 1.89-1.60 (6H, m), 1.51-1.21 (98H, m), 0.88 (6H, t, *J* = 6.9 Hz); ^{13}C NMR($CDCl_3$, 150 MHz) δ 171.3, 171.3, 170.6, 170.0, 160.8, 158.4, 156.1, 156.0, 143.9,

143.8, 141.3, 131.2, 127.7, 127.1, 125.2, 119.9, 115.9, 104.6, 99.6, 81.9, 79.0, 68.2, 68.1, 67.1, 63.1, 54.7, 48.7, 47.2, 39.9, 37.2, 32.6, 31.9, 29.7, 29.7, 29.6, 29.6, 29.5, 29.4, 29.4, 29.3, 29.1, 28.5, 28.0, 26.1, 26.0, 22.7, 22.2, 14.1; HRMS (ESI-MS) $[M+Na]^+$ calcd for $C_{85}H_{139}N_3O_{11}$ 1401.0302, found 1401.0320.

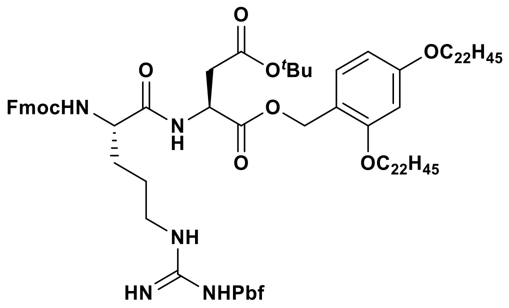

Fmoc-Arg(Pbf)-Asp(O^{t}Bu)-Tag (**3oa**). Following the general procedure, 2,6-lutidine (69.9 µl, 0.60 mmol), Bu_4NClO_4 (136.8 mg, 0.40 mmol), Ph_3P (157.4 mg, 0.60 mmol), Fmoc-Arg(Pbf)-OH (222.1 mg, 0.30 mmol), and H_2N-Asp(O^{t}Bu)-Tag (185.7 mg, 0.20 mmol) were added to Ar-purged MeCN (8.0 mL)-*c*-hex (4.0 mL). The solution was electrolyzed at constant current (0.67 mA/cm^2) under Ar at 50 ºC. After 4.8 F/mol of electricity based on H_2N-Asp(O^{t}Bu)-Tag was passed, the reaction mixture was extracted with *c*-hex (20 mL x 2), and then the solvent was replaced with MeCN. The resulting precipitants were collected by filtration and washed with MeCN to give the product in 93% yield (290.1 mg) as white solid.

^{1}H NMR ($CDCl_3$, 600 MHz) δ 7.75 (2H, d, *J* = 7.6 Hz), 7.57 (2H, d, *J* = 7.6 Hz), 7.41-7.34 (2H, m), 7.32-7.26 (2H, m), 7.12 (1H, d, *J* = 8.3 Hz), 7.01 (1H, s), 6.42-6.34 (2H, m), 6.13-5.94 (2H, m), 5.72 (1H, s), 5.13 (2H, s), 4.91-4.84 (1H, m), 4.42-4.24 (3H, m), 4.18 (1H, t, *J* = 6.9 Hz), 3.91 (4H, t, *J* = 6.9 Hz), 3.27-3.10 (2H, m), 2.99-2.88 (3H, m), 2.69 (1H, dd, *J* = 17.2 Hz, 4.1 Hz), 2.60 (2.5H, s, major rotamer), 2.59 (0.5H, s, minor rotamer), 2.53 (2.5H, s, minor rotamer), 2.52 (0.5H, minor rotamer), 2.09 (2.5H, s, major rotamer), 2.08 (0.5H, s, minor rotamer), 1.78-1.71 (4H, m), 1.70-1.53 (3H, m), 1.46-1.19 (93H, m), 0.88 (6H, t, *J* = 6.9 Hz); ^{13}C NMR($CDCl_3$, 150 MHz) δ 171.4, 171.3, 170.1, 160.9, 158.7, 158.3, 156.2, 143.8, 143.7, 141.3, 141.3, 138.4, 133.1, 132.3, 131.1, 127.7, 127.1, 125.1, 124.5, 120.0, 119.9, 117.4, 115.6, 104.6, 99.6, 86.3, 82.0, 68.2, 68.2,

67.1, 63.3, 53.9, 48.8, 47.1, 43.2, 40.5, 40.5, 40.4, 37.1, 31.9, 30.2, 29.7, 29.7, 29.6, 29.5, 29.4, 29.3, 29.1, 28.6, 27.9, 26.1, 26.0, 22.7, 19.3, 18.0, 14.1, 12.5; HRMS (ESI-MS) $[M+Na]^+$ calcd for $C_{93}H_{147}N_5O_{12}S$ 1581.0659, found 1581.0668.

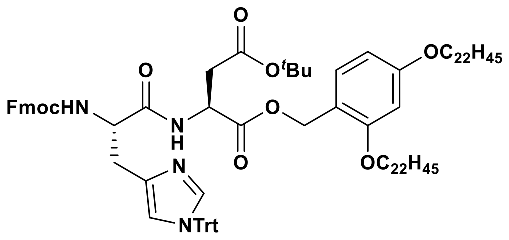

Fmoc-His(Trt)-Asp(O^{t}Bu)-Tag (**3pa**). Following the general procedure, 2,6-lutidine (69.9 μl, 0.60 mmol), Bu_4NClO_4 (136.8 mg, 0.40 mmol), Ph_3P (157.4 mg, 0.60 mmol), Fmoc-His(Trt)-OH (185.9 mg, 0.30 mmol), and H_2N-Asp(O^{t}Bu)-Tag (185.7 mg, 0.20 mmol) were added to Ar-purged MeCN (8.0 mL)-*c*-hex (4.0 mL). The solution was electrolyzed at constant current (0.67 mA/cm^2) under Ar at room temperature. After 4.8 F/mol of electricity based on H_2N-Asp(O^{t}Bu)-Tag was passed, the reaction mixture was extracted with *c*-hex (20 mL x 2), and then the solvent was replaced with MeCN. The resulting precipitants were collected by filtration and washed with MeCN to give the product in 96% yield (dr =75:25, 294.4 mg) as white solid.

^{1}H NMR ($CDCl_3$, 600 MHz) δ 7.82 (1H, d, *J* = 7.6 Hz), 7.75 (2H, d, *J* = 7.6 Hz), 7.65-7.53 (2H, m), 7.41-7.34 (3H, m), 7.34-7.26 (11H, m), 7.16-7.05 (7H, m), 6.98 (0.25H, d, *J* = 5.5 Hz), 6.74 (0.75H, d, *J* = 6.2 Hz), 6.69-6.58 (1H, m), 6.45-6.27 (2H, m), 5.16-5.04 (2H, m), 4.83-4.78 (0.75H, m), 4.78-4.74 (0.25H, m), 4.54 (0.75H, dd, *J* = 12.7 Hz, 5.5 Hz), 4.50 (0.25H, dd, *J* = 11.7Hz, 5.5 Hz), 4.35-4.27 (2H, m), 4.24-4.12 (1H, m), 3.94-3.81 (4H, m), 3.12-2.94 (2H, m), 2.92-2.81 (1H, m), 2.76-2.69 (0.25H, m), 2.64 (0.75H, dd, *J* = 15.8 Hz, 3.4 Hz), 1.80-1.68 (4H, m), 1.45-1.21 (85H, m), 0.88 (6H, t, *J* = 6.9 Hz); ^{13}C NMR ($CDCl_3$, 150 MHz) δ 171.0, 170.6, 169.5, 160.6, 158.1, 156.3, 144.0, 144.0, 144.0, 143.9, 142.4, 141.2, 138.6, 138.4, 136.9, 136.8, 132.1, 132.1, 130.7, 129.8, 128.0, 127.6, 127.1, 127.1, 125.4, 125.3, 119.9, 119.6, 119.5, 116.0, 104.5, 99.6, 99.6, 81.5, 81.4, 75.3, 68.1, 68.0, 67.2, 62.7, 55.5, 55.0, 48.9, 47.1, 37.6, 37.5, 31.9, 30.6, 29.7, 29.7, 29.6, 29.6, 29.4, 29.4, 29.3, 29.1, 27.9, 27.9, 26.1, 26.0, 22.7, 14.1; HRMS (ESI-MS)

[M+Na]$^+$ calcd for $C_{99}H_{140}N_4O_9$ 1552.0513, found 1552.0528.

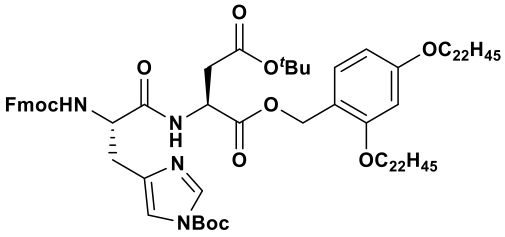

Fmoc-His(Boc)-Asp(O^{t}Bu)-Tag (**3qa**). Following the general procedure, 2,6-lutidine (69.9 µl, 0.60 mmol), Bu_4NClO_4 (136.8 mg, 0.40 mmol), Ph_3P (157.4 mg, 0.60 mmol), Fmoc-His(Boc)-OH (143.2 mg, 0.30 mmol), and H_2N-Asp(O^{t}Bu)-Tag (185.7 mg, 0.20 mmol) were added to Ar-purged MeCN (8.0 mL)-*c*-hex (4.0 mL). The solution was electrolyzed at constant current (0.67 mA/cm^2) under Ar at room temperature. After 4.8 F/mol of electricity based on H_2N-Asp(O^{t}Bu)-Tag was passed, the reaction mixture was extracted with *c*-hex (20 mL x 2), and then the solvent was replaced with MeCN. The resulting precipitants were collected by filtration and washed with MeCN to give the product in 99% yield (274.8 mg) as white solid.

^{1}H NMR ($CDCl_3$, 600 MHz) δ 8.00 (1H, s), 7.75 (2H, d, *J* = 7.6 Hz), 7.64-7.50 (3H, m), 7.39 (2H, t, *J* = 7.2 Hz), 7.30 (2H, t, *J* = 6.9 Hz), 7.20 (1H, s), 7.12 (1H, d, *J* = 8.3 Hz), 6.47 (1H, d, *J* = 6.9 Hz), 6.43-6.29 (2H, m), 5.14 (1H, d, *J* = 11.7 Hz), 5.12 (1H, d, *J* = 11.7 Hz), 4.82-4.77 (1H, m), 4.63-4.48 (1H, m), 4.34 (2H, d, *J* = 7.6 Hz), 4.28-4.18 (1H, m), 3.94-3.82 (4H, m), 3.12 (1H, dd, *J* = 15.0 Hz, 4.1 Hz), 2.99 (1H,dd, *J* = 14.4 Hz, 5.5 Hz), 2.87 (1H, dd, *J* = 16.5 Hz, 4.1 Hz), 2.63 (1H, dd, *J* = 16.5 Hz, 3.4 Hz), 1.78-1.69 (4H, m), 1.59 (9H, s), 1.48-1.23 (85H, m), 0.88 (6H, t, *J* = 6.9 Hz); ^{13}C NMR ($CDCl_3$, 150 MHz) δ 170.7, 170.4, 169.4, 160.7, 158.2, 156.1, 146.9, 144.0, 143.9, 141.3, 138.9, 136.8, 130.8, 127.6, 127.1, 127.1, 125.3, 125.3, 119.9, 116.0, 114.8, 104.5, 99.6, 85.5, 81.5, 68.1, 68.1, 67.3, 62.8, 54.6, 49.0, 47.1, 37.5, 31.9, 30.3, 29.7, 29.7, 29.6, 29.6, 29.4, 29.4, 29.3, 29.1, 27.9, 27.9, 26.1, 26.0, 22.7, 14.1; HRMS (ESI-MS) [M+Na]$^+$ calcd for $C_{85}H_{134}N_4O_{11}$ 1409.9941, found 1409.9961.

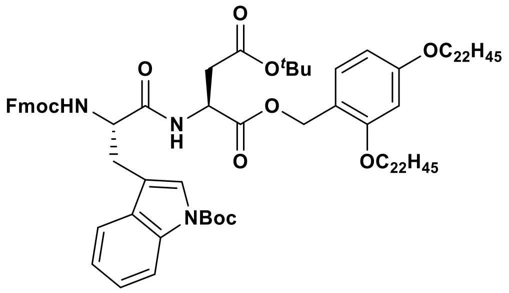

Fmoc-Trp(Boc)-Asp(O^{t}Bu)-Tag (**3ra**). Following the general procedure, 2,6-lutidine (69.9 μl, 0.60 mmol), Bu_4NClO_4 (136.8 mg, 0.40 mmol), Ph_3P (131.1 mg, 0.50 mmol), Fmoc-Trp(Boc)-OH (158.0 mg, 0.30 mmol), and H_2N-Asp(O^{t}Bu)-Tag (185.7 mg, 0.20 mmol) were added to Ar-purged MeCN (8.0 mL)-*c*-hex (4.0 mL). The solution was electrolyzed at constant current (0.67 mA/cm^2) under Ar at room temperature. After 4.8 F/mol of electricity based on H_2N-Asp(O^{t}Bu)-Tag was passed, the reaction mixture was extracted with *c*-hex (20 mL x 2), and then the solvent was replaced with MeCN. The resulting precipitants were collected by filtration and washed with MeCN to give the product in 94% yield (271.1 mg) as white solid.

^{1}H NMR ($CDCl_3$, 600 MHz) δ 8.13 (1H, s), 7.75 (2H, d, *J* = 7.6 Hz), 7.66 (1H, d, *J* = 7.6 Hz), 7.59-7.45 (3H, m), 7.42-7.34 (2H, m), 7.33-7.26 (3H, m), 7.22 (1H, t, *J* = 7.6 Hz), 7.14 (1H, d, *J* = 8.3 Hz), 6.74 (1H, d, *J* = 7.6 Hz), 6.44-6.31 (2H, m), 5.55 (1H, d, *J* = 6.9 Hz), 5.17 (1H, d, *J* = 11.7 Hz), 5.12 (1H, d, *J* = 11.7 Hz), 4.82-4.69 (1H, m), 4.63-4.50 (1H, m), 4.40-4.26 (2H, m), 4.25-4.13 (1H, m), 3.95-3.80 (4H, m), 3.33-3.09 (2H, m), 2.98-2.76 (1H, m), 2.71-2.54 (1H, m), 1.80-1.70 (4H, m), 1.64 (9H, s), 1.45-1.22 (85H, m), 0.88 (6H, t, *J* = 6.9 Hz); ^{13}C NMR ($CDCl_3$, 150 MHz) δ170.5, 170.2, 169.6, 160.8, 158.3, 155.8, 149.5, 143.8, 143.8, 141.2, 135.6, 130.9, 130.2, 127.7, 127.1, 125.1, 124.9, 124.5, 122.7, 119.9, 119.0, 115.9, 115.4, 114.9, 104.5, 99.6, 83.4, 81.7, 68.1, 68.1, 67.3, 62.9, 54.7, 48.8, 47.1, 37.2, 31.9, 29.7, 29.7, 29.6, 29.4, 29.4, 29.3, 29.1, 28.5, 28.2, 27.8, 26.0, 26.02, 22.7, 14.1; HRMS (ESI-MS) [M+Na]$^+$ calcd for $C_{90}H_{137}N_3O_{11}$ 1459.0145, found 1459.0140.

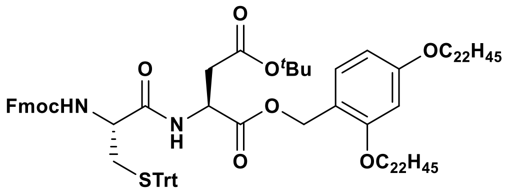

Fmoc-Cys(Trt)-Asp(O^{t}Bu)-Tag (**3sa**). Following the general procedure, 2,6-lutidine (69.9 μl, 0.60 mmol), Bu_4NClO_4 (136.8 mg, 0.40 mmol), Ph_3P (157.4 mg, 0.60 mmol), Fmoc-Cys(Trt)-OH (175.7 mg, 0.30 mmol), and H_2N-Asp(O^{t}Bu)-Tag (185.7 mg, 0.20 mmol) were added to Ar-purged MeCN (8.0 mL)-*c*-hex (4.0 mL). The solution was electrolyzed at constant current (0.67 mA/cm^2) under Ar at 40 °C. After 4.8 F/mol of electricity based on H_2N-Asp(O^{t}Bu)-Tag was passed, the reaction mixture was extracted with *c*-hex (20 mL x 2), and then the solvent was replaced with MeCN. The resulting precipitants were collected by filtration and washed with MeCN to give the product in 95% yield (278.2 mg) as white solid.

^{1}H NMR ($CDCl_3$, 600 MHz) δ 7.78-7.71 (2H, m), 7.62-7.49 (2H, m), 7.45-7.35 (8H, m), 7.30-7.23 (8H, m), 7.22-7.17 (3H, m), 7.11 (1H, d, *J* = 8.3 Hz), 6.85 (1H, d, *J* = 6.9 Hz), 6.43-6.30 (2H, m), 5.13 (2H, s), 5.01 (1H, d, *J* = 7.6 Hz), 4.72-4.66 (1H, m), 4.40-4.26 (2H, m), 4.19 (1H, t, *J* = 6.2 Hz), 3.94-3.84 (4H, m), 3.79 (1H, dd, *J* = 13.1 Hz, 7.6 Hz), 2.85 (1H, dd, *J* = 17.1 Hz, 4.1 Hz), 2.75-2.67 (2H, m), 2.63 (1H, dd, *J* = 13.1 Hz, 4.8 Hz), 1.74 (4H, tt, *J* = 6.9 Hz), 1.45-1.21 (85H, m), 0.88 (6H, t, *J* = 6.9 Hz); ^{13}C NMR ($CDCl_3$, 150 MHz) δ 170.3, 169.8, 169.6, 160.7, 158.2, 155.8, 144.4, 141.3, 130.8, 129.6, 128.1, 127.7, 127.1, 126.9, 125.1, 119.9, 115.9, 104.5, 99.6, 81.6, 68.1, 68.1, 67.3, 62.9, 53.9, 49.0, 47.1, 37.3, 33.9, 31.9, 29.7, 29.7, 29.6, 29.6, 29.4, 29.4, 29.3, 29.1, 27.9, 26.1, 26.0, 22.7, 14.1; HRMS (ESI-MS) [M+Na]$^+$ calcd for $C_{96}H_{138}N_2O_9S$ 1518.0015, found 1518.0006.

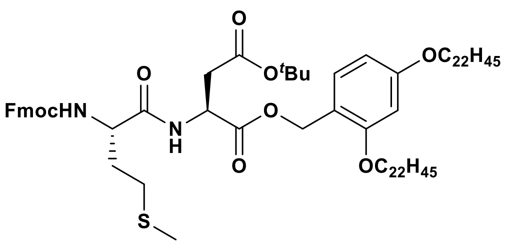

Fmoc-Met-Asp(O^{t}Bu)-Tag (**3ta**). Following the general procedure, 2,6-lutidine (69.9 μl, 0.60 mmol), Bu_4NClO_4 (136.8 mg, 0.40 mmol), Ph_3P (104.9 mg, 0.40 mmol), Fmoc-Met-OH (111.4 mg, 0.30 mmol), and H_2N-Asp(O^{t}Bu)-Tag (185.7 mg, 0.20 mmol) were added to Ar-purged MeCN (8.0 mL)-*c*-hex (4.0 mL). The solution was electrolyzed at constant current (0.67 mA/cm^2) under Ar at 50 °C. After 4.8 F/mol of electricity based on H_2N-Asp(O^{t}Bu)-Tag was passed, the reaction mixture was extracted with *c*-hex (20 mL x 2), and then the solvent was replaced with MeCN. The resulting precipitants were collected by filtration and washed with MeCN to give the product in 95% yield (242.7 mg) as white solid.

^{1}H NMR ($CDCl_3$, 600 MHz) δ 7.76 (2H, d, *J* = 7.6 Hz), 7.62-7.56 (2H, m), 7.40 (2H, t, *J* = 7.2 Hz), 7.34-7.28 (2H, m), 7.16 (1H, d, *J* = 7.6 Hz), 7.00 (1H, d, *J* = 8.9 Hz), 6.44-6.35 (2H, m), 5.59 (1H, d, *J* = 8.3 Hz), 5.18 (1H, d, *J* = 11.7 Hz), 5.14 (1H, d, *J* = 11.7 Hz), 4.88-4.80 (1H, m), 4.47-4.30 (3H, m), 4.21 (1H, t, *J* = 6.9 Hz), 3.96-3.87 (4H, m), 2.96 (1H, dd, *J* = 17.1 Hz, 4.8 Hz), 2.70 (1H, dd, *J* = 17.2 Hz, 4.8 Hz), 2.63-2.53(2H, m), 2.09 (3H, s), 2.08-2.02 (1H, m), 2.00-1.93 (1H, m), 1.80-1.71 (4H, m), 1.46-1.20 (85H, m), 0.88 (6H, t, *J* = 7.2 Hz); ^{13}C NMR ($CDCl_3$, 150 MHz) δ 170.7, 170.4, 169.9, 160.9, 158.4, 155.8, 143.9, 143.7, 141.3, 131.1, 127.7, 127.1, 125.1, 120.0, 120.0, 115.8, 104.6, 99.6, 81.9, 68.2, 68.1, 67.1, 63.1, 53.5, 48.7, 47.2, 37.3, 32.1, 31.9, 29.7, 29.7, 29.6, 29.6, 29.4, 29.4, 29.3, 29.2, 28.0, 26.1, 26.0, 22.7, 15.0, 14.1; HRMS (ESI-MS) $[M+Na]^+$ calcd for $C_{79}H_{128}N_2O_9S$ 1303.9233, found 1303.9230.

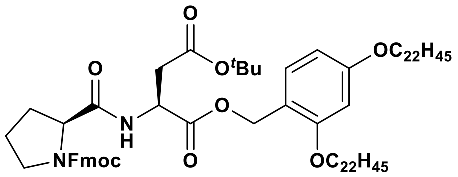

Fmoc-Pro-Asp(O^{t}Bu)-Tag (**3ua**). Following the general procedure, 2,6-lutidine (69.9 μl, 0.60 mmol), $NaClO_4$ (49.0 mg, 0.40 mmol), Ph_3P (209.8 mg, 0.80 mmol), Fmoc-Pro-OH (135.0 mg, 0.40 mmol), and H_2N-Asp(O^{t}Bu)-Tag (185.7 mg, 0.20 mmol) were added to Ar-purged MeCN (8.0 mL)-*c*-hex (4.0 mL). The solution was electrolyzed at constant current (0.67 mA/cm^2) under Ar at 60 °C. After 4.8 F/mol of electricity based on H_2N-Asp(O^{t}Bu)-Tag was passed, the reaction

mixture was extracted with *c*-hex (20 mL x 2), and then the solvent was replaced with MeCN. The resulting precipitants were collected by filtration and washed with MeCN to give the product in 98% yield (247.0 mg) as white solid.

^{1}H NMR ($CDCl_3$, 600 MHz) δ 7.73 (2H, d, J = 7.6 Hz), 7.63-7.48 (2H, m), 7.42-7.33 (2H, m), 7.32-7.25 (3H, m), 7.10 (1H, brs), 6.45-6.29 (2H, m), 5.21-5.04 (2H, m), 4.82-4.70 (1H, m), 4.44-4.15 (4H, m), 3.96-3.83 (4H, m), 3.59-3.40 (2H, m), 2.85 (1H, dd, J = 16.5 Hz, 4.8 Hz), 2.73 (1H, dd, J = 16.5 Hz, 4.1 Hz), 2.22 (1H, brs), 2.12-1.99 (1H, brs), 1.96-1.81 (2H, m), 1.79-1.69 (4H, m), 1.49-1.22 (85H, m), 0.88 (6H, t, J = 7.6 Hz); ^{13}C NMR ($CDCl_3$, 150 MHz) δ 171.8, 171.5, 170.7, 170.4, 169.8, 169.8, 160.7, 158.3, 155.7, 155.1, 144.3, 144.1, 143.9, 143.8, 141.3, 131.0, 127.7, 127.0, 125.2, 119.9, 116.3, 116.1, 115.8, 104.5, 104.5, 99.6, 81.6, 81.5, 68.1, 68.1, 67.9, 67.7, 63.0, 62.9, 60.9, 60.6, 48.9, 47.5, 47.2, 47.1, 47.0, 37.5, 37.4, 31.9, 31.3, 29.7, 29.7, 29.6, 29.6, 29.4, 29.4, 29.3, 29.1, 29.0, 27.9, 26.9, 26.1, 26.0, 24.5, 23.4, 22.7, 14.1; HRMS (ESI-MS) $[M+Na]^+$ calcd for $C_{79}H_{126}N_2O_9$ 1269.9356, found 1296.9367.

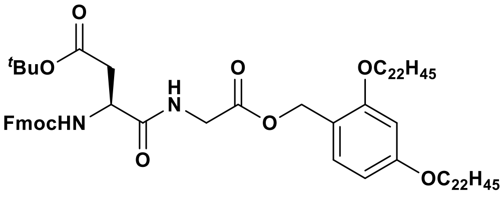

Fmoc-Asp(O^{t}Bu)-Gly-Tag (**3ab**). Following the general procedure, 2,6-lutidine (69.9 μl, 0.60 mmol), Bu_4NClO_4 (136.8 mg, 0.40 mmol), Ph_3P (131.1 mg, 0.50 mmol), Fmoc-Asp(O^{t}Bu)-OH (123.4 mg, 0.30 mmol), and H_2N-Gly-Tag (162.9 mg, 0.20 mmol) were added to Ar-purged MeCN(8.0 mL)-*c*-hex (4.0 mL). The solution was electrolyzed at constant current (0.67 mA/cm^2) under Ar at room temparature. After 4.8 F/mol of electricity based on H_2N-Gly-Tag was passed, the reaction mixture was extracted with *c*-hex (20 mL x 2), and then the solvent was replaced with MeCN. The resulting precipitants were collected by filtration and washed with MeCN to give the product in 97% yield (235.4 mg) as white solid.

^{1}H NMR ($CDCl_3$, 400 MHz) δ 7.76 (2H, d, *J* = 7.3 Hz), 7.59 (2H, d, *J* = 7.3 Hz), 7.40 (2H, t, *J* = 7.3 Hz), 7.35-7.27 (2H, m), 7.18 (1H, d, *J* = 8.2 Hz), 6.99 (1H, brs), 6.44-6.37 (2H, m), 5.96 (1H, d, *J* = 8.2 Hz), 5.16 (2H, s), 4.67-4.33 (3H, m), 4.23 (1H, t, *J* = 6.9 Hz), 4.10-3.87 (6H, m), 2.91 (1H, dd, *J* = 17.9 Hz, 4.1 Hz), 2.64 (1H, dd, *J* = 16.9 Hz, 6.4 Hz), 1.80-1.70 (4H, m), 1.48-1.20 (85H, m), 0.88 (6H, t, *J* = 6.9 Hz); ^{13}C NMR($CDCl_3$, 150 MHz) δ 170.5, 169.9, 168.4, 160.9, 158.4, 156.4, 143.8, 143.8, 141.3, 131.1, 127.7, 127.1, 125.1, 120.0, 115.9, 104.6, 99.6, 81.8, 68.2, 68.1, 67.3, 63.1, 48.8, 47.1, 44.3, 37.3, 31.9, 29.7, 29.7, 29.6, 29.6, 29.4, 29.4, 29.3, 29.2, 27.9, 26.1, 22.7, 14.1; HRMS (ESI-MS) $[M+Na]^+$ calcd for $C_{76}H_{122}N_2O_9$ 1229.9043, found 1229.9046.

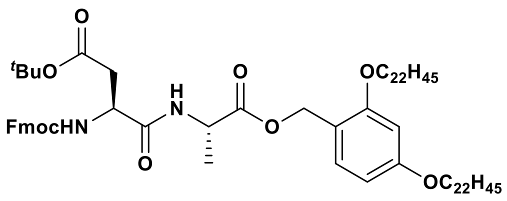

Fmoc-Asp(O^{t}Bu)-Ala-Tag (**3ac**). Following the general procedure, 2,6-lutidine (69.9 μl, 0.60 mmol), Bu_4NClO_4 (136.8 mg, 0.40 mmol), Ph_3P (131.1 mg, 0.50 mmol), Fmoc-Asp(O^{t}Bu)-OH (164.6 mg, 0.40 mmol), and H_2N-Ala-Tag (165.7 mg, 0.20 mmol) were added to Ar-purged MeCN(8.0 mL)-*c*-hex (4.0 mL). The solution was electrolyzed at constant current (0.67 mA/cm^2) under Ar at 50 °C. After 4.8 F/mol of electricity based on H_2N-Ala-Tag was passed, the reaction mixture was extracted with *c*-hex (20 mL x 2), and then the solvent was replaced with MeCN. The resulting precipitants were collected by filtration and washed with MeCN to give the product in 94% yield (230.4 mg) as white solid.

^{1}H NMR ($CDCl_3$, 600 MHz) δ 7.76 (2H, d, *J* = 7.6 Hz), 7.59 (2H, d, *J* = 6.9 Hz), 7.40 (2H, t, *J* = 7.6 Hz), 7.31 (2H, t, *J* = 7.6 Hz), 7.16 (1H, d, *J* = 8.3 Hz), 7.12 (1H, d, *J* = 6.9 Hz), 6.45-6.37 (2H, m), 5.97 (1H, d, *J* = 7.6 Hz), 5.18 (1H, d, *J* = 11.7 Hz), 5.09 (1H, d, *J* = 11.7 Hz), 4.60-4.49 (2H, m), 4.45-4.36 (2H, m), 4.23 (1H, t, *J* = 6.9 Hz), 3.96-3.88 (4H, m), 2.92 (1H, dd, *J* = 17.2 Hz, 4.1 Hz), 2.60 (1H, dd, *J* = 17.2 Hz, 6.9 Hz), 1.79-1.71 (4H, m), 1.53-1.22 (88H, m), 0.88 (6H, t, *J* = 6.9 Hz); ^{13}C NMR($CDCl_3$, 150 MHz) δ 172.5, 171.3, 170.0, 160.9, 158.4, 155.9, 143.8, 143.7,

141.3, 131.1, 127.8, 127.1, 125.1, 120.0, 115.9, 104.5, 99.7, 81.9, 68.1, 67.3, 62.9, 50.9, 48.5, 47.1, 37.7, 31.9, 29.7, 29.7, 29.6, 29.6, 29.4, 29.4, 29.3, 29.2, 28.0, 26.0, 26.0, 22.7, 18.3, 14.1; HRMS (ESI-MS) $[M+Na]^+$ calcd for $C_{77}H_{124}N_2O_9$ 1243.9199, found 1243.9189.

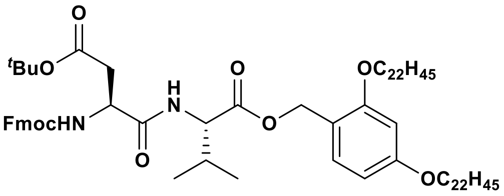

Fmoc-Asp(O^{t}Bu)-Val-Tag (**3ad**). Following the general procedure, 2,6-lutidine (69.9 μl, 0.60 mmol), Bu_4NClO_4 (136.8 mg, 0.40 mmol), Ph_3P (131.1 mg, 0.50 mmol), Fmoc-Asp(O^{t}Bu)-OH (123.4 mg, 0.30 mmol), and H_2N-Val-Tag (171.3 mg, 0.20 mmol) were added to Ar-purged MeCN(8.0 mL)-*c*-hex (4.0 mL). The solution was electrolyzed at constant current (0.67 mA/cm^2) under Ar at 60 °C. After 4.8 F/mol of electricity based on H_2N-Val-Tag was passed, the reaction mixture was extracted with *c*-hex (20 mL x 2), and then the solvent was replaced with MeCN. The resulting precipitants were collected by filtration and washed with MeCN to give the product in 98% yield (246.9 mg) as white solid.

^{1}H NMR ($CDCl_3$, 400 MHz) δ 7.76 (2H, d, *J* = 7.3 Hz), 7.59 (2H, d, *J* = 7.3 Hz), 7.40 (2H, t, *J* = 7.3 Hz), 7.31 (2H, t, *J* = 7.3 Hz), 7.22-7.05 (2H, m), 6.47-6.34 (2H, m), 6.05 (1H, d, *J* = 8.2 Hz), 5.18 (1H, d, *J* = 11.9 Hz), 5.06 (1H, d, *J* = 11.9 Hz), 4.64-4.54 (1H, m), 4.50 (1H, dd, *J* = 8.7 Hz, 4.6 Hz), 4.45-4.33 (2H, m), 4.23 (1H , t, *J* = 6.9 Hz), 3.98-3.85 (4H, m), 2.92 (1H, dd, *J* = 17.4 Hz, 4.1 Hz), 2.61 (1H, dd, *J* = 17.4 Hz, 7.3 Hz), 2.26-2.12 (1H, m), 1.81-1.68 (4H, m), 1.49-1.19 (85H, m), 0.95-0.81 (12H, m); ^{13}C NMR($CDCl_3$, 100 MHz) δ 171.6, 171.4, 170.5, 160.9, 158.5, 155.9, 143.9, 143.7, 141.3, 131.4, 127.7, 127.1, 125.1, 120.0, 116.0, 104.5, 99.7, 81.9, 68.1, 67.3, 62.7, 57.5, 50.9, 47.1, 37.7, 31.9, 31.1, 29.7, 29.6, 29.4, 29.4, 29.3, 29.2, 28.0, 26.1, 26.0, 22.7, 19.0, 17.5, 14.1; HRMS (ESI-MS) $[M+Na]^+$ calcd for $C_{79}H_{128}N_2O_9$ 1271.9512, found 1271.9505.

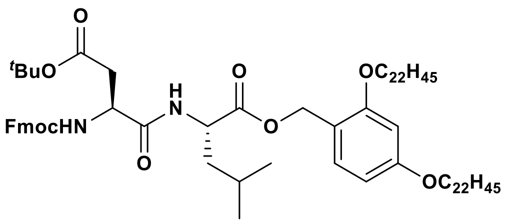

Fmoc- Asp(O^{t}Bu)-Leu-Tag (**3ae**). Following the general procedure, 2,6-lutidine (69.9 μl, 0.60 mmol), Bu_4NClO_4 (136.8 mg, 0.40 mmol), Ph_3P (131.1 mg, 0.50 mmol), Fmoc-Asp(O^{t}Bu)-OH (123.4 mg, 0.30 mmol), and H_2N-Leu-Tag (174.1 mg, 0.20 mmol) were added to Ar-purged MeCN(8.0 mL)-*c*-hex (4.0 mL). The solution was electrolyzed at constant current (0.67 mA/cm^2) under Ar at 50 °C. After 4.8 F/mol of electricity based on H_2N-Leu-Tag was passed, the reaction mixture was extracted with *c*-hex (20 mL x 2), and then the solvent was replaced with MeCN. The resulting precipitants were collected by filtration and washed with MeCN to give the product in 96% yield (241.9 mg) as white solid.

^{1}H NMR ($CDCl_3$, 400 MHz) δ 7.76 (2H, d, *J* = 7.3 Hz), 7.59 (2H, t, *J* =7.3 Hz), 7.40 (2H, t, *J* = 7.3 Hz), 7.35-7.27 (2H, m), 7.16 (1H, d, *J* = 7.8 Hz), 6.99 (1H, d, *J* =7.8 Hz), 6.46-6.35 (2H, m), 6.01 (1H, d, *J* =8.2 Hz), 5.16 (1H, d, *J* =11.9 Hz), 5.07 (1H, d, *J* =11.9 Hz), 4.65-4.50 (2H, m), 4.40 (2H, d, *J* =7.3 Hz), 4.22 (1H, t, *J* =7.1 Hz), 3.92 (4H,t, *J* =6.4 Hz), 2.92 (1H, dd, *J* =17.4 Hz, 4.1 Hz), 2.59 (1H, dd, *J* =16.9 Hz, 6.9 Hz), 1.81-1.51 (7H, m), 1.50-1.19 (85H, m), 0.92-0.82 (12H, m); ^{13}C NMR($CDCl_3$, 100 MHz) δ 172.5, 171.5, 170.2, 160.8, 158.4, 155.9, 143.8, 143.7, 141.3, 131.1, 127.8, 127.1, 125.1, 120.0, 116.0, 104.5, 99.6, 81.9, 68.1, 67.3, 62.7, 51.2, 50.9, 47.1, 41.5, 37.7, 31.9, 30.0, 29.7, 29.6, 29.6, 29.4, 29.4, 29.3, 29.2, 28.0, 26.1, 26.0, 24.7, 22.8, 22.7, 21.9, 14.1; HRMS (ESI-MS) [M+Na]$^+$ calcd for $C_{80}H_{130}N_2O_9$ 1285.9669, found 1285.9652.

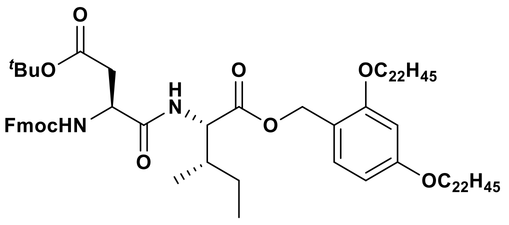

Fmoc- Asp(O^{t}Bu)-Ile-Tag (**3fa**). Following the general procedure, 2,6-lutidine (69.9 μl, 0.60

mmol), Bu_4NClO_4 (136.8 mg, 0.40 mmol), Ph_3P (131.1 mg, 0.50 mmol), Fmoc-Asp(O^{t}Bu)-OH (123.4 mg, 0.30 mmol), and H_2N-Ile-Tag (188.0 mg, 0.20 mmol) were added to Ar-purged MeCN(8.0 mL)-*c*-hex (4.0 mL). The solution was electrolyzed at constant current (0.67 mA/cm^2) under Ar at 60 °C. After 4.8 F/mol of electricity based on H_2N-Ile-Tag was passed, the reaction mixture was extracted with *c*-hex (20 mL x 2), and then the solvent was replaced with MeCN. The resulting precipitants were collected by filtration and washed with MeCN to give the product in 93% yield (234.4 mg) as white solid.

^{1}H NMR (CDCl$_3$, 400 MHz) δ 7.76 (2H, d, *J* = 7.3 Hz), 7.59 (2H, d, *J* = 7.3 Hz), 7.40 (2H, t, *J* = 7.3 Hz), 7.35-7.27 (2H, m), 7.22-7.07 (2H, m), 6.46-6.31 (2H, m), 6.05 (1H, d, *J* = 7.8 Hz), 5.17 (1H, d, *J* = 11.9 Hz), 5.06 (1H, d, *J* = 11.9 Hz), 4.63-4.48 (2H, m), 4.46-4.32 (2H, m), 4.23 (1H, t, *J* = 6.9 Hz), 3.91 (4H, t, *J* = 6.4 Hz), 2.91 (1H, dd, *J* = 17.4 Hz, 4.1 Hz), 2.61 (1H, dd, *J* = 17.4 Hz, 6.9 Hz), 1.98-1.82 (1H, m), 1.82-1.66 (4H, m), 1.49-1.20 (86H, m), 1.2-1.07 (1H, m), 0.94-0.79 (12H, m); ^{13}C NMR(CDCl$_3$, 100 MHz) δ 171.6, 171.4, 170.3, 160.9, 158.4, 155.9, 143.9, 143.7, 141.3, 131.4, 127.7, 127.1, 125.1, 120.0, 116.0, 104.4, 99.6, 81.9, 68.1, 68.1, 67.3, 62.6, 56.9, 50.9, 47.1, 37.7, 31.9, 30.0, 29.7, 29.6, 29.4, 29.4, 29.3, 29.2, 28.0, 26.1, 24.9, 22.7, 15.5, 14.1, 11.7; HRMS (ESI-MS) [M+Na]$^+$ calcd for $C_{80}H_{130}N_2O_9$ 1285.9669, found 1285.9656.

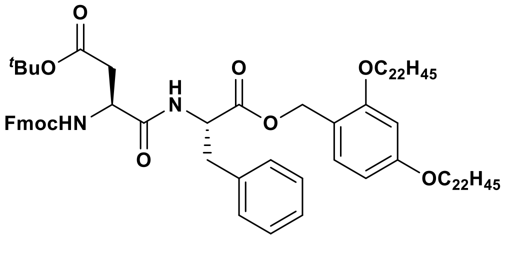

Fmoc-Asp(O^{t}Bu)-Phe-Tag (**3ag**). Following the general procedure, 2,6-lutidine (69.9 μl, 0.60 mmol), Bu_4NClO_4 (136.8 mg, 0.40 mmol), Ph_3P (131.1 mg, 0.50 mmol), Fmoc-Asp(O^{t}Bu)-OH (164.6 mg, 0.40 mmol), and H_2N-Phe-Tag (180.9 mg, 0.20 mmol) were added to Ar-purged MeCN(8.0 mL)-*c*-hex (4.0 mL). The solution was electrolyzed at constant current (0.67 mA/cm^2) under Ar at 40 °C. After 4.8 F/mol of electricity based on H_2N-Phe-Tag was passed, the reaction mixture was extracted with *c*-hex (20 mL x 2), and then the solvent was replaced

with MeCN. The resulting precipitants were collected by filtration and washed with MeCN to give the product in 95% yield (245.9 mg) as white solid.

^{1}H NMR ($CDCl_3$, 400 MHz) δ 7.76 (2H, d, *J* = 7.3 Hz), 7.57 (2H, d, *J* = 7.3 Hz), 7.40 (2H, t, *J* = 7.3 Hz), 7.30 (2H, t, *J* = 7.3 Hz), 7.23-7.15 (3H, m), 7.12 (1H, d, *J* = 8.7 Hz), 7.09-7.03 (2H, m), 6.99 (1H, d, *J* = 7.3 Hz), 6.48-6.35 (2H, m), 5.92 (1H, d, *J* = 8.2 Hz), 5.17 (1H, d, *J* = 11.9 Hz), 5.09 (1H, d, *J* = 11.9 Hz), 4.88-4.76 (1H, m), 4.63-4.44 (1H, m), 4.43-4.28 (2H, m), 4.20 (1H, t, *J* = 6.9 Hz), 4.01-3.87 (4H, m), 3.22-3.00 (2H, m), 2.87 (1H, dd, *J* =16.9 Hz, 4.1 Hz), 2.59 (1H, dd, *J* = 16.9 Hz, 6.4 Hz), 1.87-1.66 (4H, m), 1.50-1.20 (85H, m), 0.88 (6H, t, *J* = 6.9 Hz); ^{13}C NMR($CDCl_3$, 100 MHz) δ 171.2, 170.9, 170.1, 161.0, 158.5, 155.9, 143.8, 143.7, 141.3, 135.7, 131.6, 129.4, 128.5, 127.7, 127.1, 127.0, 125.1, 120.0, 115.8, 104.5, 99.7, 81.8, 68.2, 68.1, 67.3, 62.9, 53.5, 50.9, 47.1, 37.6, 37.4, 31.9, 30.0, 29.7, 29.6, 29.4, 29.4, 29.3, 29.2, 28.0 26.1, 22.7, 14.1; HRMS (ESI-MS) $[M+Na]^+$ calcd for $C_{83}H_{128}N_2O_9$ 1319.9512, found 1319.9483.

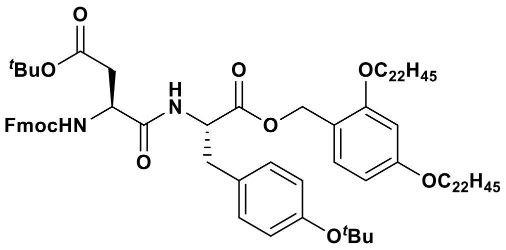

Fmoc-Asp(O^{t}Bu)-Tyr(O^{t}Bu)-Tag (**3ah**). Following the general procedure, 2,6-lutidine (69.9 μl, 0.60 mmol), Bu_4NClO_4 (136.8 mg, 0.40 mmol), Ph_3P (131.1 mg, 0.50 mmol), Fmoc-Asp(O^{t}Bu)-OH (164.6 mg, 0.40 mmol), and H_2N- Tyr(O^{t}Bu)-Tag (195.3 mg, 0.20 mmol) were added to Ar-purged MeCN(8.0 mL)-*c*-hex (4.0 mL). The solution was electrolyzed at constant current (0.67 mA/cm^2) under Ar at room temparature. After 4.8 F/mol of electricity based on H_2N-Tyr(O^{t}Bu)-Tag was passed, the reaction mixture was extracted with *c*-hex (20 mL x 2), and then the solvent was replaced with MeCN. The resulting precipitants were collected by filtration and washed with MeCN to give the product in 95% yield (261.4 mg) as white solid.

^{1}H NMR ($CDCl_3$, 400 MHz) δ 7.76 (2H, d, *J* = 7.3 Hz), 7.58 (2H, d, *J* = 7.3 Hz), 7.40 (2H, t, *J* = 7.3 Hz), 7.31 (2H, t, *J* = 7.3 Hz), 7.13 (1H, d, *J* = 8.2 Hz), 7.00 (1H, d, *J* = 7.8 Hz), 6.95 (2H, d,

J = 8.2 H), 6.82 (2H, d, J = 8.2 Hz), 6.47-6.36 (2H, m), 5.92 (1H, d, J = 7.8 Hz), 5.16 (1H, d, J = 11.9 Hz), 5.09 (1H, d, J = 11.9 Hz), 4.85-4.75 (1H, m), 4.59-4.45 (1H, m), 4.42-4.29 (2H, m), 4.22 (1H, t, J = 7.3 Hz), 3.99-3.88 (4H, m), 3.13-2.97 (2H, m), 2.87 (1H, dd, J = 17.4 Hz, 3.7 Hz), 2.59 (1H, dd, J = 17.4 Hz, 6.9 Hz), 1.84-1.71 (4H, m), 1.53-1.20 (94H, m), 0.88 (6H, t, J = 6.9 Hz); ^{13}C NMR(CDCl$_3$, 100 MHz) δ 171.2, 171.0, 170.0, 161.0, 158.5, 155.9, 154.4, 143.9, 143.7, 141.3, 131.6, 130.4, 129.9, 127.7, 127.1, 125.2, 124.1, 120.0, 115.9, 104.5, 99.6, 81.8, 78.2, 68.2, 68.1, 67.3, 62.8, 53.6, 50.9, 47.1, 37.5, 37.0, 31.9, 30.0, 29.7, 29.6, 29.4, 29.4, 29.3, 29.2, 28.8, 28.0, 26.1, 22.7, 14.1; HRMS (ESI-MS) [M+Na]$^+$ calcd for $C_{87}H_{136}N_2O_{10}$ 1392.0087, found 1392.0067.

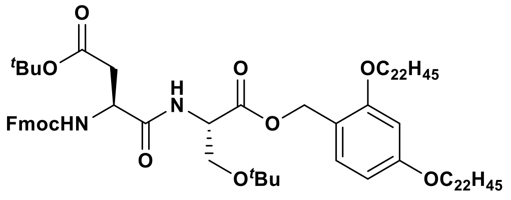

Fmoc-Asp(O^{t}Bu)-Ser(O^{t}Bu)-Tag (**3ai**). Following the general procedure, 2,6-lutidine (69.9 µl, 0.60 mmol), Bu_4NClO_4 (136.8 mg, 0.40 mmol), Ph_3P (131.1 mg, 0.50 mmol), Fmoc-Asp(O^{t}Bu)-OH (123.4 mg, 0.30 mmol), and H_2N-Ser(O^{t}Bu)-Tag (180.1 mg, 0.20 mmol) were added to Ar-purged MeCN(8.0 mL)-*c*-hex (4.0 mL). The solution was electrolyzed at constant current (0.67 mA/cm^2) under Ar at 40 °C. After 4.8 F/mol of electricity based on H_2N-Ser(O^{t}Bu)-Tag was passed, the reaction mixture was extracted with *c*-hex (20 mL x 2), and then the solvent was replaced with MeCN. The resulting precipitants were collected by filtration and washed with MeCN to give the product in 98% yield (254.6 mg) as white solid.

^{1}H NMR (CDCl$_3$, 400 MHz) δ 7.76 (2H, d, J = 7.3 Hz), 7.59 (2H, d, J = 7.3 Hz), 7.43-7.34 (3H, m), 7.31 (2H, td, J = 7.3 Hz, 0.9 Hz), 7.18 (1H, d, J = 8.2 Hz), 6.44-6.34 (2H, m), 5.99 (1H, d, J = 7.8 Hz), 5.16 (2H, s), 4.71-4.55 (2H, m), 4.38 (2H, d, J = 7.3 Hz), 4.23 (1H, t, J = 7.3 Hz), 3.98-3.86 (4H, m), 3.83 (1H, dd, J = 8.7 Hz, 2.8 Hz), 3.54 (1H, dd, J = 8.7 Hz, 3.2 Hz), 2.87 (1H, dd, J = 17.4 Hz, 4.1 Hz), 2.66 (1H, dd, J = 16.9 Hz, 7.3 Hz), 1.82-1.70 (4H, m), 1.48-.20 (85H, m),

1.10 (9H, m), 0.88 (6H, t, *J* = 6.9 Hz); ^{13}C NMR($CDCl_3$, 100 MHz) δ 171.1, 170.5, 167.0, 160.6, 158.2, 155.8, 143.9, 143.8, 141.3, 130.8, 127.7, 127.1, 125.1, 120.0, 116.3, 104.6, 99.6, 81.8, 73.4, 68.1, 68.1, 67.3, 62.4, 61.6, 53.2, 50.7, 47.1, 38.0, 31.9, 29.7, 29.6, 29.4, 29.4, 29.3, 29.2, 28.1, 27.2, 26.1, 22.7, 14.1; HRMS (ESI-MS) $[M+Na]^+$ calcd for $C_{81}H_{132}N_2O_{10}$ 1315.9774, found 1315.9763.

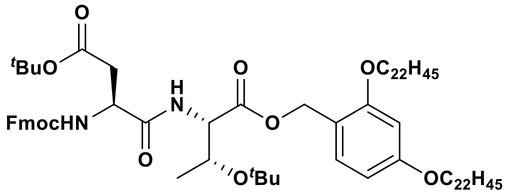

Fmoc-Asp(O^{t}Bu)-Thr(O^{t}Bu)-Tag (**3aj**). Following the general procedure, 2,6-lutidine (69.9 μl, 0.60 mmol), Bu_4NClO_4 (136.8 mg, 0.40 mmol), Ph_3P (131.1 mg, 0.50 mmol), Fmoc-Asp(O^{t}Bu)-OH (123.4 mg, 0.30 mmol), and H_2N-Thr(O^{t}Bu)-Tag (182.9 mg, 0.20 mmol) were added to Ar-purged MeCN(8.0 mL)-*c*-hex (4.0 mL). The solution was electrolyzed at constant current (0.67 mA/cm^2) under Ar at 40 °C. The product was obtained in 95% yield (249.5 mg) as white solid.

^{1}H NMR ($CDCl_3$, 400 MHz) δ 7.76 (2H, d, *J* = 7.3 Hz), 7.60 (2H, d, *J* = 7.3 Hz), 7.39 (2H, t, *J* = 7.3 Hz), 7.35-7.27 (3H, m), 7.18 (1H, d, *J* = 8.7 Hz), 4.46-6.37 (2H, m), 6.03 (1H, d, *J* = 7.8 Hz), 5.15 (1H, d, *J* = 11.9 Hz), 5.03 (1H, d, *J* = 11.9 Hz), 4.75-4.57 (1H, m), 4.47 (1H, dd, *J* = 9.2 Hz, 1.4 Hz), 4.43-4.30 (2H, m), 4.29-4.17 (2H, m), 3.99-3.85 (4H, m), 2.88 (1H, dd, *J* = 17.4 Hz, 4.1 Hz), 2.66 (1H, dd, *J* = 17.4 Hz, 6.9 Hz), 1.81-1.70 (4H, m), 1.49-1.21(85H, m), 1.16 (3H, d, *J* = 6.4 Hz), 1.08 (9H, s), 0.88 (6H, t, *J* = 6.9 Hz); ^{13}C NMR($CDCl_3$, 100 MHz) δ 171.2, 171.0, 170.4, 160.8, 158.4, 155.8, 143.9, 143.8, 141.3, 131.4, 127.7, 127.1, 125.2, 120.0, 115.9, 104.6, 99.6, 81.6, 73.9, 68.1, 68.1, 67.3, 67.1, 62.6, 58.2, 50.9, 47.2, 38.1, 31.9, 29.7, 29.6, 29.4, 29.4, 29.3, 29.1, 28.3, 28.1, 26.1, 22.7, 21.1, 20.0, 14.1; HRMS (ESI-MS) $[M+Na]^+$ calcd for $C_{82}H_{134}N_2O_{10}$ 1329.9931, found 1329.9918.

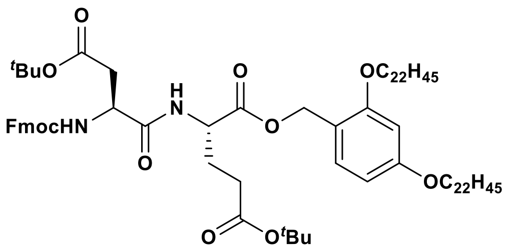

Fmoc-Asp(O^{t}Bu)-Glu(O^{t}Bu)-Tag (**3ak**). Following the general procedure, 2,6-lutidine (69.9 μl, 0.60 mmol), Bu_4NClO_4 (136.8 mg, 0.40 mmol), Ph_3P (131.1 mg, 0.50 mmol), Fmoc-Asp(O^{t}Bu)-OH (164.6 mg, 0.40 mmol), and H_2N-Glu(O^{t}Bu)-Tag (188.6 mg, 0.20 mmol) were added to Ar-purged MeCN(8.0 mL)-*c*-hex (4.0 mL). The solution was electrolyzed at constant current (0.67 mA/cm^2) under Ar at room temparature. After 4.8 F/mol of electricity based on H_2N-Thr(O^{t}Bu)-Tag was passed, the reaction mixture was extracted with *c*-hex (20 mL x 2), and then the solvent was replaced with MeCN. The resulting precipitants were collected by filtration and washed with MeCN to give the product in 95% yield (254.3 mg) as white solid.

^{1}H NMR ($CDCl_3$, 600 MHz) δ 7.76 (2H, d, J = 7.6 Hz), 7.66-7.51 (2H, m), 7.40 (2H, t, J = 7.6 Hz), 7.31 (2H, t, J = 7.6 Hz), 7.25-7.11 (2H, m), 6.46-6.34 (2H, m), 5.99 (1H, d, J = 8.3 Hz), 5.19 (1H, d, J = 11.7 Hz), 5.09 (1H, d, J = 11.7 Hz), 4.65-4.52 (2H, m), 4.41 (2H, d, J = 6.9 Hz), 4.23 (1H, t, J = 6.9 Hz), 3.97-3.85 (4H, m), 2.96 (1H, dd, J = 17.3 Hz, 3.4 Hz), 2.59 (1H, dd, J = 17.3 Hz, 5.5 Hz), 2.35-2.11 (3H, m), 2.01-1.88 (1H, m), 1.80-1.71 (4H, m), 1.53-1.20 (94H, m), 0.88 (6H, t, J = 6.9 Hz); ^{13}C NMR($CDCl_3$, 150 MHz) δ 172.0, 171.4, 170.4, 160.9, 158.4, 155.9, 143.9, 143.7, 141.3, 131.2, 127.8, 127.1, 125.1, 120.0, 115.9, 104.5, 99.7, 81.9, 80.5, 68.1, 68.1, 67.3, 63.0, 52.1, 51.0, 47.1, 37.6, 31.9, 31.2, 29.7, 29.7, 29.6, 29.4, 29.4, 29.3, 29.1, 28.1, 27.5, 26.1, 26.0, 22.7, 14.1; HRMS (ESI-MS) [M+Na]$^+$ calcd for $C_{83}H_{134}N_2O_{11}$ 1357.9880, found 1357.9891.

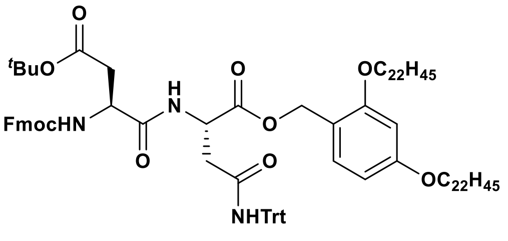

Fmoc-Asp(O^{t}Bu)-Asn(Trt)-Tag (**3al**). Following the general procedure, 2,6-lutidine (69.9 μl, 0.60

mmol), Bu_4NClO_4 (136.8 mg, 0.40 mmol), Ph_3P (131.1 mg, 0.50 mmol), Fmoc-Asp(O^{t}Bu)-OH (123.4 mg, 0.30 mmol), and H_2N-Asn(Trt)-Tag (222.8 mg, 0.20 mmol) were added to Ar-purged MeCN(8.0 mL)-*c*-hex (4.0 mL). The solution was electrolyzed at constant current (0.67 mA/cm^2) under Ar at room temparature. After 4.8 F/mol of electricity based on H_2N-Asn(Trt)-Tag was passed, the reaction mixture was extracted with *c*-hex (20 mL x 2), and then the solvent was replaced with MeCN. The resulting precipitants were collected by filtration and washed with MeCN to give the product in 96% yield (289.9 mg) as white solid.

^{1}H NMR ($CDCl_3$, 400 MHz) δ 7.75 (2H, d, J = 7.3 Hz), 7.63-7.43 (3H, m), 7.37 (2H, d, J = 7.3 Hz), 7.29-7.10 (17H, m), 7.04 (1H, d, J = 8.2 Hz), 6.70 (1H, s), 6.39 (1H, d, J = 1.8 Hz), 6.31 (1H, dd, J = 8.2 Hz, 1.8 Hz), 5.78 (1H, d, J = 8.2 Hz), 5.13 (1H, d, J = 11.9 Hz), 5.06 (1H, d, J = 11.9 Hz), 4.83-4.74 (1H, m), 4.60-4.46 (1H, m), 4.32 (2H, d, J = 7.3 Hz), 4.18 (1H, t, J = 7.3 Hz), 3.95-3.79 (4H, m), 3.03 (1H, dd, J = 15.6 Hz, 4.6 Hz), 2.89-2.70 (2H, m), 2.60 (1H, dd, J = 16.5 Hz, 5.0 Hz), 1.79-1.68 (4H, m), 1.46-1.20 (85H, m), 0.88 (6H, t, J = 6.9 Hz); ^{13}C NMR($CDCl_3$, 100 MHz) δ 171.5, 171.4, 170.9, 170.7, 160.9, 158.4, 156.0, 144.7, 143.8, 143.6, 141.3, 131.2, 128.7, 127.8, 127.8, 127.1, 126.9, 125.1, 120.0, 115.8, 104.6, 99.7, 81.9, 70.5, 68.2, 68.1, 67.3, 63.0, 52.0, 47.1, 37.4, 33.3, 31.9, 29.7, 29.4, 29.4, 29.3, 29.1, 28.0, 26.1, 26.0, 22.7, 14.1; HRMS (ESI-MS) [M+Na]$^+$ calcd for $C_{97}H_{139}N_3O_{10}$ 1529.0353, found 1529.0337.

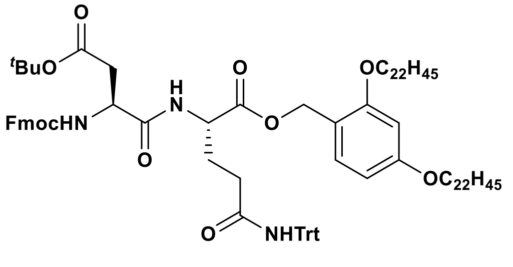

Fmoc-Asp(O^{t}Bu)-Gln(Trt)-Tag (**3am**). Following the general procedure, 2,6-lutidine (69.9 μl, 0.60 mmol), Bu_4NClO_4 (136.8 mg, 0.40 mmol), Ph_3P (131.1 mg, 0.50 mmol), Fmoc-Asp(O^{t}Bu)-OH (123.4 mg, 0.30 mmol), and H_2N-Asn(Trt)-Tag (225.6 mg, 0.20 mmol) were added to Ar-

purged MeCN(8.0 mL)-*c*-hex (4.0 mL). The solution was electrolyzed at constant current (0.67 mA/cm^2) under Ar at room temparature. After 4.8 F/mol of electricity based on H_2N-Gln(Trt)-Tag was passed, the reaction mixture was extracted with *c*-hex (20 mL x 2), and then the solvent was replaced with MeCN. The resulting precipitants were collected by filtration and washed with MeCN to give the product in 93% yield (282.0 mg) as white solid.

^{1}H NMR ($CDCl_3$, 400 MHz) δ 7.75 (2H, d, *J* = 7.3 Hz), 7.62-7.50 (2H, m), 7.39 (2H, t, *J* = 7.3 Hz), 7.33-7.26 (2H, m), 7.24-7.13 (18H, m), 6.43-6.33 (2H, m), 5.85 (1H, d, *J* = 8.2 Hz), 5.17 (1H, d, *J* = 11.9 Hz), 5.10 (1H, d, *J* = 11.9 Hz), 4.66-4.56 (1H, m), 4.56-4.46 (1H, m), 4.38 (2H, d, *J* = 6.0 Hz), 4.20 (1H, t, *J* = 6.9 Hz), 3.95-3.82 (4H, m), 2.94 (1H, dd, *J* = 17.5 Hz, 4.1 H), 2.59 (1H, dd, *J* = 17.5 Hz, 4.6 Hz), 2.41-2.16 (3H, m), 1.95-1.81 (1H, m), 1.80-1.67 (4H, m), 1.49-1.20 (85H, m), 0.88 (6H, t, *J* = 6.9 Hz); ^{13}C NMR($CDCl_3$, 100 MHz) δ 170.5, 170.4, 169.1, 160.6, 158.0, 155.9, 144.4, 144.0, 143.7, 141.2, 130.7, 128.7, 128.0, 127.7, 127.1, 125.2, 119.9, 116.0, 104.6, 99.6, 81.6, 70.9, 68.1, 68.1, 67.3, 62.9, 51.4, 49.5, 47.0, 38.3, 37.9, 31.9, 30.0, 29.7, 29.4, 29.4, 29.3, 29.1, 28.0, 26.1, 26.0, 22.7, 14.1; HRMS (ESI-MS) [M+Na]$^+$ calcd for $C_{98}H_{141}N_3O_{10}$ 1543.0509, found 1543.0511.

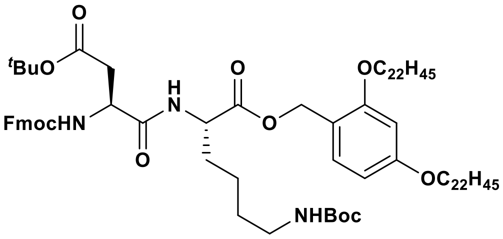

Fmoc-Asp(O^{t}Bu)-Lys(Boc)-Tag (**3an**). Following the general procedure, 2,6-lutidine (69.9 μl, 0.60 mmol), Bu_4NClO_4 (136.8 mg, 0.40 mmol), Ph_3P (131.1 mg, 0.50 mmol), Fmoc-Asp(O^{t}Bu)-OH (123.4 mg, 0.30 mmol), and H_2N-Lys(Boc)-Tag (197.1 mg, 0.20 mmol) were added to Ar-purged MeCN(8.0 mL)-*c*-hex (4.0 mL). The solution was electrolyzed at constant current (0.67 mA/cm^2) under Ar at 50 °C. After 4.8 F/mol of electricity based on H_2N-Lys(Boc)-Tag was passed, the reaction mixture was extracted with *c*-hex (20 mL x 2), and then the solvent was replaced with

MeCN. The resulting precipitants were collected by filtration and washed with MeCN to give the product in 98% yield (255.6 mg) as white solid.

^{1}H NMR ($CDCl_3$, 400 MHz) δ 7.76 (2H, d, J = 7.3 Hz), 7.59 (2H, d, J = 7.3 Hz), 7.39 (2H, t, J = 7.3 Hz), 7.35-7.27 (2H, m), 7.16 (1H, d, J = 8.2 Hz), 7.08 (1H, d, J = 7.8 Hz), 6.45-6.36 (2H, m), 6.15-5.83 (1H, m), 5.17 (1H, d, J = 11.9 Hz), 5.09 (1H, d, J = 11.9 Hz), 4.68-4.49 (3H, m), 4.41 (2H, d, J = 7.3 Hz), 4.23 (1H, t, J = 6.9 Hz), 3.97-3.86 (4H, m), 3.09-2.97 (2H, m), 2.93 (1H, dd, J = 17.4 Hz, 4.6 Hz), 2.62 (1H, dd, J = 17.4 Hz), 1.91-1.62 (6H, m), 1.50-1.21 (98H, m), 0.88 (6H, t, J = 6.9 Hz); ^{13}C NMR($CDCl_3$, 100 MHz) δ 171.8, 171.3, 170.3, 160.9, 158.4, 156.0, 155.9, 143.9, 143.7, 141.3, 131.2, 127.8, 127.1, 125.1, 120.0, 115.9, 104.6, 99.7, 81.9, 79.0, 68.2, 68.1, 67.3, 62.8, 52.3, 51.0, 47.1, 40.23, 37.6, 31.9, 29.7, 29.6, 29.4, 29.4, 29.3, 29.2, 28.4, 28.0, 26.1, 26.0, 22.7, 22.2, 14.1; HRMS (ESI-MS) $[M+Na]^+$ calcd for $C_{85}H_{139}N_3O_{11}$ 1401.0302, found 1401.0276.

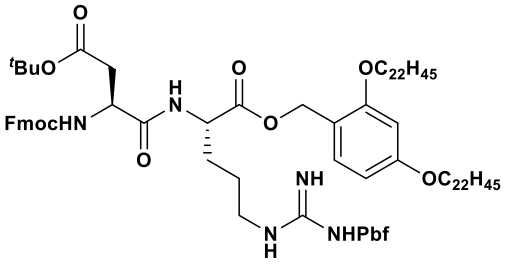

Fmoc-Asp(O^{t}Bu)-Arg(Pbf)-Tag (**3ao**). Following the general procedure, 2,6-lutidine (69.9 μl, 0.60 mmol), Bu_4NClO_4 (136.8 mg, 0.40 mmol), Ph_3P (157.4 mg, 0.60 mmol), Fmoc-Asp(O^{t}Bu)-OH (123.4 mg, 0.30 mmol), and H_2N-Arg(Pbf)-Tag (197.1 mg, 0.20 mmol) were added to Ar-purged MeCN(8.0 mL)-*c*-hex (4.0 mL). The solution was electrolyzed at constant current (0.67 mA/cm^2) under Ar at 50 °C. After 4.8 F/mol of electricity based on H_2N-Arg(Pbf)-Tag was passed, the reaction mixture was extracted with *c*-hex (20 mL x 2), and then the solvent was replaced with MeCN. The resulting precipitants were collected by filtration and washed with MeCN to give the product in 95% yield (296.7 mg) as white solid.

^{1}H NMR ($CDCl_3$, 400 MHz) δ 7.74 (2H, d, J = 7.8 Hz), 7.61-7.52 (2H, m), 7.38 (2H, t, J = 7.3

Hz), 7.32-7.26 (2H, m), 7.13 (1H, d, J = 8.2 Hz), 6.44-6.36 (2H, m), 6.18-5.77 (4H, m), 5.16 (1H, d, J = 11.9 Hz), 5.08 (1H, d, J = 11.9 Hz), 4.64-4.46 (2H, m), 4.38 (2H, d, J = 6.9 Hz), 4.18 (1H, t, J = 6.9 Hz), 3.94-3.85 (4H, m), 3.26-3.04 (2H, m), 2.93-2.81 (3H, m), 2.64 (1H, dd, J = 16.9 Hz, 4.1 Hz), 2.56 (3H, s), 2.50 (3H, s), 2.06 (3H, s), 1.92-1.63 (7H, m), 1.60-1.21 (93H, m), 0.88 (6H, t, J = 6.9 Hz); ^{13}C NMR($CDCl_3$, 100 MHz) δ 171.5, 171.0, 171.0, 161.0, 158.6, 158.4, 156.3, 156.0, 143.8, 143.5, 141.3, 138.4, 133.2, 132.3, 132.0, 131.3, 127.8, 127.1, 125.1, 124.5, 120.0, 117.4, 115.6, 104.6, 99.7, 86.2, 82.2, 68.2, 68.1, 67.4, 63.1, 51.8, 51.4, 47.0, 43.2, 40.6, 37.4, 31.9, 30.2, 29.7, 29.7, 29.5, 29.4, 29.3, 29.1, 28.6, 28.0, 26.1, 26.0, 24.6, 22.7, 19.2, 17.9, 14.1, 12.4; HRMS (ESI-MS) $[M+Na]^+$ calcd for $C_{93}H_{147}N_5O_{12}S$ 1581.0659, found 1581.0657..

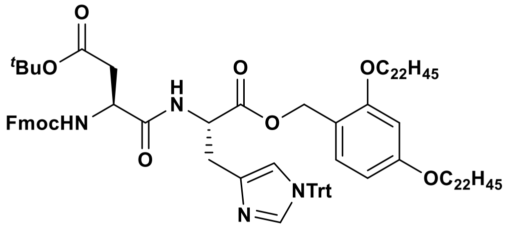

Fmoc-Asp(O^{t}Bu)-His(Trt)-Tag (**3ap**). Following the general procedure, 2,6-lutidine (69.9 μl, 0.60 mmol), Bu_4NClO_4 (136.8 mg, 0.40 mmol), Ph_3P (157.4 mg, 0.60 mmol), Fmoc-Asp(O^{t}Bu)-OH (123.4 mg, 0.30 mmol), and H_2N-His(Trt)-Tag (227.3 mg, 0.20 mmol) were added to Ar-purged MeCN(8.0 mL)-*c*-hex (4.0 mL). The solution was electrolyzed at constant current (0.67 mA/cm^2) under Ar at room temparature. After 4.8 F/mol of electricity based on H_2N-His(Trt)-Tag was passed, the reaction mixture was extracted with *c*-hex (20 mL x 2), and then the solvent was replaced with MeCN. The resulting precipitants were collected by filtration and washed with MeCN to give the product in 97% yield (297.9 mg) as white solid.

^{1}H NMR ($CDCl_3$, 400 MHz) δ 8.12 (0.4H, d, J = 7.3 Hz, minor rotamer), 7.90 (0.6H, d, J = 7.8 Hz, major rotamer), 7.73 (2H, d, J = 7.3 Hz), 7.61-7.49 (2H, m), 7.36 (2H, t, J = 7.3 Hz), 7.31-7.25 (3H, m), 7.25-7.17 (9H, m), 7.08-6.93 (7H, m), 6.56-6.48 (1H, m), 6.48-6.41 (1H, m), 6.37

(1H, d, J = 1.8 Hz), 6.26 (1H, dd, J = 8.2 Hz, 1.8 Hz), 5.09 (1H, d, J = 11.9 Hz), 5.01-4.87 (1H, m), 4.86-4.75 (1H, m), 4.69-4.56 (1H, m), 4.37-4.21 (2H, m), 4.15 (0.4H, t, J = 6.9 Hz, minor rotamer), 4.07 (0.6H, t, J = 6.9 Hz, major rotamer), 3.90-3.79 (4H, m), 3.12-2.95 (2H, m), 2.94-2.58 (2H, m), 1.79-1.64 (4H, m), 1.46-1.21 (85H, m), 0.88 (6H, t, J = 6.9 Hz); ^{13}C NMR ($CDCl_3$, 100 MHz) δ 170.9, 170.8, 170.7, 170.5, 170.4, 170.3, 170.2, 170.2, 170.1, 160.6, 160.5, 158.1, 158.1, 155.9, 155.9, 155.8, 144.0, 144.0, 143.9, 143.9, 143.8, 143.7, 143.7, 142.3, 142.2, 141.3, 138.7, 138.6, 136.5, 136.4, 130.6, 130.6, 129.7, 128.0, 127.7, 127.1, 127.1, 125.3, 125.3, 125.2, 119.9, 119.8, 119.5, 116.3, 116.2, 104.5, 99.6, 81.5, 81.3, 75.2, 75.2, 68.1, 68.0, 67.3, 67.1, 62.4, 62.4, 53.0, 52.9, 51.6, 51.4, 47.1, 37.7, 31.9, 30.0, 29.7, 29.6, 29.4, 29.4, 29.3, 29.1, 28.1, 28.0, 26.1, 26.0, 22.7, 14.1; HRMS (ESI-MS) $[M+Na]^+$ calcd for $C_{99}H_{140}N_4O_9$ 1552.0513, found 1552.0502.

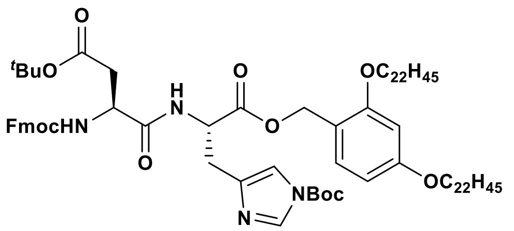

Fmoc-Asp(O^{t}Bu)-His(Boc)-Tag (**3aq**). Following the general procedure, 2,6-lutidine (69.9 μl, 0.60 mmol), Bu_4NClO_4 (136.8 mg, 0.40 mmol), Ph_3P (157.4 mg, 0.60 mmol), Fmoc-Asp(O^{t}Bu)-OH (123.4 mg, 0.30 mmol), and H_2N-His(Boc)-Tag (198.9 mg, 0.20 mmol) were added to Ar-purged MeCN(8.0 mL)-*c*-hex (4.0 mL). The solution was electrolyzed at constant current (0.67 mA/cm^2) under Ar at room temparature. After 4.8 F/mol of electricity based on H_2N-His(Boc)-Tag was passed, the reaction mixture was extracted with *c*-hex (20 mL x 2), and then the solvent was replaced with MeCN. The resulting precipitants were collected by filtration and washed with MeCN to give the product in 99% yield (274.8 mg) as white solid.

^{1}H NMR ($CDCl_3$, 600 MHz) δ 7.88 (1H, s), 7.76 (2H, d, J = 7.6 Hz), 7.67 (1H, d, J = 7.6 Hz), 7.61 (2H, t, J = 6.9 Hz), 7.39 (2H, t, J = 7.6 Hz), 7.30 (2H, t, J = 7.6 Hz), 7.13 (1H, d, J = 8.3 Hz), 7.06 (1H, s), 6.44-6.36 (2H, m), 6.13 (1H, d, J = 8.3 Hz), 5.14 (1H, d, J = 12.4 Hz), 5.11 (1H, d,

J = 12.4 Hz), 4.86-4.81 (1H, m), 4.66-4.58 (1H, m), 4.38 (2H, d, *J* = 6.9 Hz), 4.24 (1H, t, *J* = 6.9 Hz), 3.96-3.88 (4H, m), 3.06 (2H, d, *J* = 5.5 Hz), 2.89 (1H, dd, *J* = 17.2 Hz, 4.8 Hz), 2.66 (1H, dd, *J* = 17.2 Hz, 6.2 Hz), 1.80-1.73 (4H, m), 1.55 (9H, s), 1.48-1.21 (85H, m), 0.88 (6H, t, *J* = 6.9 Hz); ^{13}C NMR ($CDCl_3$, 150 MHz) δ 171.0, 170.7, 170.3, 160.7, 158.3, 155.9, 146.7, 143.9, 143.8, 141.3, 138.4, 136.8, 131.0, 127.7, 127.1, 125.2, 120.0, 116.1, 114.7, 104.5, 99.6, 85.4, 81.5, 68.2, 68.1, 67.3, 62.7, 52.4, 51.2, 47.2, 37.7, 31.9, 29.7, 29.7, 29.6, 29.4, 29.4, 29.3, 29.2, 28.0, 27.8, 26.1, 26.1, 22.7, 14.1; HRMS (ESI-MS) $[M+Na]^+$ calcd for $C_{85}H_{134}N_4O_{11}$ 1409.9941, found 1409.9939.

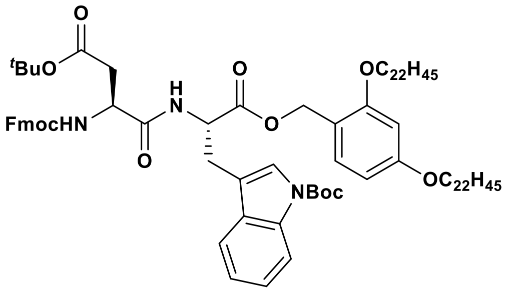

Fmoc-Asp(O^{t}Bu)-Trp(Boc)-Tag (**3ar**). Following the general procedure, 2,6-lutidine (69.9 μl, 0.60 mmol), Bu_4NClO_4 (136.8 mg, 0.40 mmol), Ph_3P (157.4 mg, 0.60 mmol), Fmoc-Asp(O^{t}Bu)-OH (123.4 mg, 0.30 mmol), and H_2N-Trp(Boc)-Tag (208.7 mg, 0.20 mmol) were added to Ar-purged MeCN(8.0 mL)-*c*-hex (4.0 mL). The solution was electrolyzed at constant current (0.67 mA/cm^2) under Ar at room temparature. After 4.8 F/mol of electricity based on H_2N-Trp(Boc)-Tag was passed, the reaction mixture was extracted with *c*-hex (20 mL x 2), and then the solvent was replaced with MeCN. The resulting precipitants were collected by filtration and washed with MeCN to give the product in 96% yield (275.8 mg) as white solid.

^{1}H NMR ($CDCl_3$, 400 MHz) δ 8.10 (1H, d, *J* = 6.4 Hz), 7.75 (2H, d, *J* = 7.3 Hz), 7.60-7.43 (4H, m), 7.38 (2H, t, *J* = 7.3 Hz), 7.32-7.25 (3H, m), 7.24-7.12 (2H, m), 7.02 (1H, d, *J* = 8.2 Hz), 6.45-6.32 (2H, m), 5.91 (1H, d, *J* = 8.2 Hz), 5.11 (1H, d, *J* = 11.9 Hz), 5.06 (1H, d, *J* = 11.9 Hz), 4.95-4.85 (1H, m), 4.59-4.45 (1H, m), 4.44-4.24 (2H, m), 4.18 (1H, t, *J* = 7.3 Hz), 3.97-3.85 (4H, m), 3.23 (2H, d, *J* = 5.5 Hz), 2.88 (1H, dd, *J* = 16.9 Hz, 3.2 Hz), 2.58 (1H, dd, *J* = 16.9 Hz, 6.9 Hz),

1.80-1.70 (4H, m), 1.63 (9H, s), 1.49-1.21 (85H, m), 0.88 (6H, t, J = 6.9 Hz); ^{13}C NMR ($CDCl_3$, 100 MHz) δ 171.3, 171.1, 170.2, 160.9, 158.4, 155.9, 149.5, 143.9, 143.7, 141.3, 131.1, 130.5, 127.7, 127.1, 125.1, 124.5, 122.6, 120.0, 119.0, 115.7, 115.3, 114.8, 104.5, 99.7, 83.5, 81.8, 68.2, 68.1, 67.3, 63.1, 52.9, 51.0, 47.1, 37.5, 31.9, 30.0, 29.7, 29.6, 29.4, 29.4, 29.3, 29.1, 28.2, 28.0, 27.3, 26.1, 26.0, 22.7, 14.1; HRMS (ESI-MS) $[M+Na]^+$ calcd for $C_{90}H_{137}N_3O_{11}$ 1459.0145, found 1459.0162.

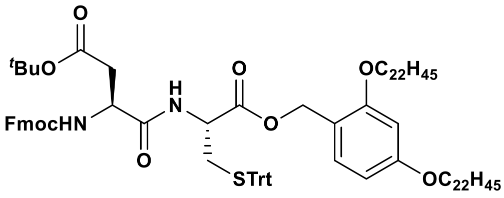

Fmoc-Asp(O^{t}Bu)-Cys(Trt)-Tag (**3as**). Following the general procedure, 2,6-lutidine (69.9 μl, 0.60 mmol), Bu_4NClO_4 (136.8 mg, 0.40 mmol), Ph_3P (157.4 mg, 0.60 mmol), Fmoc-Asp(O^{t}Bu)-OH (123.4 mg, 0.30 mmol), and H_2N-Cys(Trt)-Tag (220.4 mg, 0.20 mmol) were added to Ar-purged MeCN(8.0 mL)-*c*-hex (4.0 mL). The solution was electrolyzed at constant current (0.67 mA/cm^2) under Ar at 40 °C. After 4.8 F/mol of electricity based on H_2N-Cys(Trt)-Tag was passed, the reaction mixture was extracted with *c*-hex (20 mL x 2), and then the solvent was replaced with MeCN. The resulting precipitants were collected by filtration and washed with MeCN to give the product in 99% yield (297.8 mg) as white solid.

^{1}H NMR ($CDCl_3$, 400 MHz) δ 7.75 (2H, d, J = 7.8 Hz), 7.57 (2H, t, J = 7.8 Hz), 7.42-7.25 (10H, m), 7.25-7.07 (11H, m), 6.43-6.34 (2H, m), 5.93 (1H, d, J = 7.8 Hz), 5.21-5.01 (2H, m), 4.60-4.50 (1H, m), 4.50-4.43 (1H, m), 4.42-4.29 (2H, m), 4.20 (1H, t, J = 6.9 Hz), 3.92 (2H, t, J = 6.4 Hz), 3.86 (2H, t, J = 6.4 Hz), 2.80 (1H, dd, J = 17.4 Hz, 3.7 Hz), 2.71-2.47 (3H, m), 1.81-1.63 (4H, m), 1.50-1.20 (85H, m), 0.88 (6H, t, J = 6.9 Hz); ^{13}C NMR ($CDCl_3$, 100 MHz) δ 171.0, 170.2, 169.8, 160.8, 158.3, 155.8, 144.3, 143.9, 143.7, 141.3, 141.3, 131.0, 129.5, 127.9, 127.7, 127.1, 126.8, 125.1, 120.0, 116.0, 104.6, 99.6, 81.8, 68.1, 68.1, 67.3, 66.8, 62.8, 51.7, 50.7, 47.1, 37.8, 33.7, 31.9, 29.7, 29.6, 29.4, 29.4, 29.3, 29.1, 28.0, 26.1, 26.0, 22.7, 14.1; HRMS (ESI-MS) $[M+Na]^+$ calcd for $C_{96}H_{138}N_2O_9S$ 1518.0015, found 1518.0044.

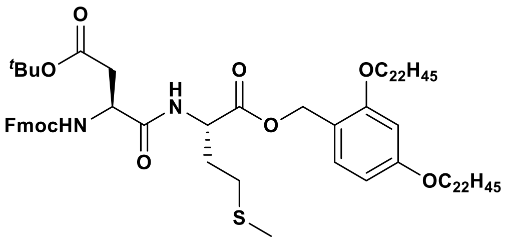

Fmoc-Asp(O^{t}Bu)-Met-Tag (**3at**). Following the general procedure, 2,6-lutidine (69.9 μl, 0.60 mmol), Bu_4NClO_4 (136.8 mg, 0.40 mmol), Ph_3P (131.1 mg, 0.50 mmol), Fmoc-Asp(O^{t}Bu)-OH (123.4 mg, 0.30 mmol), and H_2N-Met-Tag (177.7 mg, 0.20 mmol) were added to Ar-purged MeCN(8.0 mL)-*c*-hex (4.0 mL). The solution was electrolyzed at constant current (0.67 mA/cm^2) under Ar at 50 °C. After 4.8 F/mol of electricity based on H_2N-Met-Tag was passed, the reaction mixture was extracted with *c*-hex (20 mL x 2), and then the solvent was replaced with MeCN. The resulting precipitants were collected by filtration and washed with MeCN to give the product in 99% yield (253.2 mg) as white solid.

^{1}H NMR ($CDCl_3$, 600 MHz) δ 7.76 (2H, d, *J* = 7.6 Hz), 7.64-7.51 (2H, m), 7.40 (2H, t, *J* = 7.6 Hz), 7.32 (2H, t, *J* = 7.6 Hz), 7.24-7.11 (2H, m), 6.45-6.35 (2H, m), 5.98 (1H, d, *J* = 8.3 Hz), 5.20 (1H, d, *J* = 11.7 Hz), 5.09 (1H, d, *J* = 11.7 Hz), 4.67 (1H, dd, *J* = 12.4 Hz, 7.6 Hz), 4.61-4.50 (1H, m), 4.41 (2H, d, *J* = 6.9 Hz), 4.23 (1H, t, *J* = 6.9 Hz), 3.96-3.85 (4H, m), 2.95 (1H, dd, *J* = 17.2 Hz, 3.4 Hz), 2.60 (1H, dd, *J* = 17.5 Hz, 6.2 Hz), 2.52-2.38 (2H, m), 2.20-2.09 (1H, m), 2.05-1.91 (4H, m), 1.81-1.70 (4H, m), 1.52-1.21 (85H, m), 0.88 (6H, t, *J* = 6.9 Hz); ^{13}C NMR ($CDCl_3$, 150 MHz) δ 171.4, 171.4, 170.3, 161.0, 158.5, 156.0, 143.8, 143.7, 141.3, 131.4, 127.8, 127.1, 125.1, 120.0, 115.8, 104.5, 99.7, 82.0, 68.2, 68.1, 67.3, 63.1, 52.0, 51.0, 47.1, 37.5, 31.9, 29.7, 29.7, 29.6, 29.6, 29.4, 29.4, 29.3, 29.2, 28.0, 26.1, 26.0, 22.7, 15.3, 14.1; HRMS (ESI-MS) $[M+Na]^+$ calcd for $C_{79}H_{128}N_2O_9S$ 1303.9233, found 1303.9235.

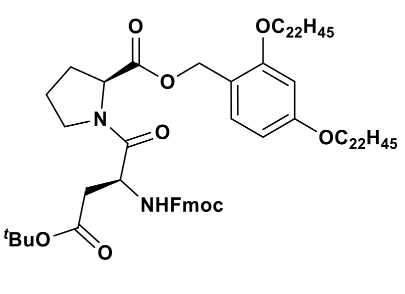

Fmoc-Pro-Asp(O^{t}Bu)-Tag (**3au**). Following the general procedure, 2,6-lutidine (69.9 μl, 0.60 mmol), Bu_4NClO_4 (136.8 mg, 0.40 mmol), Ph_3P (131.1 mg, 0.50 mmol), Fmoc-Asp(O^{t}Bu)-OH (205.7 mg, 0.50 mmol), and H_2N-Pro-Tag (168.1 mg, 0.20 mmol) were added to Ar-purged MeCN(8.0 mL)-*c*-hex (4.0 mL). The solution was electrolyzed at constant current (0.67 mA/cm^2) under Ar at room temperature. After 6.0 F/mol of electricity based on H_2N-Pro-Tag was passed, the reaction mixture was extracted with *c*-hex (20 mL x 2), and then the solvent was replaced with MeCN. The resulting precipitants were collected by filtration and washed with MeCN to give the product in 99% yield (249.5 mg) as white solid.

^{1}H NMR ($CDCl_3$, 400 MHz) δ 7.79-7.71 (2H, m), 7.62-7.51 (2H, m), 7.43-7.34 (2H, m), 7.34-7.26 (2H, m), 7.22-7.15 (0.83H, m, major rotamer), 7.15-7.08 (0.17H, m, minor rotamer), 6.46-6.33 (2H, m), 5.68 (0.83H, d, *J* = 8.9 Hz, major rotamer), 5.53 (0.17H, d, *J* = 9.2 Hz, minor rotamer), 5.21 (0.87H, d, *J* = 11.9 Hz, major rotamer), 5.15 (0.13H, d, *J* = 11.9 Hz, minor rotamer), 5.04 (0.87H, d, *J* = 11.9 Hz, major rotamer), 4.98 (0.13H, d, *J* = 11.9 Hz, minor rotamer), 4.94-4.85 (0.72H, m, major rotamer), 4.85-4.79 (0.12H, m, minor rotamer), 4.75-4.65 (0.13H, m, minor rotamer), 4.64-4.47 (1H, m), 4.44-4.25 (2H, m), 4.25-4.11 (1H, m), 3.98-3.83 (4H , m), 3.80-3.62 (2H, m), 2.77 (0.14H, dd, *J* = 16.0 Hz, 7.8 Hz, minor rotamer), 2.64 (0.86H, dd, *J* = 15.6 Hz, 4.6 Hz, major rotamer), 2.48 (1H, dd, *J* = 15.6 Hz, 8.2 Hz), 2.24-2.09 (1H, m), 2.08-1.92 (2H, m), 1.83-1.71 (4H, m), 1.49-1.21 (86H, m), 0.88 (6H, t, *J* = 6.9 Hz); ^{13}C NMR ($CDCl_3$, 100 MHz) δ 171.6, 169.5, 169.5, 160.8, 158.3, 155.8, 143.8, 143.8, 141.3, 131.1, 127.7, 127.1, 125.2, 120.0, 116.3, 104.6, 99.7, 81.4, 68.1, 67.2, 62.5, 59.1, 49.8, 47.1, 46.9, 38.6, 31.9, 29.7, 29.6, 29.4, 29.4, 29.4, 29.3, 29.2, 29.0, 28.0, 26.1, 24.7, 22.7, 14.1; HRMS (ESI-MS) [M+Na]$^+$ calcd for $C_{79}H_{126}N_2O_9$ 1269.9356, found 1296.9345.

2.5 Synthesis of Leuprorelin

(1) Preparation of the Hydrophobic Benzyl Amine HCl salt

To a solution of the hydrophobic benzaldehyde (1.51 g, 2.0 mmol) in THF (45 mL)

ethylamine hydrochloride (815.4 mg, 10.0 mmol) and DMF (14 mL), DIPEA 3.4 mL, $MgSO_4$ (4.00 g), acetic acid (1.50 mL), and $NaBH(OAc)_3$ (211.9 mg x 4 , 8.0 mmol) were added. The resulting reaction mixture was stirred at room temperature for 1.5 h followed by quench of remaining $NaBH(OAc)_3$ by 0.1 N HCl aq. The resulting reaction mixture was filtered and extracted with c-hex, and organic phase was washed with 0.1 HCl aq. and brine, dried over $MgSO_4$ and concentrated in vacuo. To a crude product, MeCN was added to give precipitants. The precipitants were collected by filtration and purified by column chromatography ($CHCl_3$: MeOH =50:1) to give the product as a white solid (1.29 g, 80% yield)

(2) Synthesis of Fmoc-Pro-NEtTag

To a solution of hydrophobic benzyl amine (328.3 mg, 0.40 mmol) in THF (8.0 mL), Fmoc-Pro-OH•H_2O (202.4 mg, 0.60 mmol), DIPEA (224 μL, 1.32 mmol), and COMU (257.0 mg, 0.60 mmol) were added. The mixture was stirred for 20 min followed by solvent replacement with MeCN to give the product as a precipitate. Precipitate was collected by filtration and washed with MeCN to give the products quantitatively as light pink solid.

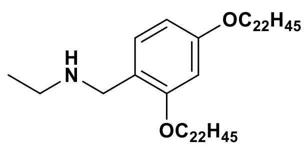

Hydrophobic benzyl amine

^{1}H NMR ($CDCl_3$, 600 MHz) δ 7.09 (1H, d, J = 7.6 Hz), 6.44-6.38 (2H, m), 3.96-3.91 (4H, m), 3.72 (2H, s), 2.62 (2H, q, J = 6.9 Hz), 1.82-1.73 (4H, m), 1.64 (1H, broad s), 1.49-1.22 (76H, m), 1.11 (3H, t, J = 6.9 Hz), 0.88 (6H, t, J = 6.9Hz); ^{13}C NMR ($CDCl_3$, 150 MHz) δ 159.5, 158.1,

130.4, 120.9, 104.2, 99.7, 68.1, 67.8, 49.0, 43.2, 31.9, 29.7, 29.6, 29.6, 29.4, 29.4, 29.3, 29.3, 26.2, 26.1, 22.7, 15.3, 14.1.

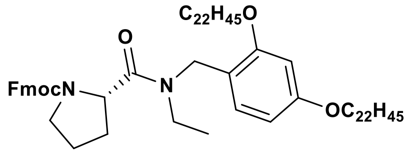

Fmoc-Pro-NEtTag

^{1}H NMR ($CDCl_3$, 600 MHz) δ 7.77-7.71 (2H, m), 7.68-7.50 (2H, m), 7.41-7.33 (2H, m), 7.33-7.23 (2H, m), 7.21-7.09 (0.8H, m), 6.89-6.84 (0.2H, m), 6.51-6.25 (1.8H, m), 5.91-5.87 (0.2H, m), 4.78-4.56 (2H, m), 4.49-4.23 (4H, m), 4.12-3.54 (7H, m), 3.51-3.17 (1H, m), 2.30-2.01 (2H, m), 1.99-1.83 (2H, m), 1.83-1.59 (4H, m), 1.47-1.21 (76H, m), 1.16-1.04 (3H, m), 0.88 (6H, t, J = 6.9 Hz); ^{13}C NMR ($CDCl_3$, 150 MHz) δ 172.5, 172.4, 172.3, 172.0, 159.8, 159.7, 159.5, 159.5, 157.9, 157.3, 157.1, 154.9, 154.9, 154.6, 154.4, 144.6, 144.5, 144.4, 144.3, 144.0, 143.9, 141.3, 141.3, 141.2, 141.2, 141.1, 130.5, 130.0, 127.9, 127.7, 127.6, 127.6, 127.4, 127.1, 127.1, 127.0, 127.0, 125.7, 125.4, 125.2, 125.1, 119.9, 119.8, 119.7, 118.1, 117.0, 116.5, 104.9, 104.7, 104.4, 104.0, 99.6, 99.4, 99.3, 68.1, 68.0, 68.0, 67.9, 67.7, 67.4, 67.3, 67.1, 66.7, 57.2, 57.1, 56.9, 56.6, 54.8, 47.4, 47.4, 47.4, 47.3, 47.3, 47.2, 47.0, 46.9, 45.0, 44.7, 42.3, 41.9, 41.6, 41.5, 41.4, 41.2, 38.4, 31.9, 31.4, 31.3, 30.3, 30.3, 29.7, 29.7, 29.6, 29.6, 29.4, 29.4, 29.3, 29.2, 26.2, 26.1, 26.0, 26.0, 24.5, 24.4, 23.4, 23.3, 22.7, 14.1, 14.1, 14.1, 14.0, 12.7, 12.6.

(3) General Procedure for Electrochemical Peptide Elongation*

MeCN (8.0 mL) and *c*-Hex (4.0 mL) were purged with Ar gas for 2 min. To a purged solvent, 2,6-lutidine (69.9 μL, 0.6 mmol), Bu_4NClO_4 (0.5 mmol), Ph_3P (157.4 mg, 0.6 mmol), Fmoc-protected amino acid (0.4 mmol), and *N*-deprotected tagged amino acid (0.2 mmol) were added. The solution was electrolyzed at constant current (0.67 mA/cm^2) under Ar at 40 °C. After 4.8 F/mol of electricity based on N-deprotected tagged amino

acid was passed, the reaction mixture was extracted with *c*-hex (20 mL x 2), and then the solvent was replaced with MeCN. The resulting precipitants were collected by filtration and washed with MeCN to give the products.

* When condensation with (1) Fmoc-Arg(Pbf)-OH, (2) Fmoc-Ser(OtBu)-OH, and (3) pGlu, following conditions were applied.

(1) Fmoc-Arg(Pbf)-OH 3.5 eq., Ph_3P 5.0 eq., 6.0 F/mol, 60 ºC.

(2) Fmoc-Ser(O*ᵗ*Bu)-OH 3.0 eq., Ph_3P 4.0 eq., 6.0 F/mol, 60 ºC.

(3) pGlu 3.0 eq., Ph3P 4.0 eq., 6.0 F/mol, 60 ºC.

(4) Fmoc-deprotection of Peptides

Following the **2.3**, Fmoc was deprotected and products were obtained.

(5) Global deprotection of the protected leuprorelin (5)

To a solution of 2.5% triisopropylsilane and 2.5% H_2O in TFA, the protected form of leuprorelin (**5**) (20.0 mg, 0.0080 mmol) was added. The resulting reaction mixture was stirred at room temperature for 2h, followed by filtration using celite cake. Filtrate was concentrated in vacuo and DIPE (30 mL) was added. The resulting precipitate was recovered by cooling centrifugation and washed with DIPE (30 mL x 3). Lyophilization gave leuprorelin (**6**) quantitatively.

Leuprorelin (**6**)

White solid, HRMS (ESI-MS) [M+Na] calcd $C_{59}H_{84}O_{12}N_{16}$ for 1231.6347, found 1231.6350.

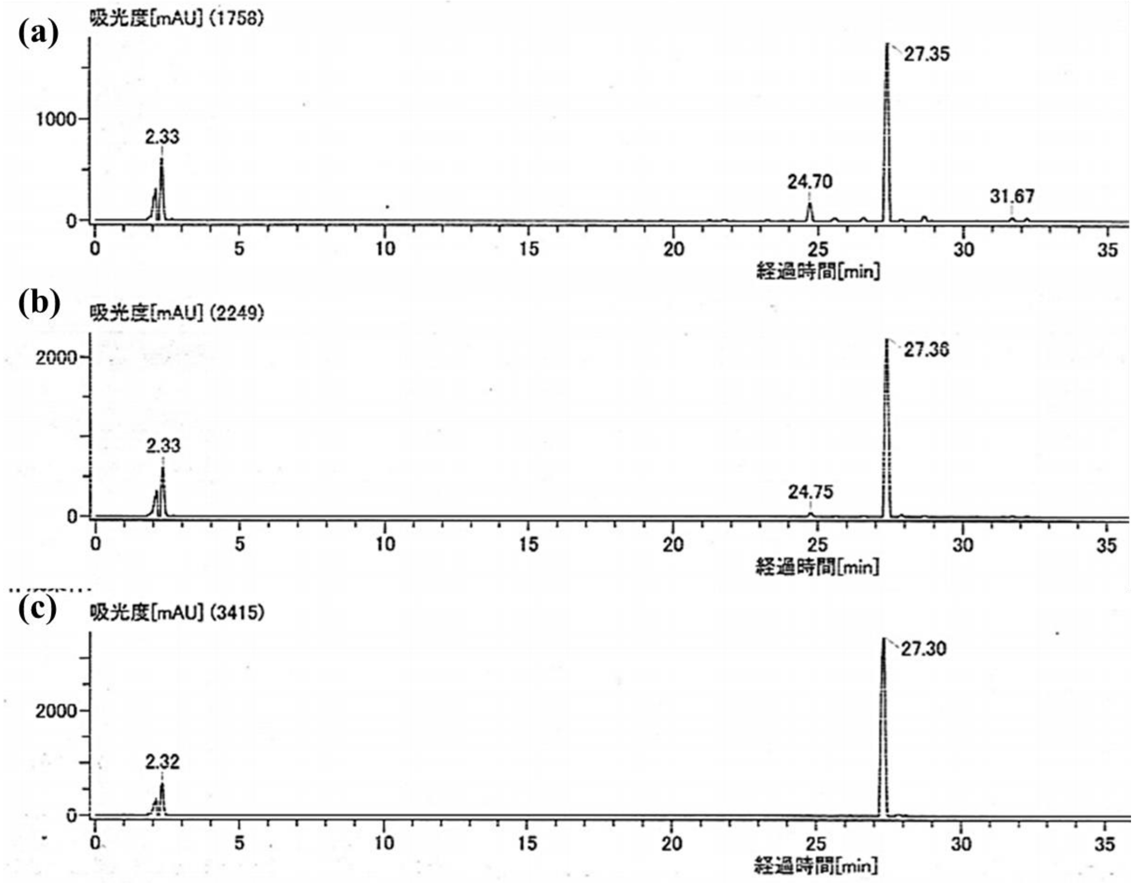

Figure S3. HPLC UV/Vis absorption spectrum (210-220 nm) of (a) synthesized, (b) synthesized and standard, and (c) standard sample of leuprorelin (**6**). C18 column was used a linear gradient of 15-42.5% acetonitrile in water, containing 0.1% trifluoroacetic acid.

3. ^{1}H and ^{13}C NMR Spectra of Compounds

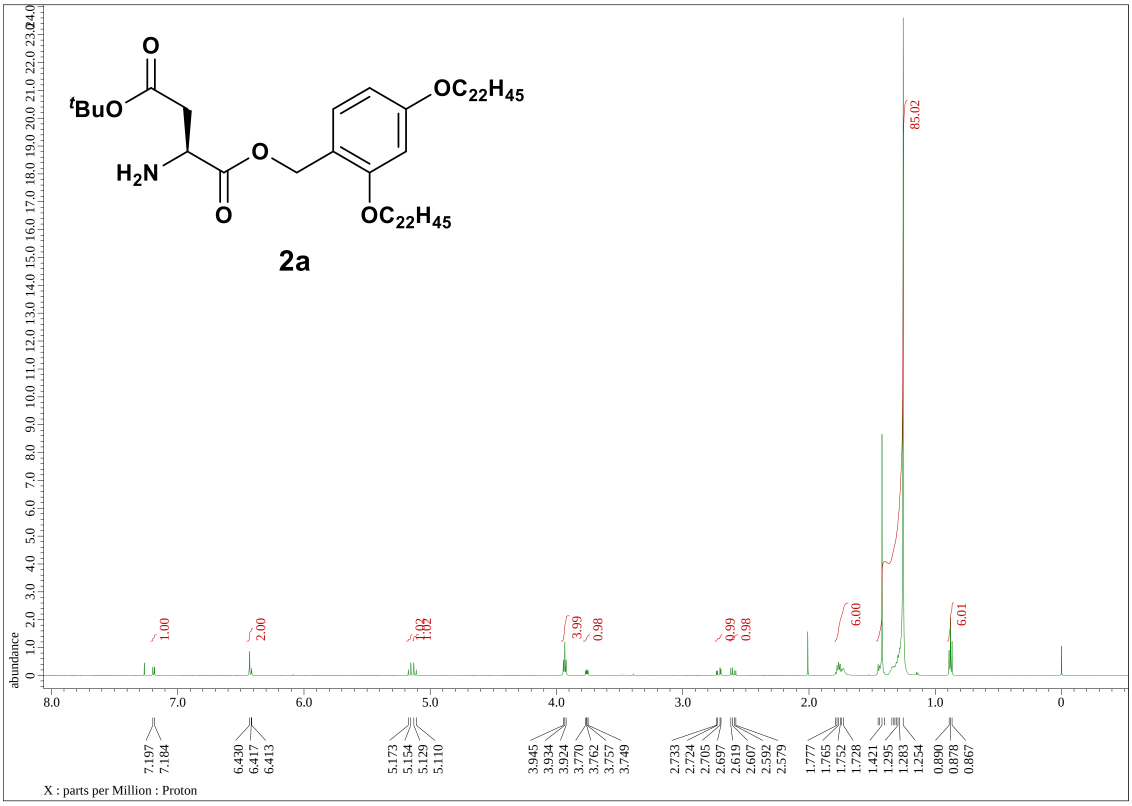

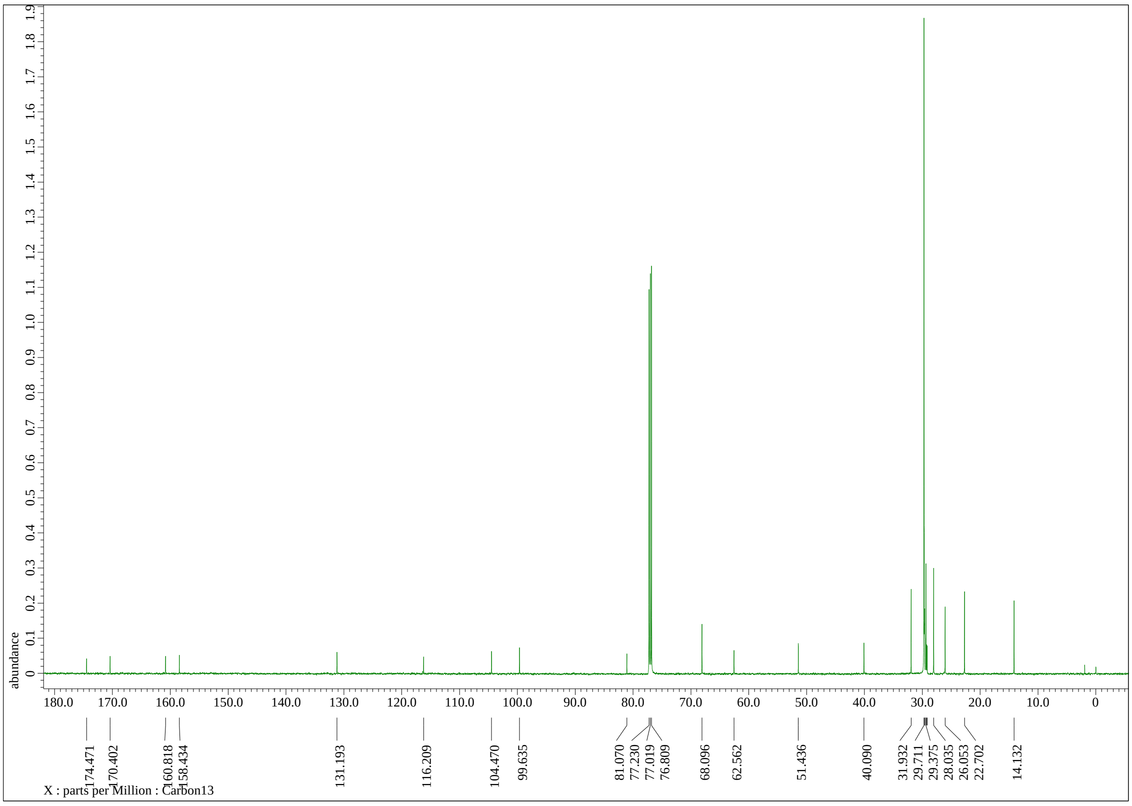

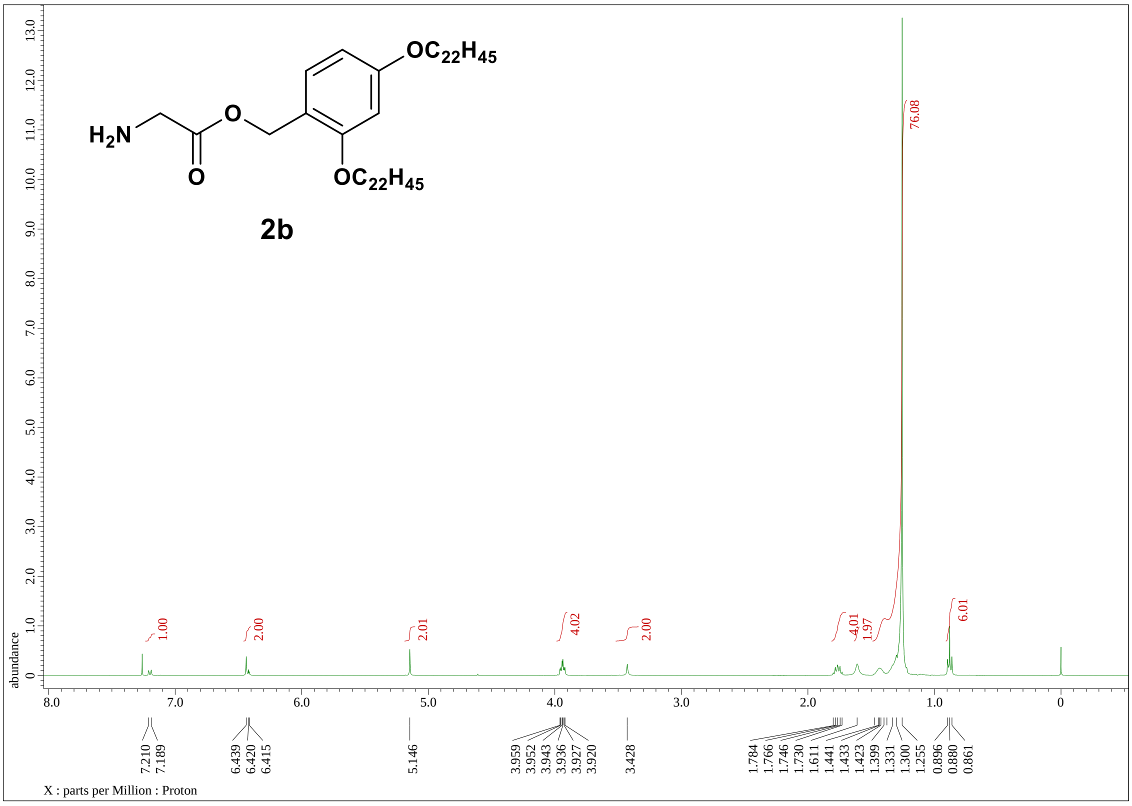

2b
abundance
X : parts per Million : Proton
7.210
7.189
6.439
6.420
6.415
5.146
3.959
3.952
3.943
3.936
3.927
3.920
3.428
1.784
1.766
1.746
1.730
1.611
1.441
1.433
1.423
1.399
1.331
1.300
1.255
0.896
0.880
0.861
1.00
2.00
2.01
4.02
2.00
4.01
1.97
76.08
6.01

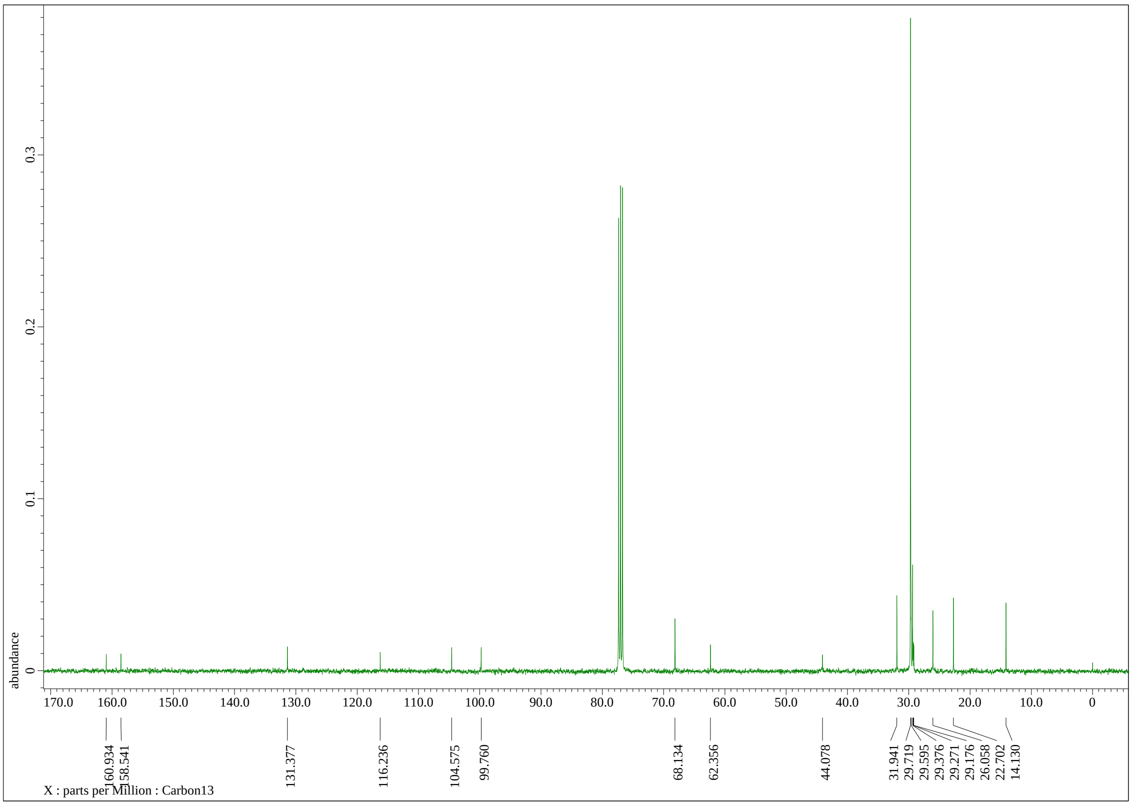

abundance
X : parts per Million : Carbon13
160.934
158.541
131.377
116.236
104.575
99.760
68.134
62.356
44.078
31.941
29.719
29.595
29.376
29.271
29.176
26.058
22.702
14.130

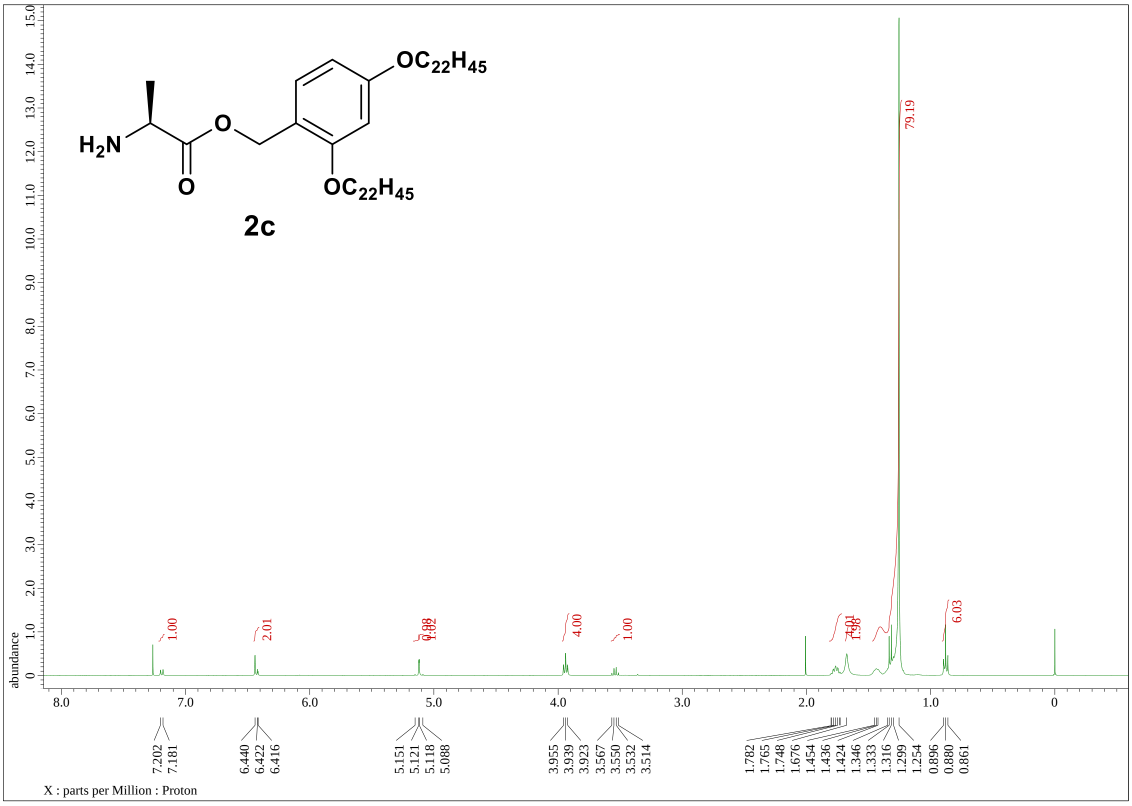

H2N
O
O
OC22H45
OC22H45
2c
abundance
79.19
1.00
2.01
0.98
1.02
4.00
1.00
4.01
1.98
6.03
7.202
7.181
6.440
6.422
6.416
5.151
5.121
5.118
5.088
3.955
3.939
3.923
3.567
3.550
3.532
3.514
1.782
1.765
1.748
1.676
1.454
1.436
1.424
1.346
1.333
1.316
1.299
1.254
0.896
0.880
0.861
X : parts per Million : Proton

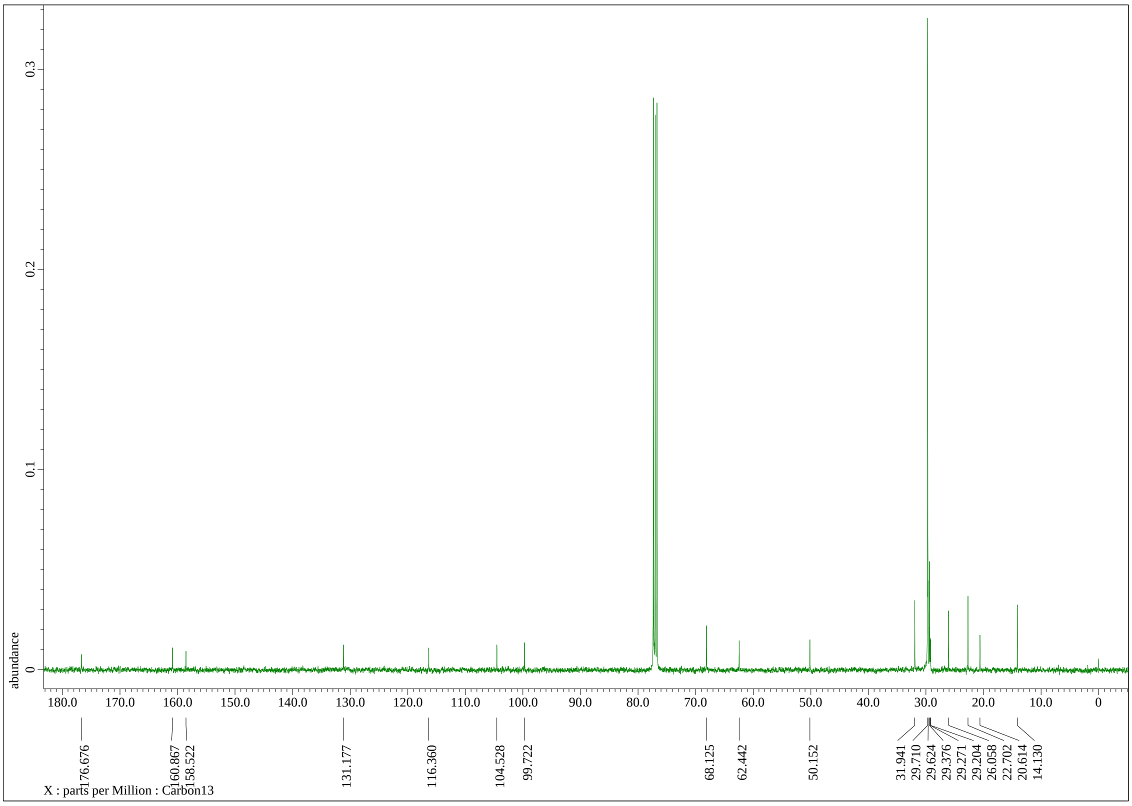

abundance
176.676
160.867
158.522
131.177
116.360
104.528
99.722
68.125
62.442
50.152
31.941
29.710
29.624
29.376
29.271
29.204
26.058
22.702
20.614
14.130
X : parts per Million : Carbon13

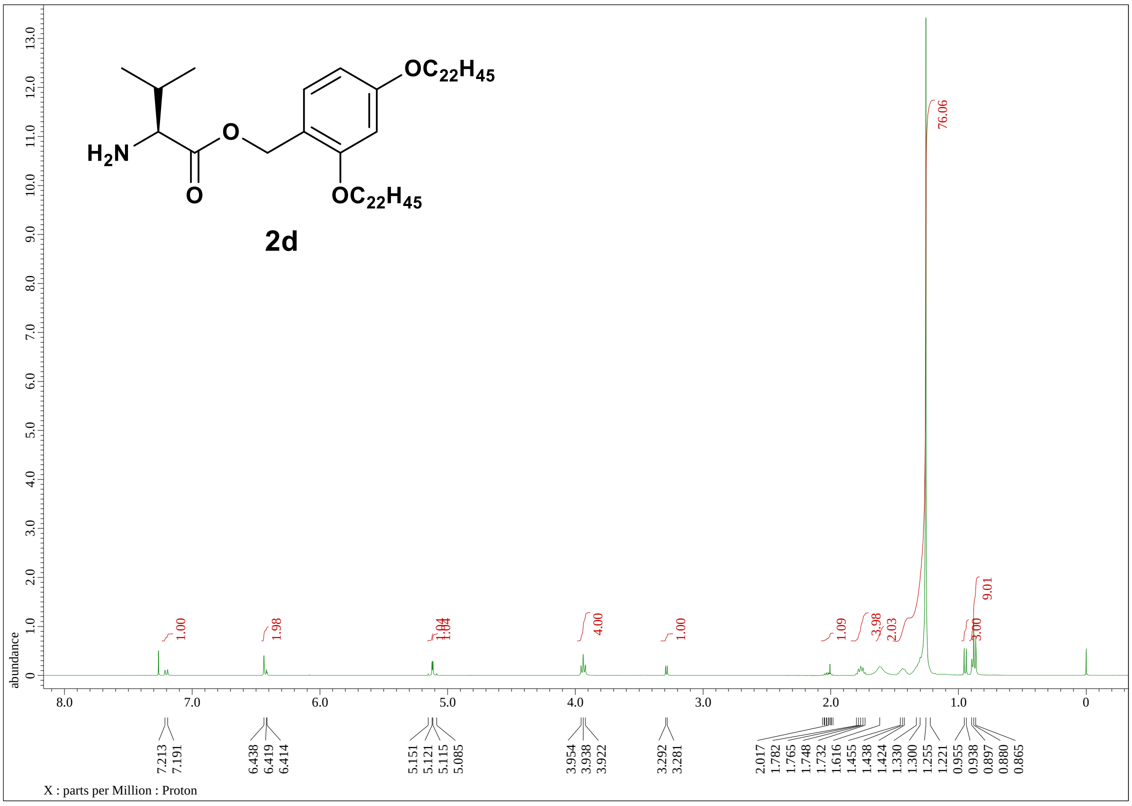
OC22H45
H2N
O
O
OC22H45
2d
76.06
9.01
1.00
1.98
1.04
1.04
4.00
1.00
1.09
3.98
2.03
3.00
7.213
7.191
6.438
6.419
6.414
5.151
5.121
5.115
5.085
3.954
3.938
3.922
3.292
3.281
2.017
1.782
1.765
1.748
1.732
1.616
1.455
1.438
1.424
1.330
1.300
1.255
1.221
0.955
0.938
0.897
0.880
0.865
abundance
X : parts per Million : Proton

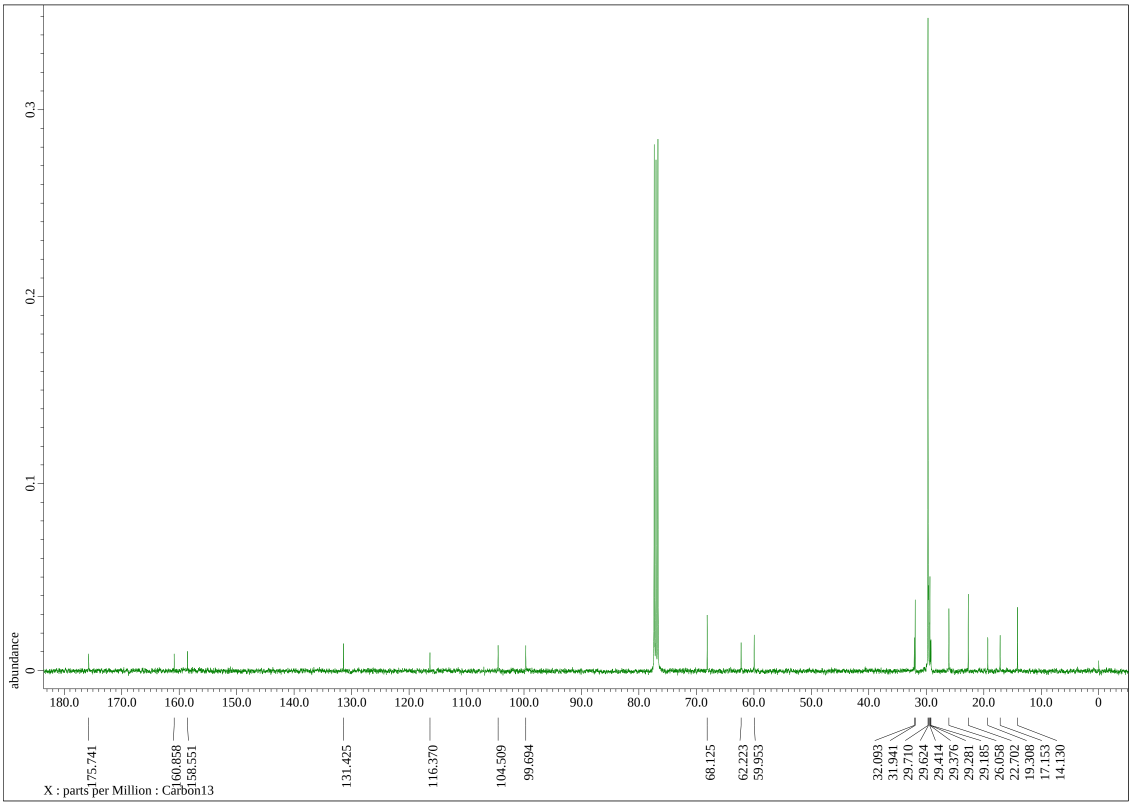
175.741
160.858
158.551
131.425
116.370
104.509
99.694
68.125
62.223
59.953
32.093
31.941
29.710
29.624
29.414
29.376
29.281
29.185
26.058
22.702
19.308
17.153
14.130
abundance
X : parts per Million : Carbon13

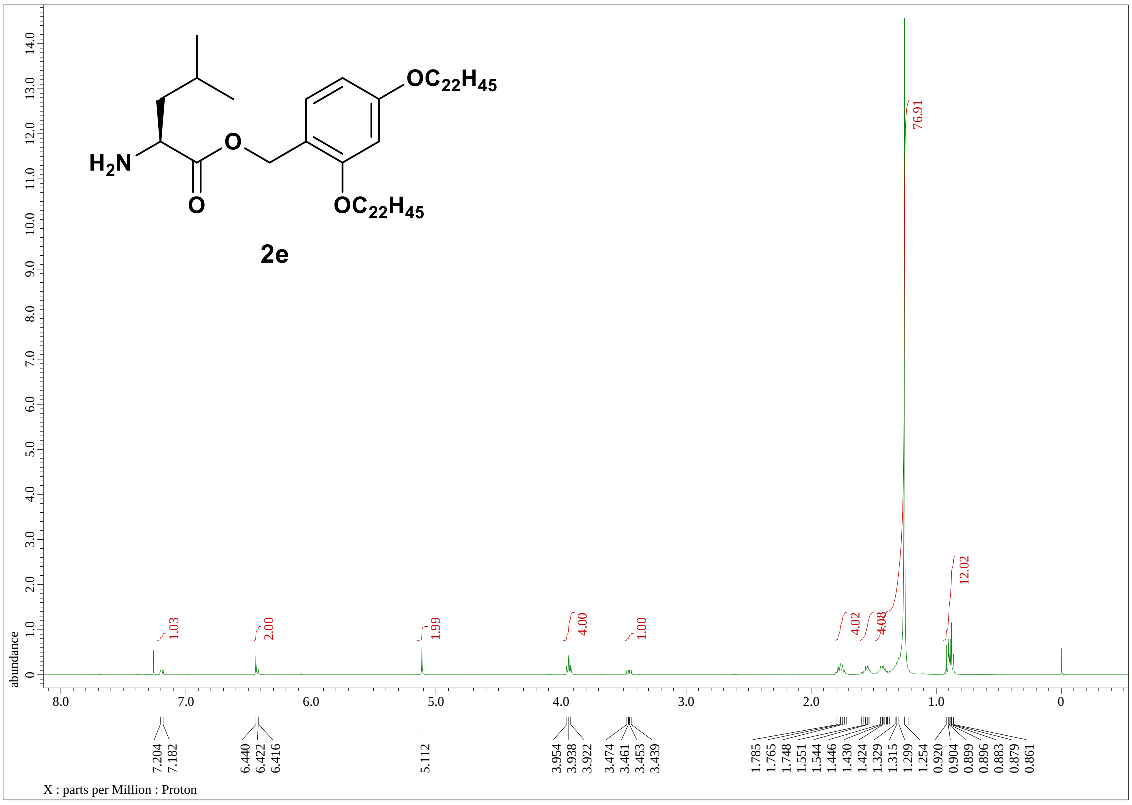
OC22H45
H2N
O
O
OC22H45
2e
1.03
2.00
1.99
4.00
1.00
4.02
4.08
76.91
12.02
7.204
7.182
6.440
6.422
6.416
5.112
3.954
3.938
3.922
3.474
3.461
3.453
3.439
1.785
1.765
1.748
1.551
1.544
1.446
1.430
1.424
1.329
1.315
1.299
1.254
0.920
0.904
0.899
0.896
0.883
0.879
0.861
abundance
X : parts per Million : Proton

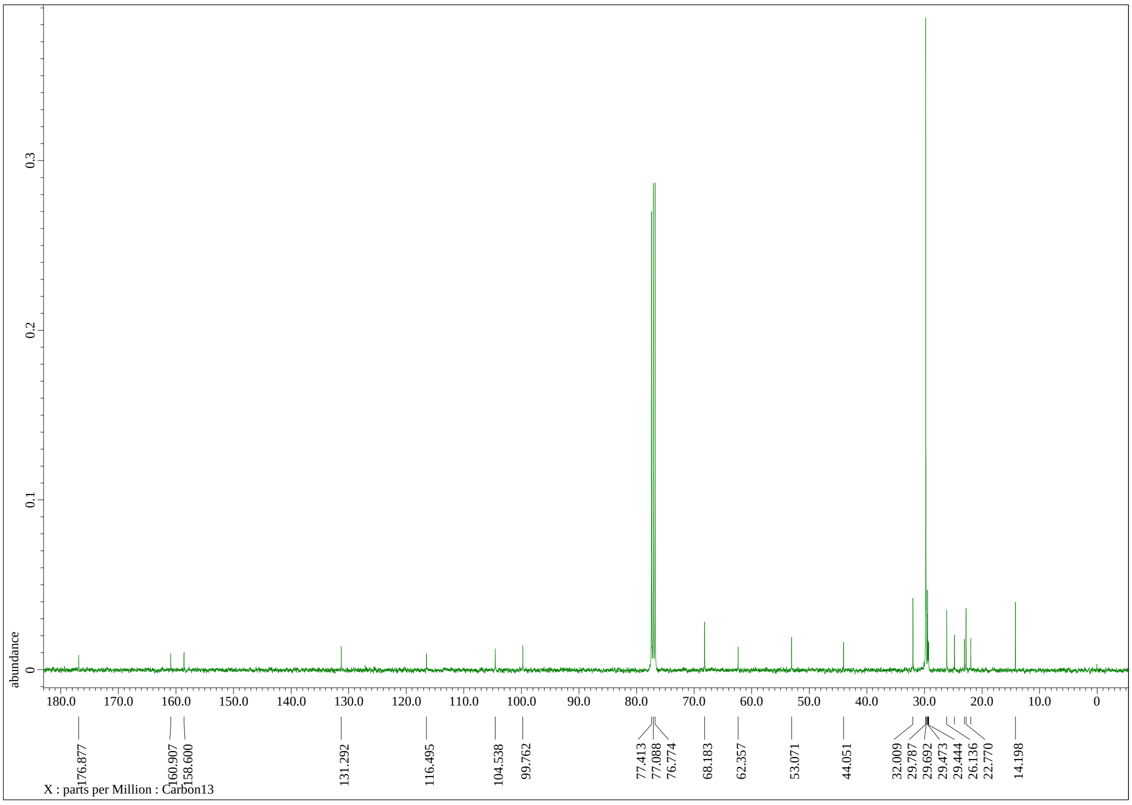
176.877
160.907
158.600
131.292
116.495
104.538
99.762
77.413
77.088
76.774
68.183
62.357
53.071
44.051
32.009
29.787
29.692
29.473
29.444
26.136
22.770
14.198
abundance
X : parts per Million : Carbon13

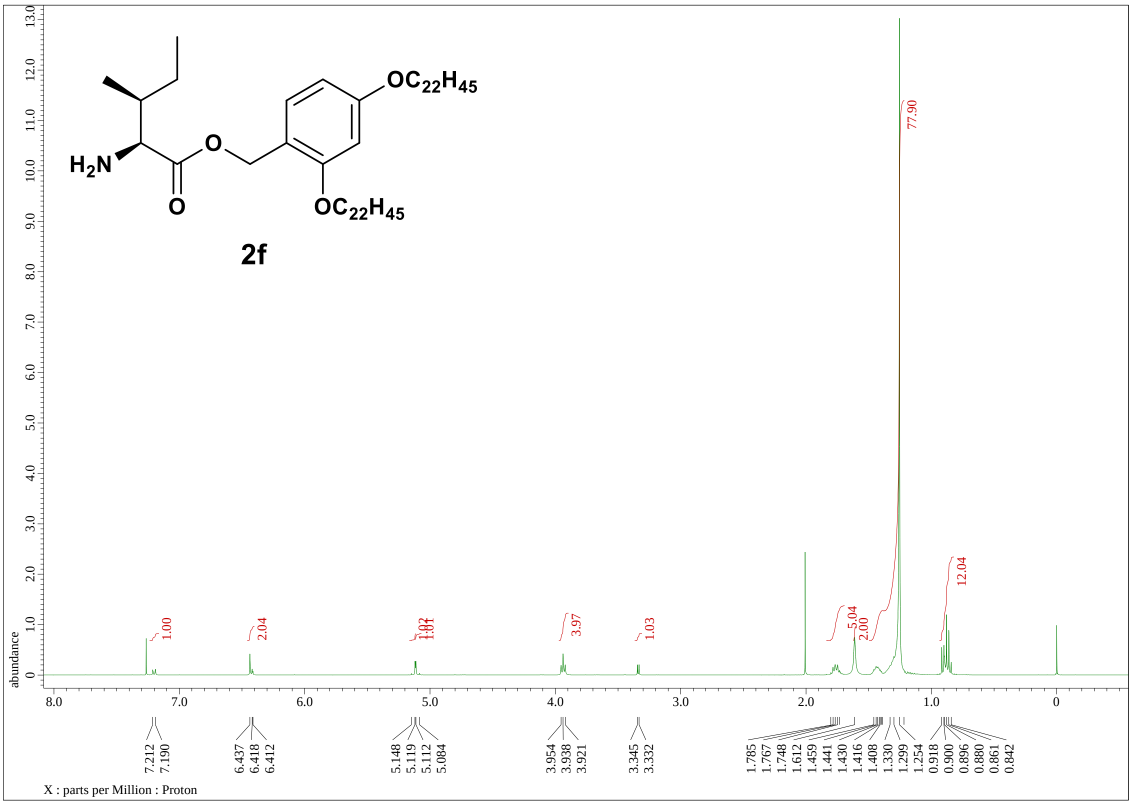

OC22H45
H2N
O
O
OC22H45
2f
77.90
12.04
1.00
2.04
1.02
1.01
3.97
1.03
5.04
2.00
abundance
7.212
7.190
6.437
6.418
6.412
5.148
5.119
5.112
5.084
3.954
3.938
3.921
3.345
3.332
1.785
1.767
1.748
1.612
1.459
1.441
1.430
1.416
1.408
1.330
1.299
1.254
0.918
0.900
0.896
0.880
0.861
0.842
X : parts per Million : Proton

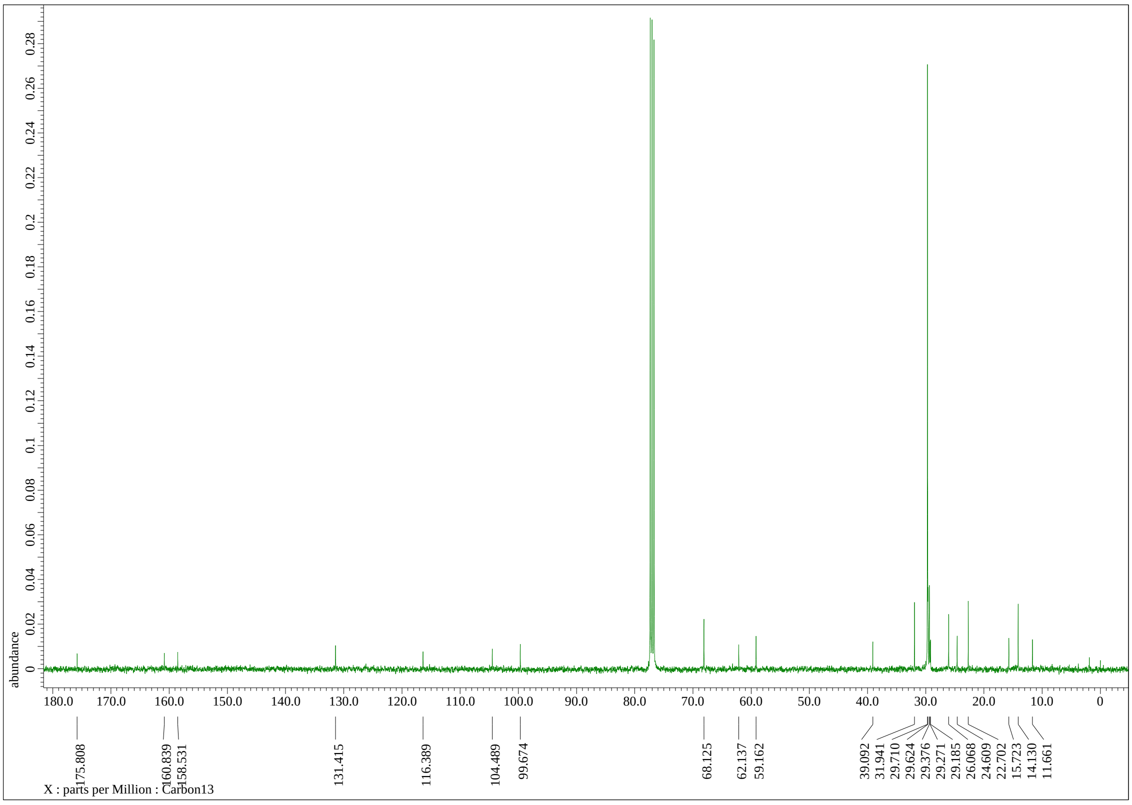

abundance
175.808
160.839
158.531
131.415
116.389
104.489
99.674
68.125
62.137
59.162
39.092
31.941
29.710
29.624
29.376
29.271
29.185
26.068
24.609
22.702
15.723
14.130
11.661
X : parts per Million : Carbon13

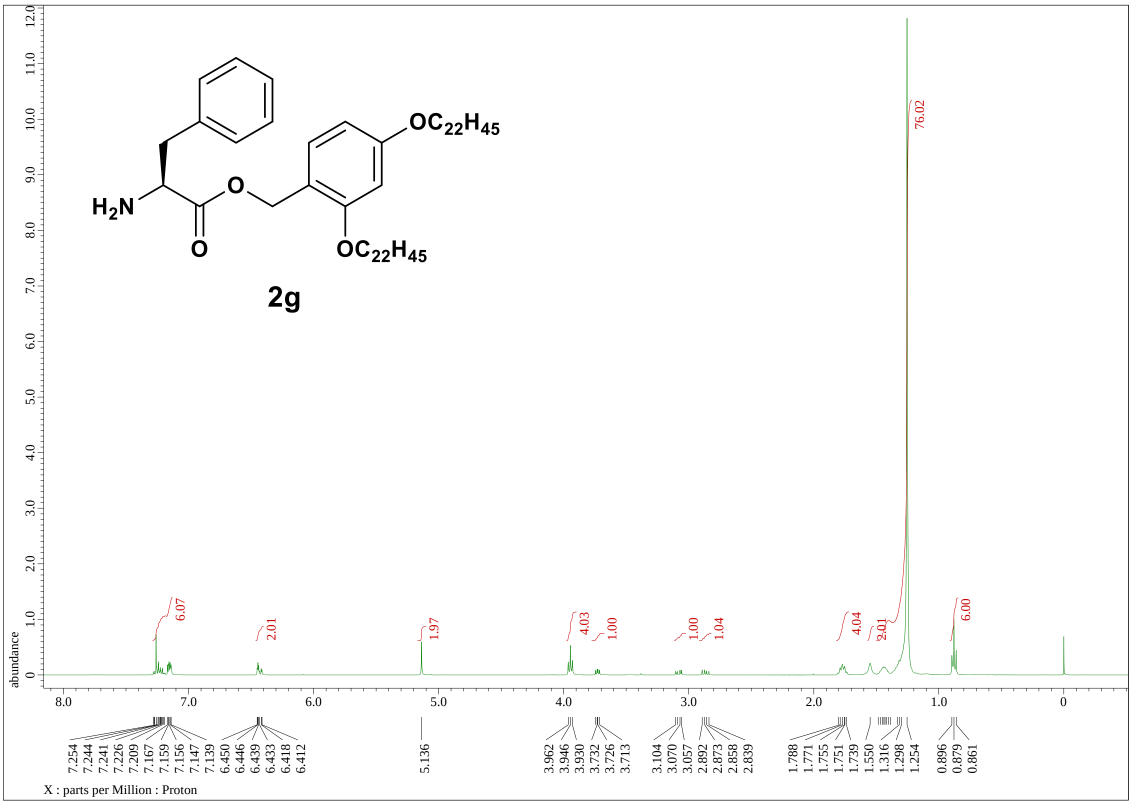
OC22H45
H2N
O
O
OC22H45
2g
abundance
6.07
2.01
1.97
4.03
1.00
1.00
1.04
4.04
2.01
76.02
6.00
7.254 7.244 7.241 7.226 7.209 7.167 7.159 7.156 7.147 7.139 6.450 6.446 6.439 6.433 6.418 6.412
5.136
3.962 3.946 3.930 3.732 3.726 3.713 3.104 3.070 3.057 2.892 2.873 2.858 2.839
1.788 1.771 1.755 1.751 1.739 1.550 1.316 1.298 1.254 0.896 0.879 0.861
X : parts per Million : Proton

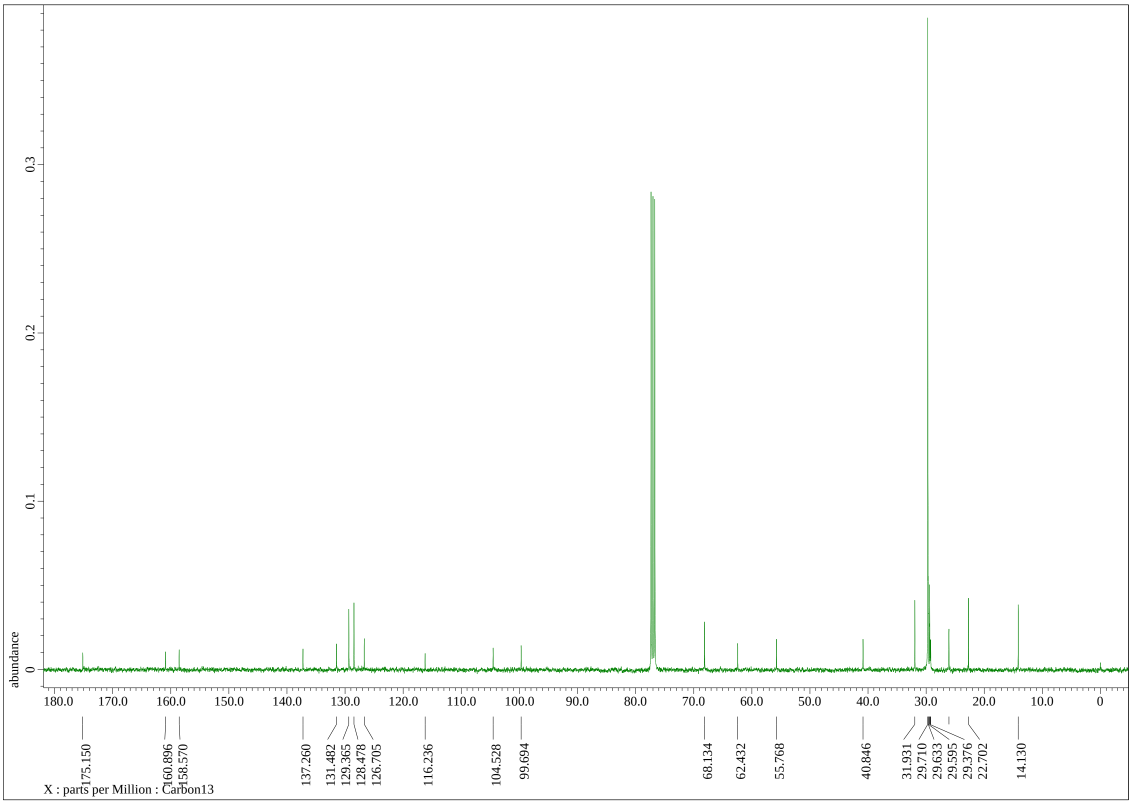
abundance
175.150
160.896 158.570
137.260
131.482 129.365 128.478 126.705
116.236
104.528
99.694
68.134
62.432
55.768
40.846
31.931 29.710 29.633 29.595 29.376 22.702
14.130
X : parts per Million : Carbon13

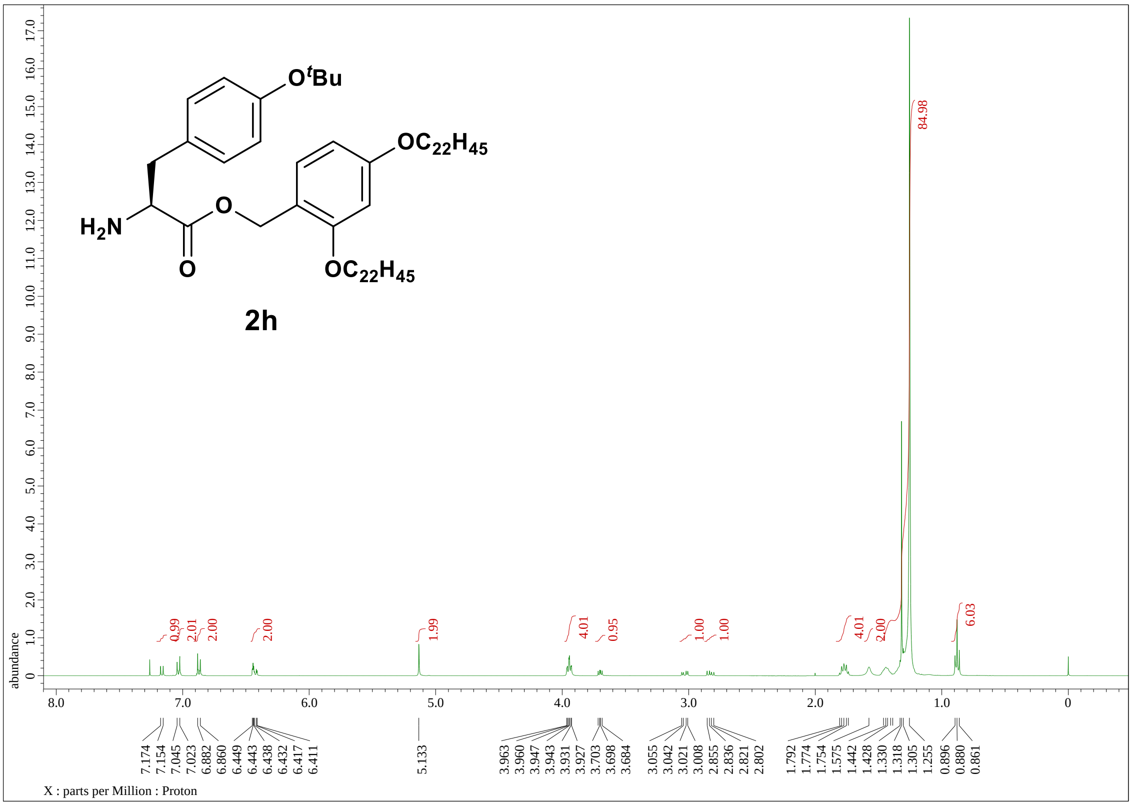

O^{t}Bu
OC22H45
H2N
O
O
OC22H45
2h
abundance
X : parts per Million : Proton

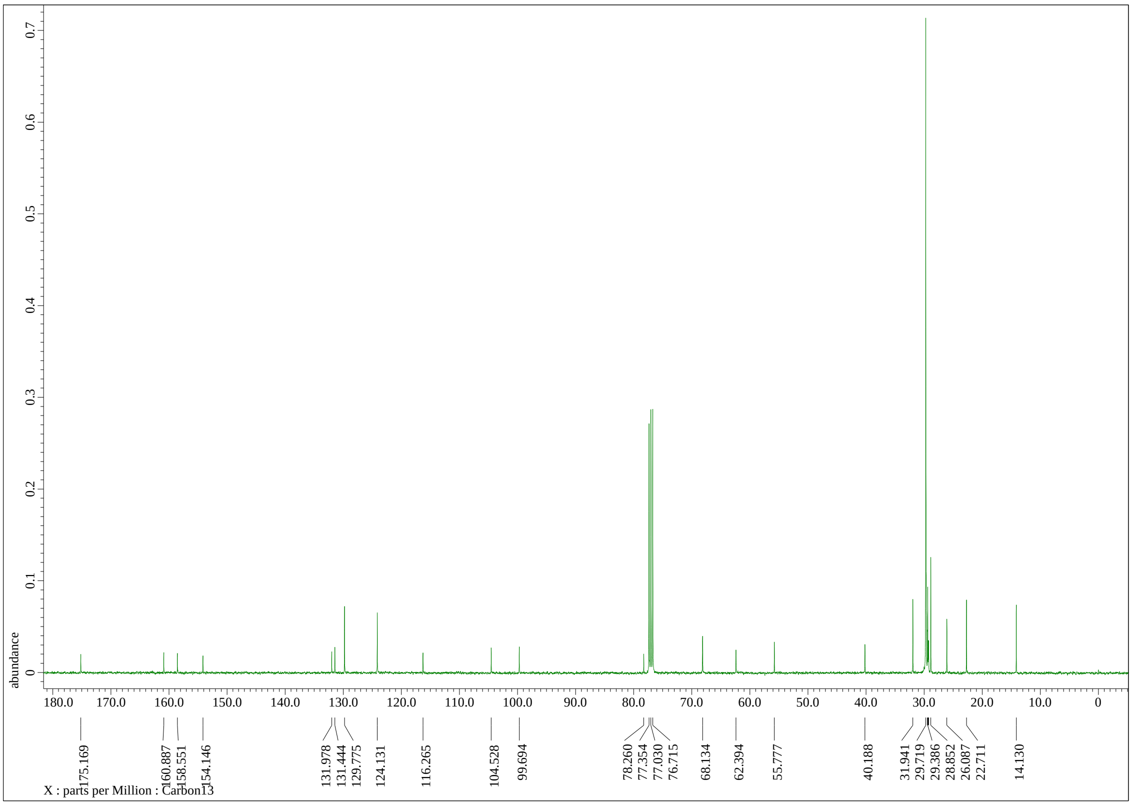

abundance
X : parts per Million : Carbon13

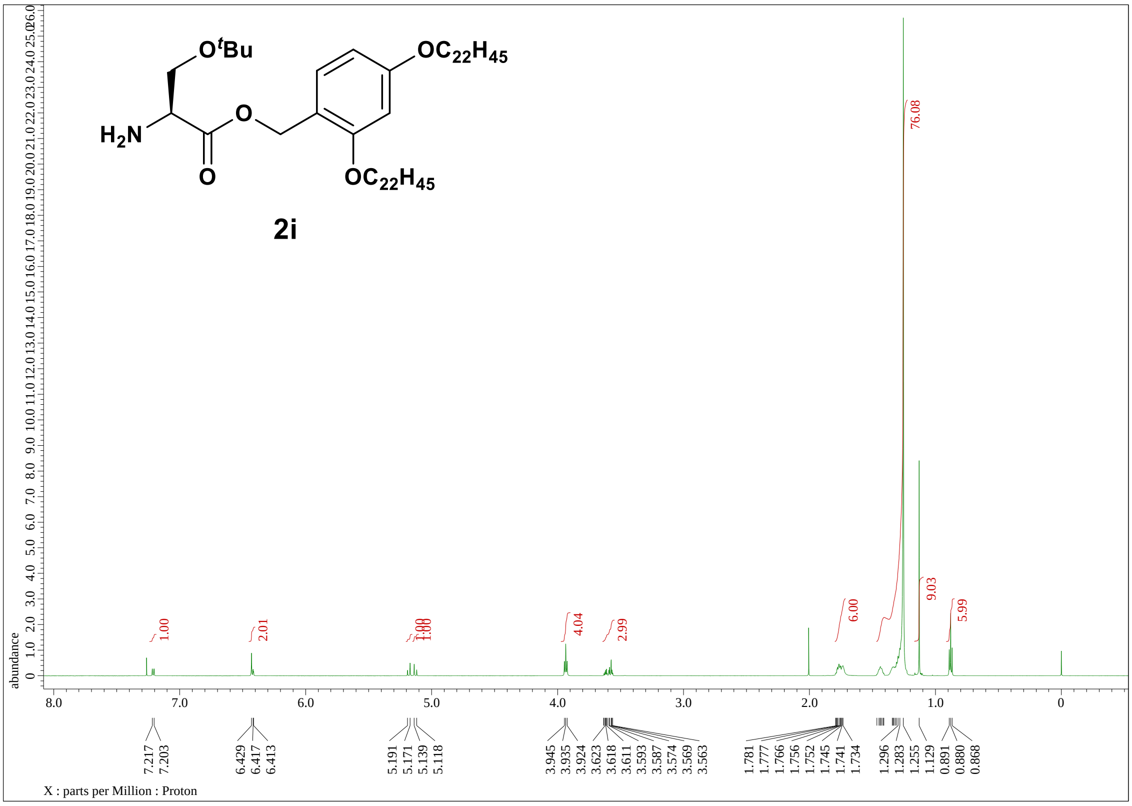
O^{t}Bu
H$_2$N
O
O
OC$_{22}$H$_{45}$
OC$_{22}$H$_{45}$
2i
abundance
1.00
2.01
1.00
1.00
4.04
2.99
6.00
76.08
9.03
5.99
8.0 7.0 6.0 5.0 4.0 3.0 2.0 1.0 0
7.217 7.203
6.429 6.417 6.413
5.191 5.171 5.139 5.118
3.945 3.935 3.924 3.623 3.618 3.611 3.593 3.587 3.574 3.569 3.563
1.781 1.777 1.766 1.756 1.752 1.745 1.741 1.734
1.296 1.283 1.255 1.129 0.891 0.880 0.868
X : parts per Million : Proton

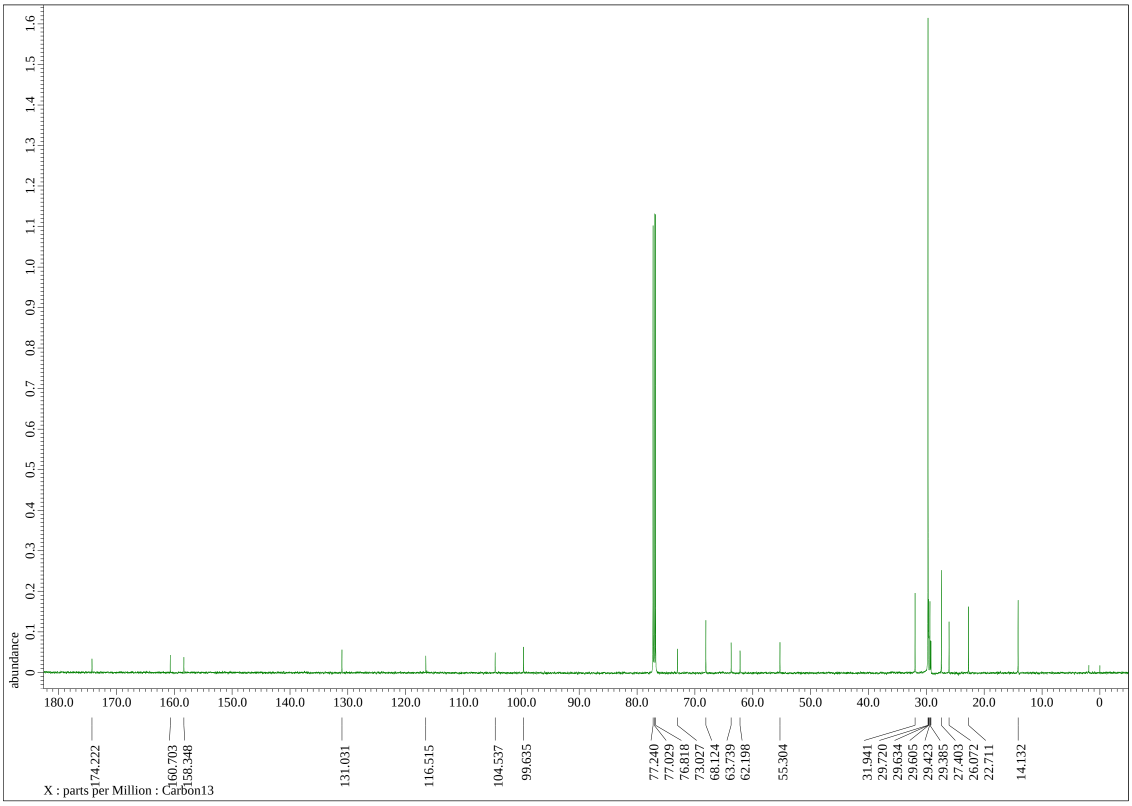
abundance
180.0 170.0 160.0 150.0 140.0 130.0 120.0 110.0 100.0 90.0 80.0 70.0 60.0 50.0 40.0 30.0 20.0 10.0 0
174.222
160.703
158.348
131.031
116.515
104.537
99.635
77.240 77.029 76.818 73.027 68.124 63.739 62.198
55.304
31.941 29.720 29.634 29.605 29.423 29.385 27.403 26.072 22.711
14.132
X : parts per Million : Carbon13

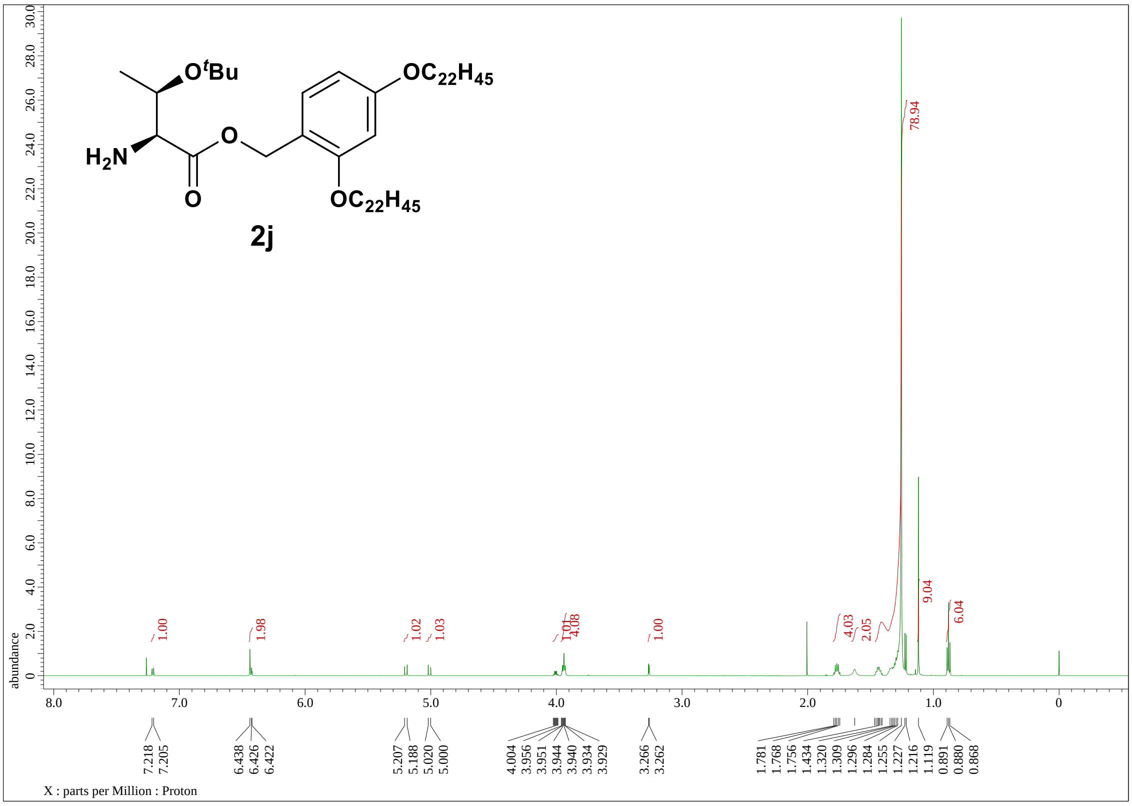
O^{t}Bu
H_2N
O
O
$OC_{22}H_{45}$
$OC_{22}H_{45}$
2j
1.00
1.98
1.02
1.03
1.01
4.08
1.00
4.03
2.05
78.94
9.04
6.04
abundance
7.218 7.205
6.438 6.426 6.422
5.207 5.188 5.020 5.000
4.004 3.956 3.951 3.944 3.940 3.934 3.929
3.266 3.262
1.781 1.768 1.756 1.434 1.320 1.309 1.296 1.284 1.255 1.227 1.216 1.119 0.891 0.880 0.868
X : parts per Million : Proton

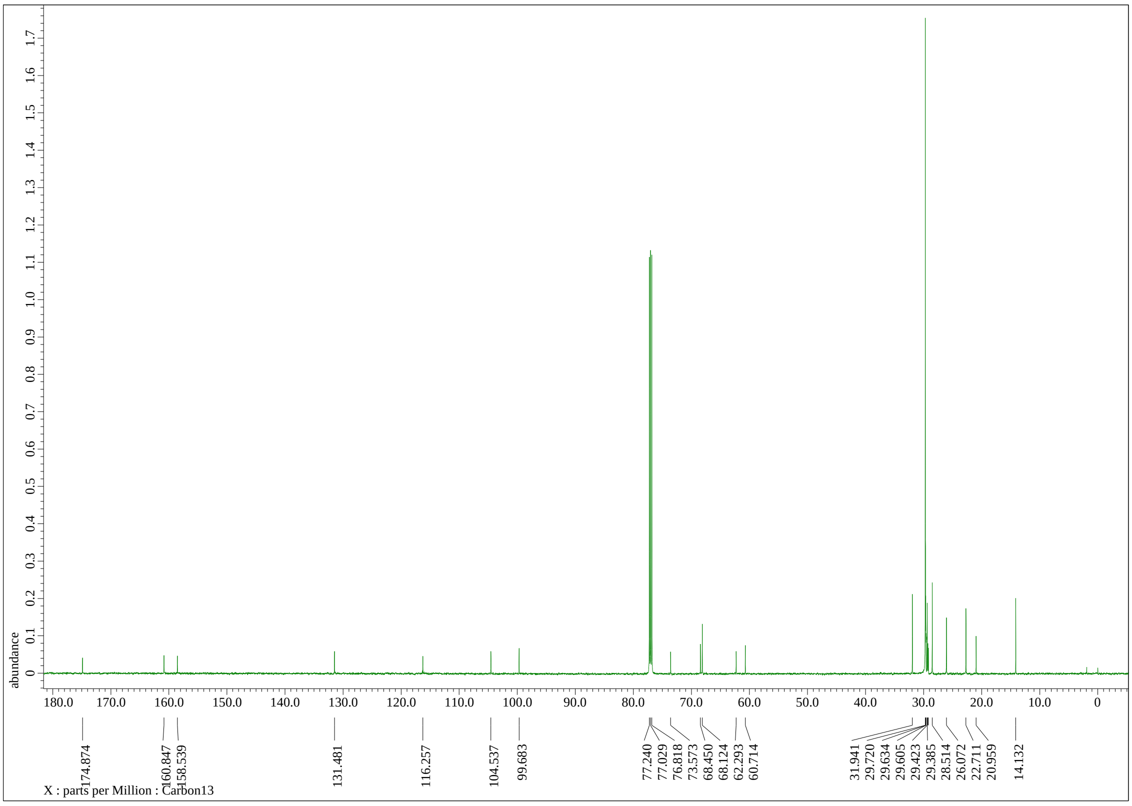
abundance
174.874
160.847 158.539
131.481
116.257
104.537
99.683
77.240 77.029 76.818 73.573 68.450 68.124 62.293 60.714
31.941 29.720 29.634 29.605 29.423 29.385 28.514 26.072 22.711 20.959
14.132
X : parts per Million : Carbon13

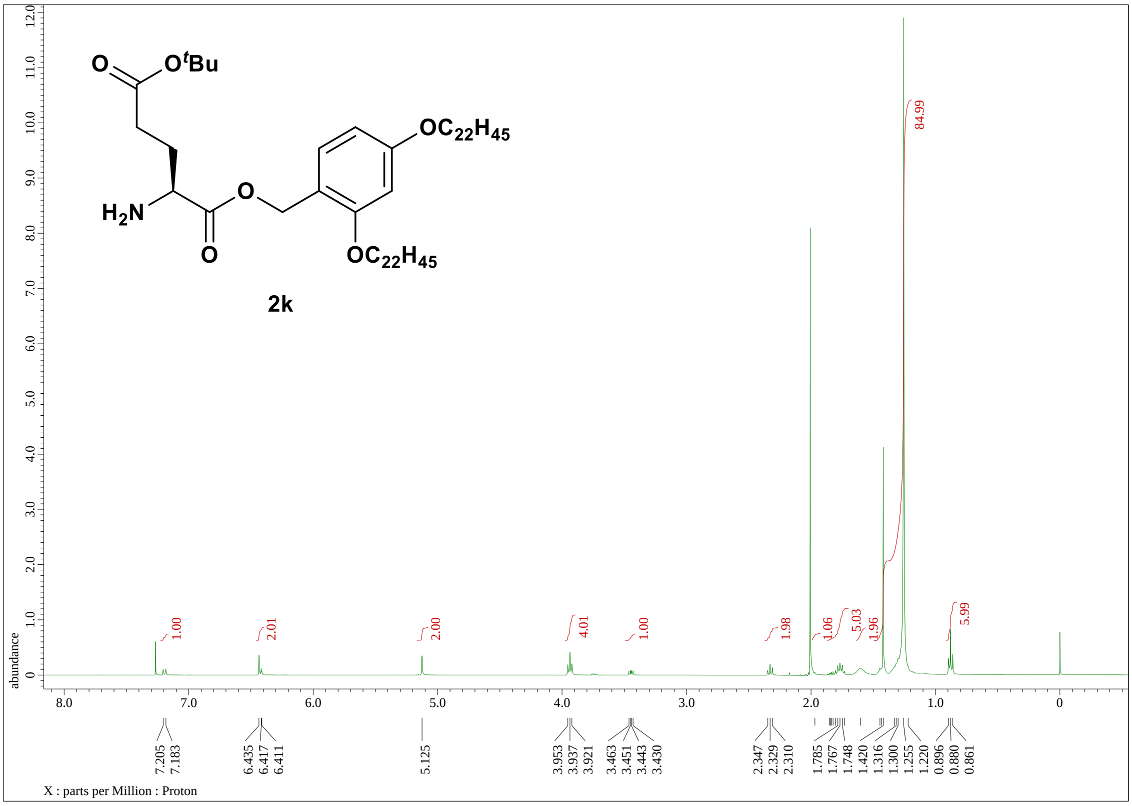
O^{t}Bu
NH_2
$OC_{22}H_{45}$
$OC_{22}H_{45}$
2k
abundance
1.00
2.01
2.00
4.01
1.00
1.98
1.06
5.03
1.96
84.99
5.99
7.205 7.183
6.435 6.417 6.411
5.125
3.953 3.937 3.921
3.463 3.451 3.443 3.430
2.347 2.329 2.310
1.785 1.767 1.748
1.420 1.316 1.300 1.255 1.220
0.896 0.880 0.861
X : parts per Million : Proton

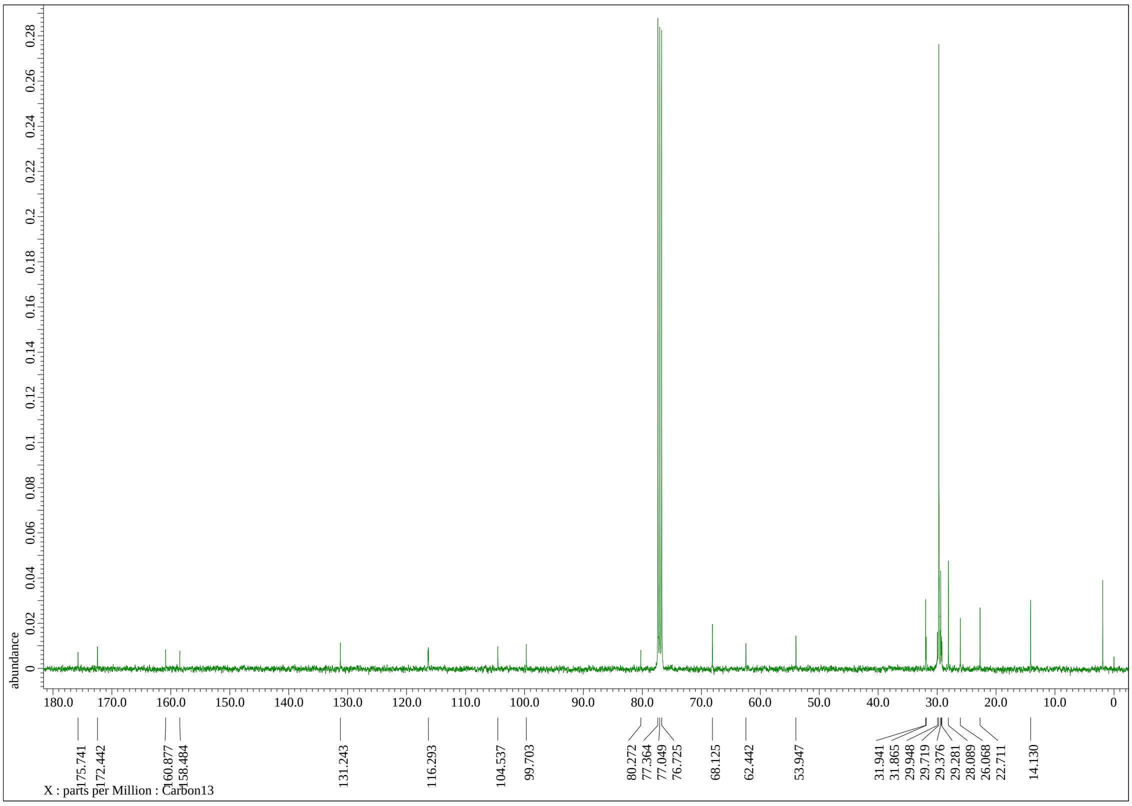
abundance
175.741
172.442
160.877
158.484
131.243
116.293
104.537
99.703
80.272
77.364
77.049
76.725
68.125
62.442
53.947
31.941
31.865
29.948
29.719
29.376
29.281
28.089
26.068
22.711
14.130
X : parts per Million : Carbon13

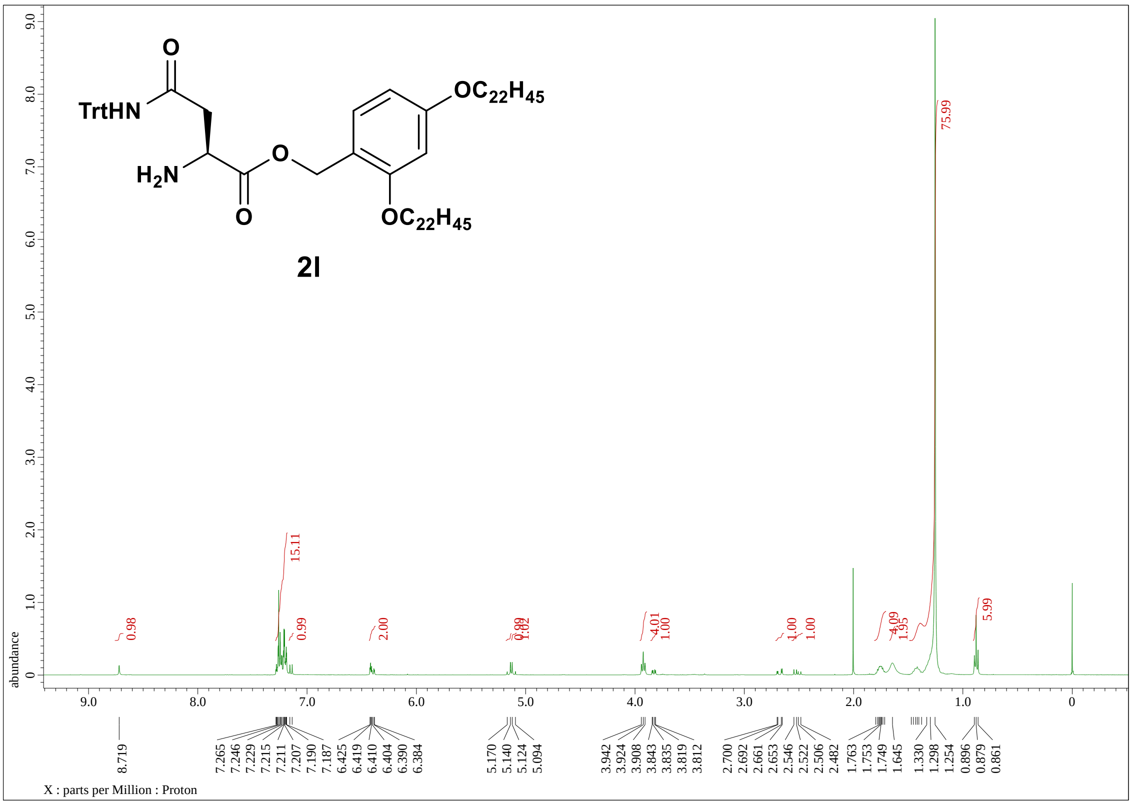

TrtHN
H2N
OC22H45
OC22H45
2l
abundance
X : parts per Million : Proton

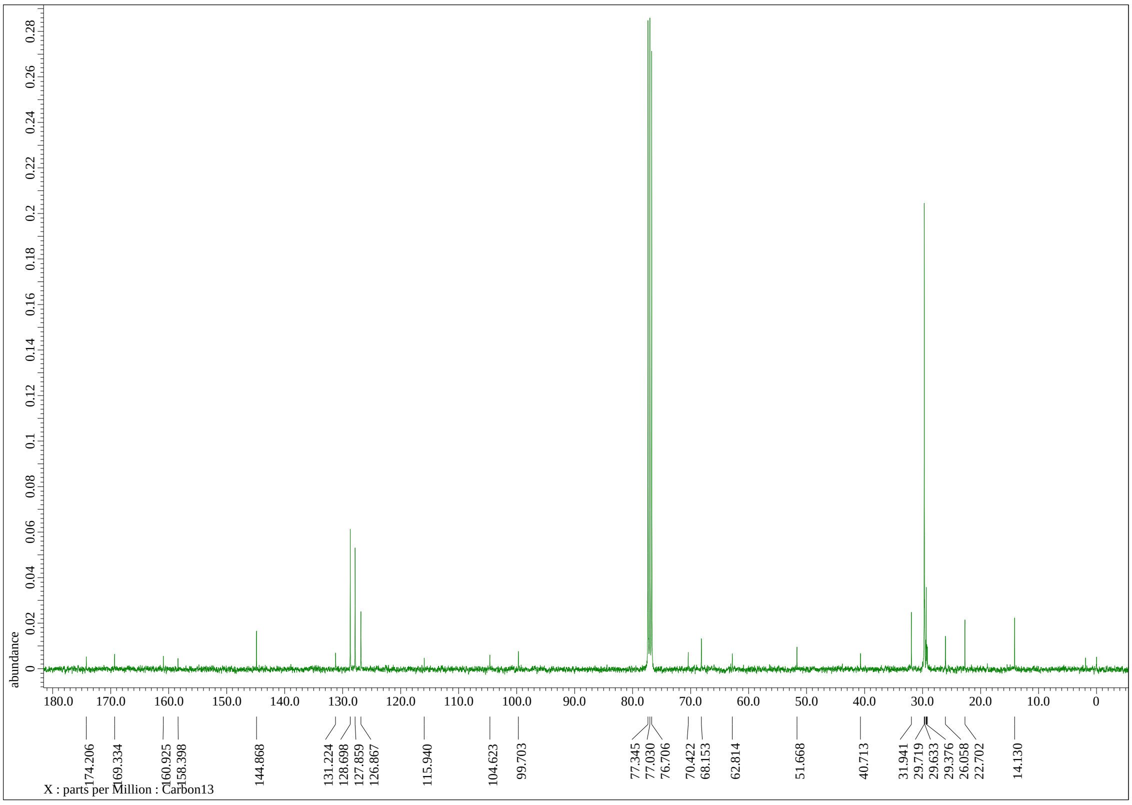

abundance
X : parts per Million : Carbon13

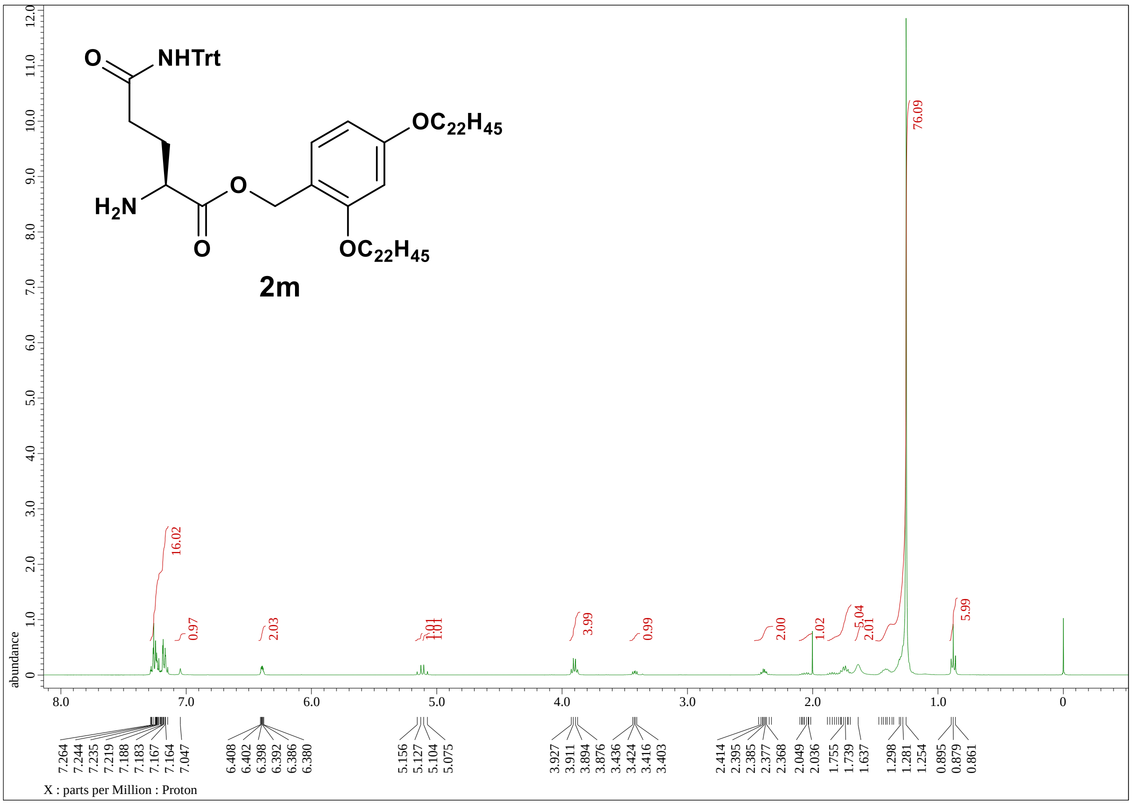
O
NHTrt
OC22H45
H2N
O
O
OC22H45
2m
16.02
0.97
2.03
1.01
1.01
3.99
0.99
2.00
1.02
5.04
2.01
76.09
5.99
7.264 7.244 7.235 7.219 7.188 7.183 7.167 7.164 7.047
6.408 6.402 6.398 6.392 6.386 6.380
5.156 5.127 5.104 5.075
3.927 3.911 3.894 3.876 3.436 3.424 3.416 3.403
2.414 2.395 2.385 2.377 2.368 2.049 2.036 1.755 1.739 1.637 1.298 1.281 1.254 0.895 0.879 0.861
abundance
X : parts per Million : Proton

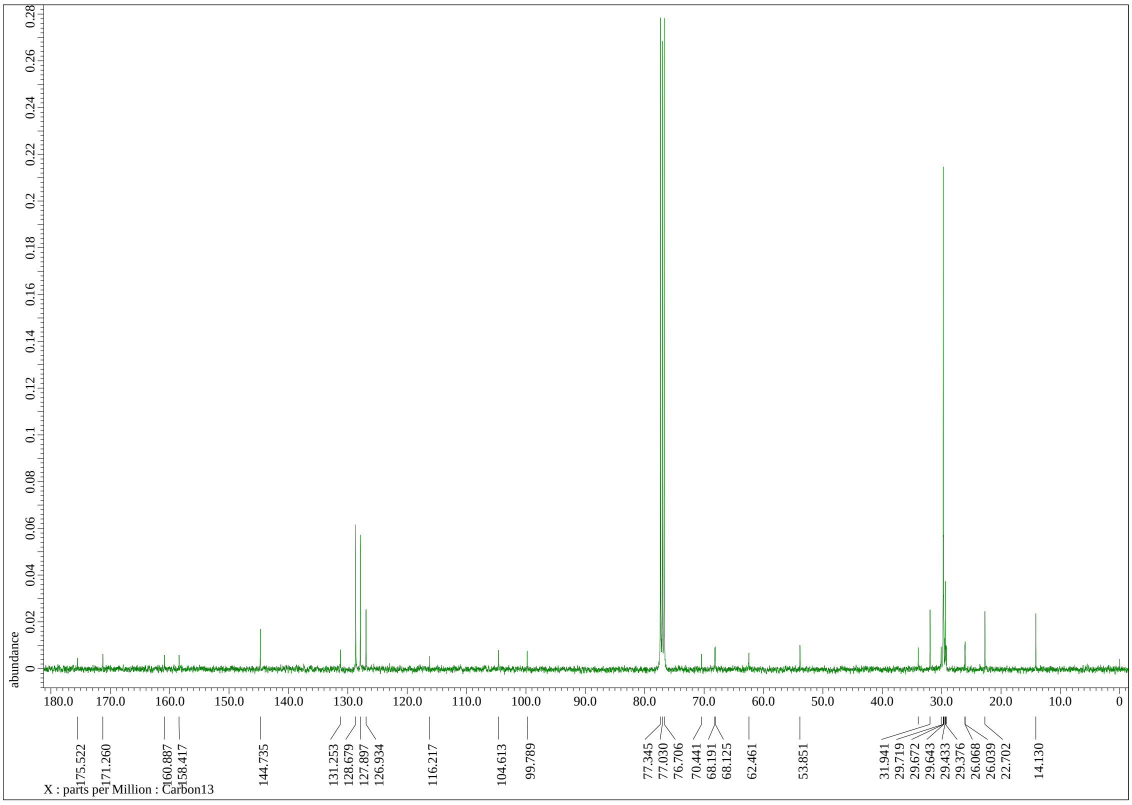
175.522 171.260 160.887 158.417 144.735 131.253 128.679 127.897 126.934 116.217 104.613 99.789 77.345 77.030 76.706 70.441 68.191 68.125 62.461 53.851 31.941 29.719 29.672 29.643 29.433 29.376 26.068 26.039 22.702 14.130
abundance
X : parts per Million : Carbon13

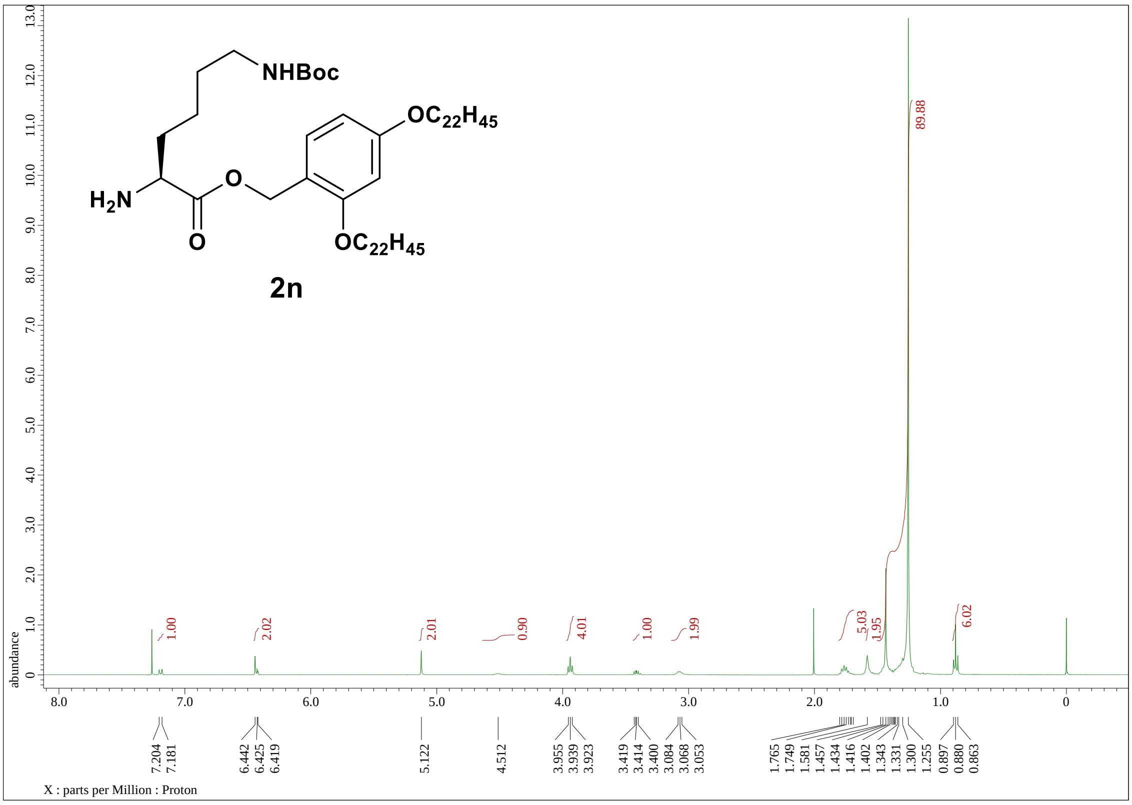
NHBoc
H_2N
O
O
$OC_{22}H_{45}$
$OC_{22}H_{45}$
2n
abundance
1.00
2.02
2.01
0.90
4.01
1.00
1.99
5.03
1.95
89.88
6.02
7.204
7.181
6.442
6.425
6.419
5.122
4.512
3.955
3.939
3.923
3.419
3.414
3.400
3.084
3.068
3.053
1.765
1.749
1.581
1.457
1.434
1.416
1.402
1.343
1.331
1.300
1.255
0.897
0.880
0.863
X : parts per Million : Proton

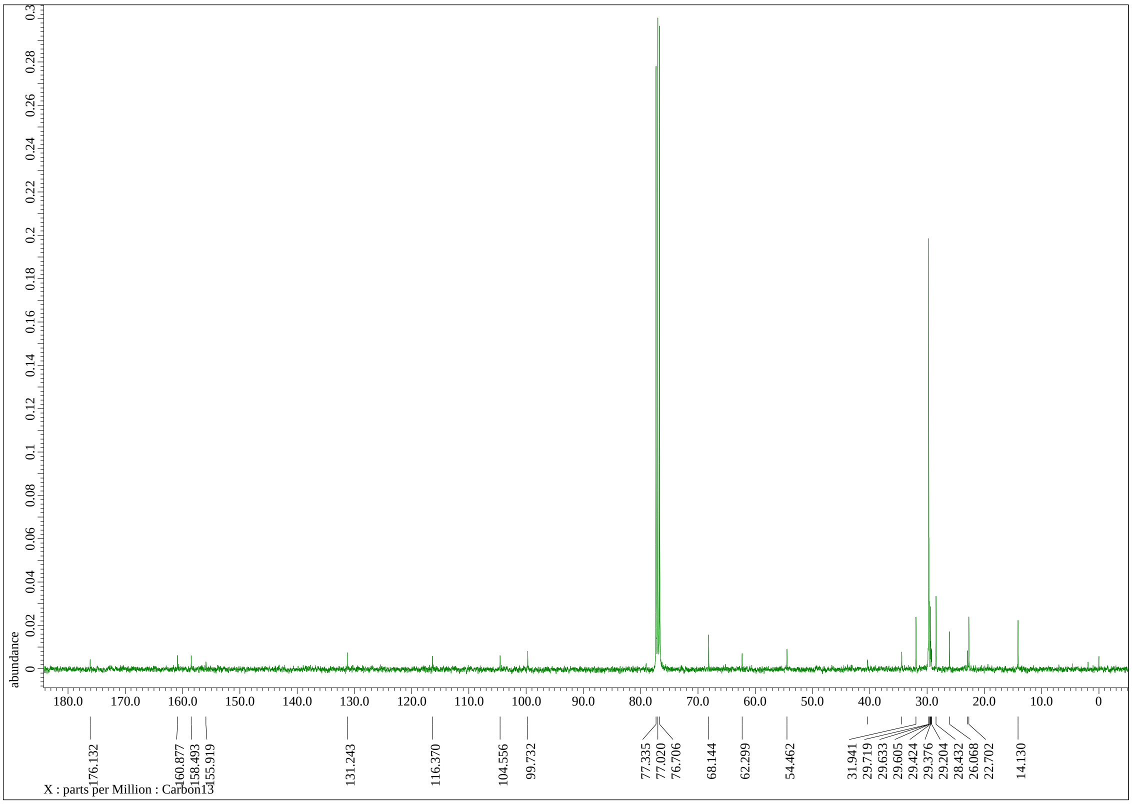
abundance
176.132
160.877
158.493
155.919
131.243
116.370
104.556
99.732
77.335
77.020
76.706
68.144
62.299
54.462
31.941
29.719
29.633
29.605
29.424
29.376
29.204
28.432
26.068
22.702
14.130
X : parts per Million : Carbon13

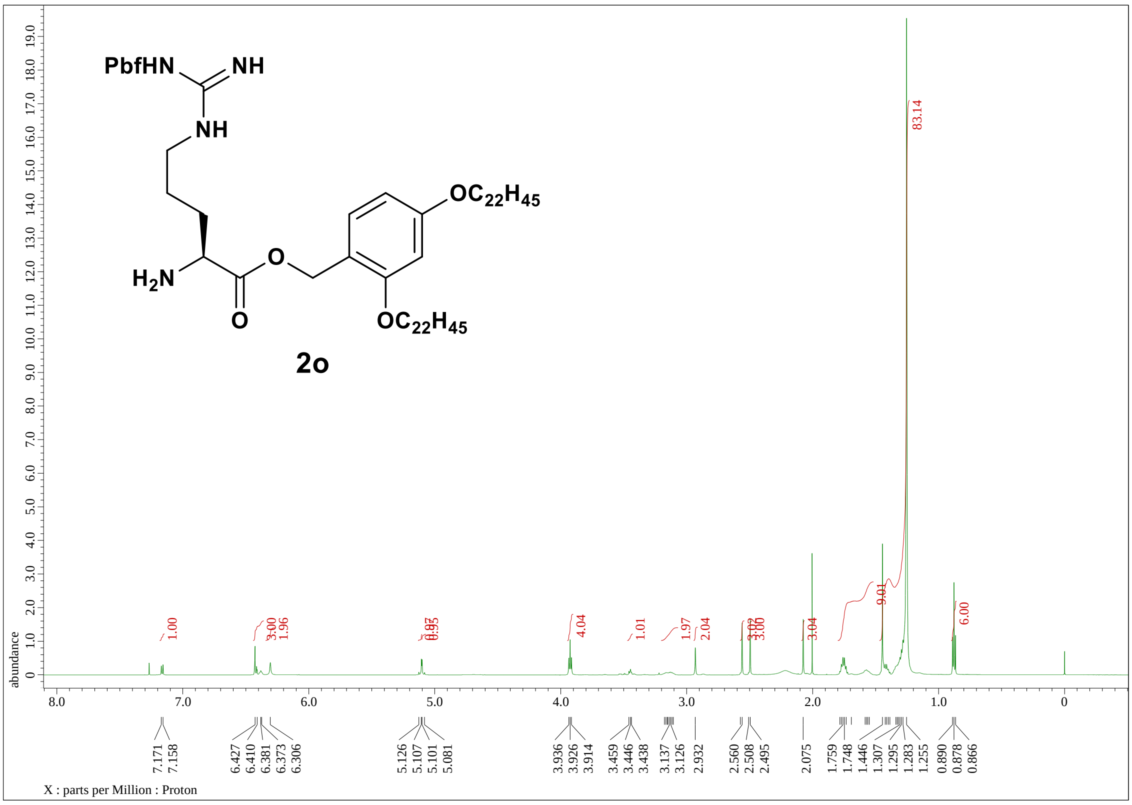
PbfHN
NH
NH
H2N
O
O
OC22H45
OC22H45
2o
X : parts per Million : Proton
abundance

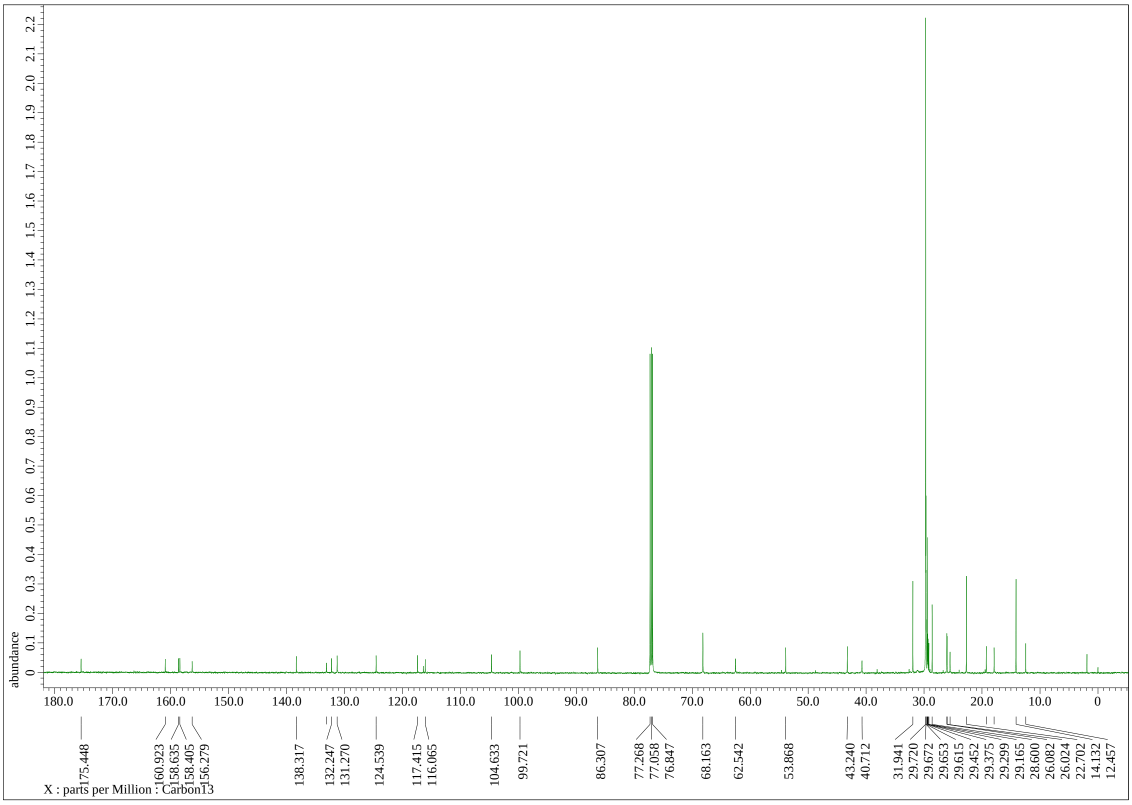
X : parts per Million : Carbon13
abundance

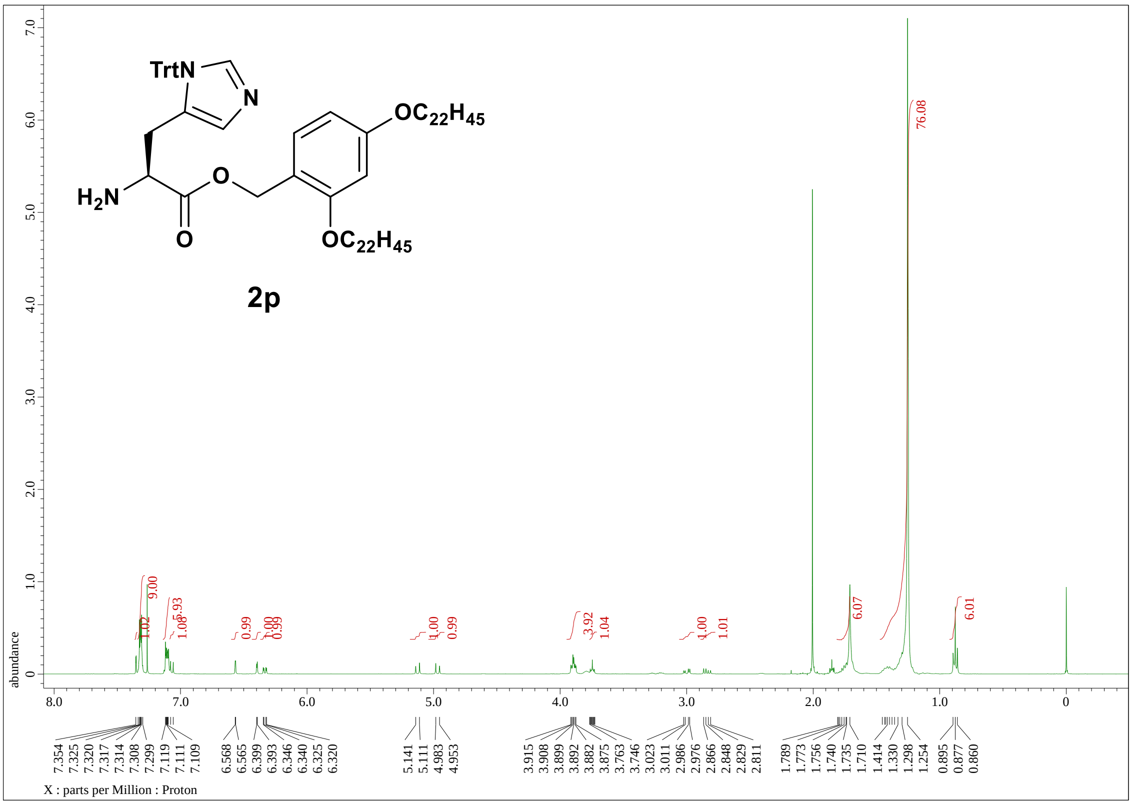

TrtN
N
H2N
O
O
OC22H45
OC22H45
2p
X : parts per Million : Proton
abundance

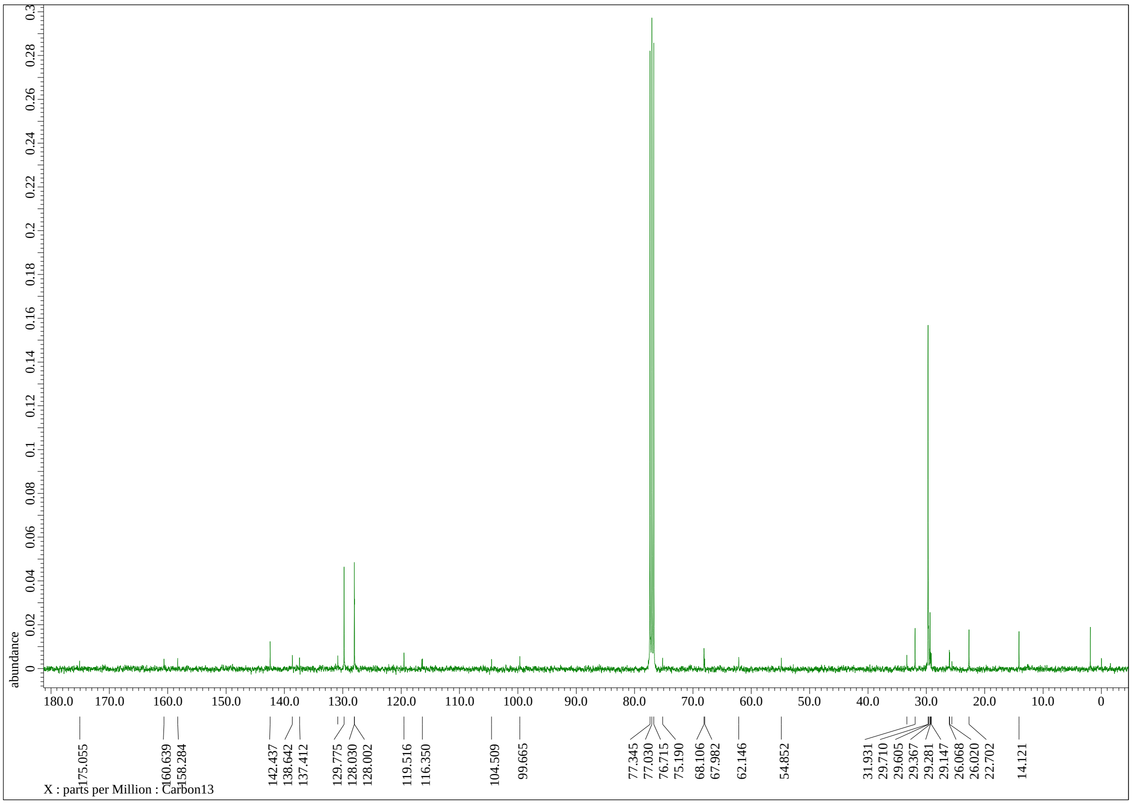

X : parts per Million : Carbon13
abundance

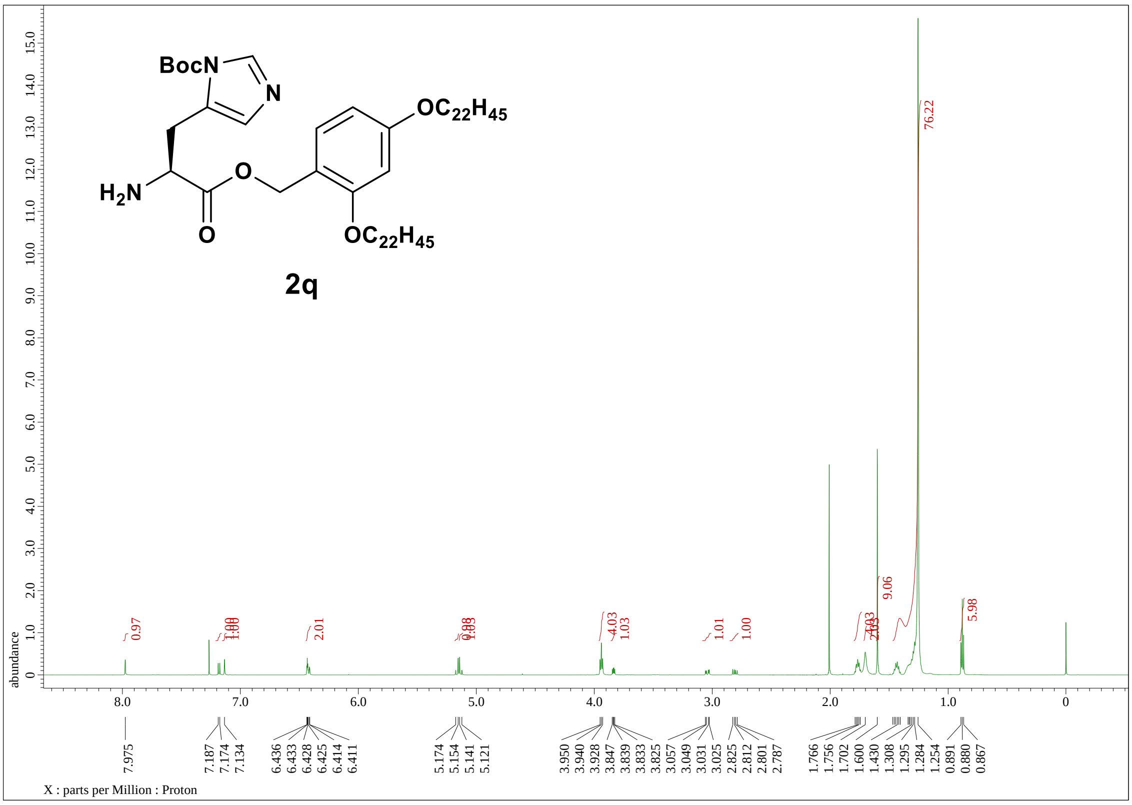

BocN
N
H2N
O
O
OC22H45
OC22H45
2q
abundance
0.97
1.00
1.00
2.01
0.88
1.03
4.03
1.03
1.01
1.00
2.03
9.06
76.22
5.98
7.975
7.187
7.174
7.134
6.436
6.433
6.428
6.425
6.414
6.411
5.174
5.154
5.141
5.121
3.950
3.940
3.928
3.847
3.839
3.833
3.825
3.057
3.049
3.031
3.025
2.825
2.812
2.801
2.787
1.766
1.756
1.702
1.600
1.430
1.308
1.295
1.284
1.254
0.891
0.880
0.867
X : parts per Million : Proton

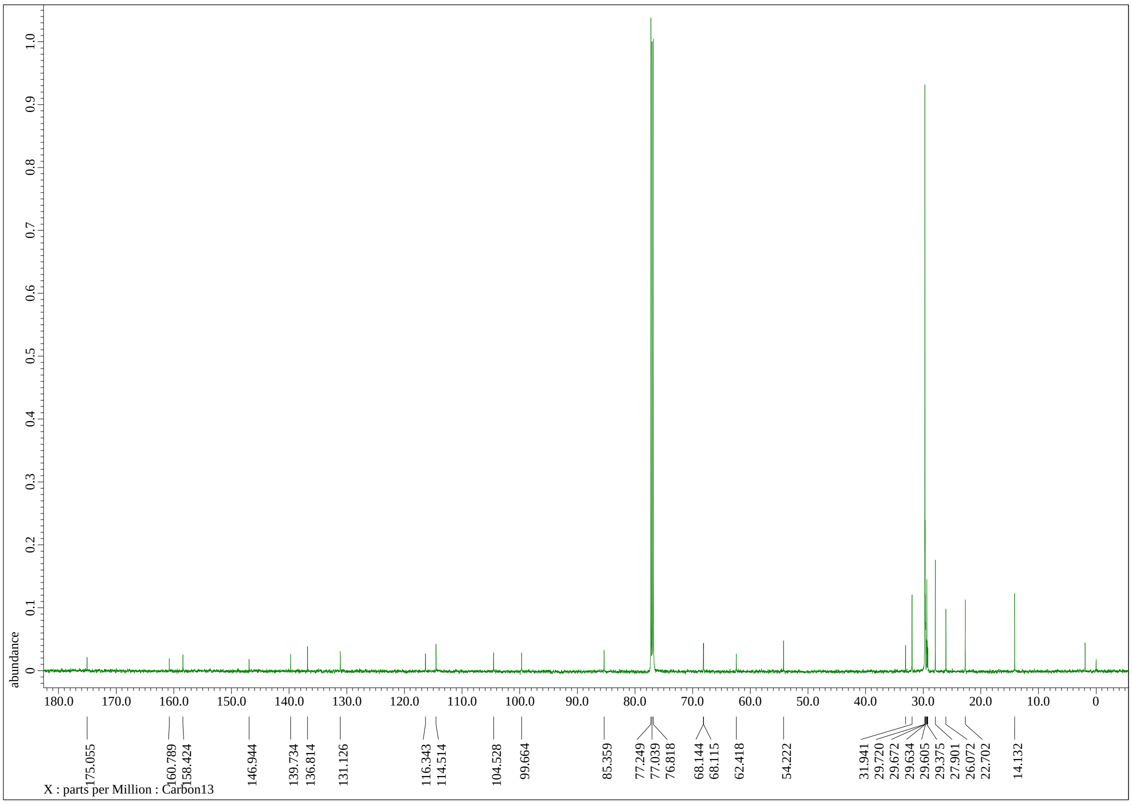

abundance
175.055
160.789
158.424
146.944
139.734
136.814
131.126
116.343
114.514
104.528
99.664
85.359
77.249
77.039
76.818
68.144
68.115
62.418
54.222
31.941
29.720
29.672
29.634
29.605
29.375
27.901
26.072
22.702
14.132
X : parts per Million : Carbon13

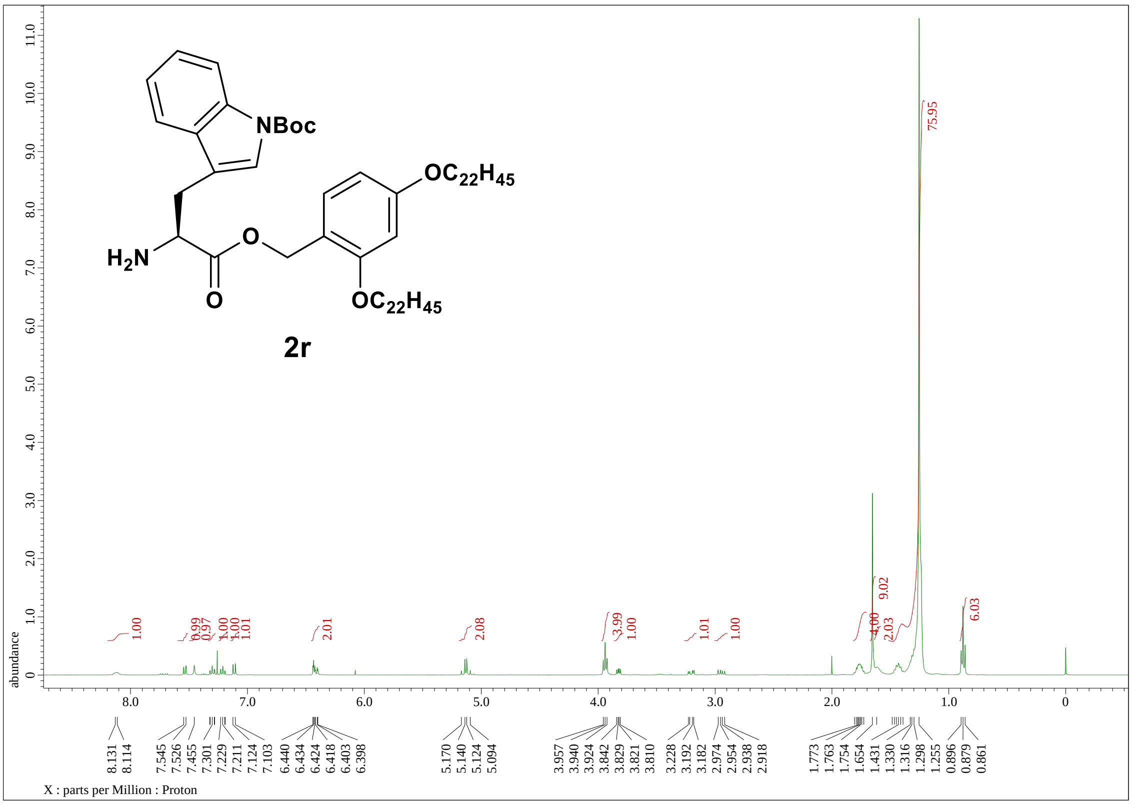
NBoc
H2N
O
O
OC22H45
OC22H45
2r
abundance
X : parts per Million : Proton
1.00 0.99 0.97 1.00 1.00 1.01 2.01 2.08 3.99 1.00 1.01 1.00 4.00 9.02 2.03 75.95 6.03
8.131 8.114 7.545 7.526 7.455 7.301 7.229 7.211 7.124 7.103 6.440 6.434 6.424 6.418 6.403 6.398
5.170 5.140 5.124 5.094
3.957 3.940 3.924 3.842 3.829 3.821 3.810 3.228 3.192 3.182 2.974 2.954 2.938 2.918
1.773 1.763 1.754 1.654 1.431 1.330 1.316 1.298 1.255 0.896 0.879 0.861

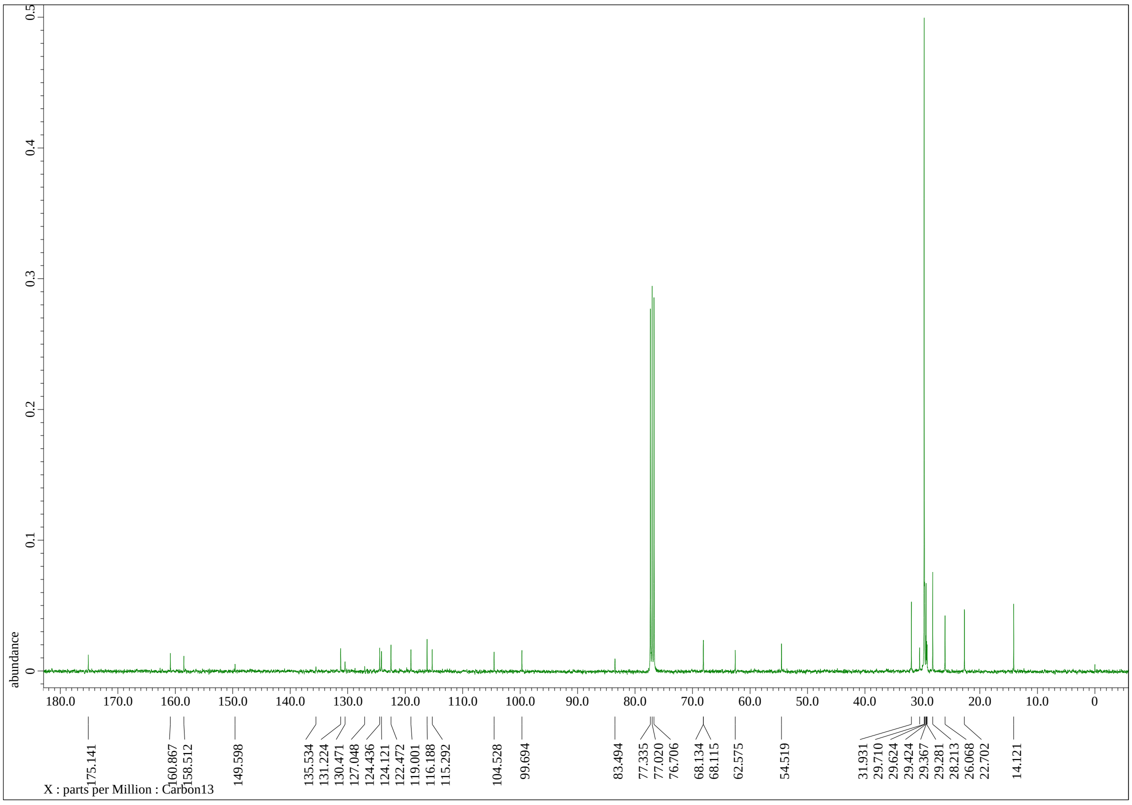
abundance
X : parts per Million : Carbon13
175.141 160.867 158.512 149.598 135.534 131.224 130.471 127.048 124.436 124.121 122.472 119.001 116.188 115.292 104.528 99.694 83.494 77.335 77.020 76.706 68.134 68.115 62.575 54.519 31.931 29.710 29.624 29.424 29.367 29.281 28.213 26.068 22.702 14.121

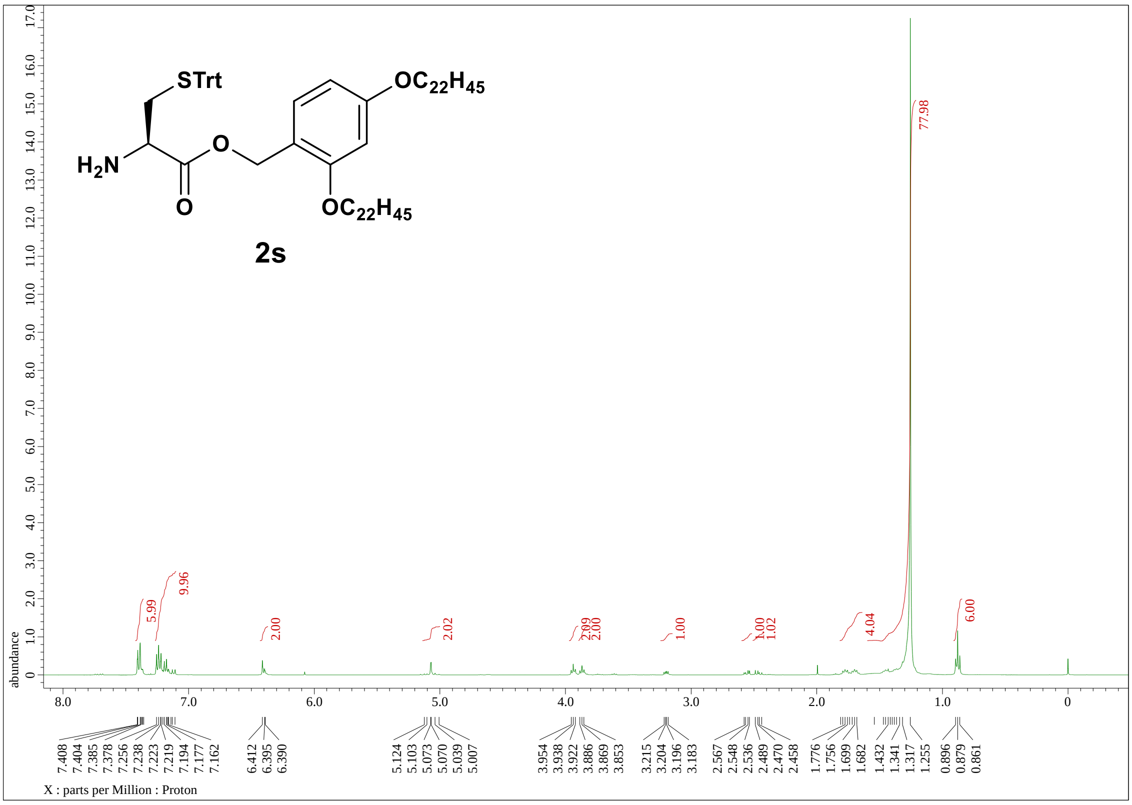
STrt
H_2N
O
O
$OC_{22}H_{45}$
$OC_{22}H_{45}$
2s
abundance
X : parts per Million : Proton
5.99
9.96
2.00
2.02
2.09
2.00
1.00
1.00
1.02
4.04
77.98
6.00
7.408
7.404
7.385
7.378
7.256
7.238
7.223
7.219
7.194
7.177
7.162
6.412
6.395
6.390
5.124
5.103
5.073
5.070
5.039
5.007
3.954
3.938
3.922
3.886
3.869
3.853
3.215
3.204
3.196
3.183
2.567
2.548
2.536
2.489
2.470
2.458
1.776
1.756
1.699
1.682
1.432
1.341
1.317
1.255
0.896
0.879
0.861

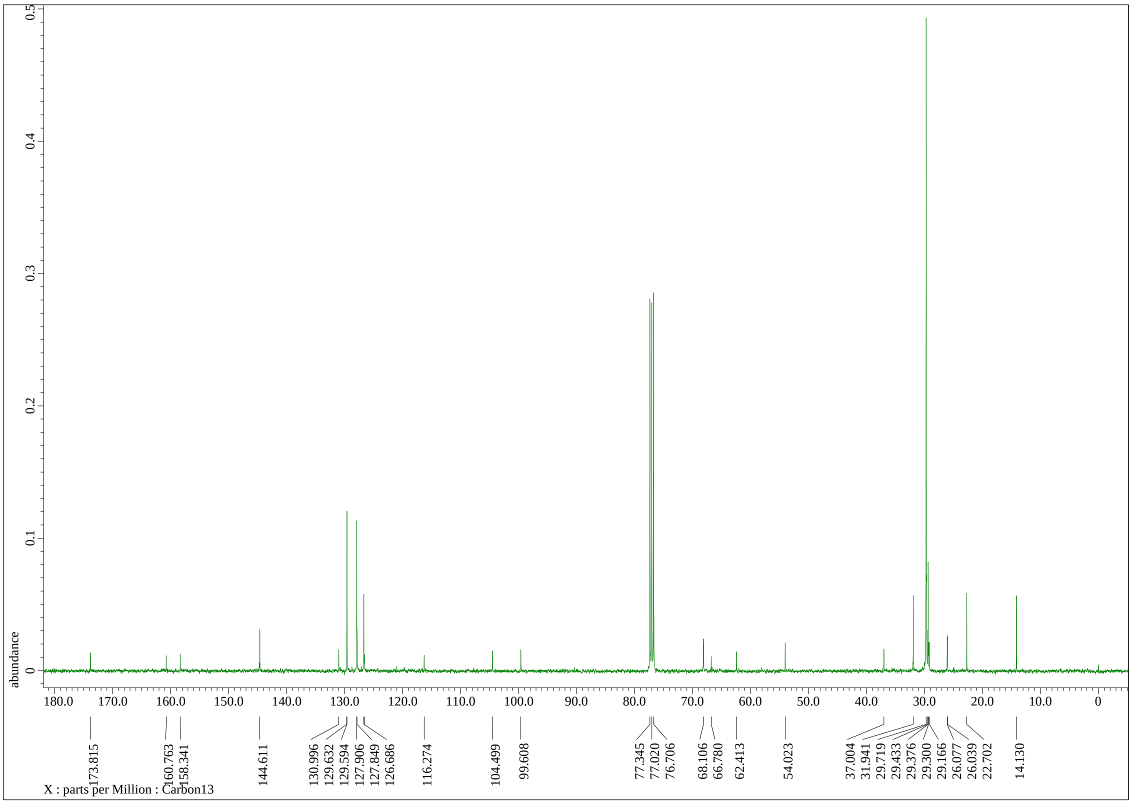
abundance
X : parts per Million : Carbon13
173.815
160.763
158.341
144.611
130.996
129.632
129.594
127.906
127.849
126.686
116.274
104.499
99.608
77.345
77.020
76.706
68.106
66.780
62.413
54.023
37.004
31.941
29.719
29.433
29.376
29.300
29.166
26.077
26.039
22.702
14.130

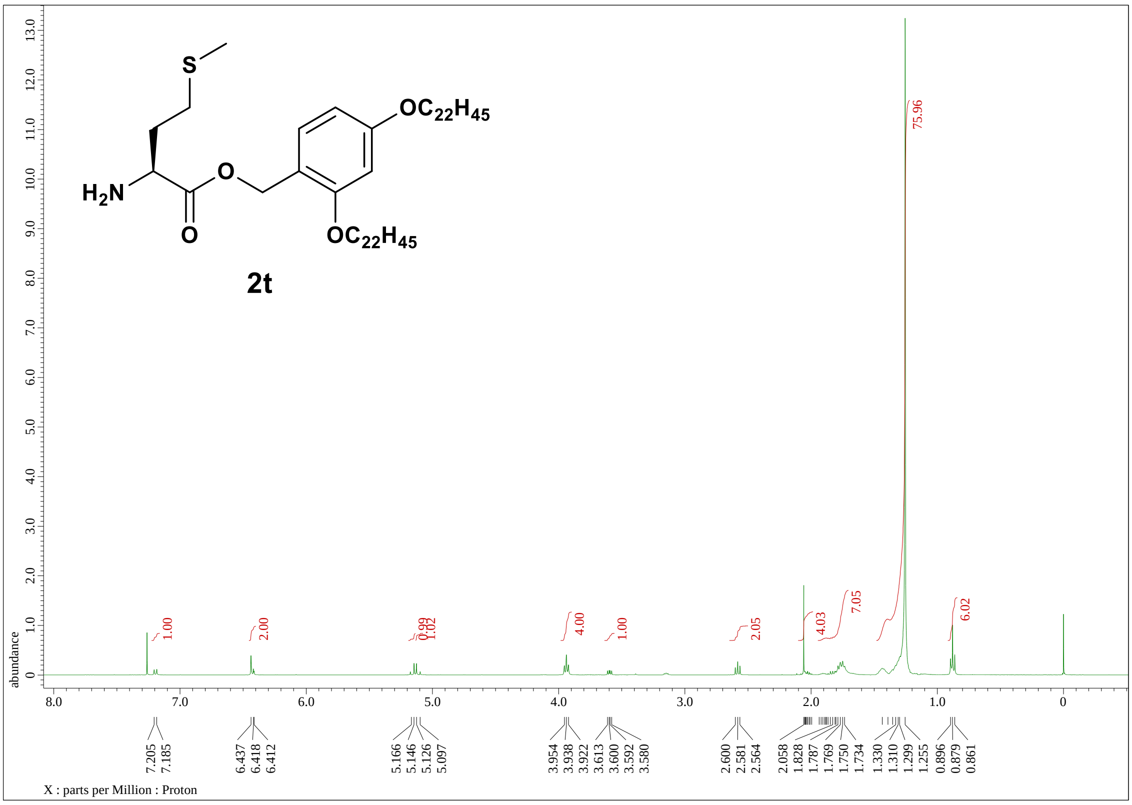
S
H2N
O
O
OC22H45
OC22H45
2t
abundance
X : parts per Million : Proton
7.205
7.185
6.437
6.418
6.412
5.166
5.146
5.126
5.097
3.954
3.938
3.922
3.613
3.600
3.592
3.580
2.600
2.581
2.564
2.058
1.828
1.787
1.769
1.750
1.734
1.330
1.310
1.299
1.255
0.896
0.879
0.861
1.00
2.00
1.02
4.00
1.00
2.05
4.03
7.05
75.96
6.02

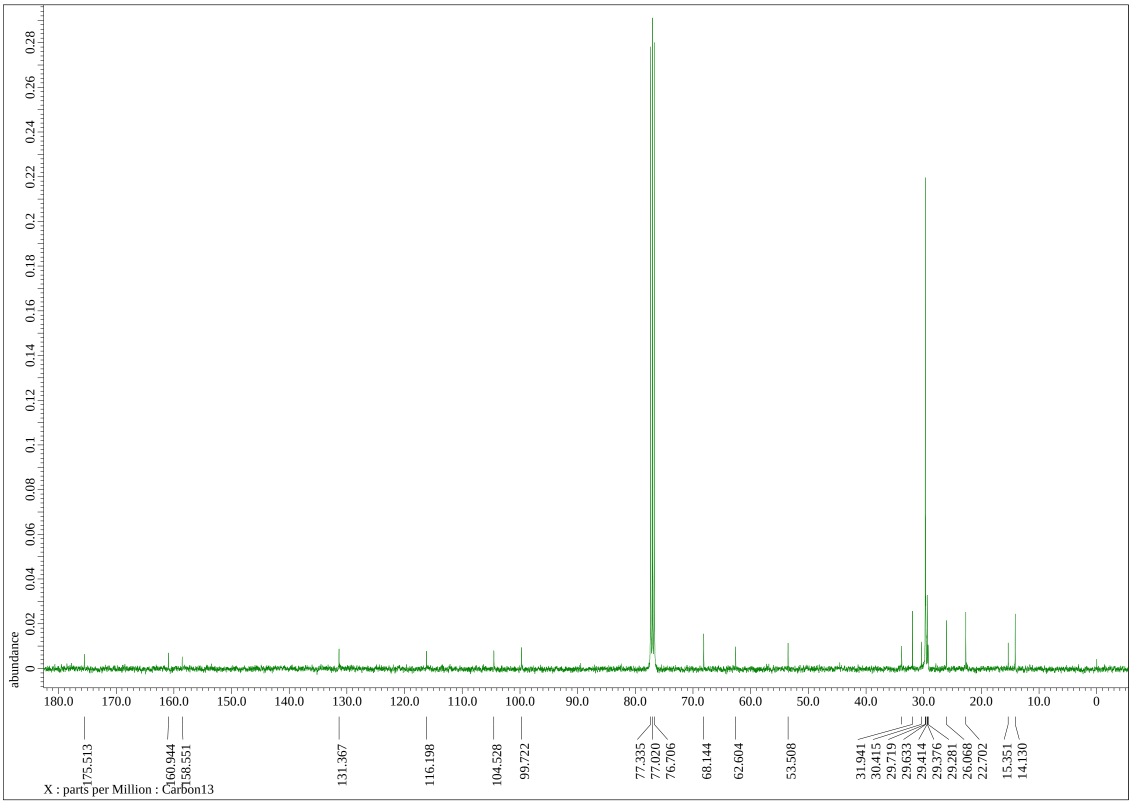
abundance
X : parts per Million : Carbon13
175.513
160.944
158.551
131.367
116.198
104.528
99.722
77.335
77.020
76.706
68.144
62.604
53.508
31.941
30.415
29.719
29.633
29.414
29.376
29.281
26.068
22.702
15.351
14.130

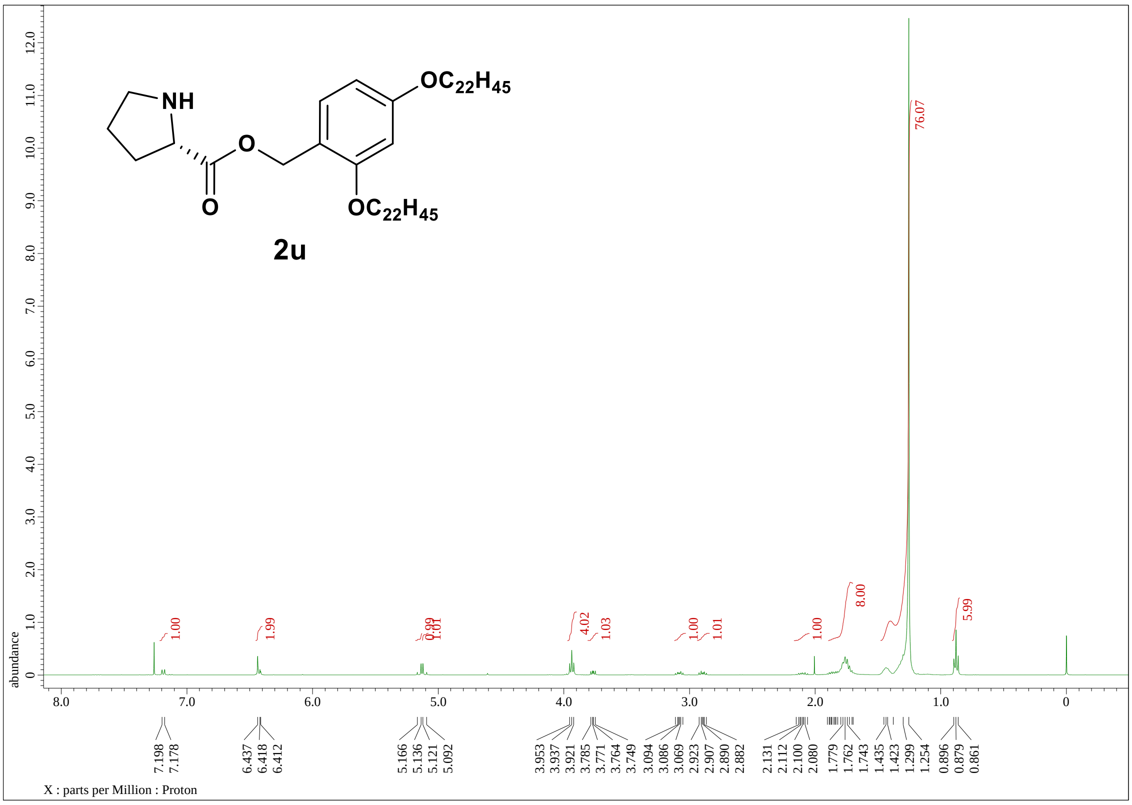
NH
O
O
OC22H45
OC22H45
2u
76.07
8.00
5.99
1.00
1.99
0.99
1.01
4.02
1.03
1.00
1.01
1.00
abundance
X : parts per Million : Proton
7.198
7.178
6.437
6.418
6.412
5.166
5.136
5.121
5.092
3.953
3.937
3.921
3.785
3.771
3.764
3.749
3.094
3.086
3.069
2.923
2.907
2.890
2.882
2.131
2.112
2.100
2.080
1.779
1.762
1.743
1.435
1.423
1.299
1.254
0.896
0.879
0.861

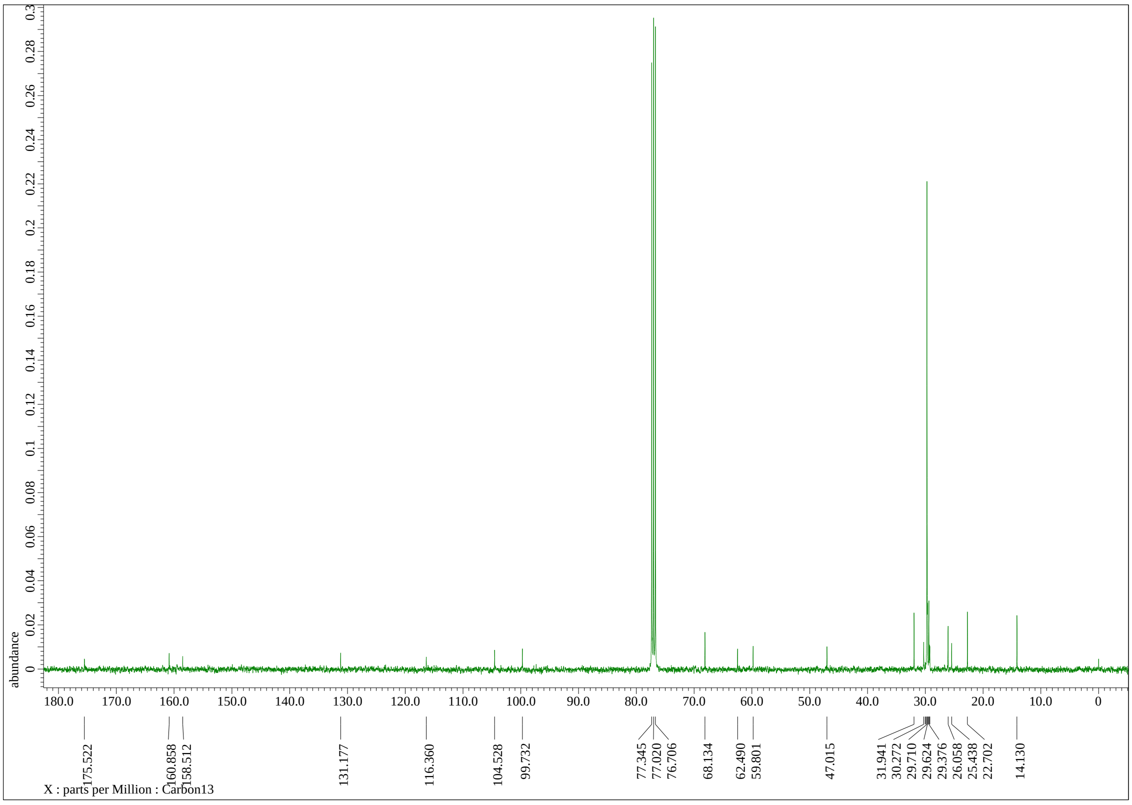
abundance
X : parts per Million : Carbon13
175.522
160.858
158.512
131.177
116.360
104.528
99.732
77.345
77.020
76.706
68.134
62.490
59.801
47.015
31.941
30.272
29.710
29.624
29.376
26.058
25.438
22.702
14.130

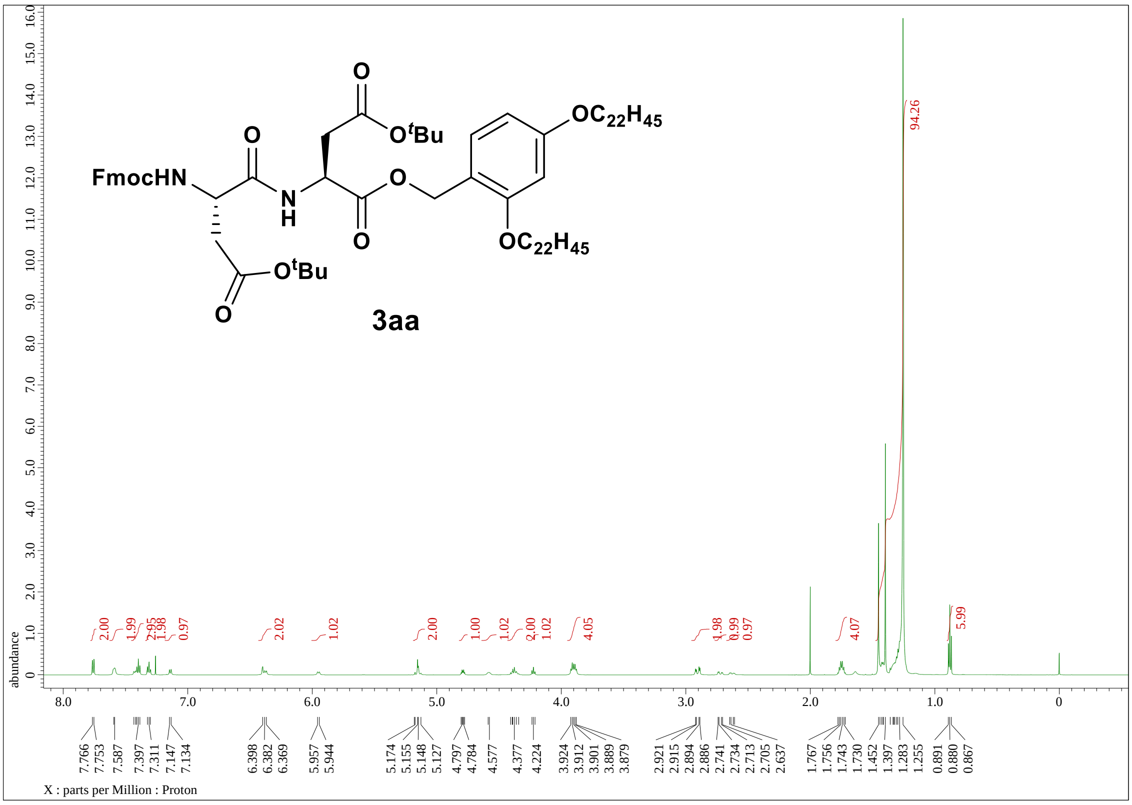
FmocHN
O
N
H
O
O
OtBu
O
O
OtBu
OC22H45
OC22H45
3aa
X : parts per Million : Proton
abundance

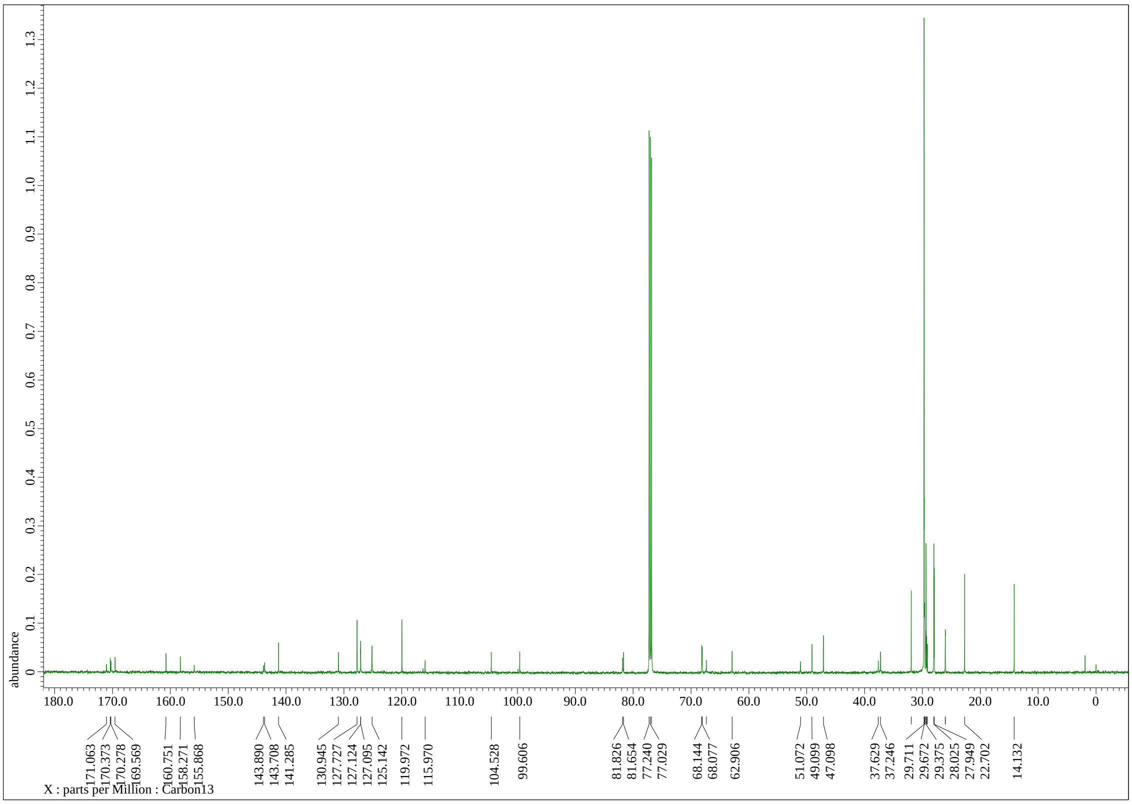
X : parts per Million : Carbon13
abundance

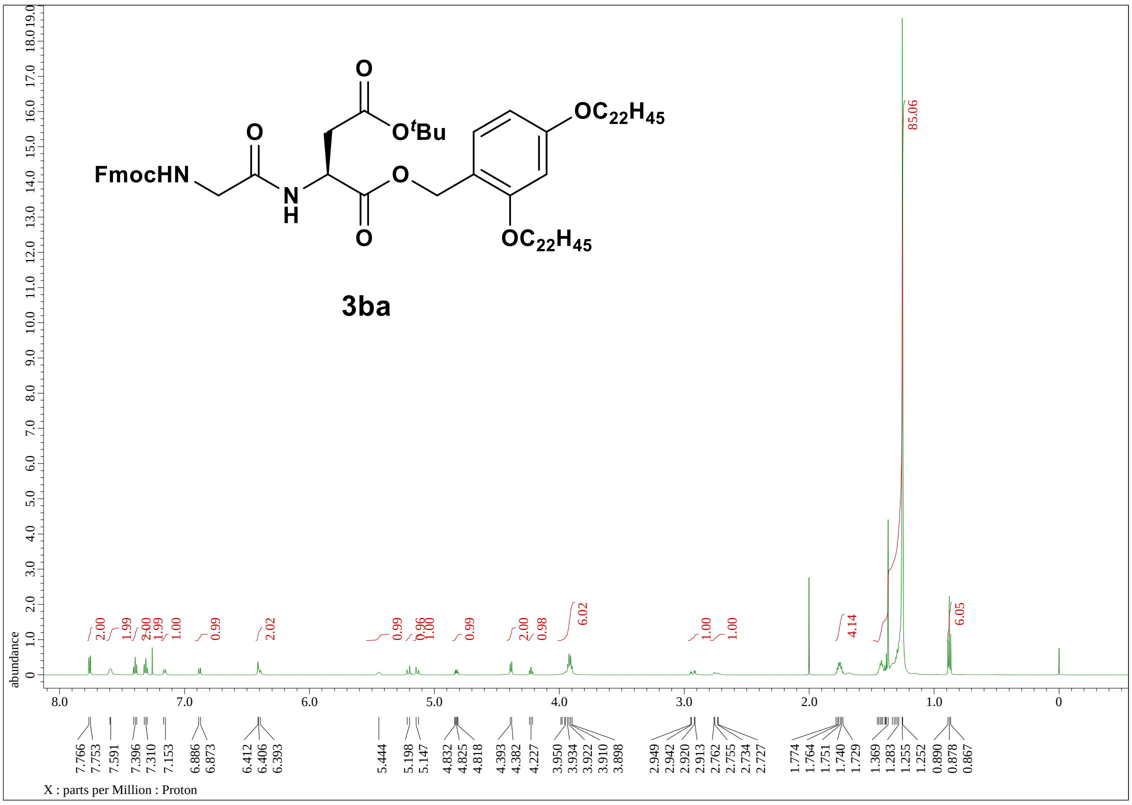
FmocHN
O
N
H
O
O
O
O^tBu
OC22H45
OC22H45
3ba
X : parts per Million : Proton
abundance

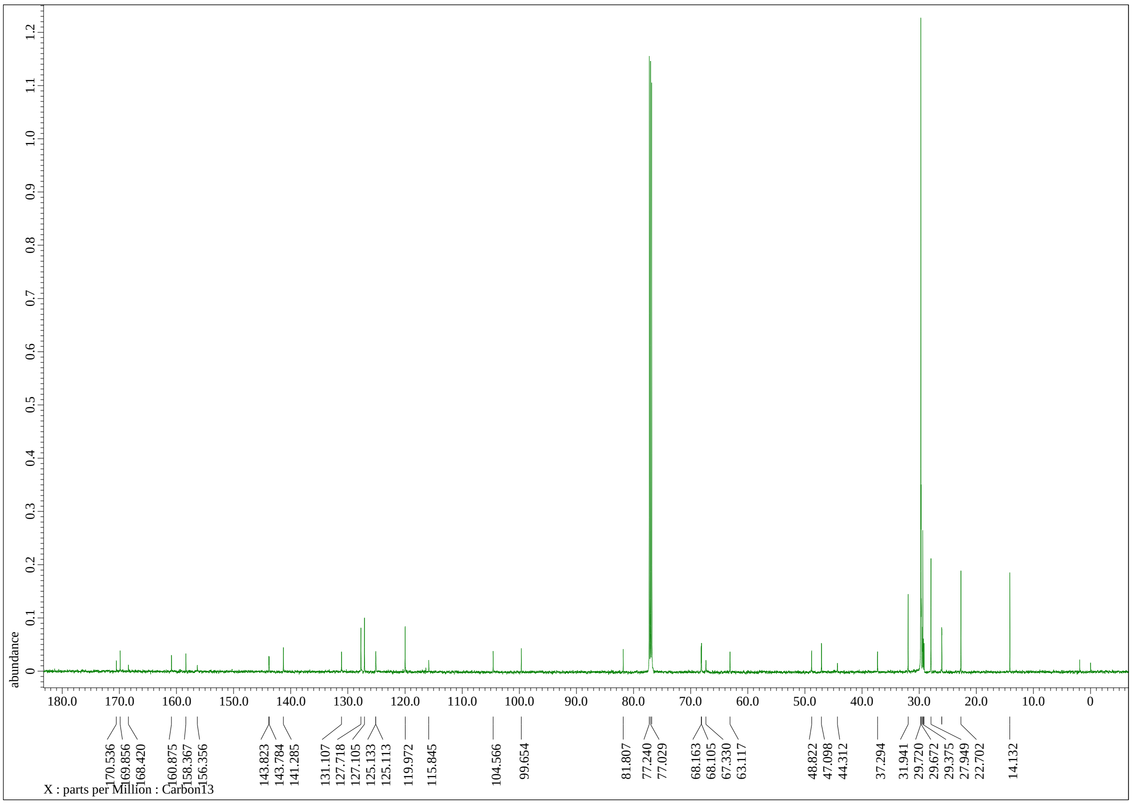
X : parts per Million : Carbon13
abundance

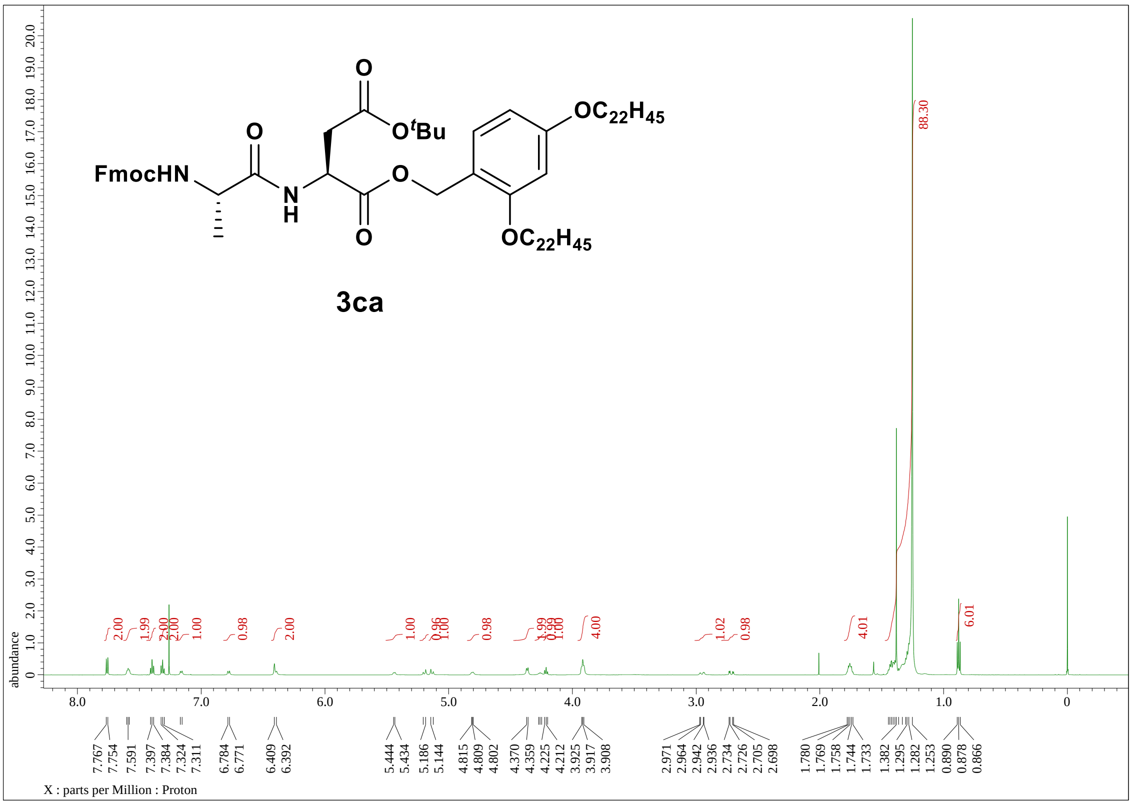
FmocHN
O
N
H
O
O
O^tBu
O
OC22H45
OC22H45
3ca
88.30
2.00
1.99
2.00
2.00
1.00
0.98
2.00
1.00
0.96
1.00
0.98
1.99
0.99
1.00
4.00
1.02
0.98
4.01
6.01
abundance
7.767
7.754
7.591
7.397
7.384
7.324
7.311
6.784
6.771
6.409
6.392
5.444
5.434
5.186
5.144
4.815
4.809
4.802
4.370
4.359
4.225
4.212
3.925
3.917
3.908
2.971
2.964
2.942
2.936
2.734
2.726
2.705
2.698
1.780
1.769
1.758
1.744
1.733
1.382
1.295
1.282
1.253
0.890
0.878
0.866
X : parts per Million : Proton

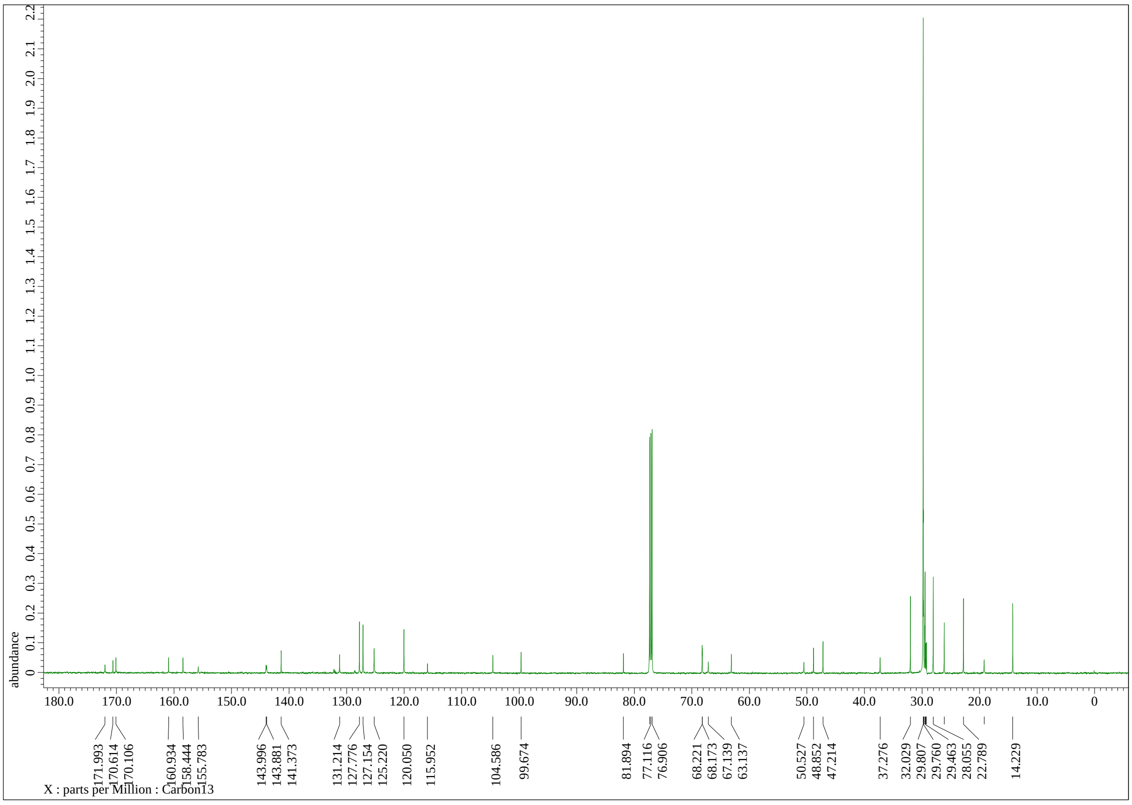
abundance
171.993
170.614
170.106
160.934
158.444
155.783
143.996
143.881
141.373
131.214
127.776
127.154
125.220
120.050
115.952
104.586
99.674
81.894
77.116
76.906
68.221
68.173
67.139
63.137
50.527
48.852
47.214
37.276
32.029
29.807
29.760
29.463
28.055
22.789
14.229
X : parts per Million : Carbon13

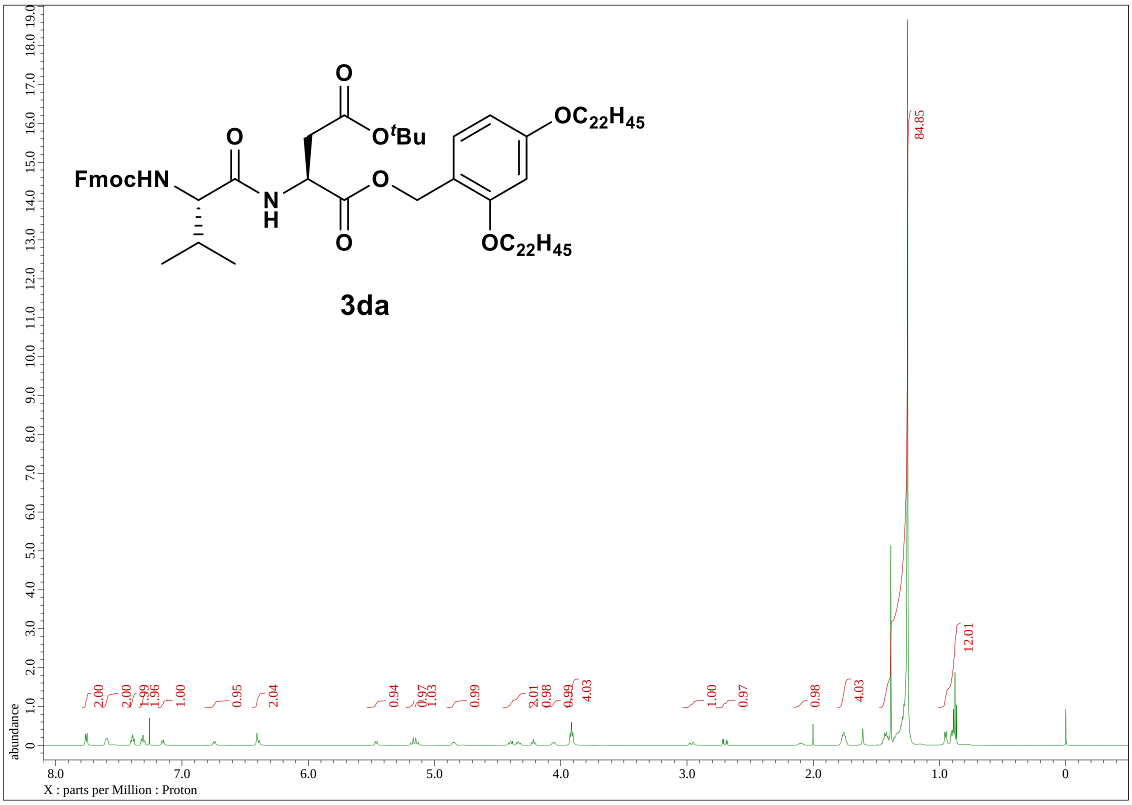
FmocHN
O
N
H
O
O^tBu
O
O
OC22H45
OC22H45
3da
84.85
12.01
2.00 2.00 1.99 1.96 1.00 0.95 2.04 0.94 0.97 1.03 0.99 2.01 0.98 0.99 4.03 1.00 0.97 0.98 4.03
abundance
X : parts per Million : Proton

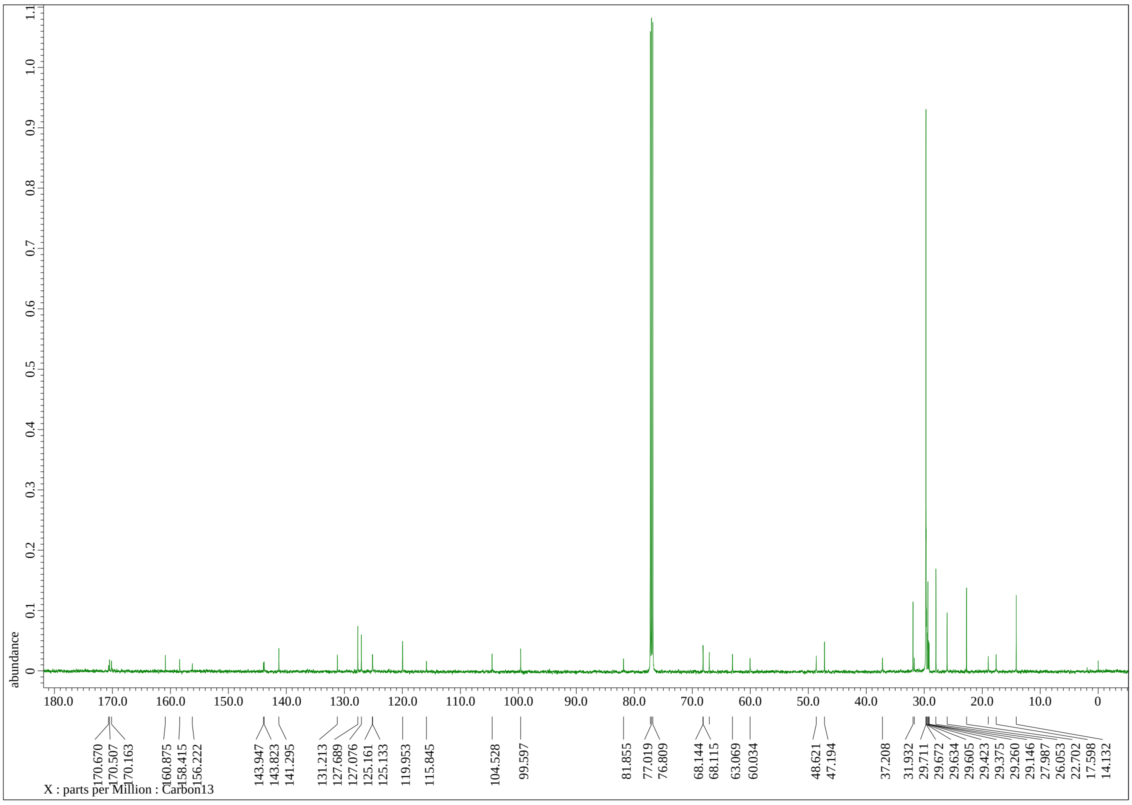
abundance
170.670
170.507
170.163
160.875
158.415
156.222
143.947
143.823
141.295
131.213
127.689
127.076
125.161
125.133
119.953
115.845
104.528
99.597
81.855
77.019
76.809
68.144
68.115
63.069
60.034
48.621
47.194
37.208
31.932
29.711
29.672
29.634
29.605
29.423
29.375
29.260
29.146
27.987
26.053
22.702
17.598
14.132
X : parts per Million : Carbon13

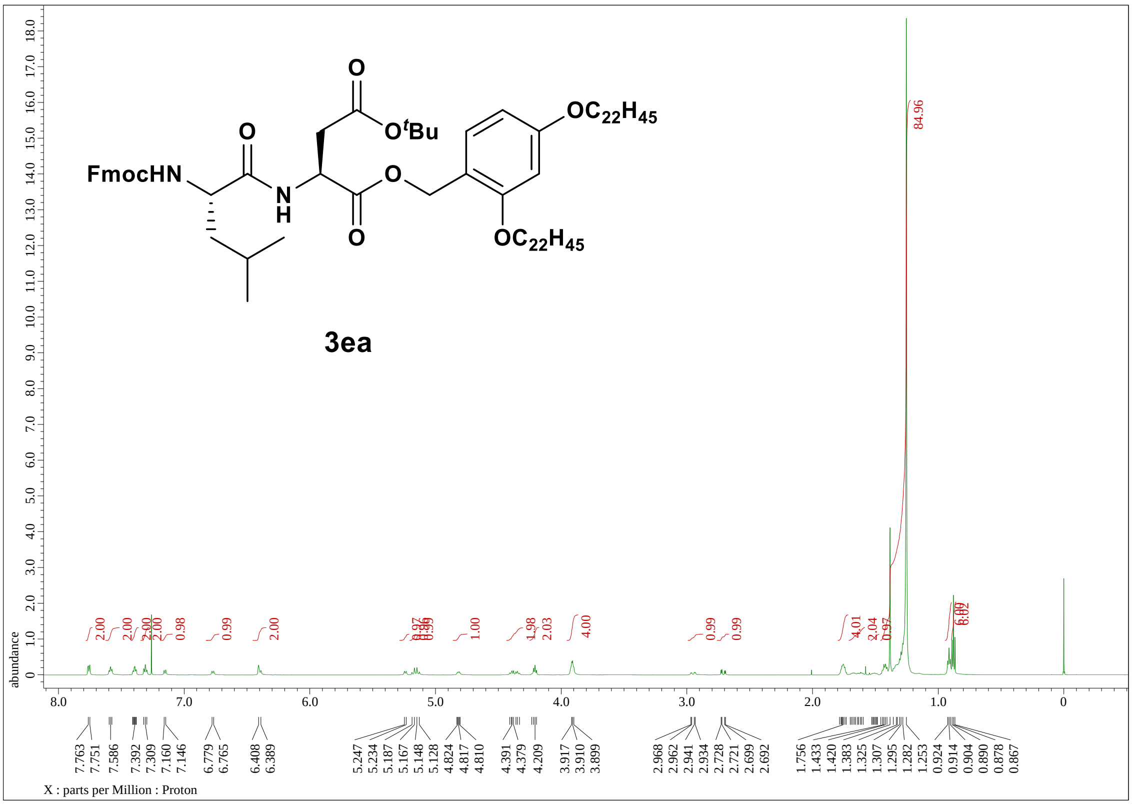
FmocHN
N
H
O
O
O
O
O
O^tBu
OC22H45
OC22H45
3ea
X : parts per Million : Proton
abundance

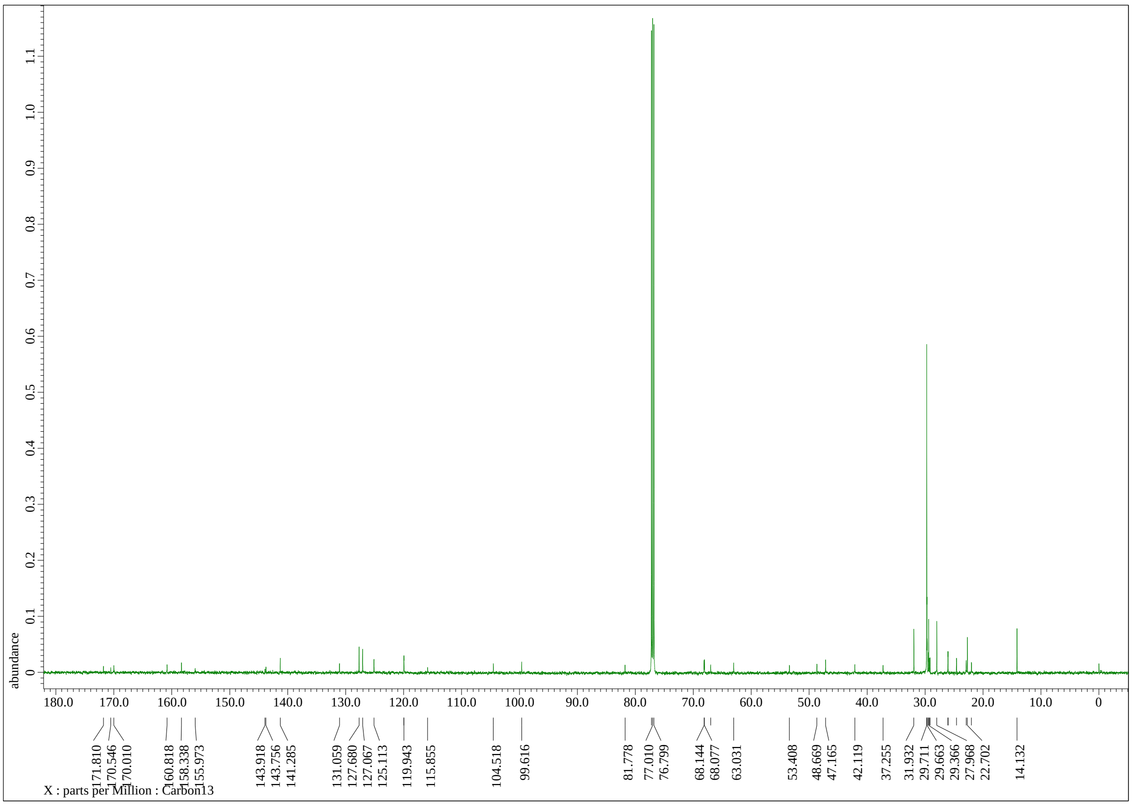
X : parts per Million : Carbon13
abundance

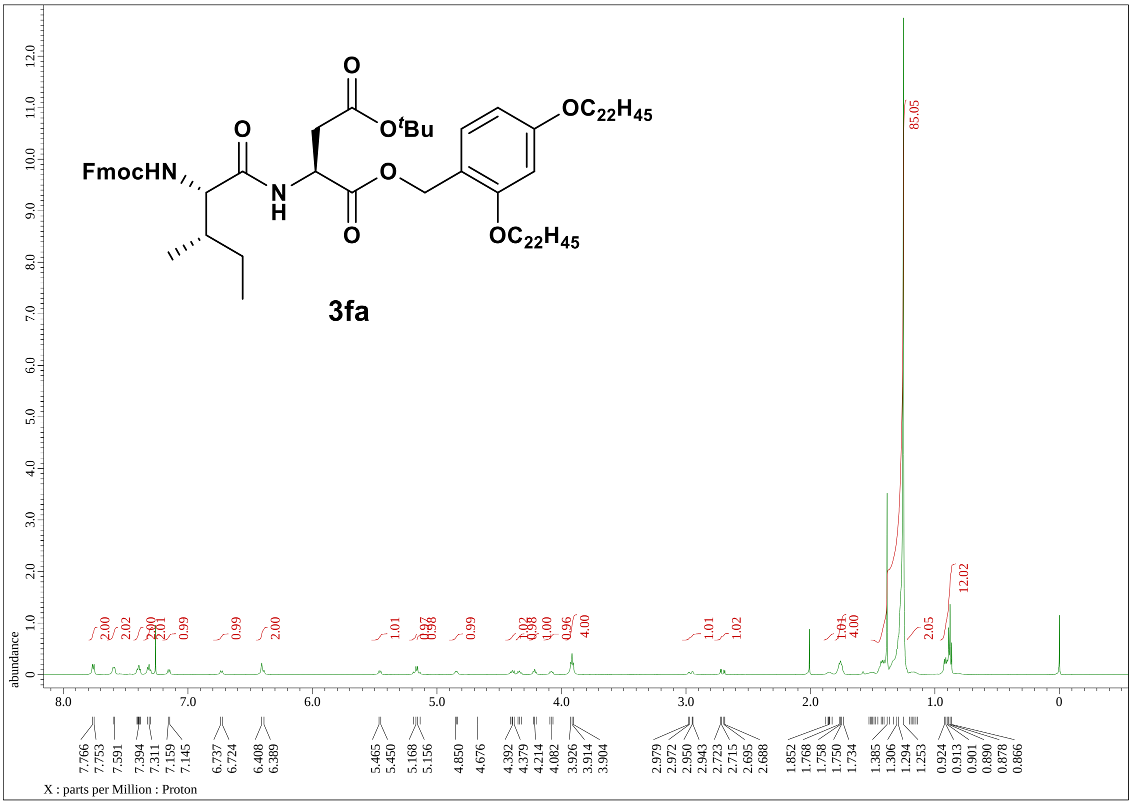
FmocHN
O
N
H
O
O
O^{t}Bu
O
$OC_{22}H_{45}$
$OC_{22}H_{45}$
3fa
85.05
12.02
2.00 2.02 2.00 2.01 0.99 0.99 2.00 1.01 0.97 0.98 0.99 1.02 0.98 1.00 0.96 4.00 1.01 1.02 1.01 4.00 2.05
7.766 7.753 7.591 7.394 7.311 7.159 7.145 6.737 6.724 6.408 6.389 5.465 5.450 5.168 5.156 4.850 4.676 4.392 4.379 4.214 4.082 3.926 3.914 3.904 2.979 2.972 2.950 2.943 2.723 2.715 2.695 2.688 1.852 1.768 1.758 1.750 1.734 1.385 1.306 1.294 1.253 0.924 0.913 0.901 0.890 0.878 0.866
abundance
X : parts per Million : Proton

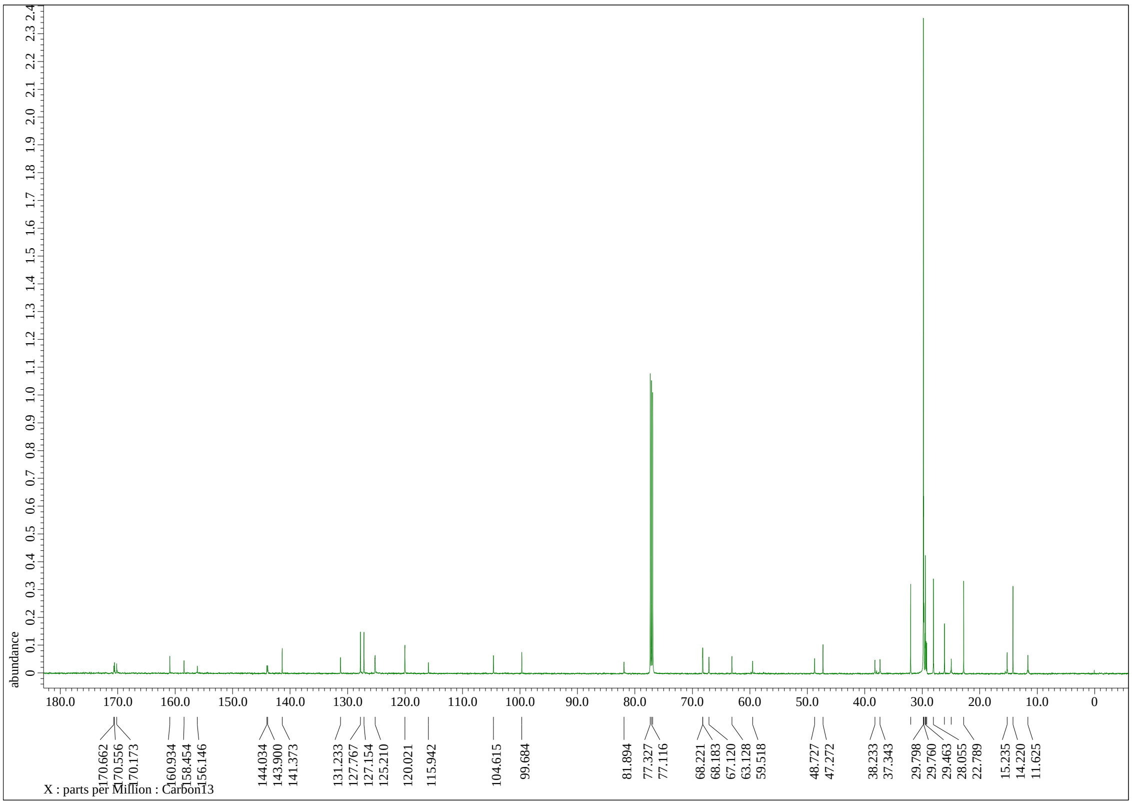
170.662 170.556 170.173 160.934 158.454 156.146 144.034 143.900 141.373 131.233 127.767 127.154 125.210 120.021 115.942 104.615 99.684 81.894 77.327 77.116 68.221 68.183 67.120 63.128 59.518 48.727 47.272 38.233 37.343 29.798 29.760 29.463 28.055 22.789 15.235 14.220 11.625
abundance
X : parts per Million : Carbon13

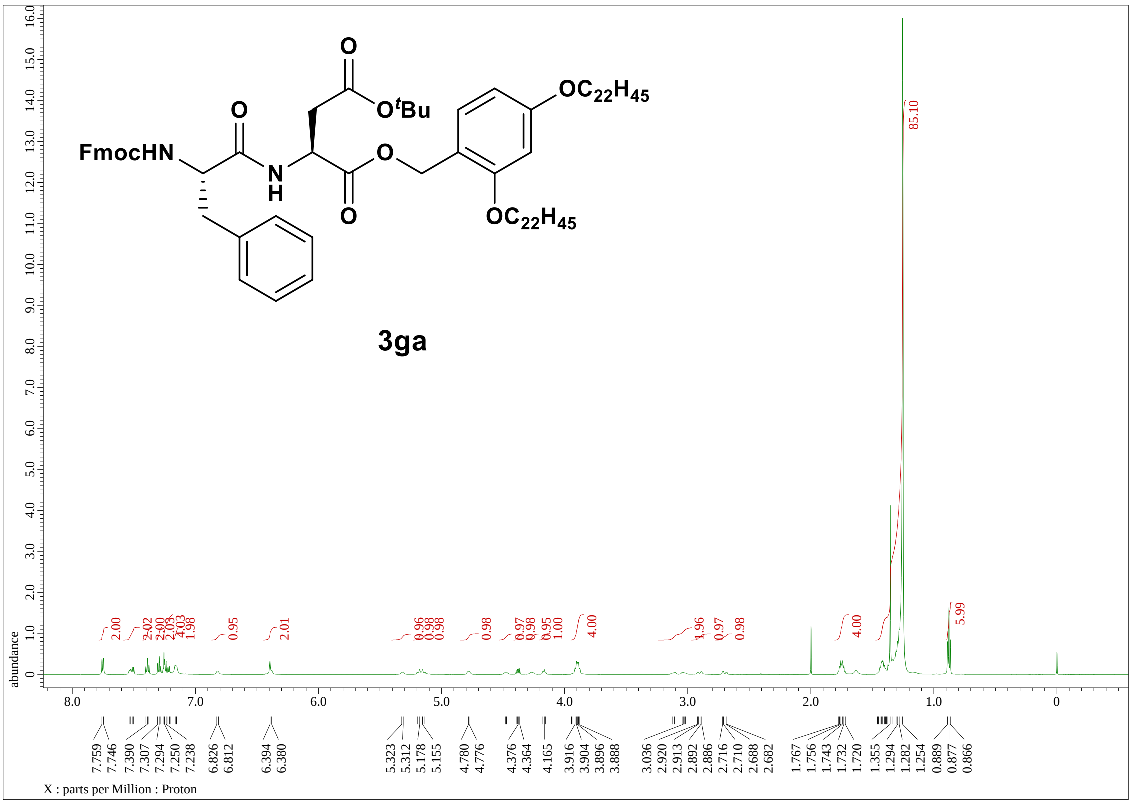
FmocHN
O
N
H
O
O
O^tBu
O
OC22H45
OC22H45
3ga
abundance
X : parts per Million : Proton
2.00
2.02
2.00
2.03
4.03
1.98
0.95
2.01
0.96
0.98
0.98
0.98
0.98
0.97
0.98
0.95
1.00
4.00
1.96
0.97
0.98
4.00
85.10
5.99
7.759
7.746
7.390
7.307
7.294
7.250
7.238
6.826
6.812
6.394
6.380
5.323
5.312
5.178
5.155
4.780
4.776
4.376
4.364
4.165
3.916
3.904
3.896
3.888
3.036
2.920
2.913
2.892
2.886
2.716
2.710
2.688
2.682
1.767
1.756
1.743
1.732
1.720
1.355
1.294
1.282
1.254
0.889
0.877
0.866

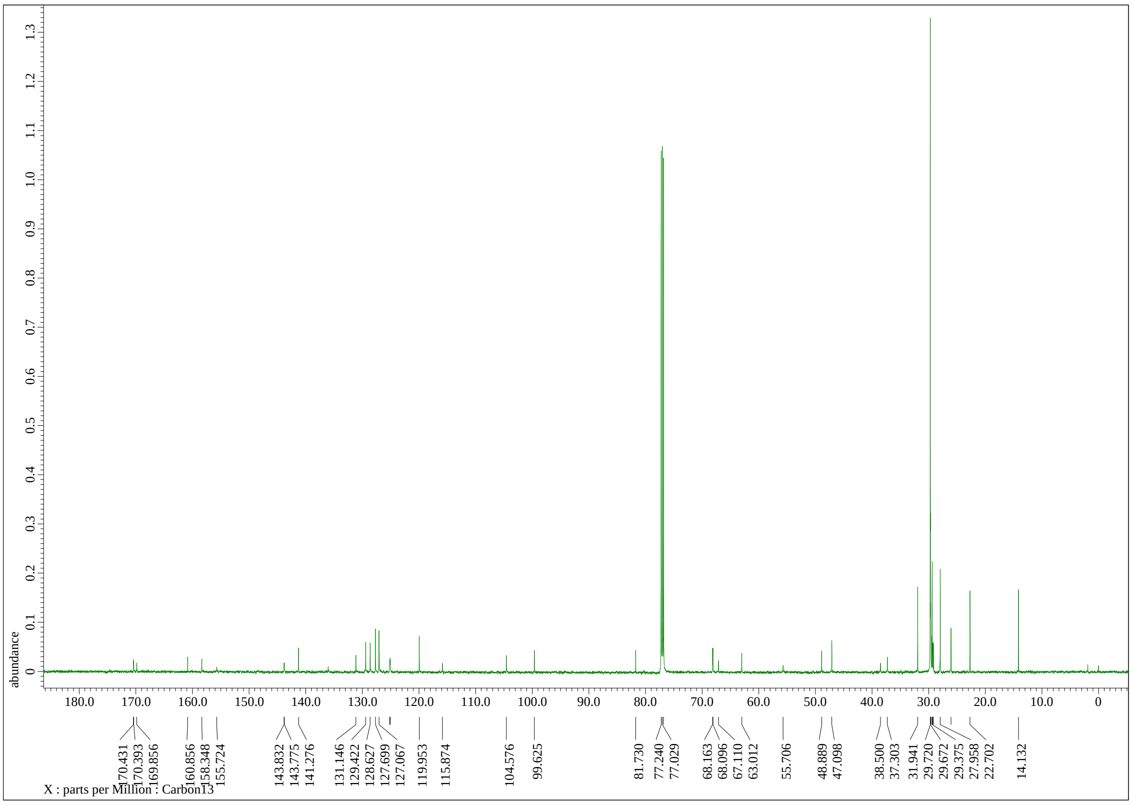
abundance
X : parts per Million : Carbon13
170.431
170.393
169.856
160.856
158.348
155.724
143.832
143.775
141.276
131.146
129.422
128.627
127.699
127.067
119.953
115.874
104.576
99.625
81.730
77.240
77.029
68.163
68.096
67.110
63.012
55.706
48.889
47.098
38.500
37.303
31.941
29.720
29.672
29.375
27.958
22.702
14.132

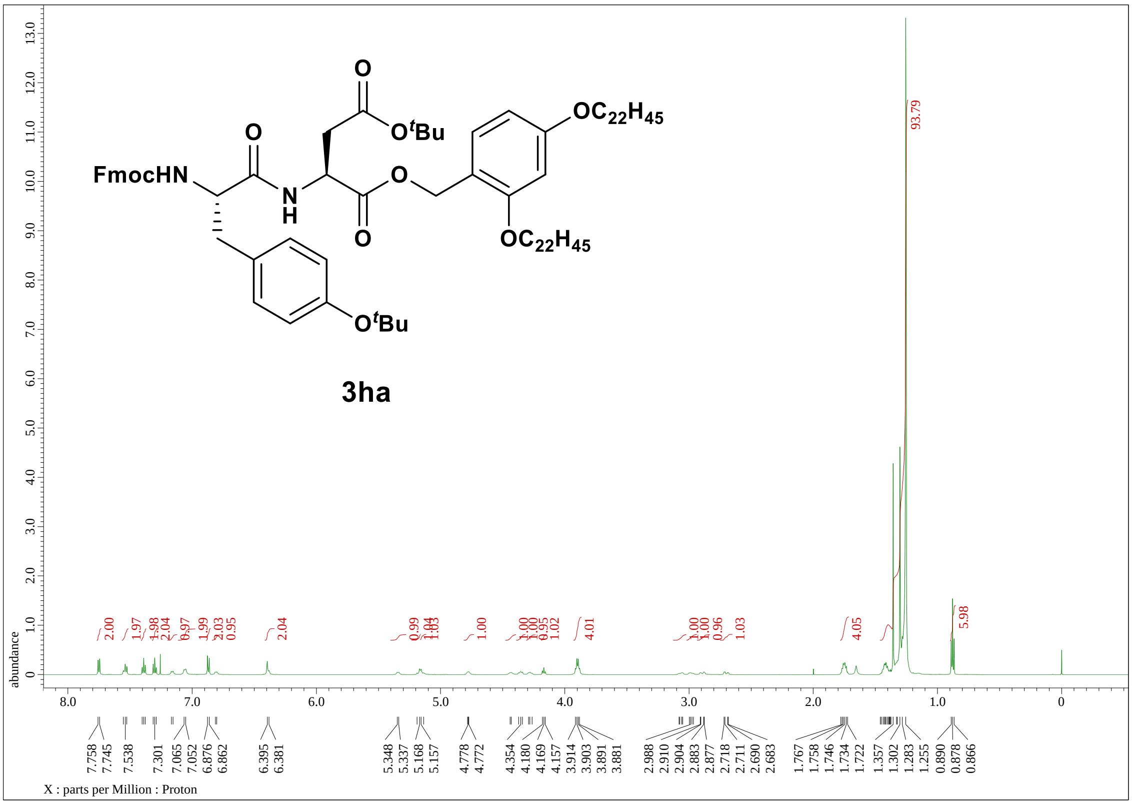
FmocHN
O
N
H
O
O^tBu
O
O
OC22H45
OC22H45
O^tBu
3ha
93.79
abundance
X : parts per Million : Proton

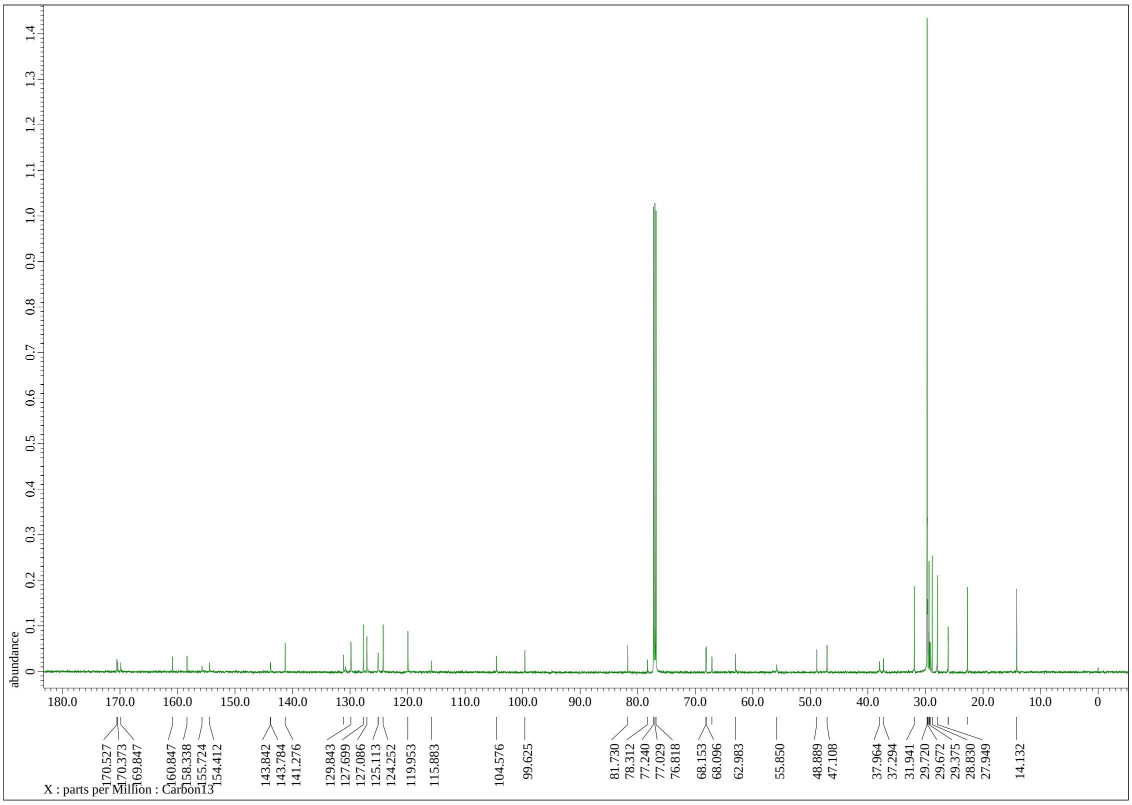
abundance
X : parts per Million : Carbon13

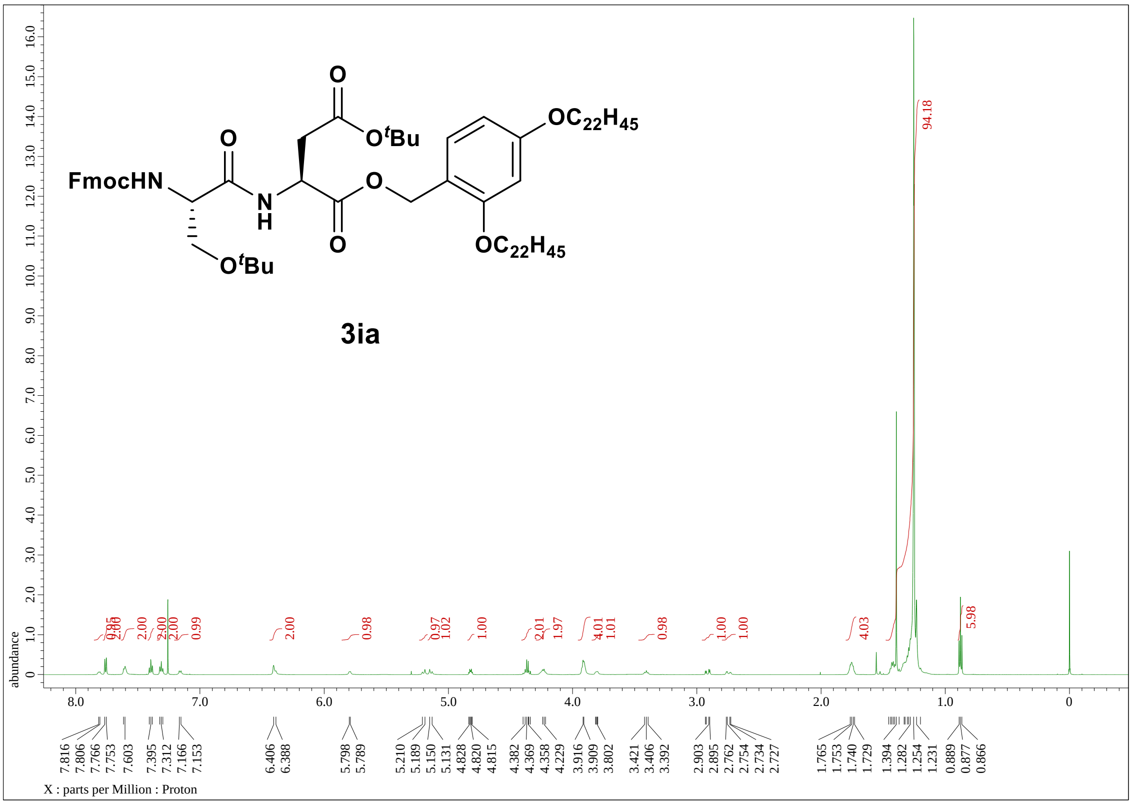

FmocHN
O
N
H
O
O
O^{t}Bu
O
O^{t}Bu
OC$_{22}$H$_{45}$
OC$_{22}$H$_{45}$
3ia
abundance
X : parts per Million : Proton
94.18
5.98
4.03
7.816
7.806
7.766
7.753
7.603
7.395
7.312
7.166
7.153
6.406
6.388
5.798
5.789
5.210
5.189
5.150
5.131
4.828
4.820
4.815
4.382
4.369
4.358
4.229
3.916
3.909
3.802
3.421
3.406
3.392
2.903
2.895
2.762
2.754
2.734
2.727
1.765
1.753
1.740
1.729
1.394
1.282
1.254
1.231
0.889
0.877
0.866

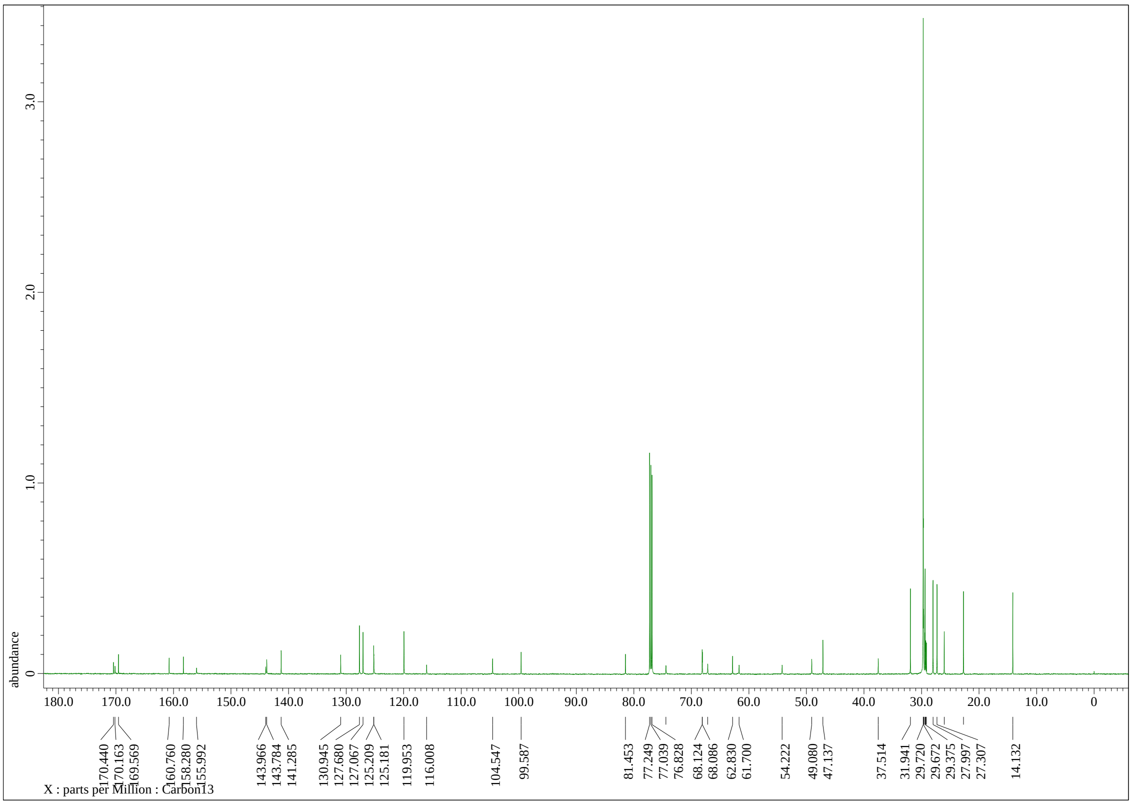

abundance
X : parts per Million : Carbon13
170.440
170.163
169.569
160.760
158.280
155.992
143.966
143.784
141.285
130.945
127.680
127.067
125.209
125.181
119.953
116.008
104.547
99.587
81.453
77.249
77.039
76.828
68.124
68.086
62.830
61.700
54.222
49.080
47.137
37.514
31.941
29.720
29.672
29.375
27.997
27.307
14.132

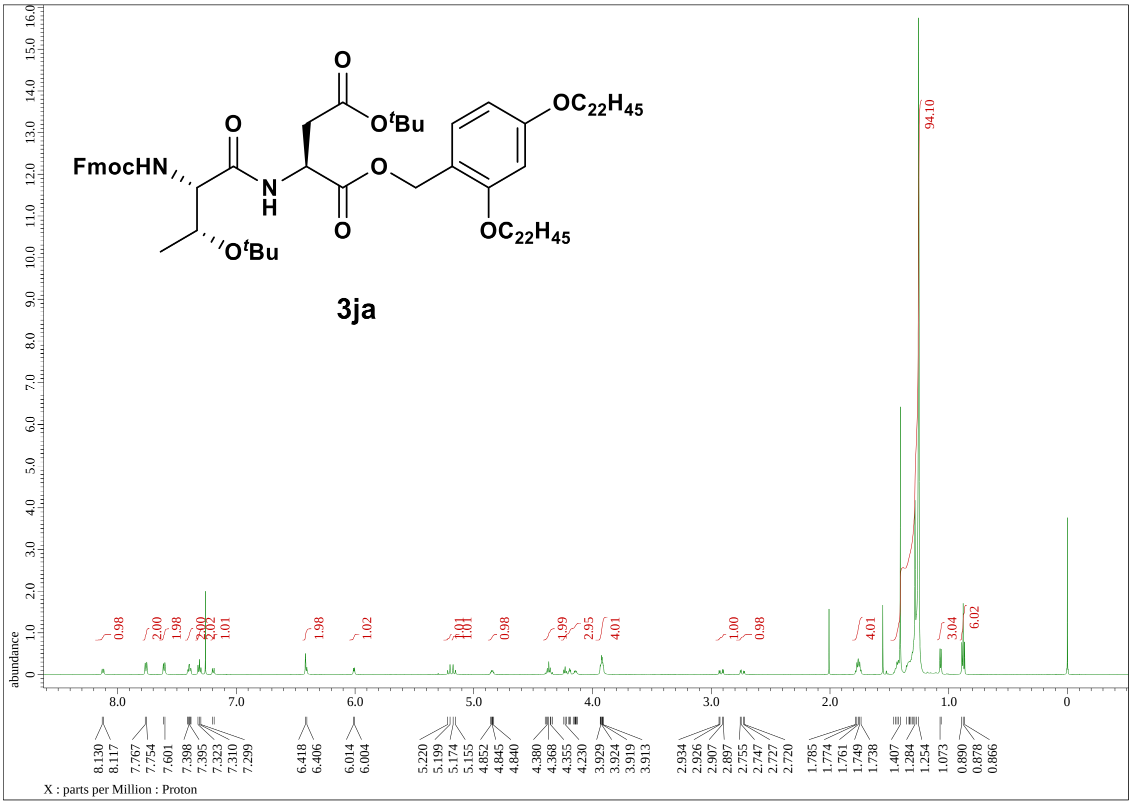
FmocHN
O
N
H
O
O^{t}Bu
O
O^{t}Bu
O
O
OC$_{22}$H$_{45}$
OC$_{22}$H$_{45}$
3ja
abundance
X : parts per Million : Proton
0.98
2.00
1.98
2.00
2.02
1.01
1.98
1.02
1.01
1.01
0.98
1.99
2.95
4.01
1.00
0.98
4.01
94.10
3.04
6.02
8.130
8.117
7.767
7.754
7.601
7.398
7.395
7.323
7.310
7.299
6.418
6.406
6.014
6.004
5.220
5.199
5.174
5.155
4.852
4.845
4.840
4.380
4.368
4.355
4.230
3.929
3.924
3.919
3.913
2.934
2.926
2.907
2.897
2.755
2.747
2.727
2.720
1.785
1.774
1.761
1.749
1.738
1.407
1.284
1.254
1.073
0.890
0.878
0.866

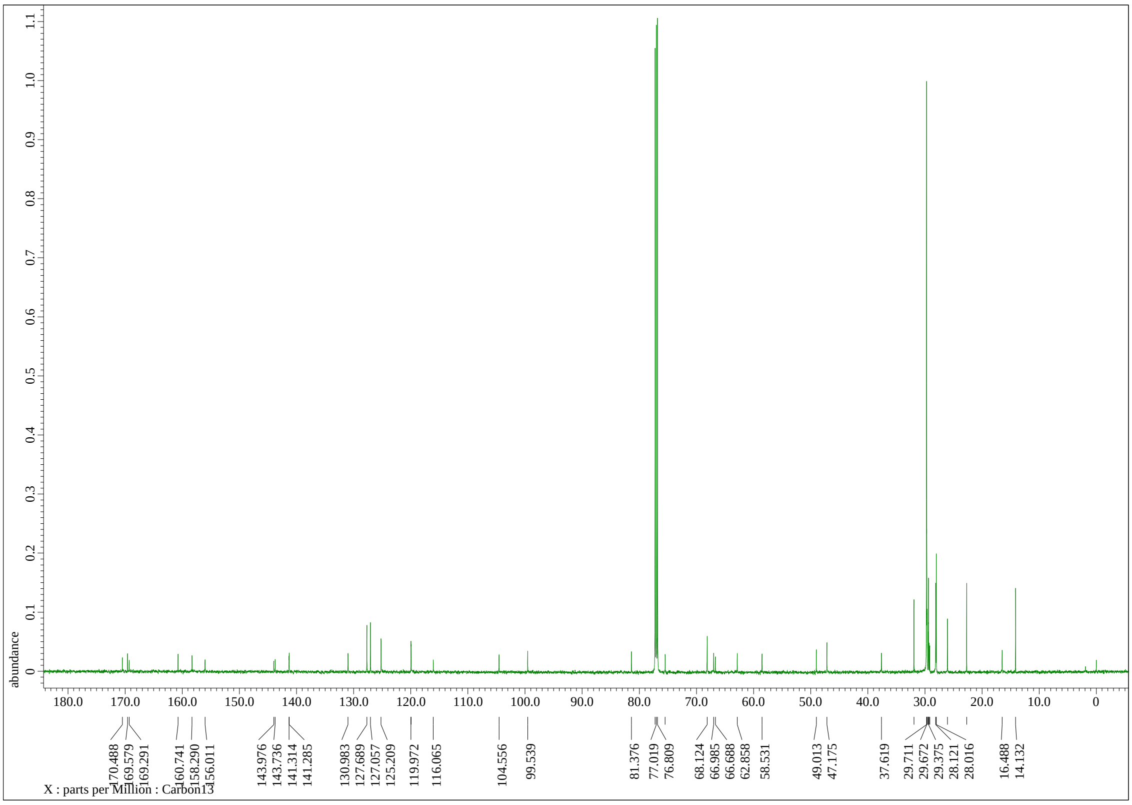
abundance
X : parts per Million : Carbon13
170.488
169.579
169.291
160.741
158.290
156.011
143.976
143.736
141.314
141.285
130.983
127.689
127.057
125.209
119.972
116.065
104.556
99.539
81.376
77.019
76.809
68.124
66.985
66.688
62.858
58.531
49.013
47.175
37.619
29.711
29.672
29.375
28.121
28.016
16.488
14.132

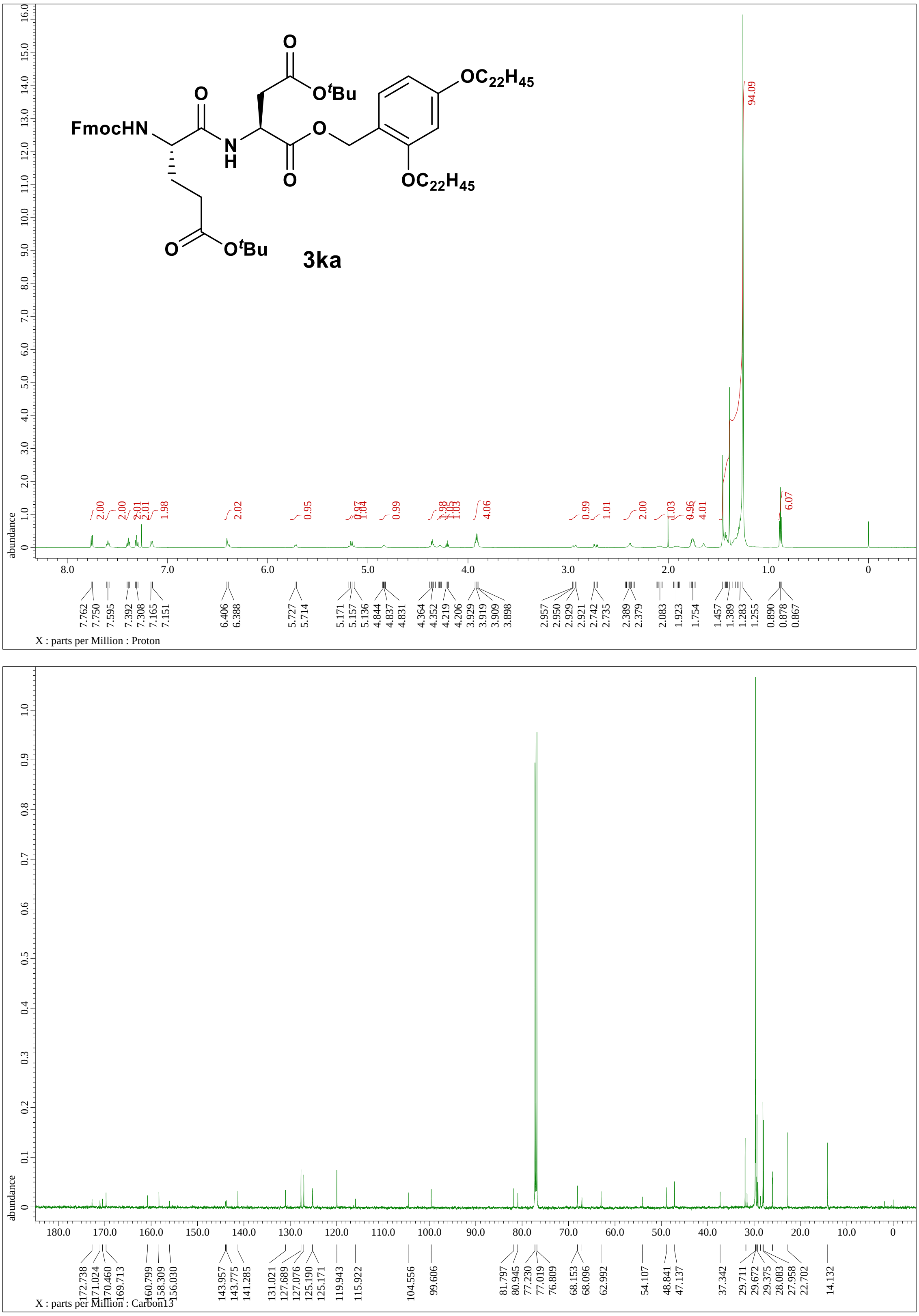
FmocHN
O
N
H
O
O
O
O^tBu
OC22H45
OC22H45
O
O^tBu
3ka
X : parts per Million : Proton
abundance
X : parts per Million : Carbon13

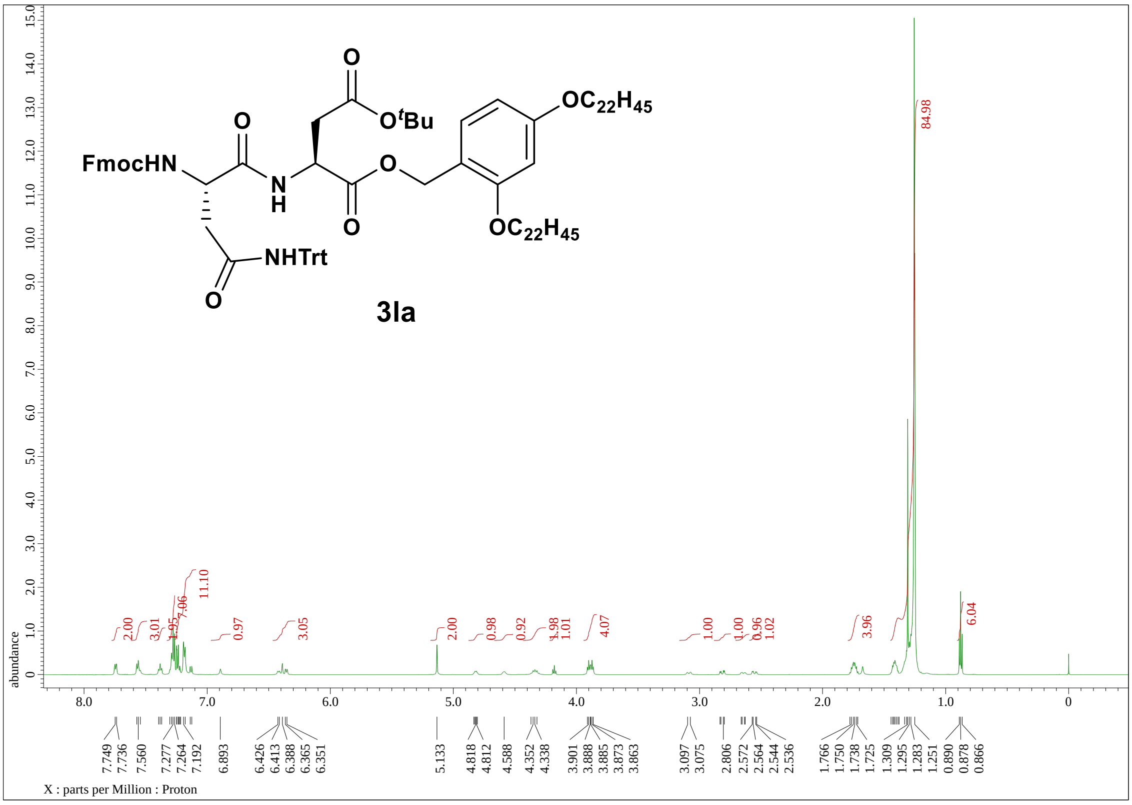
FmocHN
NHTrt
O^tBu
OC22H45
OC22H45
3la
84.98
6.04
3.96
X : parts per Million : Proton
abundance

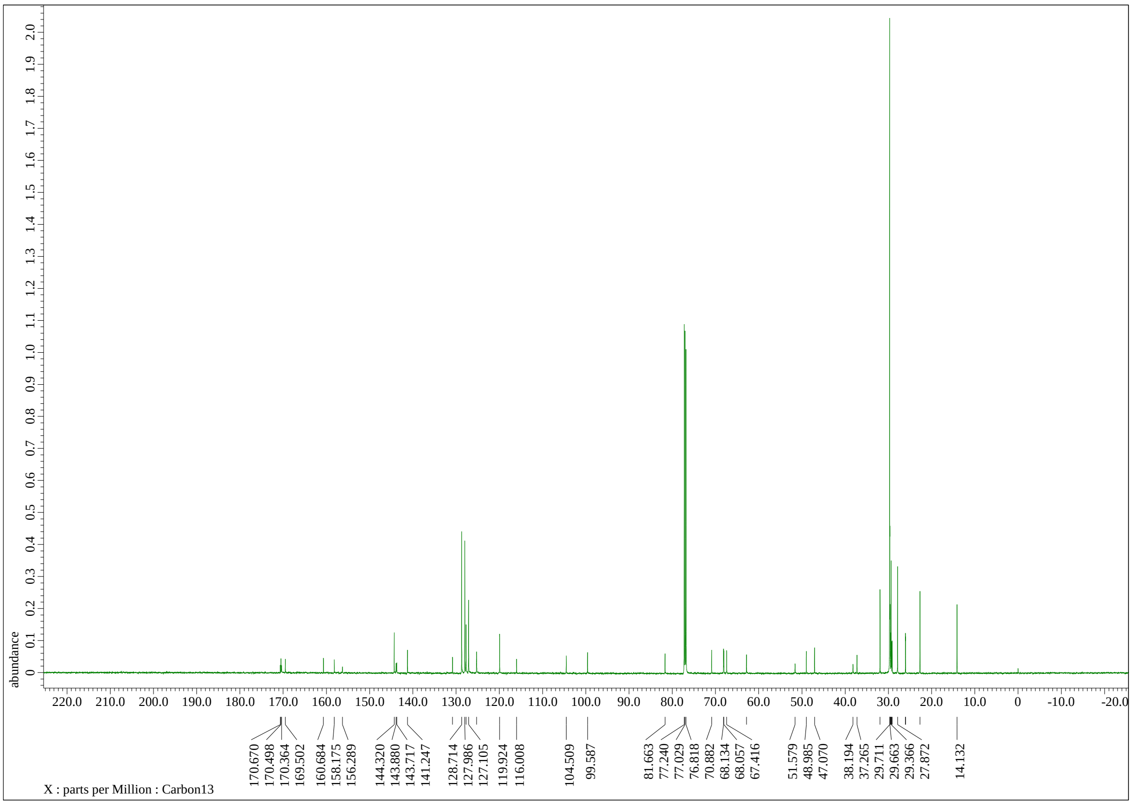
X : parts per Million : Carbon13
abundance

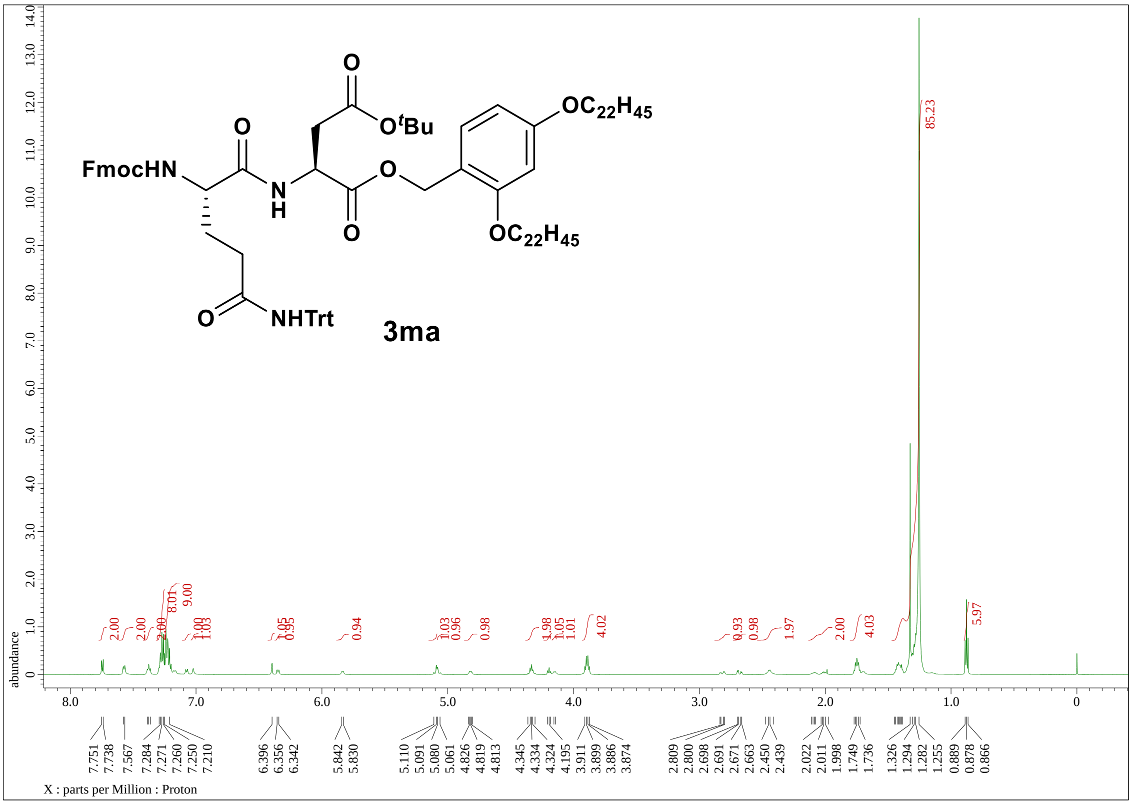

FmocHN
NHTrt
O'Bu
OC22H45
OC22H45
3ma
X : parts per Million : Proton

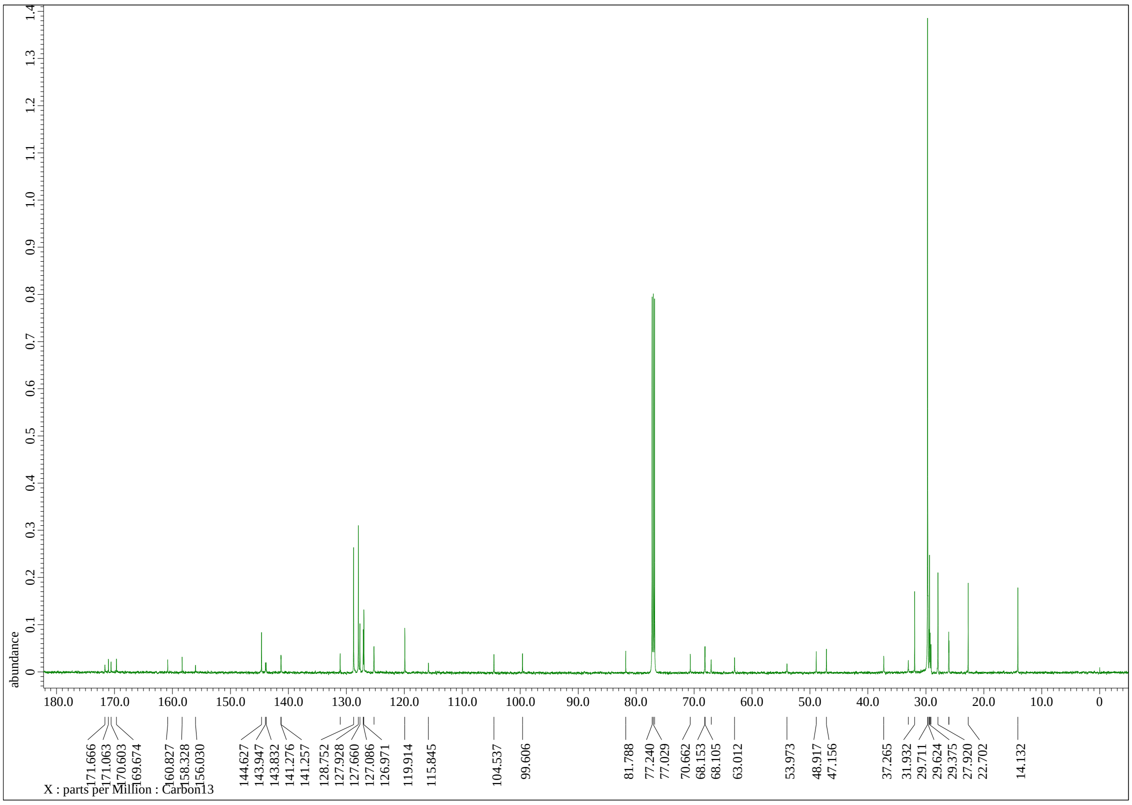

X : parts per Million : Carbon13

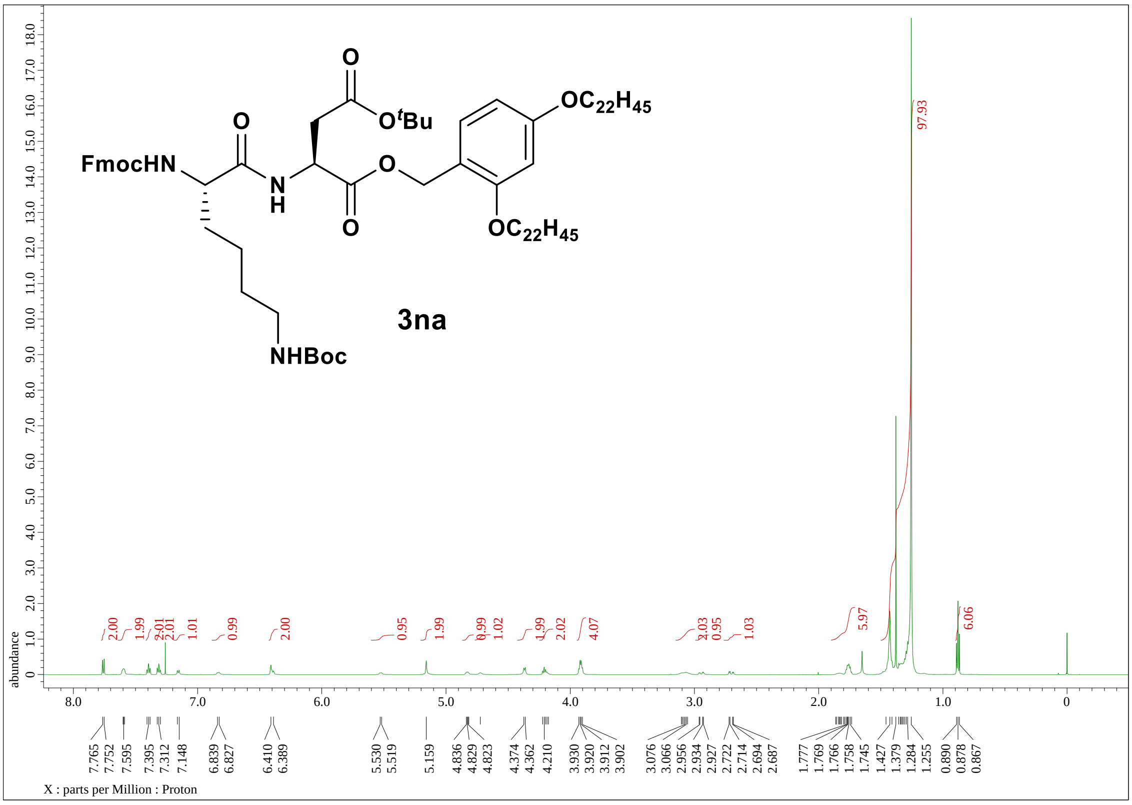
FmocHN
O
N
H
O
O
O^tBu
O
OC22H45
OC22H45
NHBoc
3na
abundance
2.00
1.99
2.01
2.01
1.01
0.99
2.00
0.95
1.99
0.99
1.02
1.99
2.02
4.07
2.03
0.95
1.03
5.97
97.93
6.06
7.765
7.752
7.595
7.395
7.312
7.148
6.839
6.827
6.410
6.389
5.530
5.519
5.159
4.836
4.829
4.823
4.374
4.362
4.210
3.930
3.920
3.912
3.902
3.076
3.066
2.956
2.934
2.927
2.722
2.714
2.694
2.687
1.777
1.769
1.766
1.758
1.745
1.427
1.379
1.284
1.255
0.890
0.878
0.867
X : parts per Million : Proton

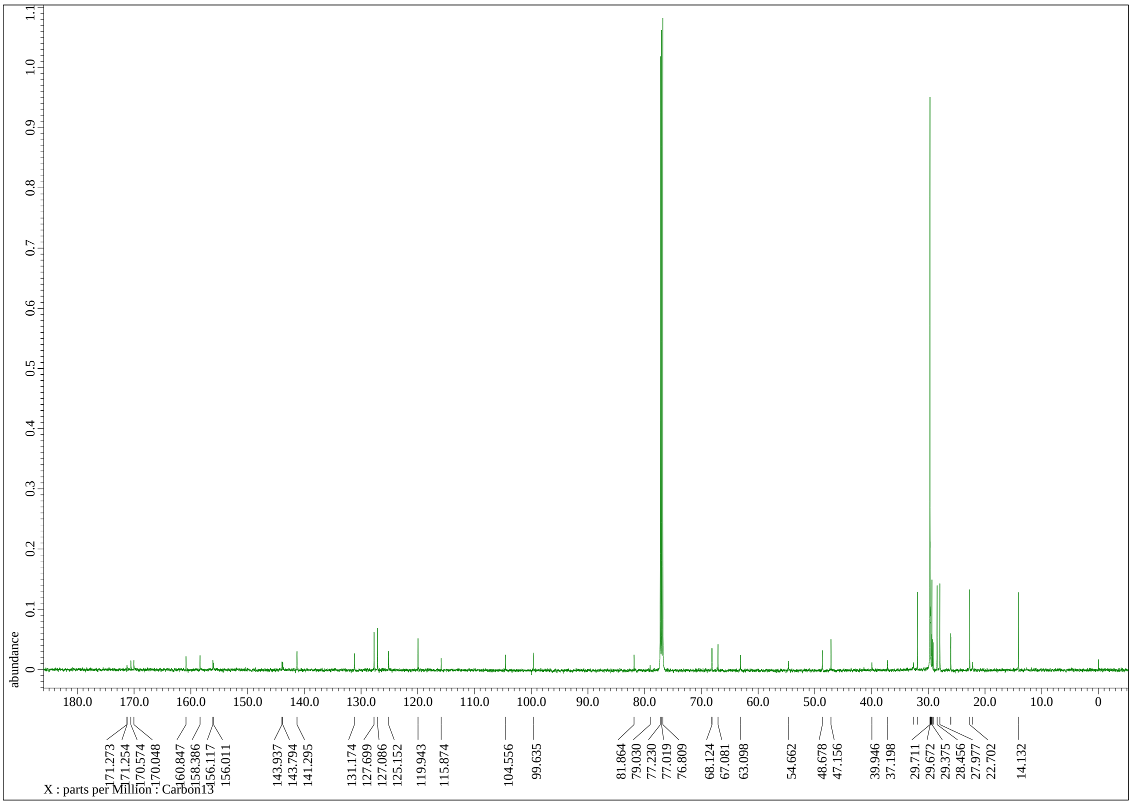
abundance
171.273
171.254
170.574
170.048
160.847
158.386
156.117
156.011
143.937
143.794
141.295
131.174
127.699
127.086
125.152
119.943
115.874
104.556
99.635
81.864
79.030
77.230
77.019
76.809
68.124
67.081
63.098
54.662
48.678
47.156
39.946
37.198
29.711
29.672
29.375
28.456
27.977
22.702
14.132
X : parts per Million : Carbon13

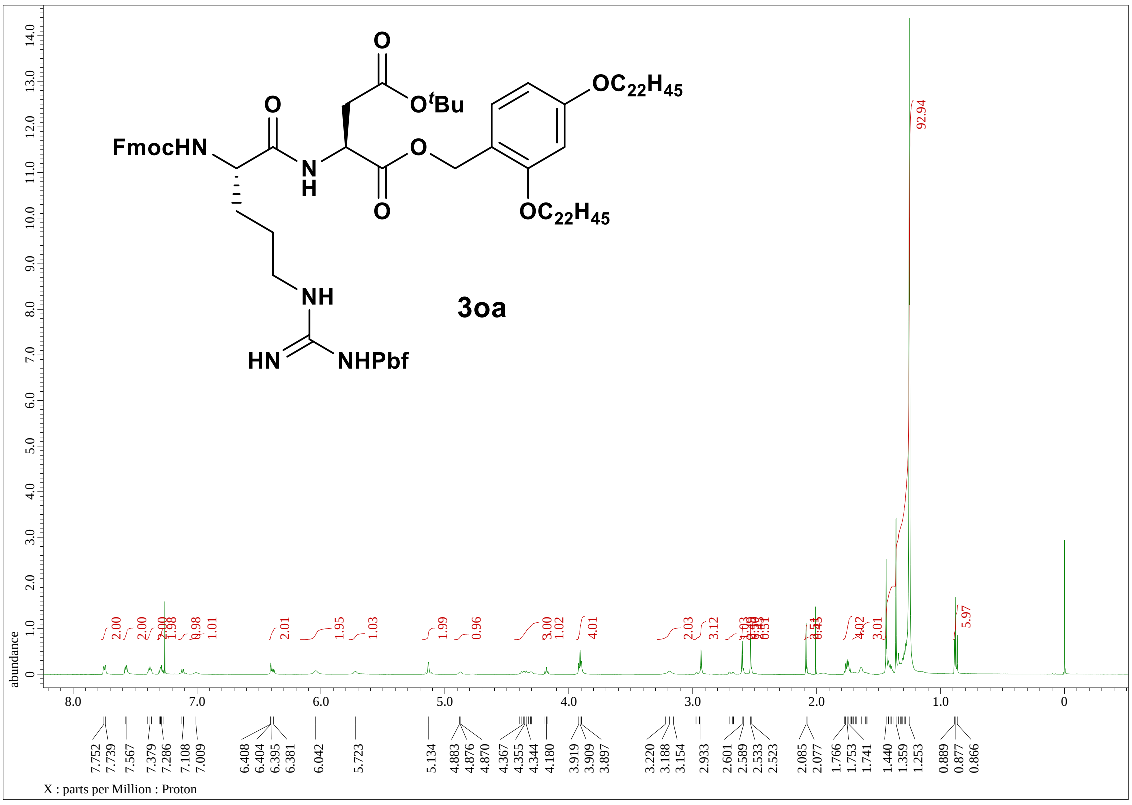
FmocHN
O
N
H
O
O^tBu
O
O
OC22H45
OC22H45
NH
HN
NHPbf
3oa
abundance
92.94
2.00 2.00 2.00 1.98 0.98 1.01 2.01 1.95 1.03 1.99 0.96 3.00 1.02 4.01 2.03 3.12 4.02 3.01 5.97
7.752 7.739 7.567 7.379 7.286 7.108 7.009 6.408 6.404 6.395 6.381 6.042 5.723 5.134 4.883 4.876 4.870 4.367 4.355 4.344 4.180 3.919 3.909 3.897 3.220 3.188 3.154 2.933 2.601 2.589 2.533 2.523 2.085 2.077 1.766 1.753 1.741 1.440 1.359 1.253 0.889 0.877 0.866
X : parts per Million : Proton

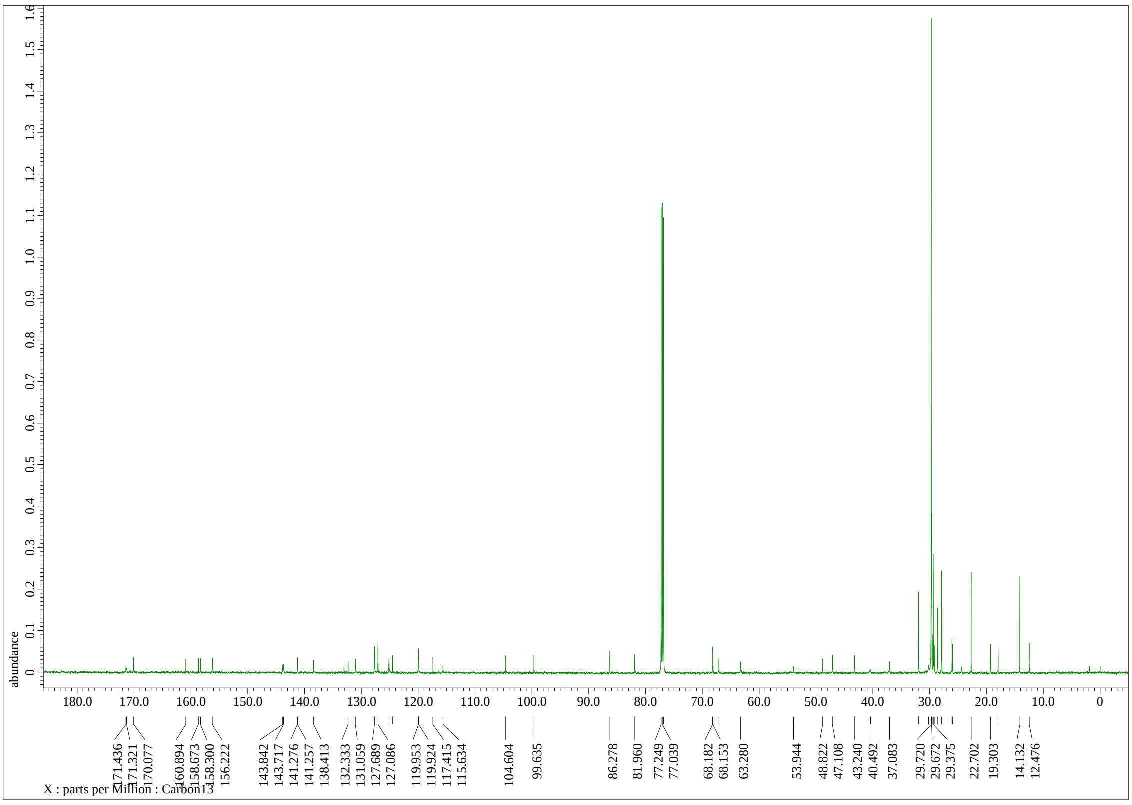
abundance
171.436 171.321 170.077 160.894 158.673 158.300 156.222 143.842 143.717 141.276 141.257 138.413 132.333 131.059 127.689 127.086 119.953 119.924 117.415 115.634 104.604 99.635 86.278 81.960 77.249 77.039 68.182 68.153 63.280 53.944 48.822 47.108 43.240 40.492 37.083 29.720 29.672 29.375 22.702 19.303 14.132 12.476
X : parts per Million : Carbon13

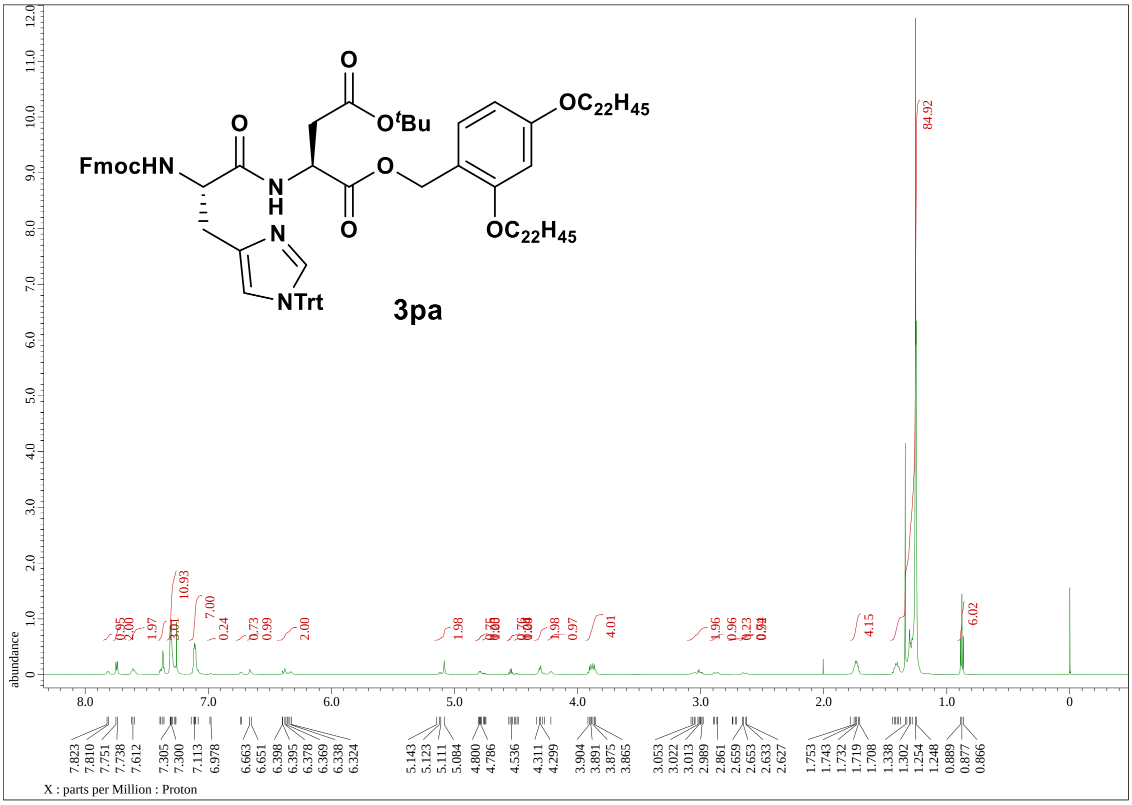
FmocHN
O
N
H
O
O^{t}Bu
O
O
OC$_{22}$H$_{45}$
OC$_{22}$H$_{45}$
N
NTrt
3pa
abundance
0.95
2.00
1.97
3.01
10.93
7.00
0.24
0.73
0.99
2.00
1.98
1.98
0.97
4.01
1.96
0.96
0.23
4.15
84.92
6.02
7.823
7.810
7.751
7.738
7.612
7.305
7.300
7.113
6.978
6.663
6.651
6.398
6.395
6.378
6.369
6.338
6.324
5.143
5.123
5.111
5.084
4.800
4.786
4.536
4.311
4.299
3.904
3.891
3.875
3.865
3.053
3.022
3.013
2.989
2.861
2.659
2.653
2.633
2.627
1.753
1.743
1.732
1.719
1.708
1.338
1.302
1.254
1.248
0.889
0.877
0.866
X : parts per Million : Proton

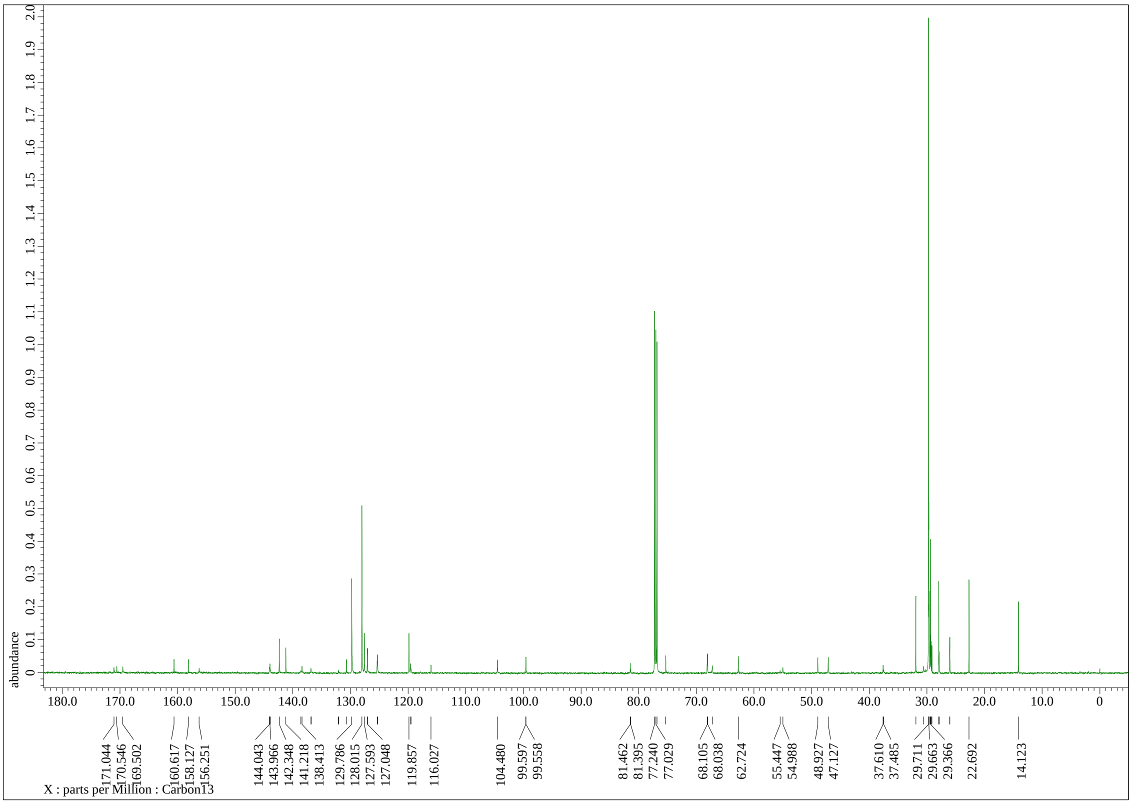
abundance
171.044
170.546
169.502
160.617
158.127
156.251
144.043
143.966
142.348
141.218
138.413
129.786
128.015
127.593
127.048
119.857
116.027
104.480
99.597
99.558
81.462
81.395
77.240
77.029
68.105
68.038
62.724
55.447
54.988
48.927
47.127
37.610
37.485
29.711
29.663
29.366
22.692
14.123
X : parts per Million : Carbon13

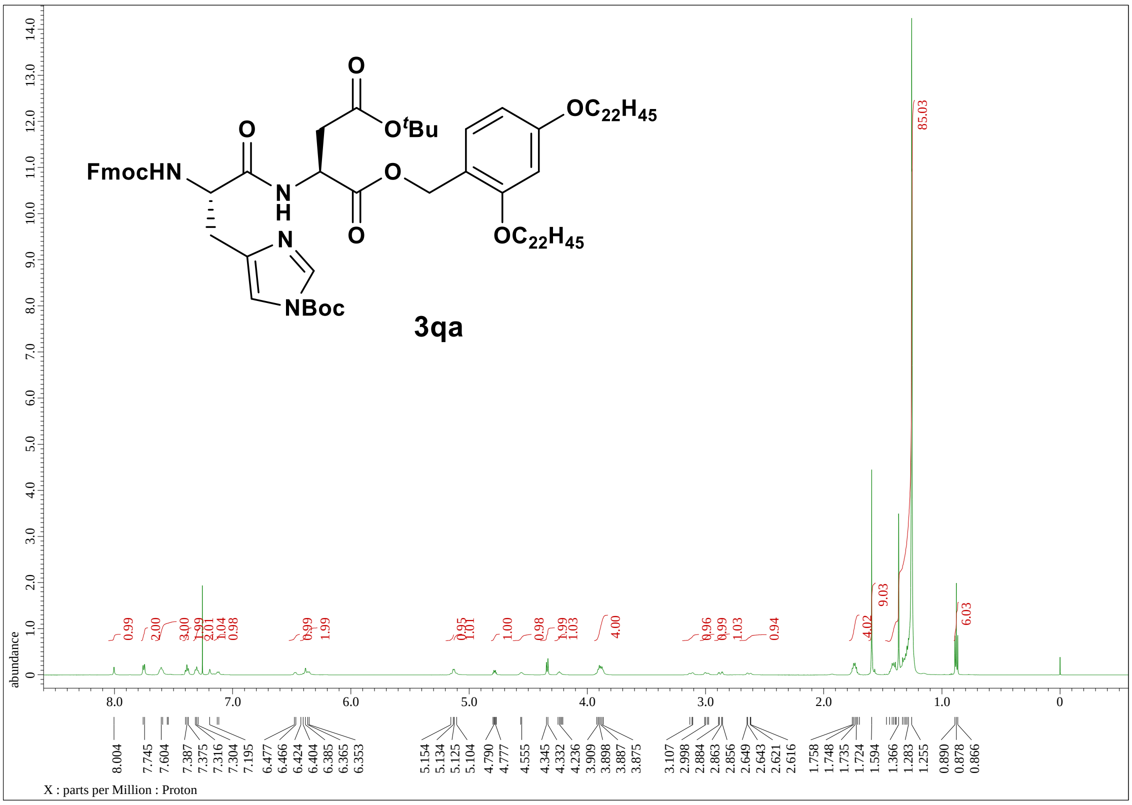
FmocHN
NBoc
O'Bu
$OC_{22}H_{45}$
$OC_{22}H_{45}$
3qa
X : parts per Million : Proton
abundance

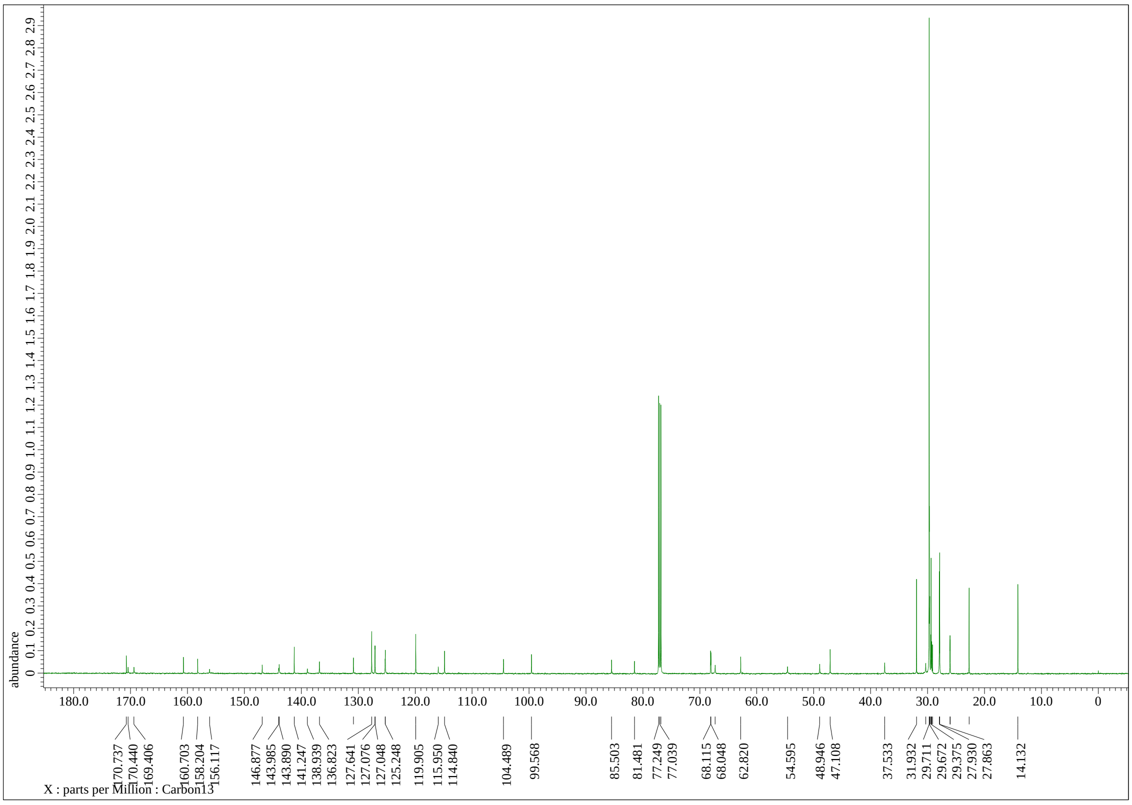
X : parts per Million : Carbon13
abundance

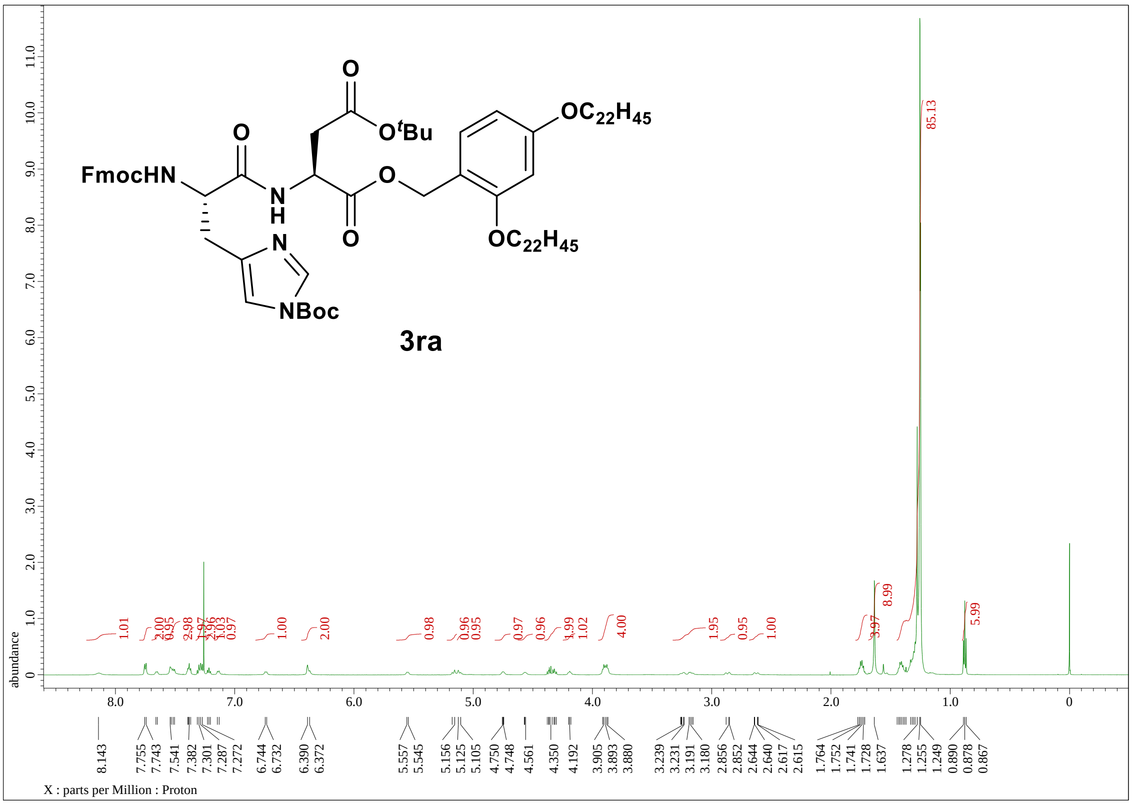
FmocHN
NBoc
O^{t}Bu
OC22H45
3ra
abundance
X : parts per Million : Proton

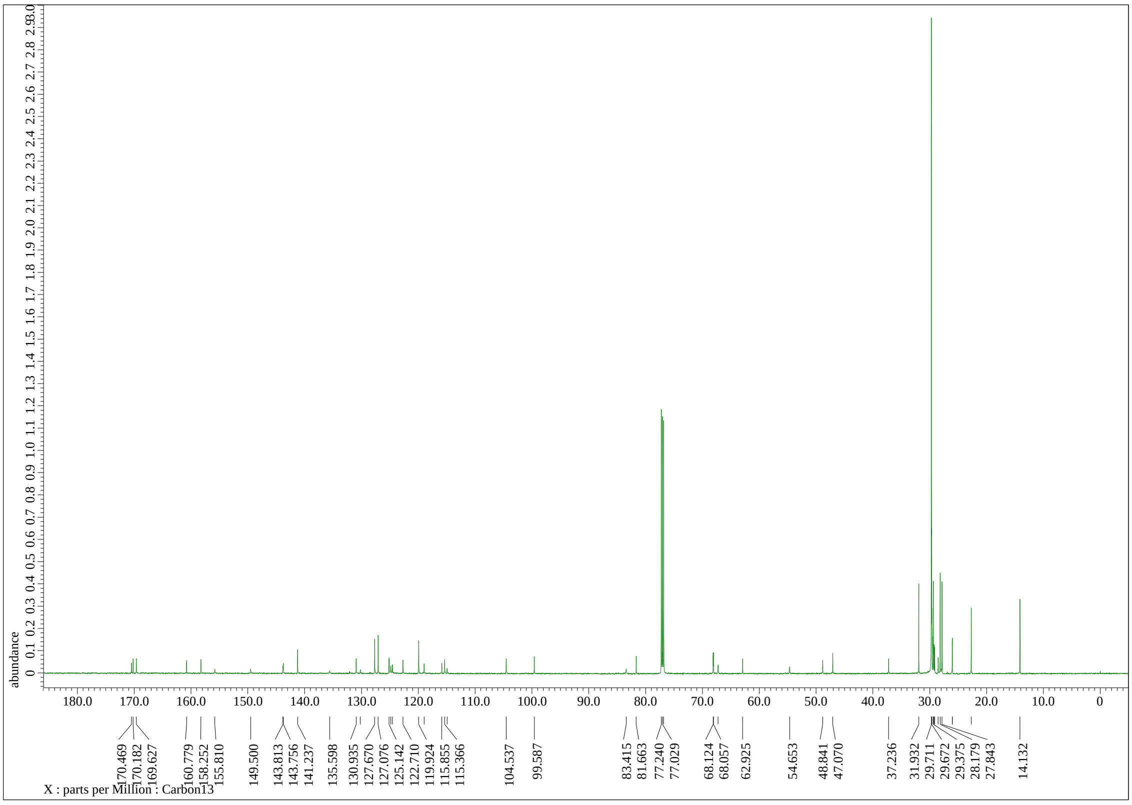
abundance
X : parts per Million : Carbon13

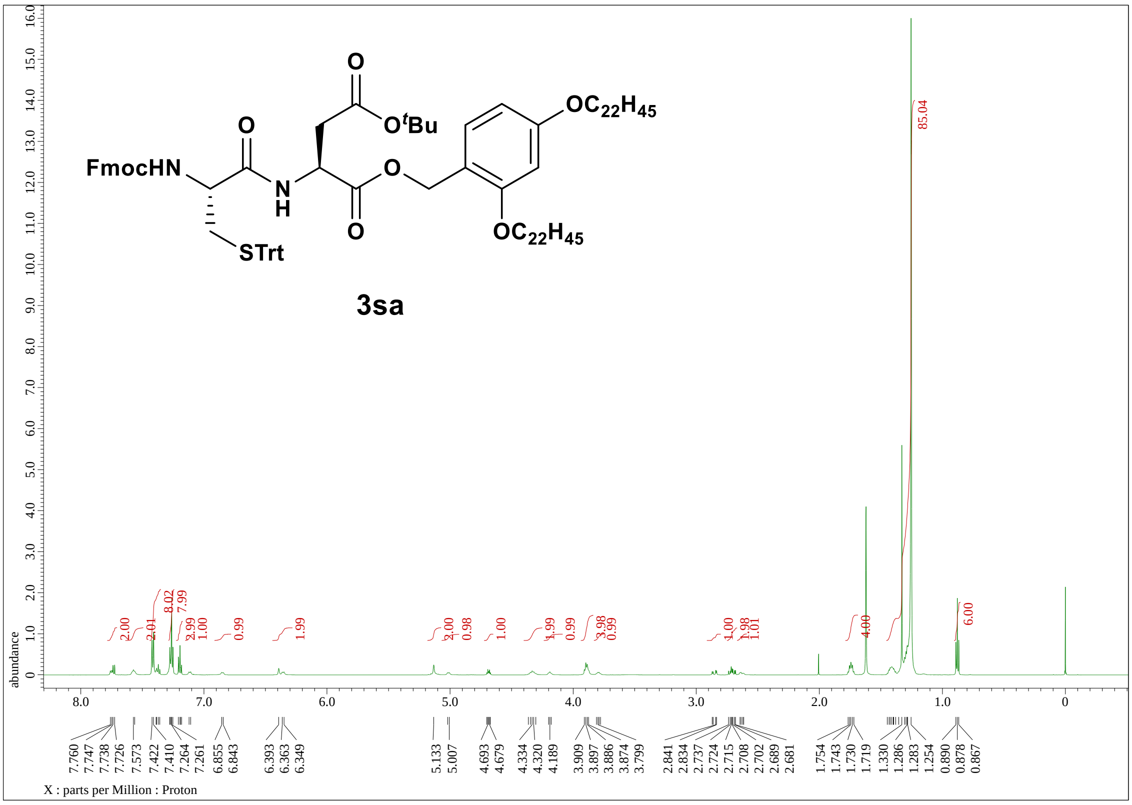

FmocHN
STrt
N
H
O
O
O
O
O
OtBu
OC22H45
OC22H45
3sa
abundance
X : parts per Million : Proton

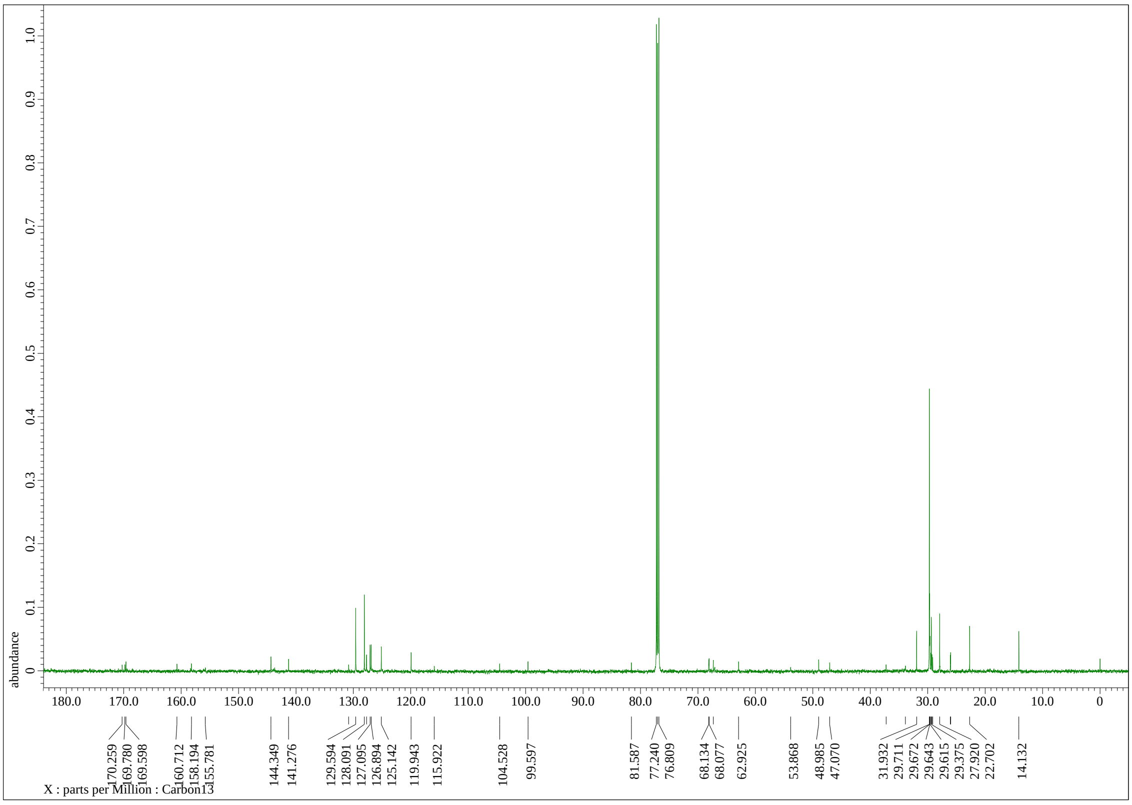

abundance
X : parts per Million : Carbon13

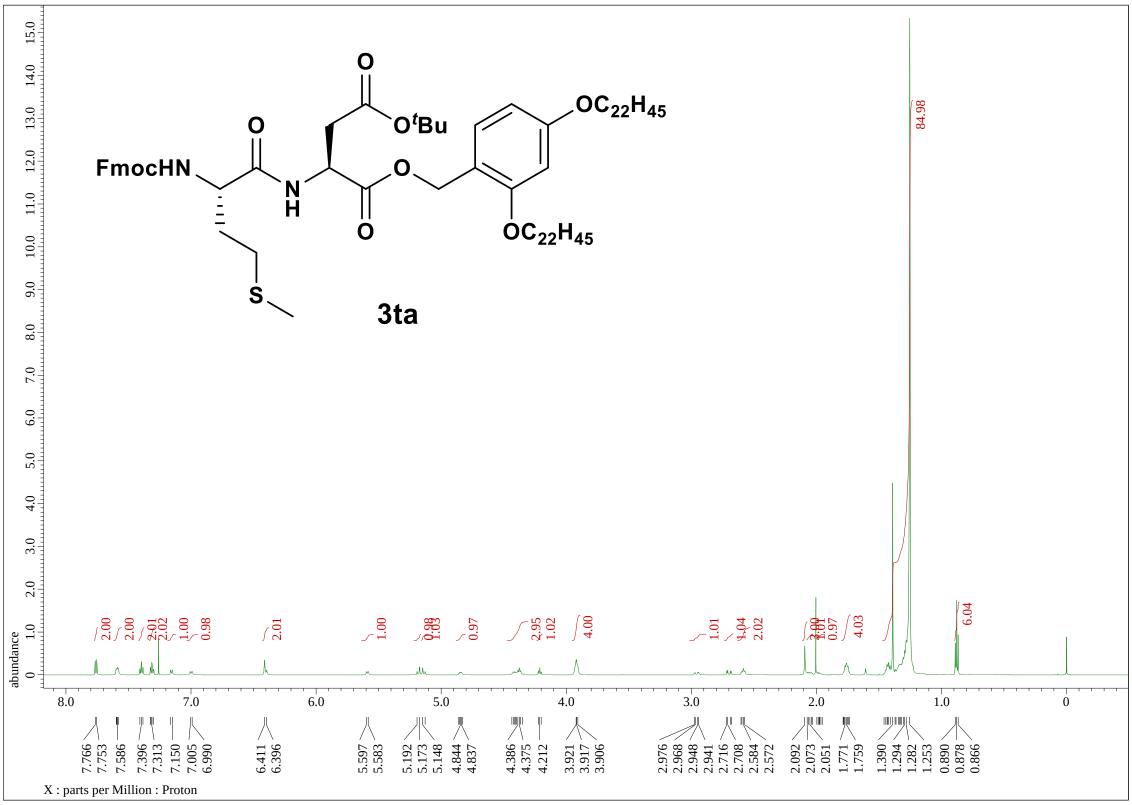
FmocHN
O
N
H
O
O
O^tBu
O
OC22H45
OC22H45
S
3ta
84.98
2.00 2.00 2.01 2.02 1.00 0.98 2.01 1.00 0.98 1.03 0.97 2.95 1.02 4.00 1.01 1.04 2.02 1.00 1.01 0.97 4.03 6.04
7.766 7.753 7.586 7.396 7.313 7.150 7.005 6.990 6.411 6.396 5.597 5.583 5.192 5.173 5.148 4.844 4.837 4.386 4.375 4.212 3.921 3.917 3.906 2.976 2.968 2.948 2.941 2.716 2.708 2.584 2.572 2.092 2.073 2.051 1.771 1.759 1.390 1.294 1.282 1.253 0.890 0.878 0.866
abundance
X : parts per Million : Proton

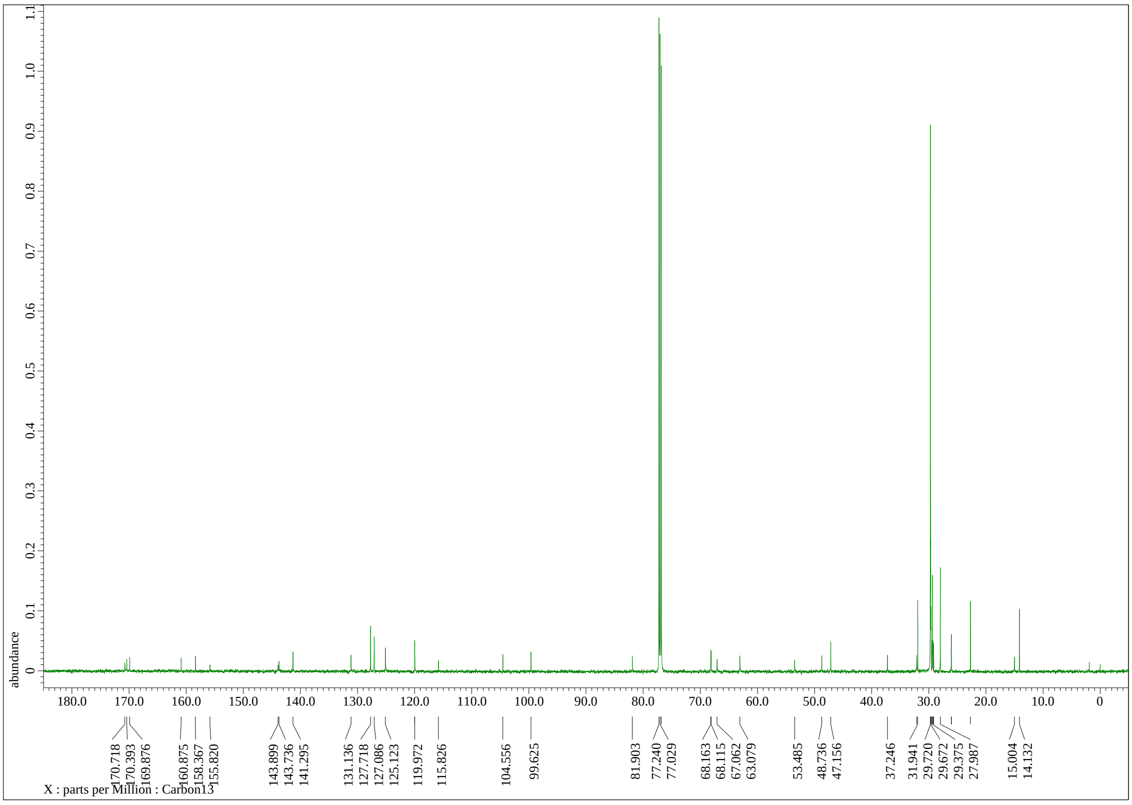
170.718 170.393 169.876 160.875 158.367 155.820 143.899 143.736 141.295 131.136 127.718 127.086 125.123 119.972 115.826 104.556 99.625 81.903 77.240 77.029 68.163 68.115 67.062 63.079 53.485 48.736 47.156 37.246 31.941 29.720 29.672 29.375 27.987 15.004 14.132
abundance
X : parts per Million : Carbon13

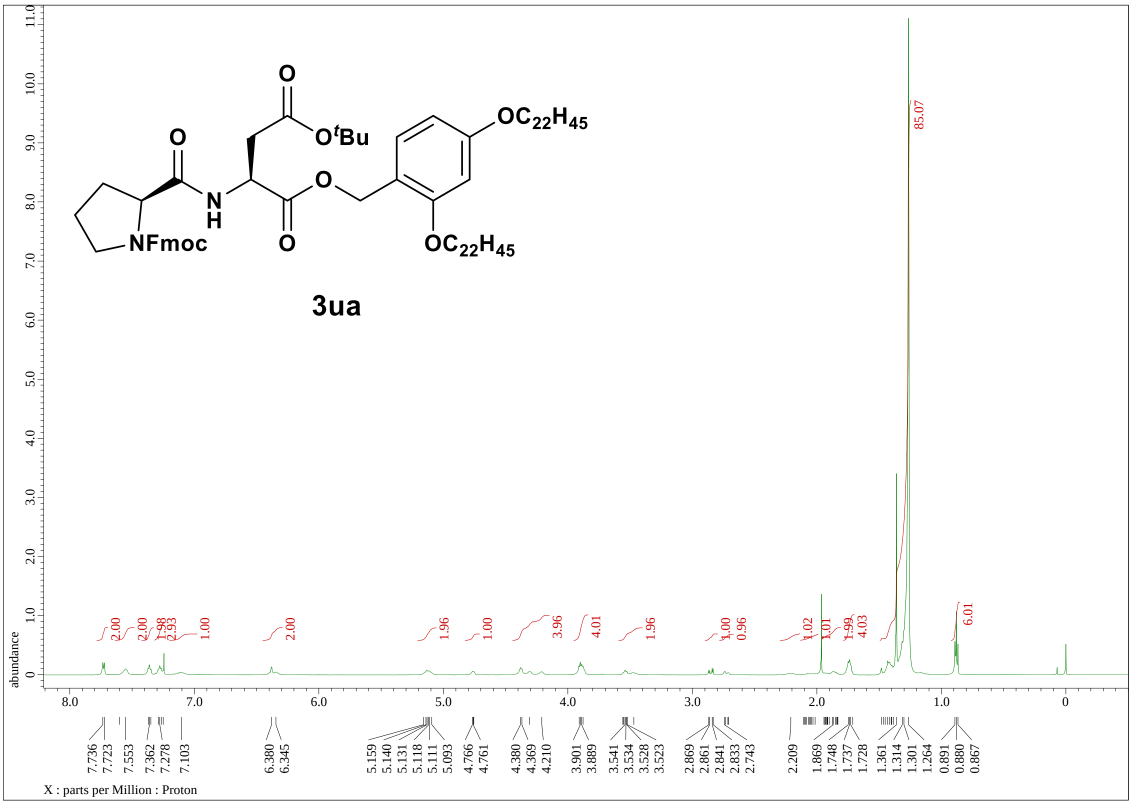

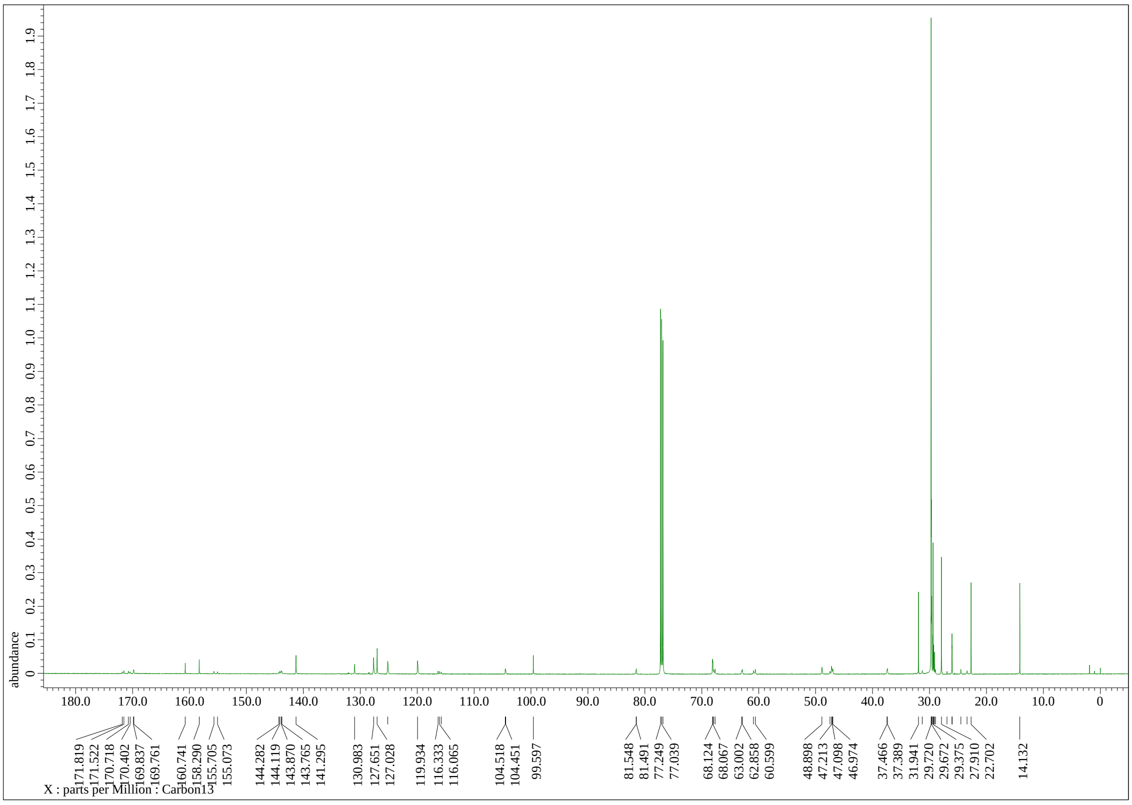

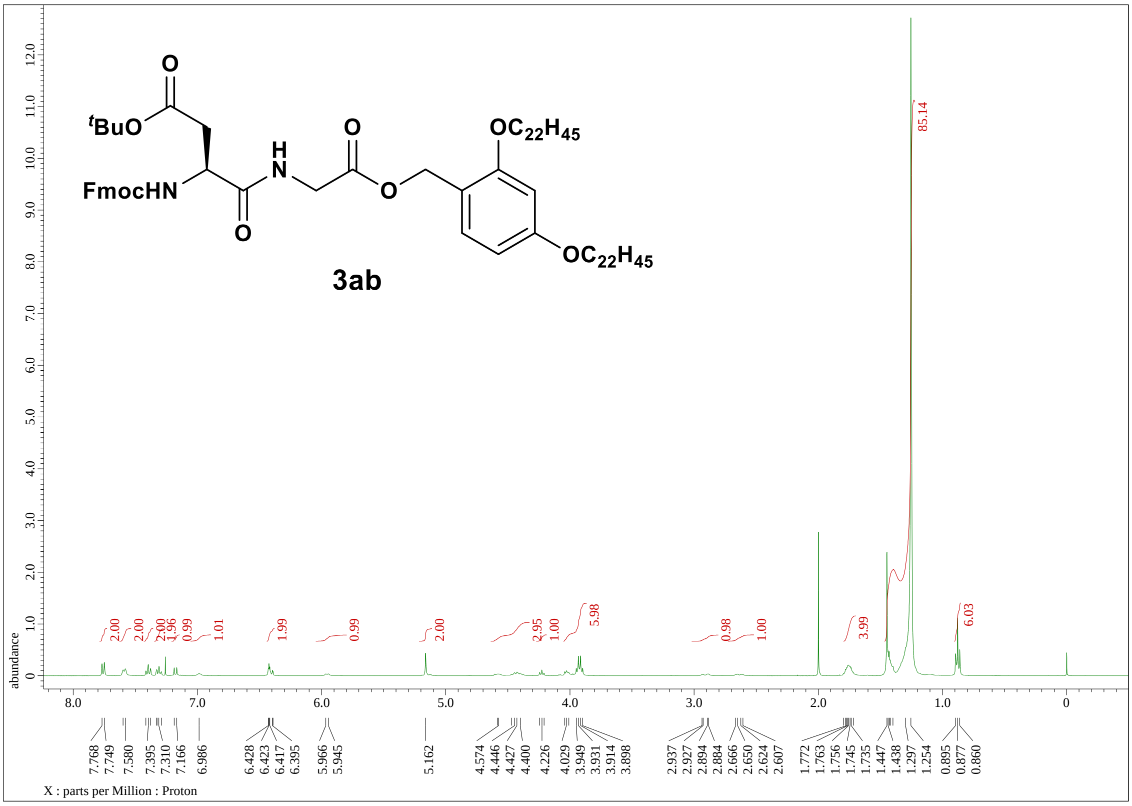
tBuO
FmocHN
OC22H45
OC22H45
3ab
85.14
2.00 2.00 2.00 1.96 0.99 1.01 1.99 0.99 2.00 2.95 1.00 5.98 0.98 1.00 3.99 6.03
7.768 7.749 7.580 7.395 7.310 7.166 6.986 6.428 6.423 6.417 6.395 5.966 5.945 5.162 4.574 4.446 4.427 4.400 4.226 4.029 3.949 3.931 3.914 3.898 2.937 2.927 2.894 2.884 2.666 2.650 2.624 2.607 1.772 1.763 1.756 1.745 1.735 1.447 1.438 1.297 1.254 0.895 0.877 0.860
abundance
X : parts per Million : Proton

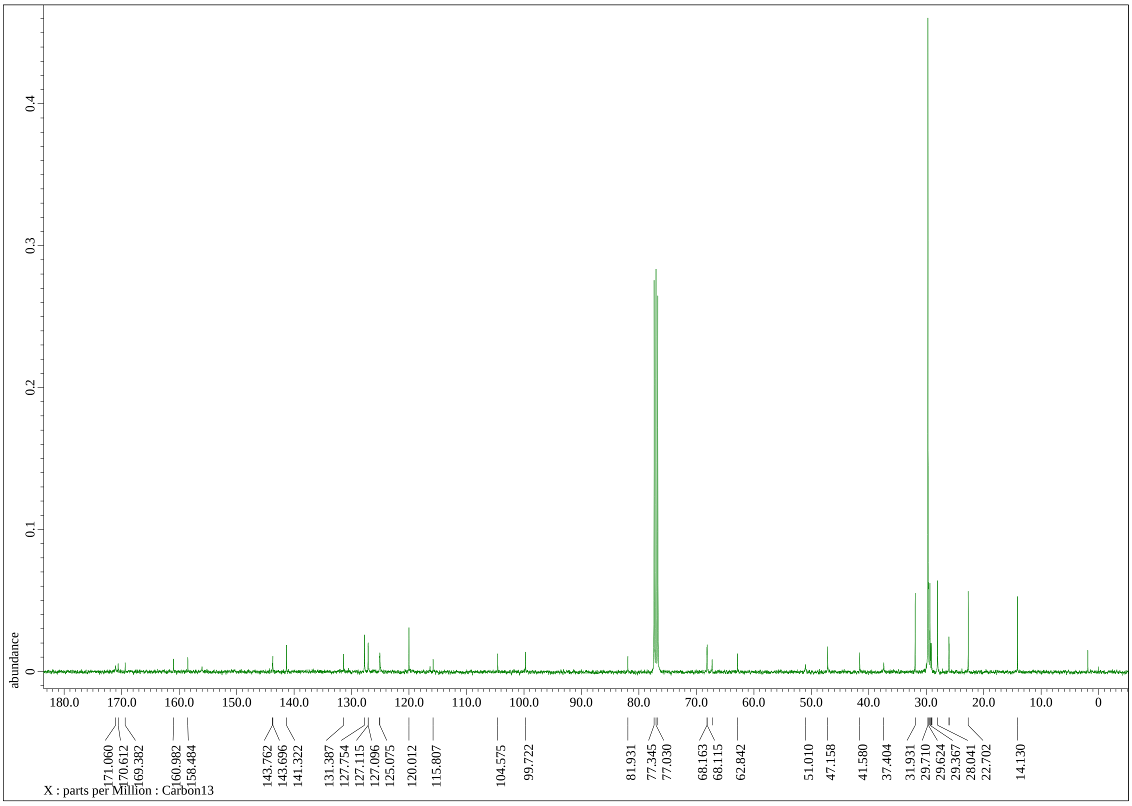
171.060 170.612 169.382 160.982 158.484 143.762 143.696 141.322 131.387 127.754 127.115 127.096 125.075 120.012 115.807 104.575 99.722 81.931 77.345 77.030 68.163 68.115 62.842 51.010 47.158 41.580 37.404 31.931 29.710 29.624 29.367 28.041 22.702 14.130
abundance
X : parts per Million : Carbon13

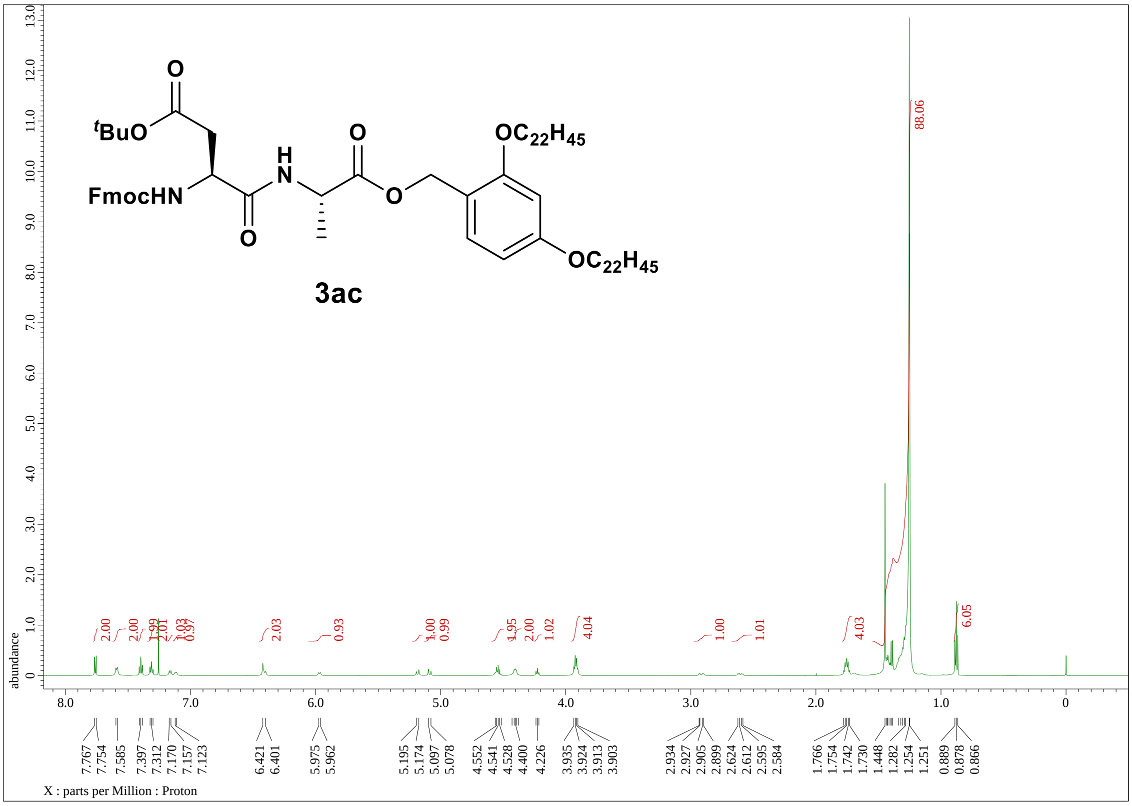

O
tBuO
FmocHN
H
N
O
O
O
OC22H45
OC22H45
3ac
abundance
X : parts per Million : Proton
2.00
2.00
1.99
2.01
1.03
0.97
2.03
0.93
1.00
0.99
1.95
2.00
1.02
4.04
1.00
1.01
4.03
88.06
6.05
7.767
7.754
7.585
7.397
7.312
7.170
7.157
7.123
6.421
6.401
5.975
5.962
5.195
5.174
5.097
5.078
4.552
4.541
4.528
4.400
4.226
3.935
3.924
3.913
3.903
2.934
2.927
2.905
2.899
2.624
2.612
2.595
2.584
1.766
1.754
1.742
1.730
1.448
1.282
1.254
1.251
0.889
0.878
0.866

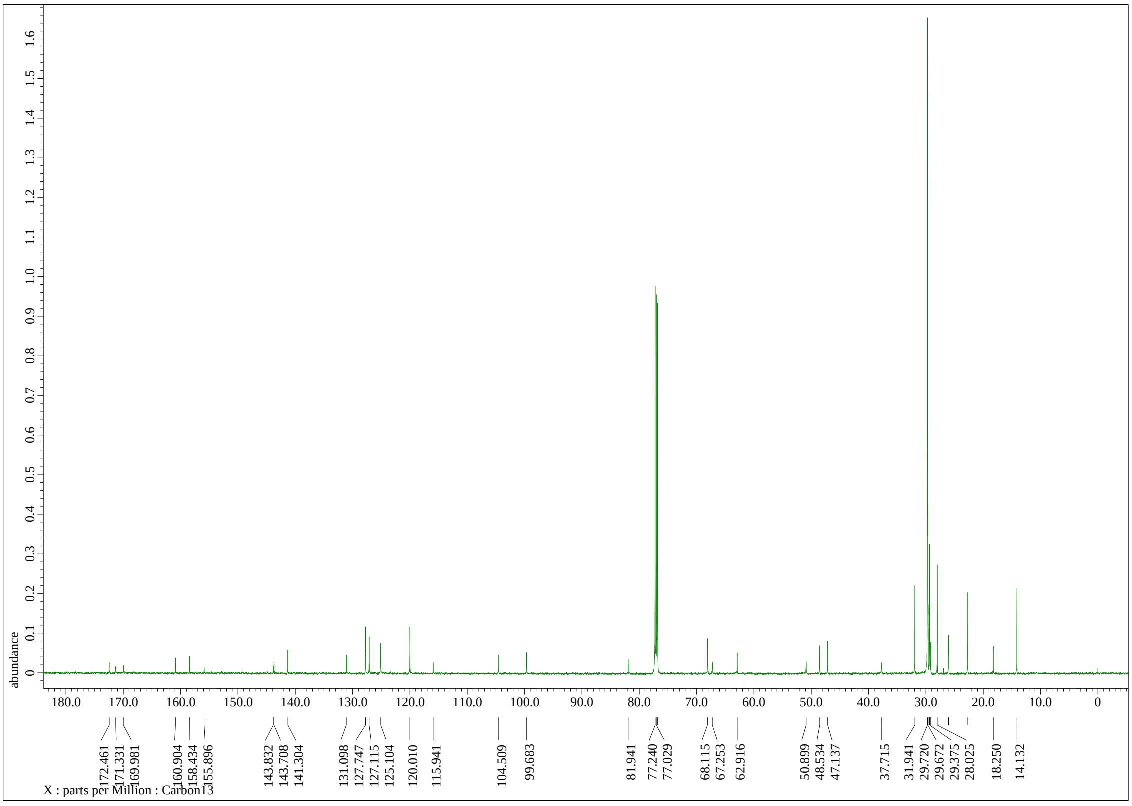

abundance
X : parts per Million : Carbon13
172.461
171.331
169.981
160.904
158.434
155.896
143.832
143.708
141.304
131.098
127.747
127.115
125.104
120.010
115.941
104.509
99.683
81.941
77.240
77.029
68.115
67.253
62.916
50.899
48.534
47.137
37.715
31.941
29.720
29.672
29.375
28.025
18.250
14.132

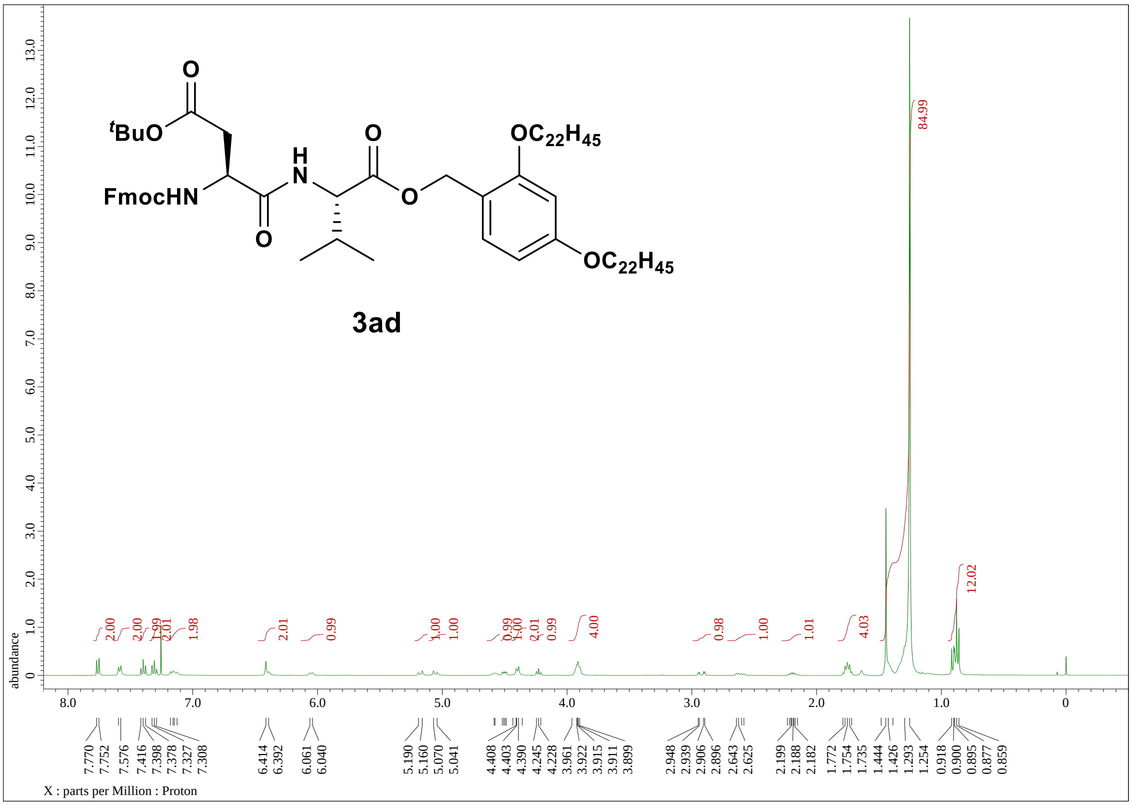

tBuO
FmocHN
3ad
OC22H45
X : parts per Million : Proton
abundance

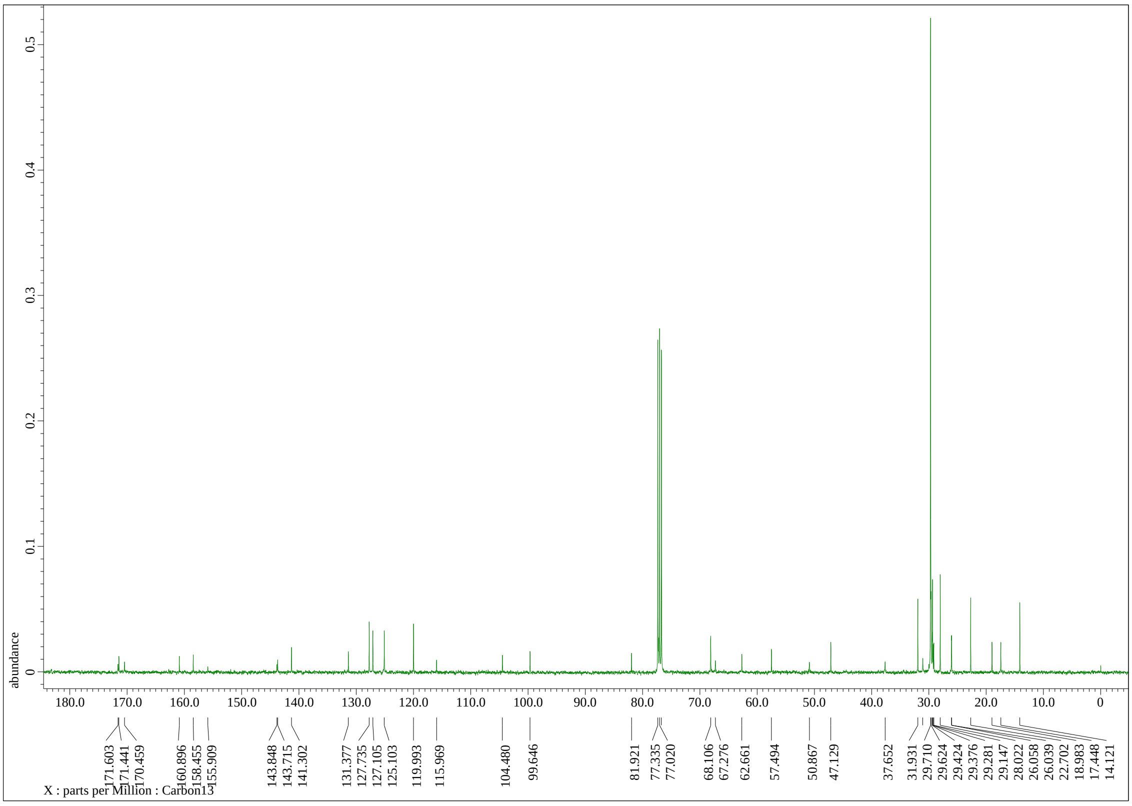

X : parts per Million : Carbon13
abundance

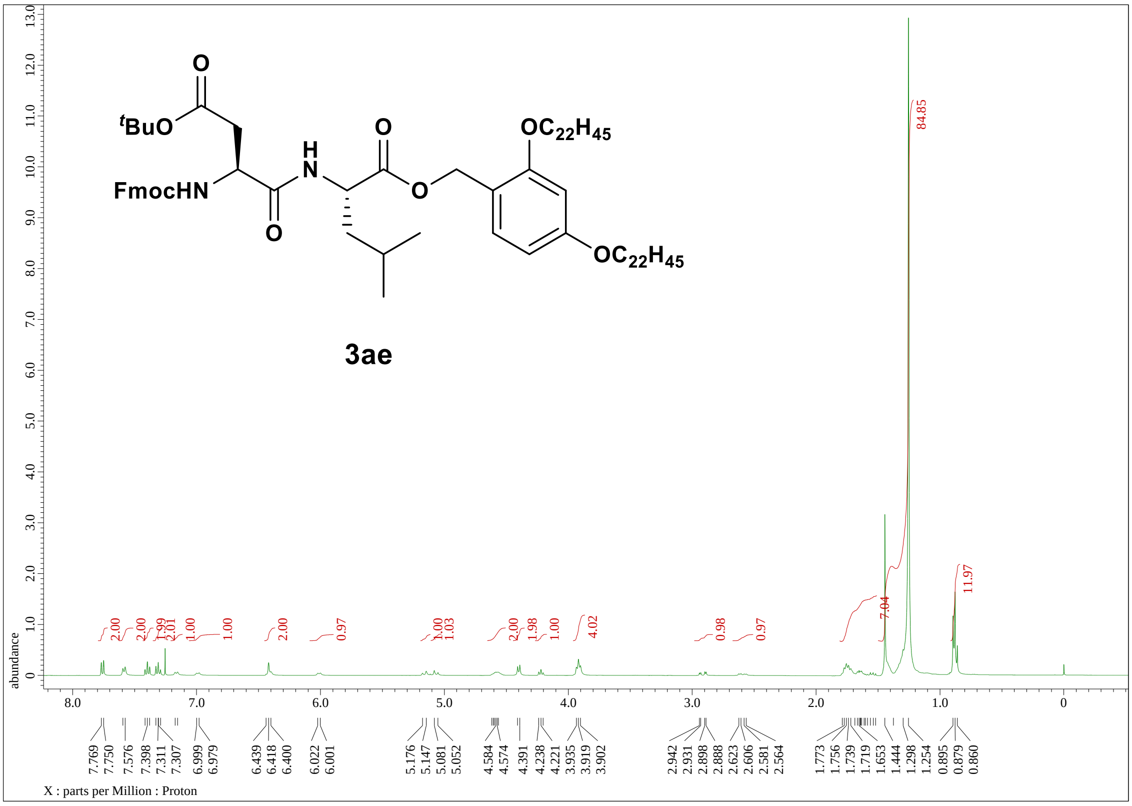
3ae
tBuO
FmocHN
OC22H45
abundance
X : parts per Million : Proton
7.769 7.750 7.576 7.398 7.311 7.307 6.999 6.979 6.439 6.418 6.400 6.022 6.001 5.176 5.147 5.081 5.052 4.584 4.574 4.391 4.238 4.221 3.935 3.919 3.902 2.942 2.931 2.898 2.888 2.623 2.606 2.581 2.564 1.773 1.756 1.739 1.719 1.653 1.444 1.298 1.254 0.895 0.879 0.860
2.00 2.00 1.99 2.01 1.00 1.00 2.00 0.97 1.00 1.03 2.00 1.98 1.00 4.02 0.98 0.97 7.04 84.85 11.97

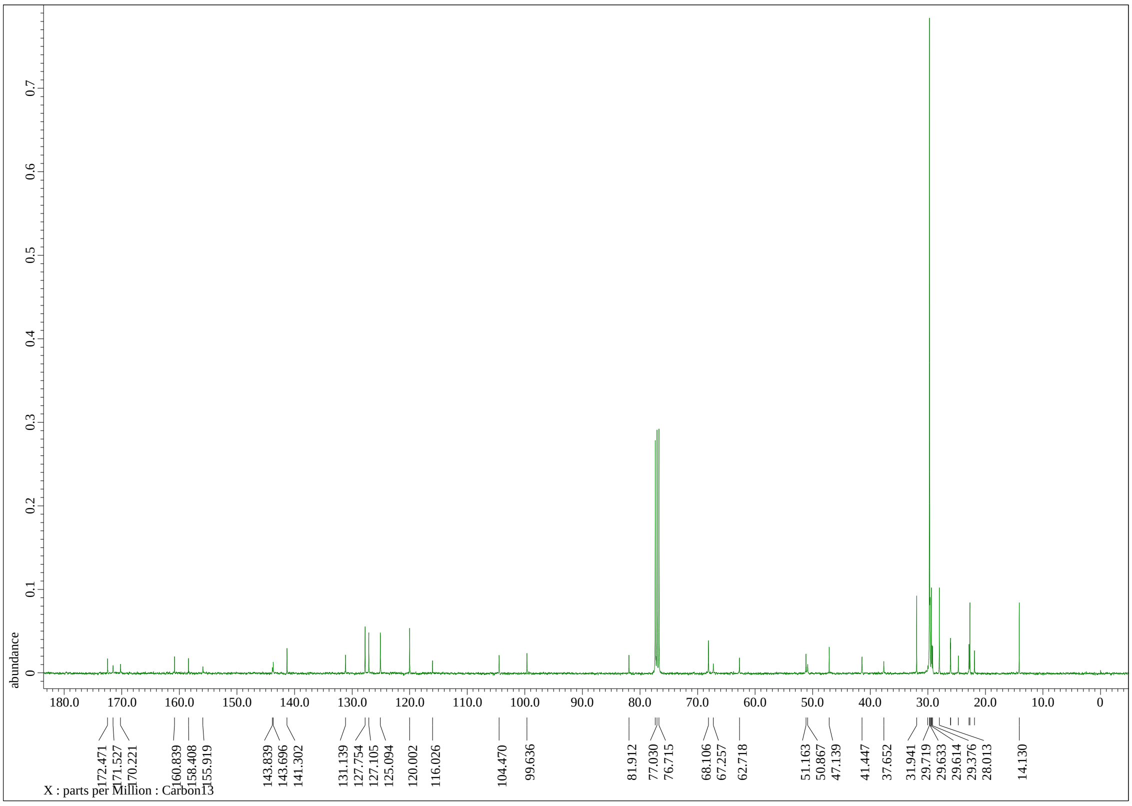
abundance
X : parts per Million : Carbon13
172.471 171.527 170.221 160.839 158.408 155.919 143.839 143.696 141.302 131.139 127.754 127.105 125.094 120.002 116.026 104.470 99.636 81.912 77.030 76.715 68.106 67.257 62.718 51.163 50.867 47.139 41.447 37.652 31.941 29.719 29.633 29.614 29.376 28.013 14.130

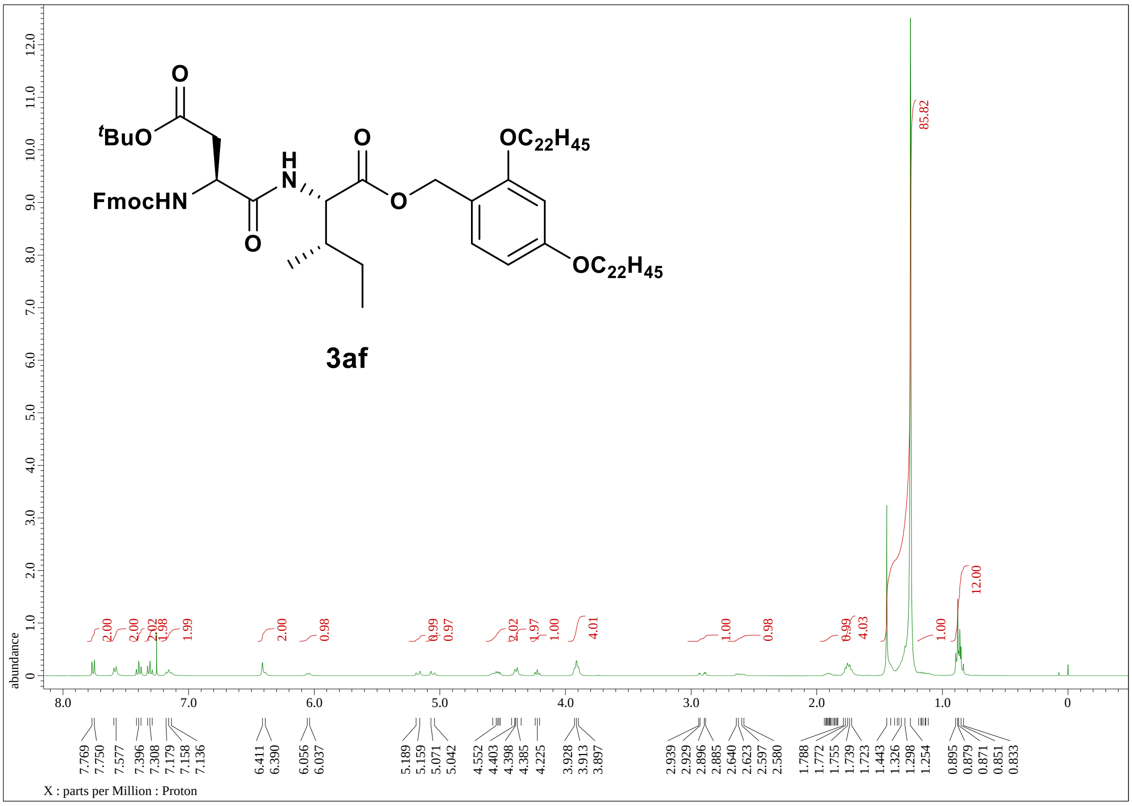
3af
abundance
7.769 7.750 7.577 7.396 7.308 7.179 7.158 7.136 6.411 6.390 6.056 6.037 5.189 5.159 5.071 5.042 4.552 4.403 4.398 4.385 4.225 3.928 3.913 3.897 2.939 2.929 2.896 2.885 2.640 2.623 2.597 2.580 1.788 1.772 1.755 1.739 1.723 1.443 1.326 1.298 1.254 0.895 0.879 0.871 0.851 0.833
2.00 2.00 2.02 1.98 1.99 2.00 0.98 0.99 0.97 2.02 1.97 1.00 4.01 1.00 0.98 0.99 4.03 1.00 85.82 12.00
X : parts per Million : Proton

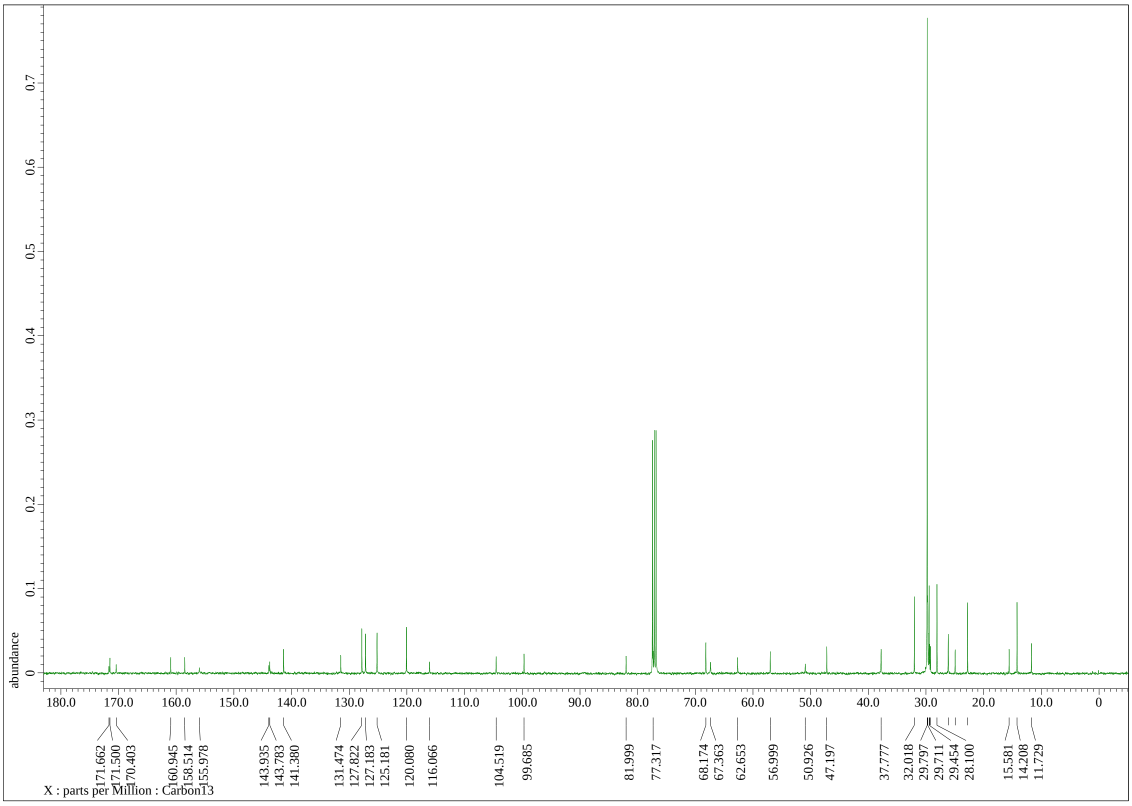
abundance
171.662 171.500 170.403 160.945 158.514 155.978 143.935 143.783 141.380 131.474 127.822 127.183 125.181 120.080 116.066 104.519 99.685 81.999 77.317 68.174 67.363 62.653 56.999 50.926 47.197 37.777 32.018 29.797 29.711 29.454 28.100 15.581 14.208 11.729
X : parts per Million : Carbon13

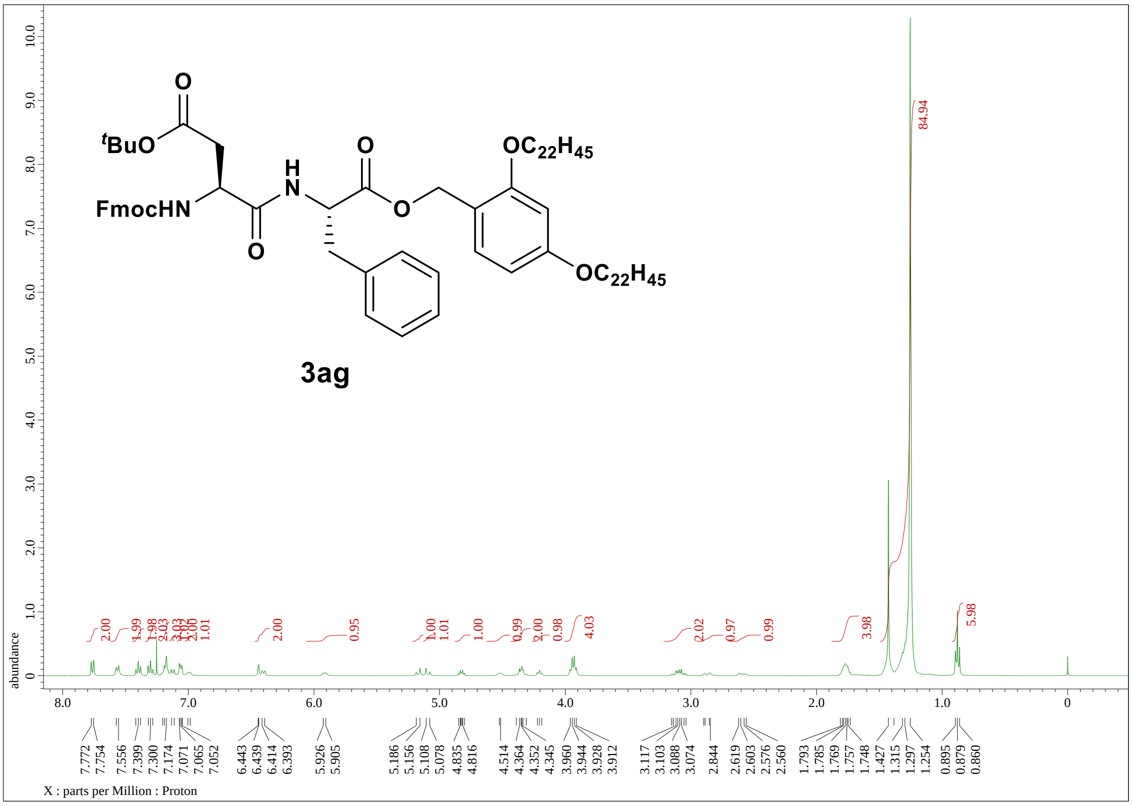

O
tBuO
FmocHN
H
N
O
O
O
OC22H45
OC22H45
3ag
abundance
X : parts per Million : Proton
84.94
3.98
5.98
4.03

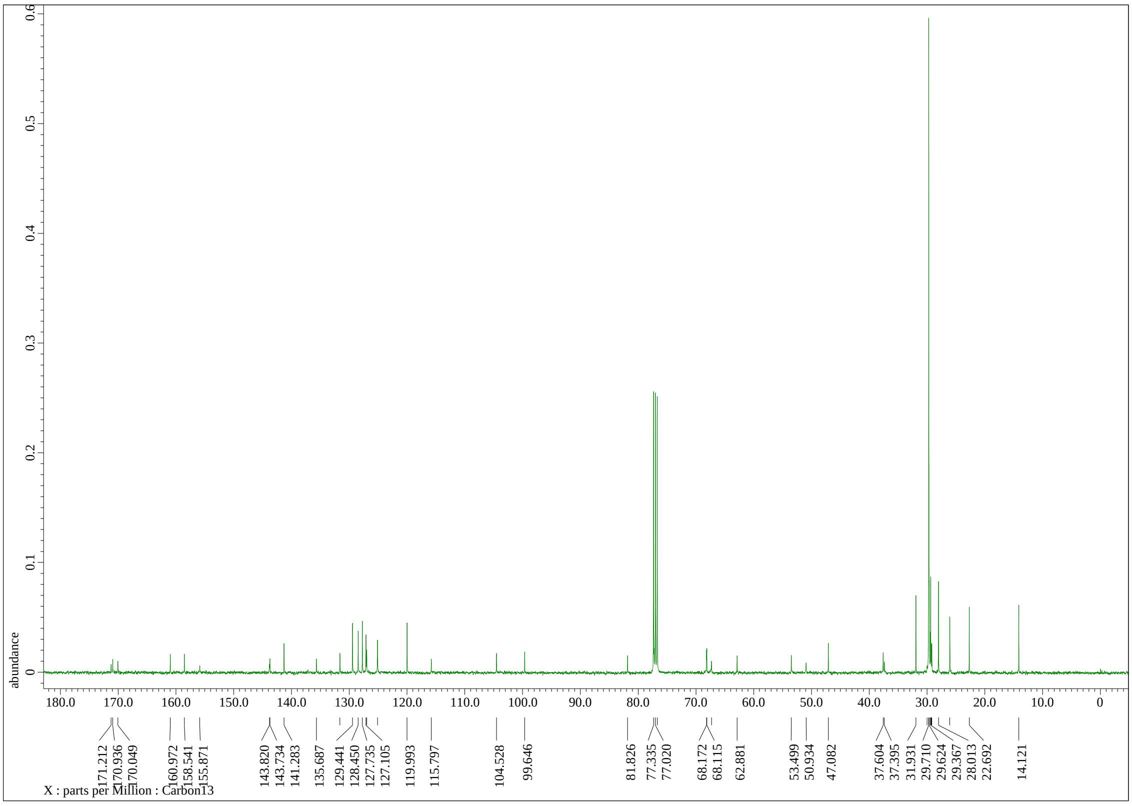

abundance
X : parts per Million : Carbon13
171.212
170.936
170.049
160.972
158.541
155.871
143.820
143.734
141.283
135.687
129.441
128.450
127.735
127.105
119.993
115.797
104.528
99.646
81.826
77.335
77.020
68.172
68.115
62.881
53.499
50.934
47.082
37.604
37.395
31.931
29.710
29.624
29.367
28.013
22.692
14.121

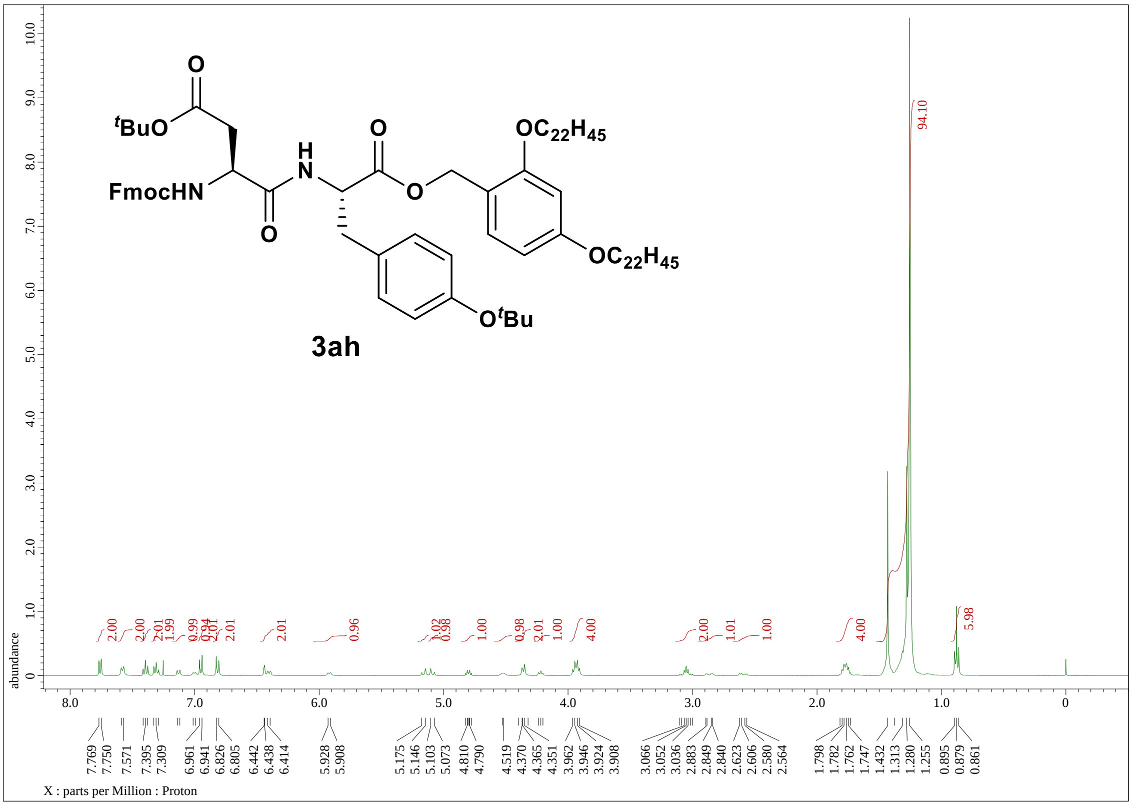
3ah
FmocHN
tBuO
OC22H45
OtBu
abundance
X : parts per Million : Proton

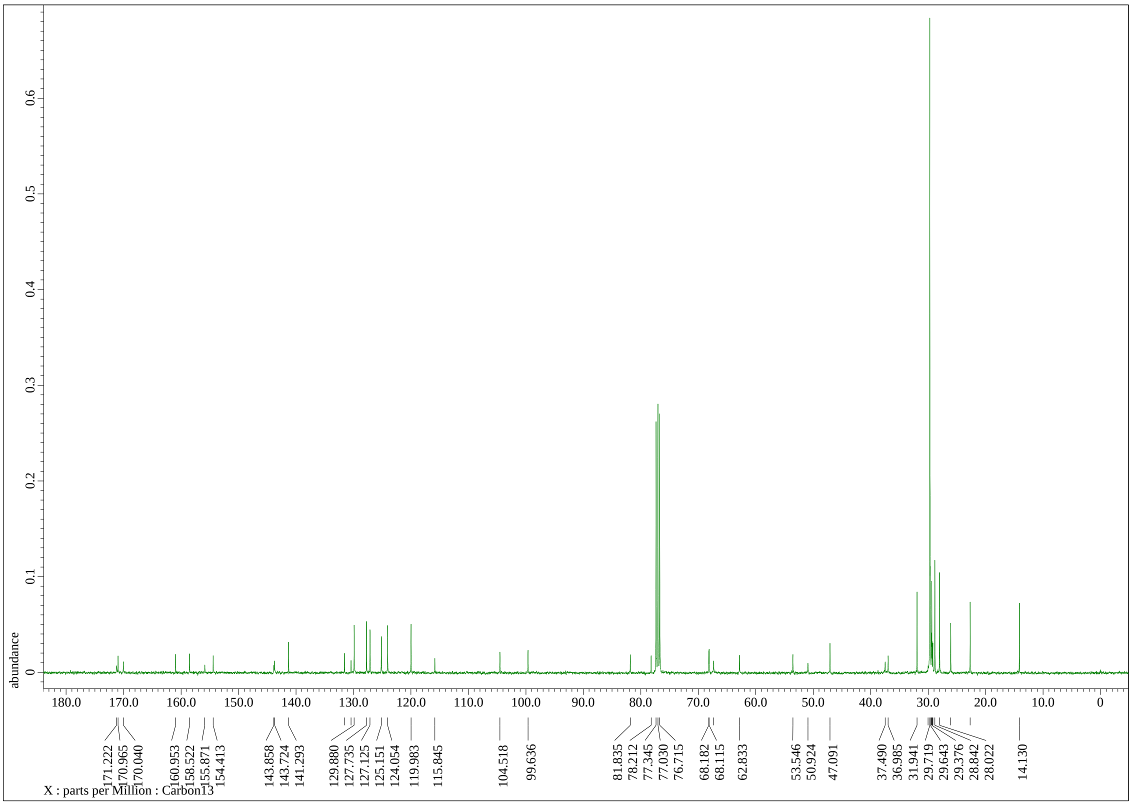
abundance
X : parts per Million : Carbon13

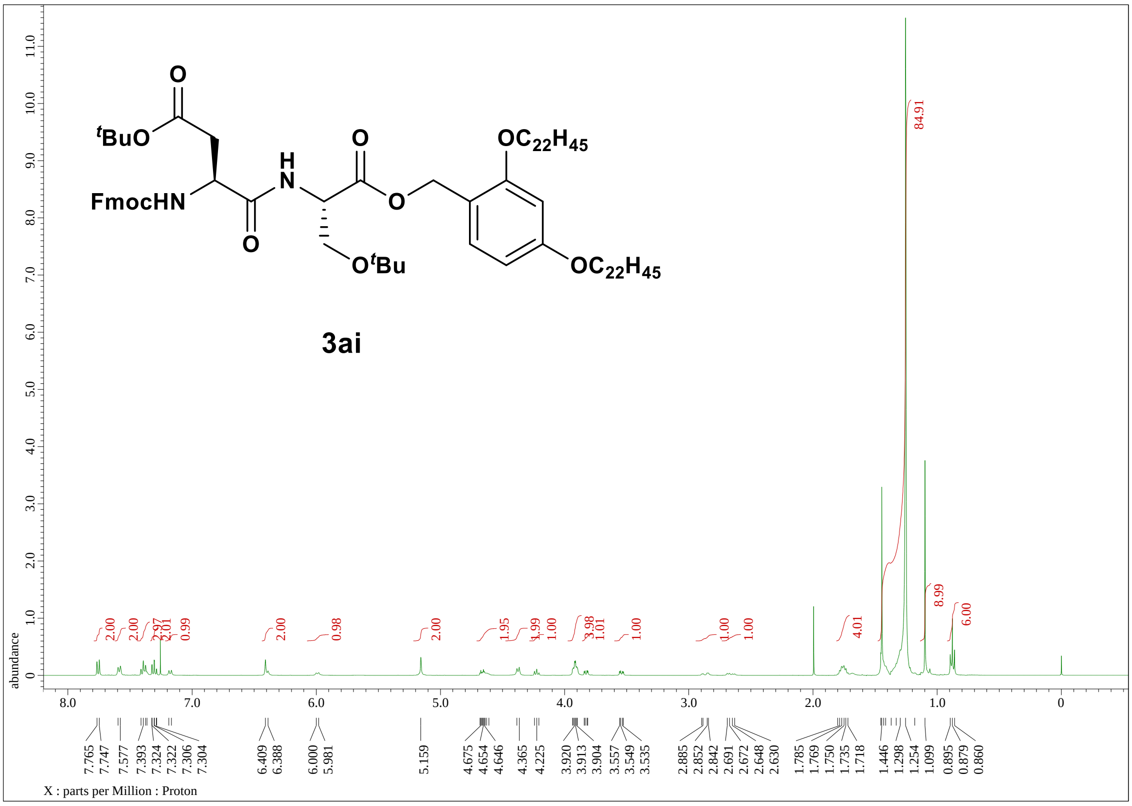

tBuO
FmocHN
OC22H45
O'Bu
OC22H45
3ai
84.91
8.99
6.00
4.01
X : parts per Million : Proton

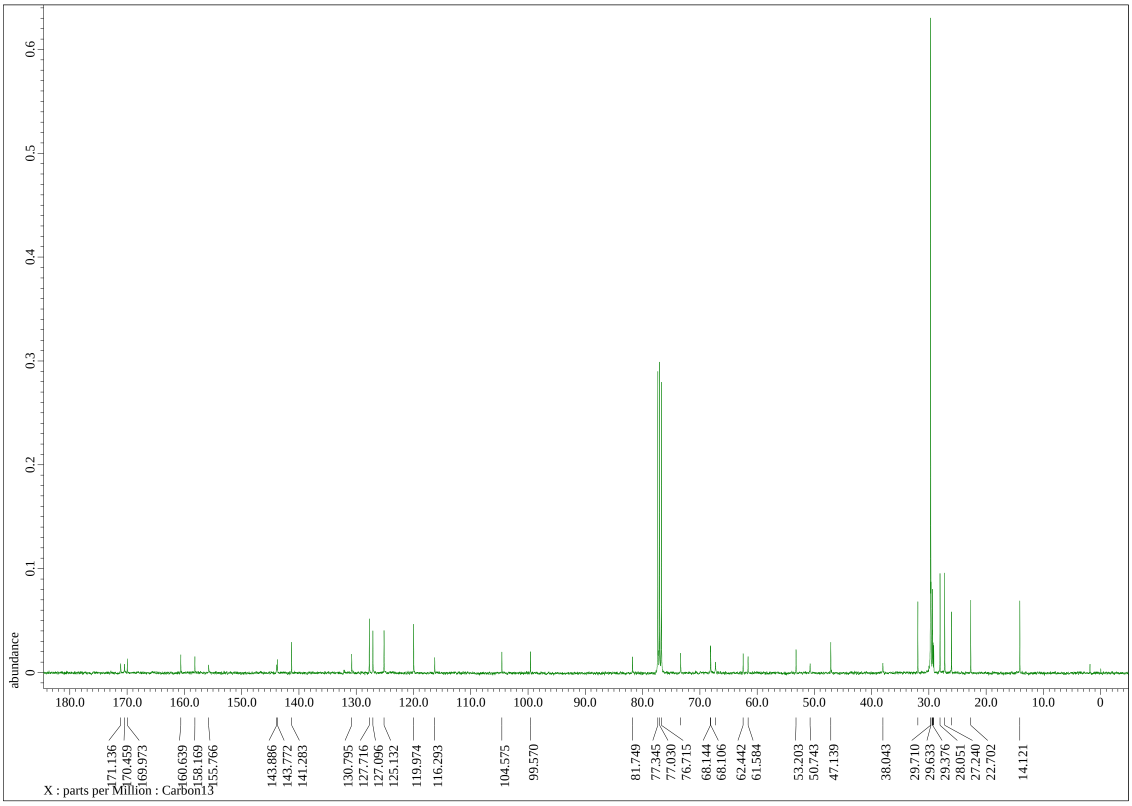

X : parts per Million : Carbon13

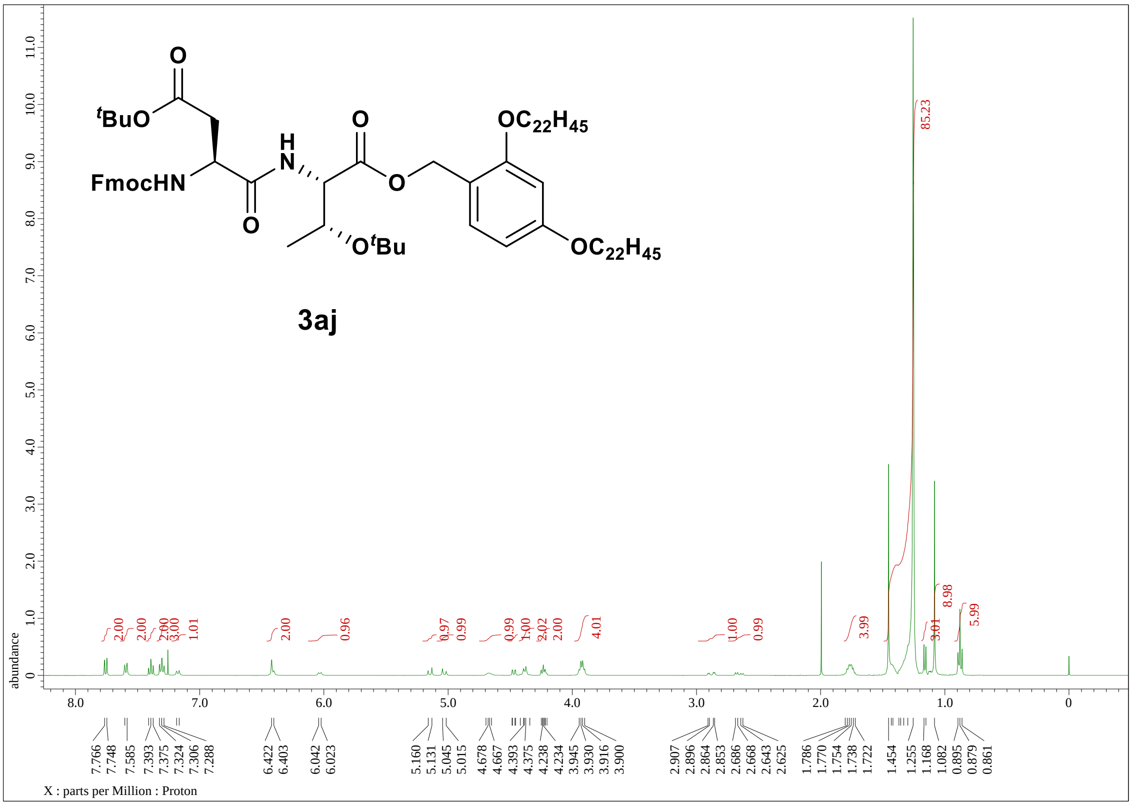

tBuO
FmocHN
O
H
N
O
O
OC22H45
OtBu
OC22H45
3aj
abundance
2.00 2.00 2.00 3.00 1.01 2.00 0.96 0.97 0.99 0.99 1.00 2.02 2.00 4.01 1.00 0.99 3.99 85.23 3.01 8.98 5.99
7.766 7.748 7.585 7.393 7.375 7.324 7.306 7.288 6.422 6.403 6.042 6.023 5.160 5.131 5.045 5.015 4.678 4.667 4.393 4.375 4.238 4.234 3.945 3.930 3.916 3.900 2.907 2.896 2.864 2.853 2.686 2.668 2.643 2.625 1.786 1.770 1.754 1.738 1.722 1.454 1.255 1.168 1.082 0.895 0.879 0.861
X : parts per Million : Proton

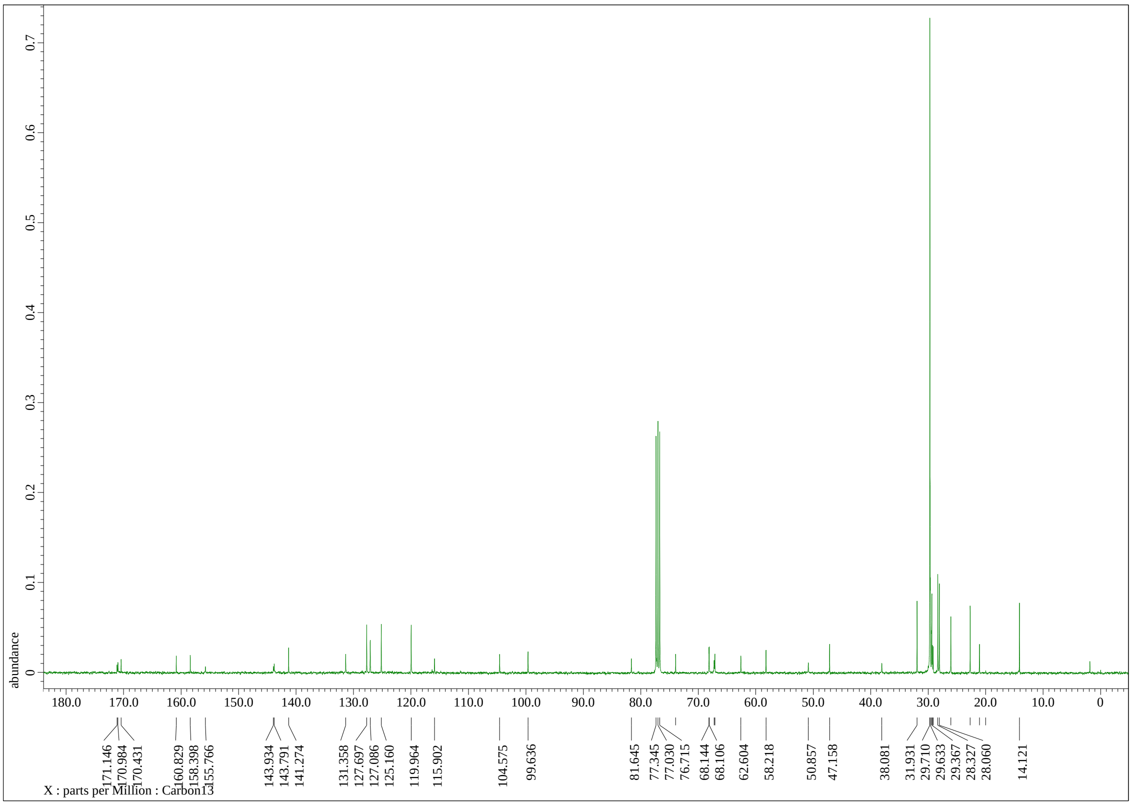

abundance
171.146 170.984 170.431 160.829 158.398 155.766 143.934 143.791 141.274 131.358 127.697 127.086 125.160 119.964 115.902 104.575 99.636 81.645 77.345 77.030 76.715 68.144 68.106 62.604 58.218 50.857 47.158 38.081 31.931 29.710 29.633 29.367 28.327 28.060 14.121
X : parts per Million : Carbon13

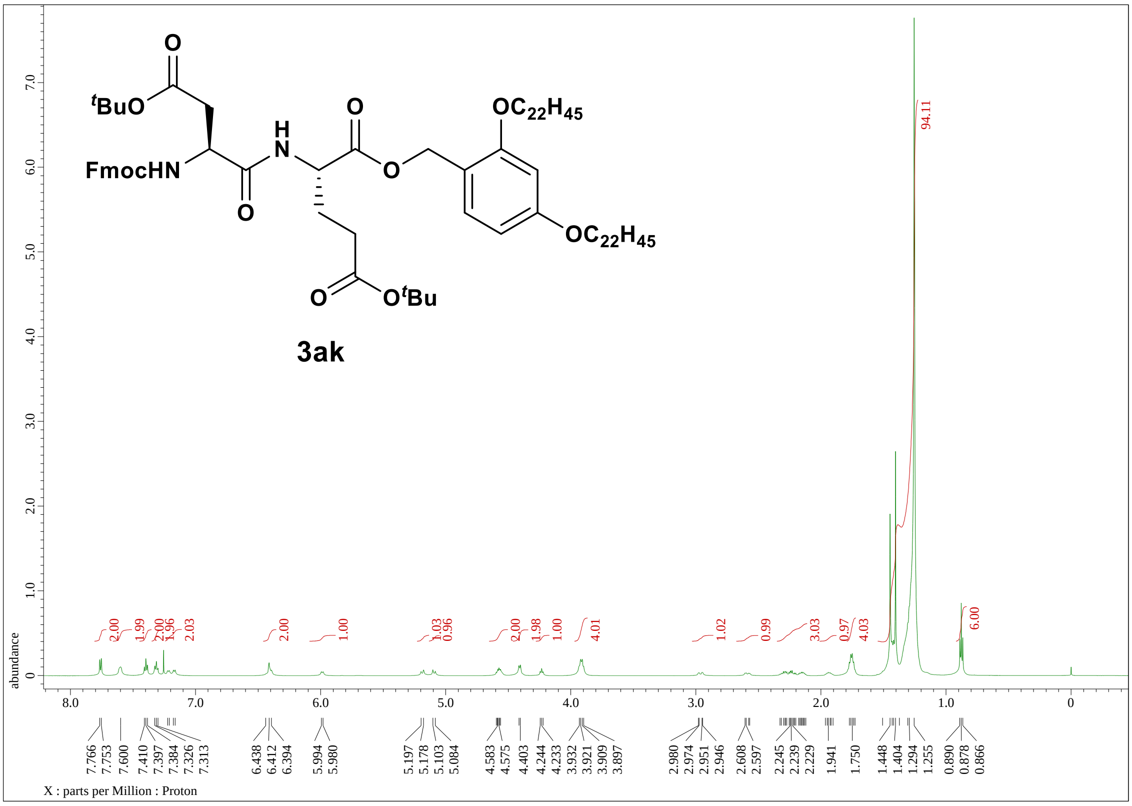

3ak
X : parts per Million : Proton
abundance
7.766
7.753
7.600
7.410
7.397
7.384
7.326
7.313
6.438
6.412
6.394
5.994
5.980
5.197
5.178
5.103
5.084
4.583
4.575
4.403
4.244
4.233
3.932
3.921
3.909
3.897
2.980
2.974
2.951
2.946
2.608
2.597
2.245
2.239
2.229
1.941
1.750
1.448
1.404
1.294
1.255
0.890
0.878
0.866
94.11

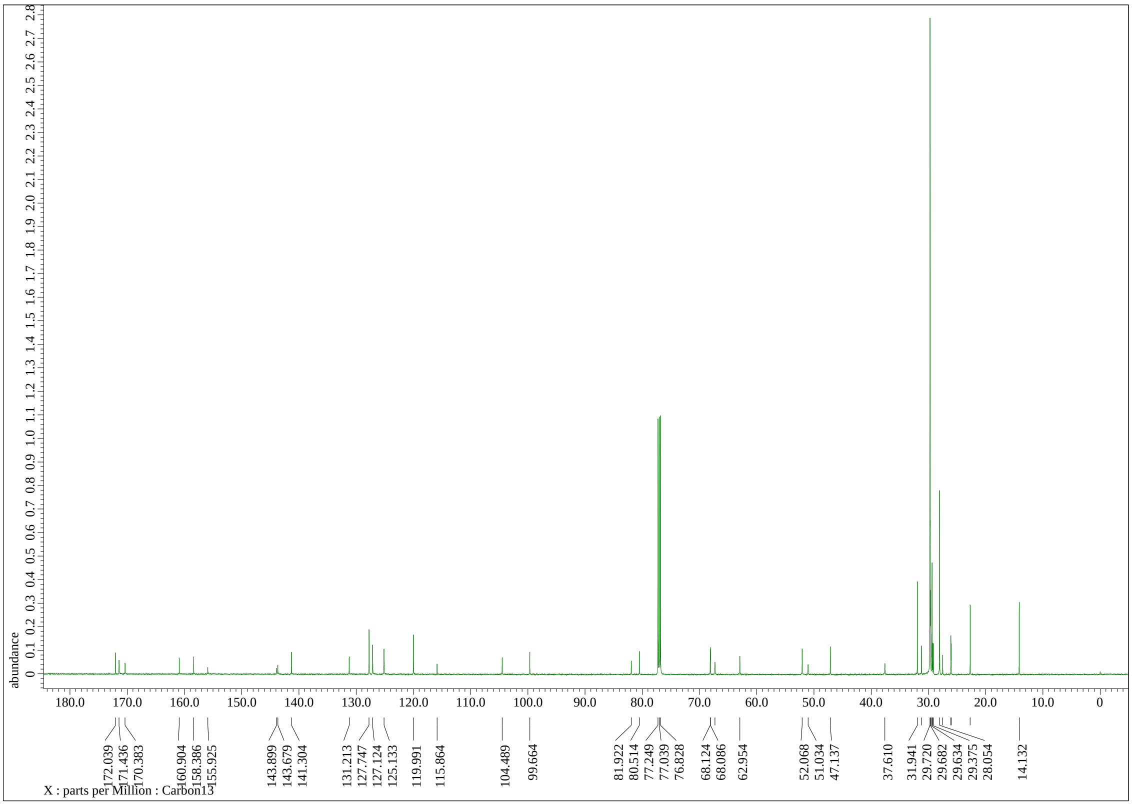

X : parts per Million : Carbon13
abundance
172.039
171.436
170.383
160.904
158.386
155.925
143.899
143.679
141.304
131.213
127.747
127.124
125.133
119.991
115.864
104.489
99.664
81.922
80.514
77.249
77.039
76.828
68.124
68.086
62.954
52.068
51.034
47.137
37.610
31.941
29.720
29.682
29.634
29.375
28.054
14.132

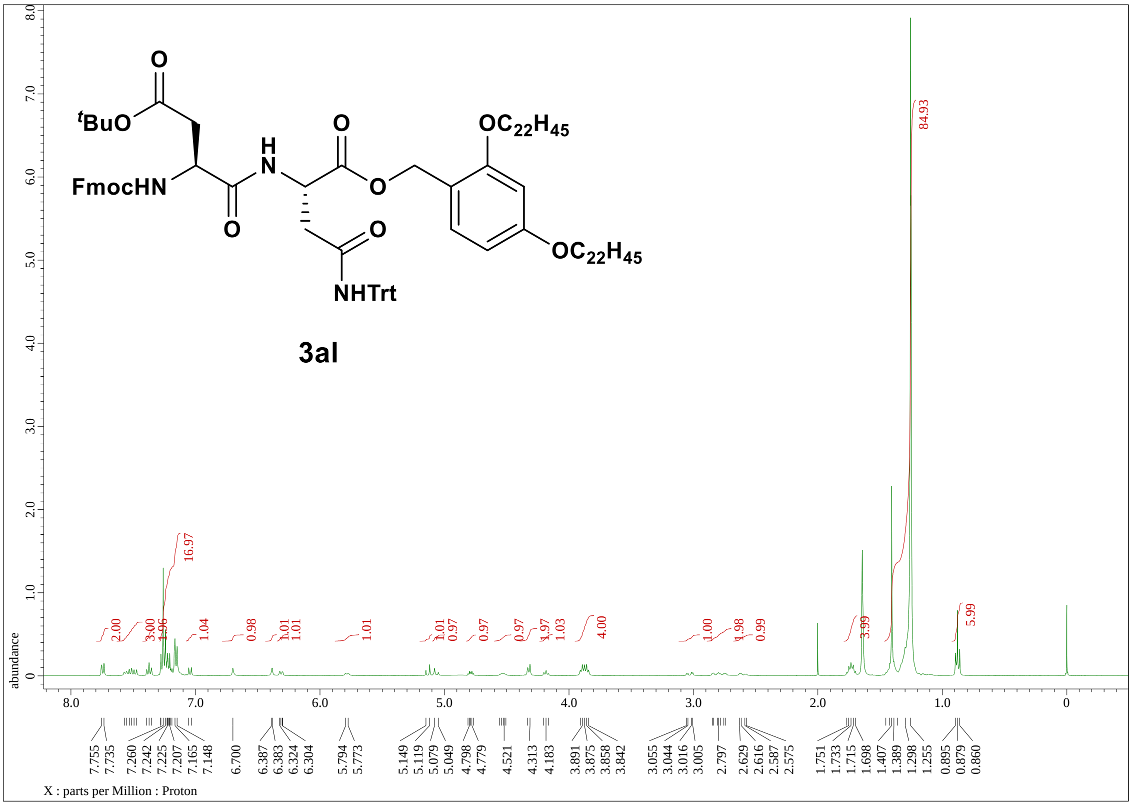

3al
X : parts per Million : Proton
abundance

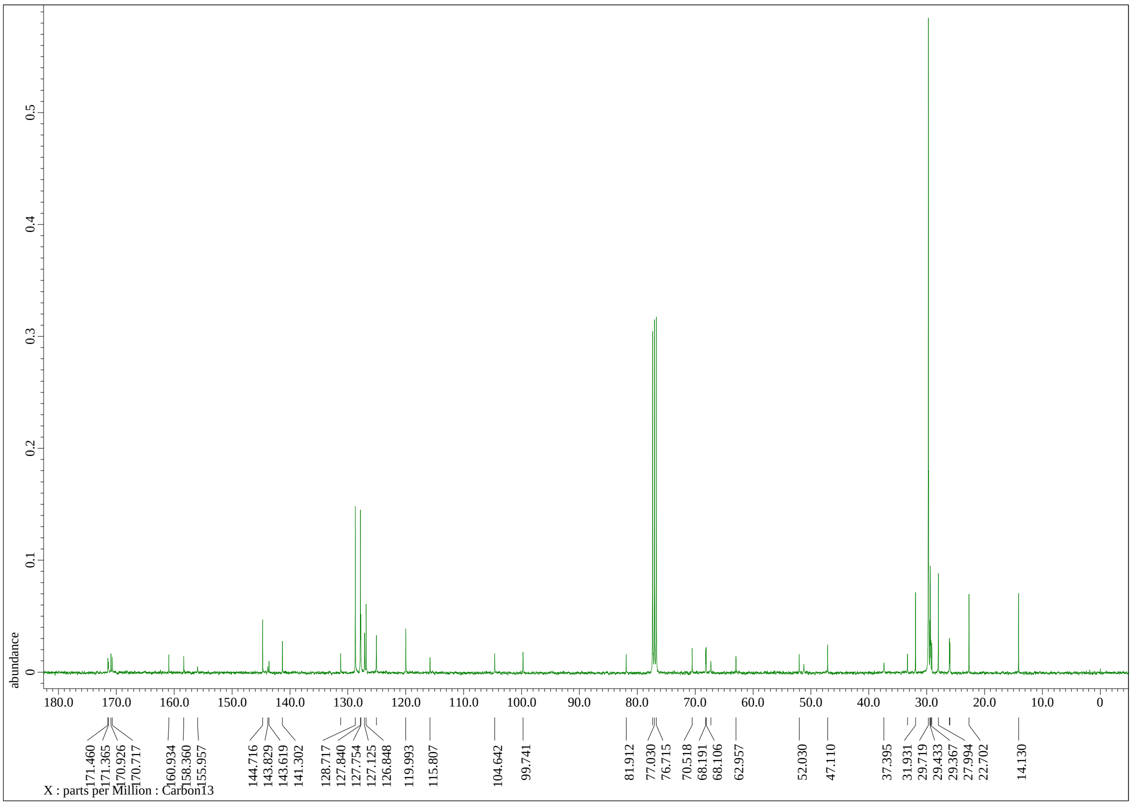

X : parts per Million : Carbon13
abundance

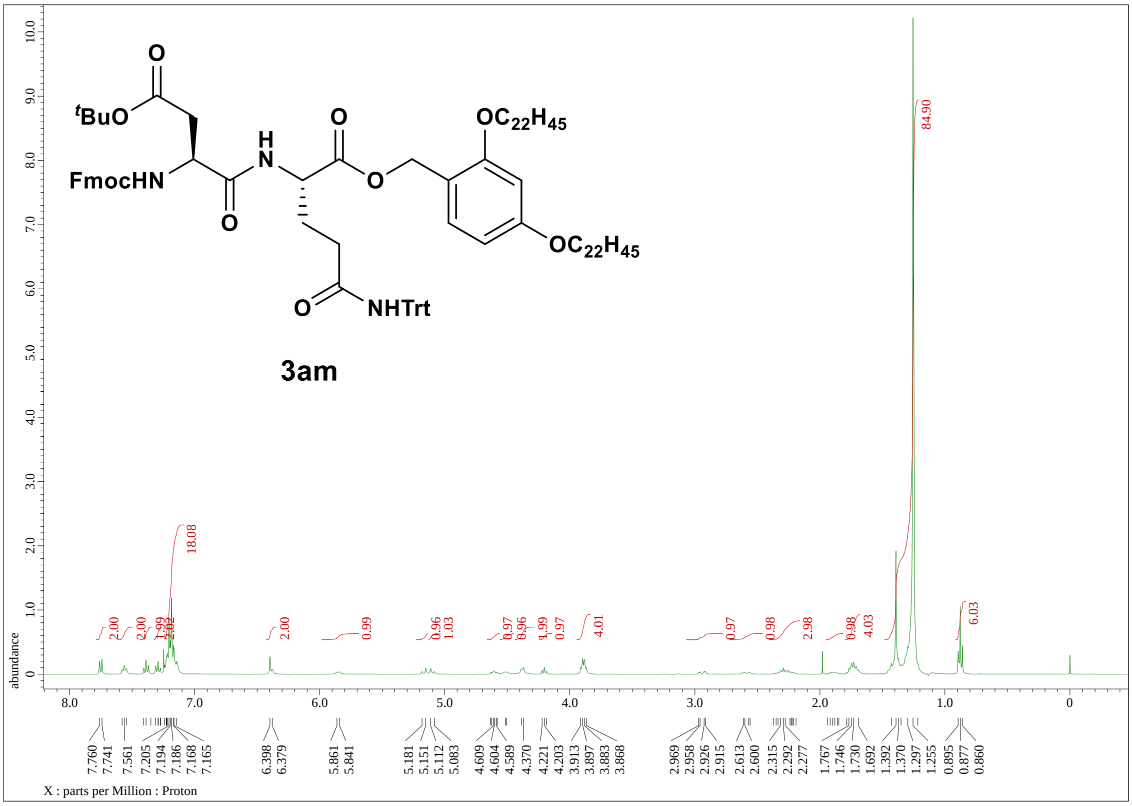
tBuO
FmocHN
OC22H45
OC22H45
NHTrt
3am
abundance
2.00 2.00 1.99 2.02 18.08 2.00 0.99 0.96 1.03 0.97 0.96 1.99 0.97 4.01 0.97 0.98 2.98 0.98 4.03 84.90 6.03
7.760 7.741 7.561 7.205 7.194 7.186 7.168 7.165 6.398 6.379 5.861 5.841 5.181 5.151 5.112 5.083 4.609 4.604 4.589 4.370 4.221 4.203 3.913 3.897 3.883 3.868 2.969 2.958 2.926 2.915 2.613 2.600 2.315 2.292 2.277 1.767 1.746 1.730 1.692 1.392 1.370 1.297 1.255 0.895 0.877 0.860
X : parts per Million : Proton

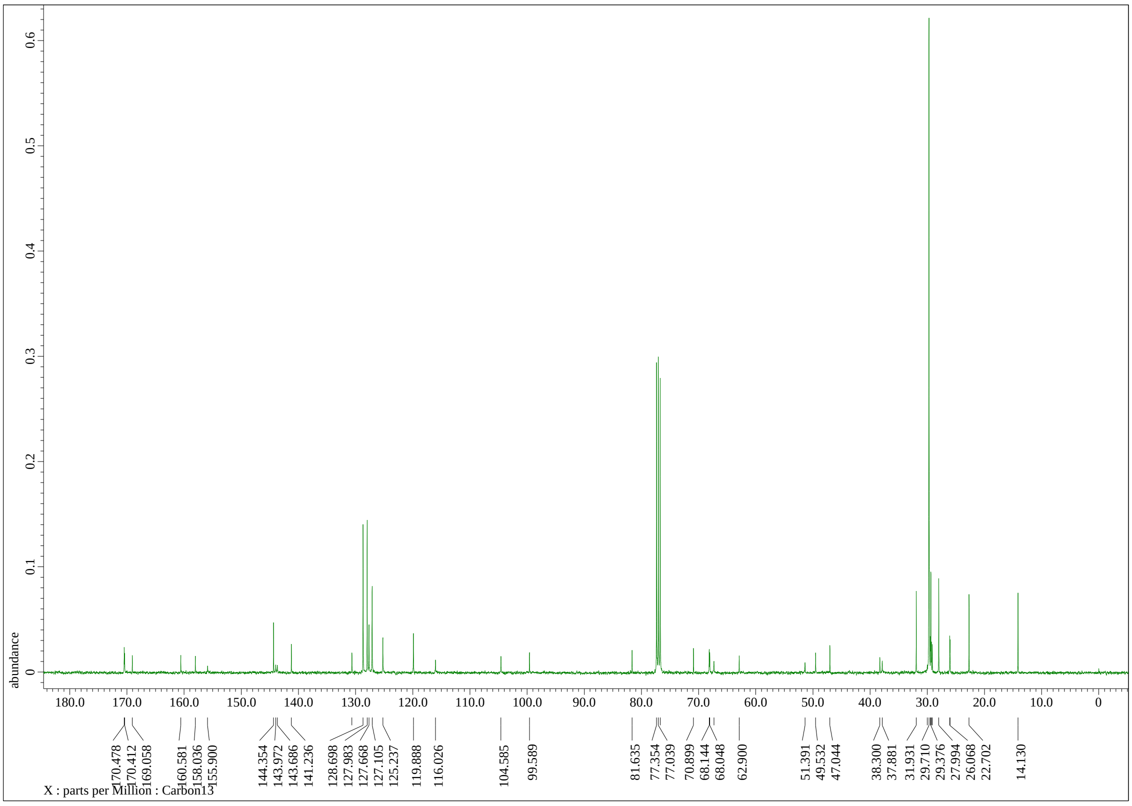
abundance
170.478 170.412 169.058 160.581 158.036 155.900 144.354 143.972 143.686 141.236 128.698 127.983 127.668 127.105 125.237 119.888 116.026 104.585 99.589 81.635 77.354 77.039 70.899 68.144 68.048 62.900 51.391 49.532 47.044 38.300 37.881 31.931 29.710 29.376 27.994 26.068 22.702 14.130
X : parts per Million : Carbon13

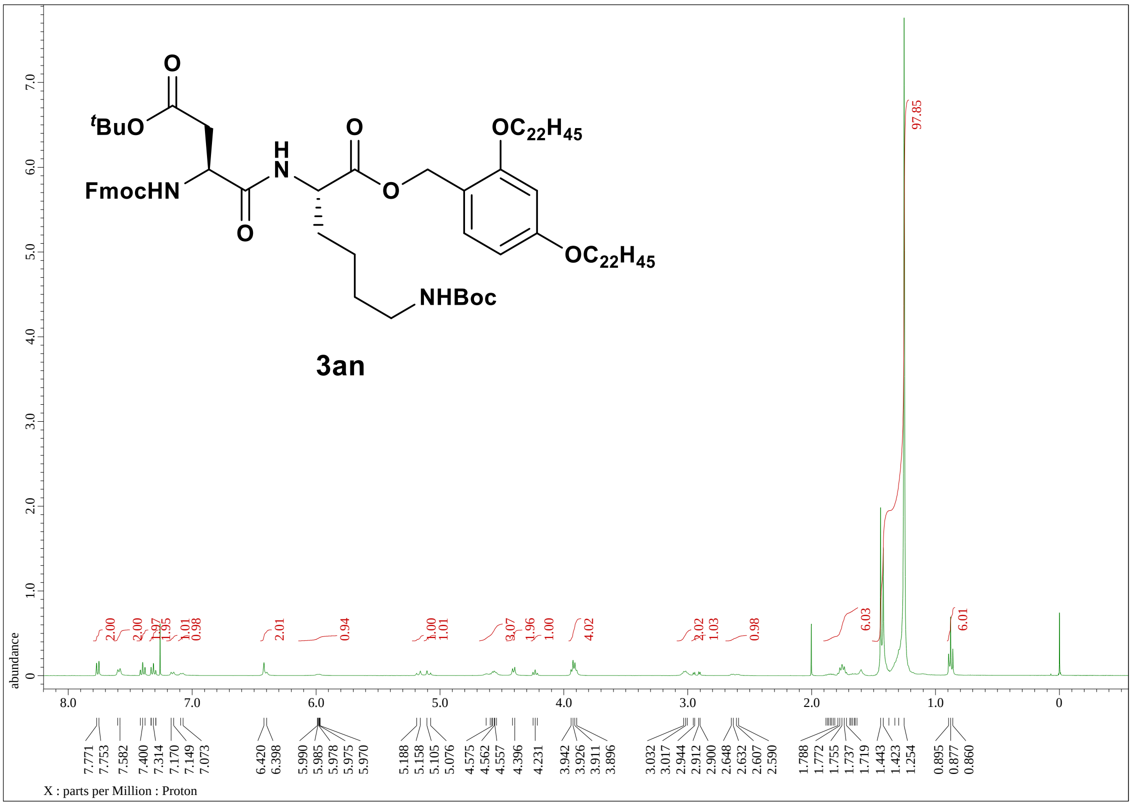

tBuO
FmocHN
NHBoc
OC22H45
OC22H45
3an
abundance
X : parts per Million : Proton
7.771 7.753 7.582 7.400 7.314 7.170 7.149 7.073 6.420 6.398 5.990 5.985 5.978 5.975 5.970 5.188 5.158 5.105 5.076 4.575 4.562 4.557 4.396 4.231 3.942 3.926 3.911 3.896 3.032 3.017 2.944 2.912 2.900 2.648 2.632 2.607 2.590 1.788 1.772 1.755 1.737 1.719 1.443 1.423 1.254 0.895 0.877 0.860
2.00 2.00 1.97 1.95 1.01 0.98 2.01 0.94 1.00 1.01 3.07 1.96 1.00 4.02 2.02 1.03 0.98 6.03 97.85 6.01

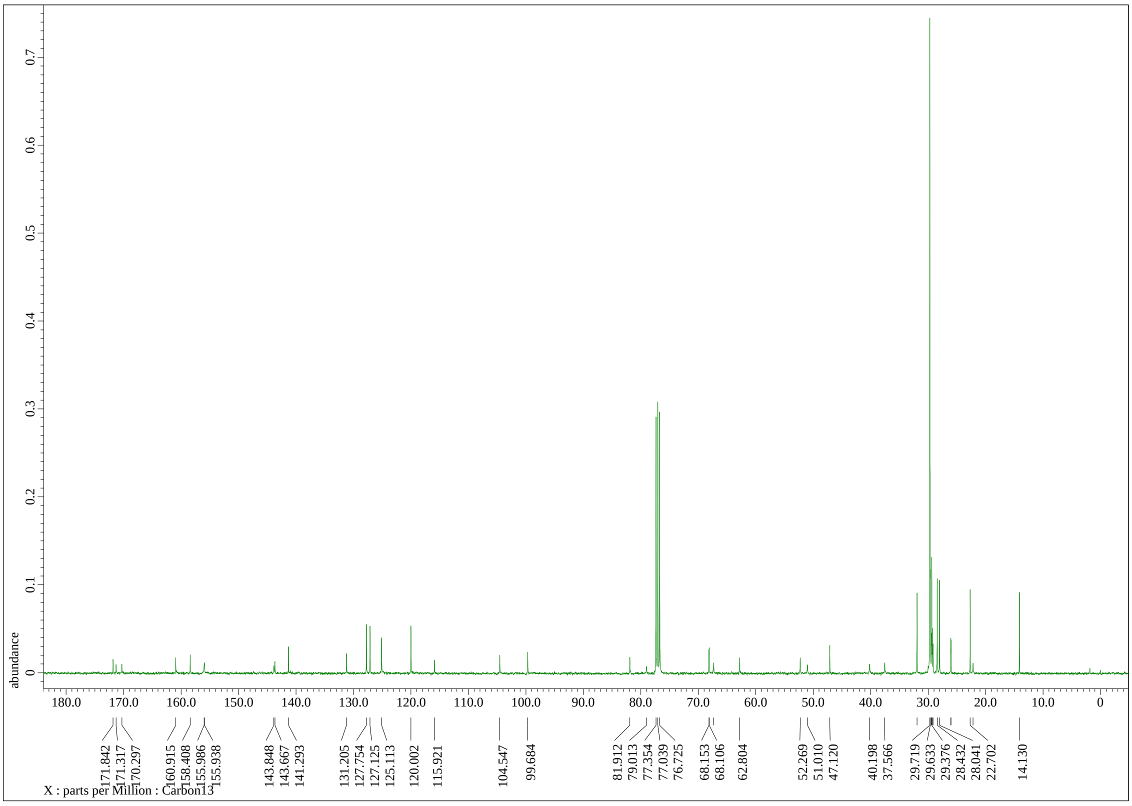

abundance
X : parts per Million : Carbon13
171.842 171.317 170.297 160.915 158.408 155.986 155.938 143.848 143.667 141.293 131.205 127.754 127.125 125.113 120.002 115.921 104.547 99.684 81.912 79.013 77.354 77.039 76.725 68.153 68.106 62.804 52.269 51.010 47.120 40.198 37.566 29.719 29.633 29.376 28.432 28.041 22.702 14.130

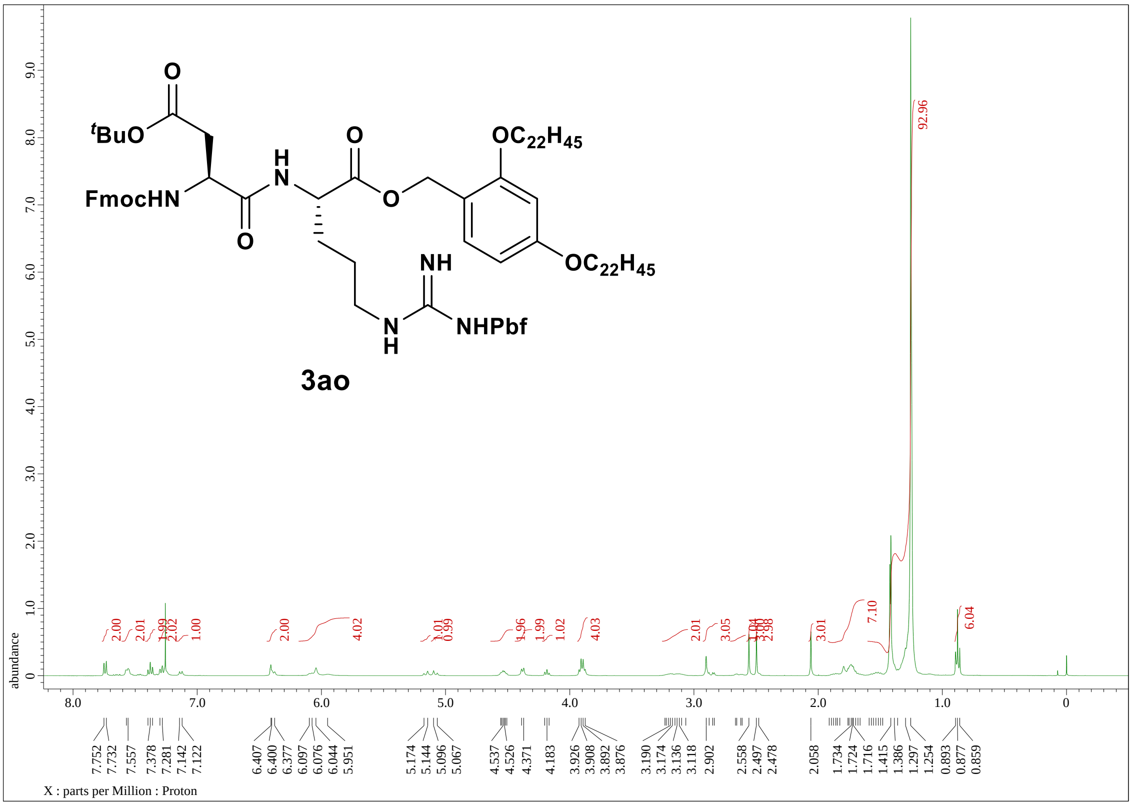

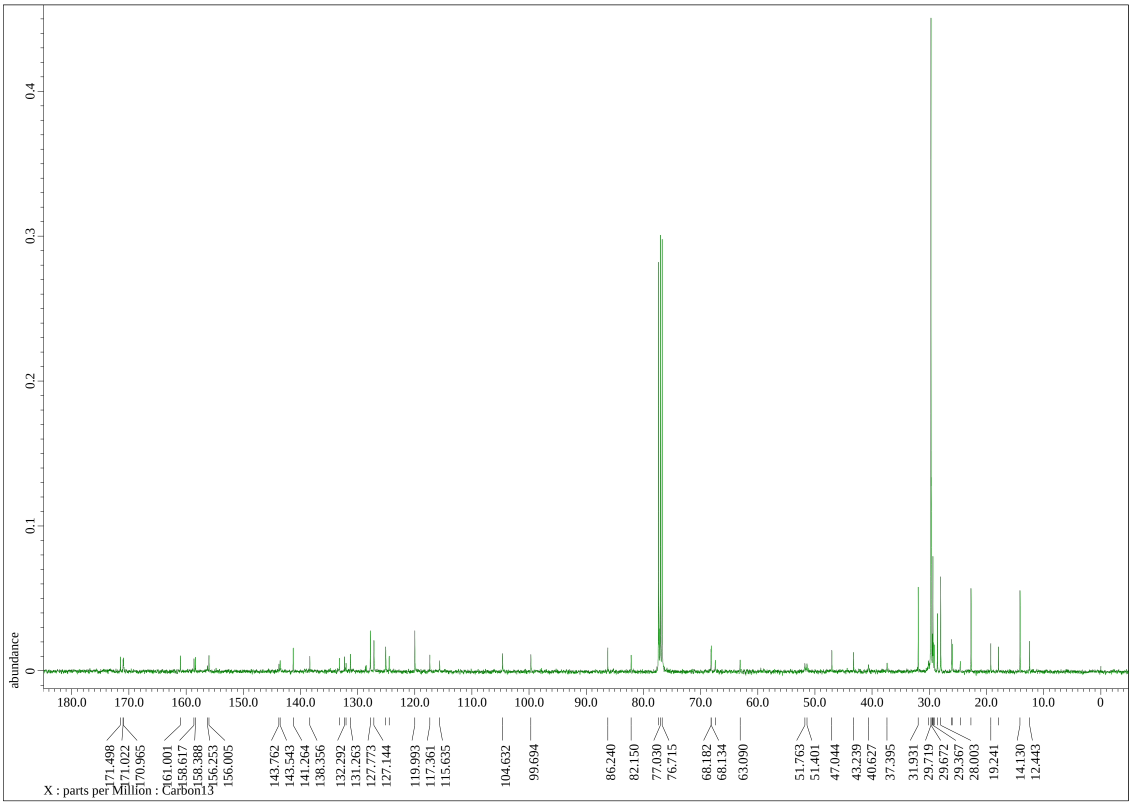

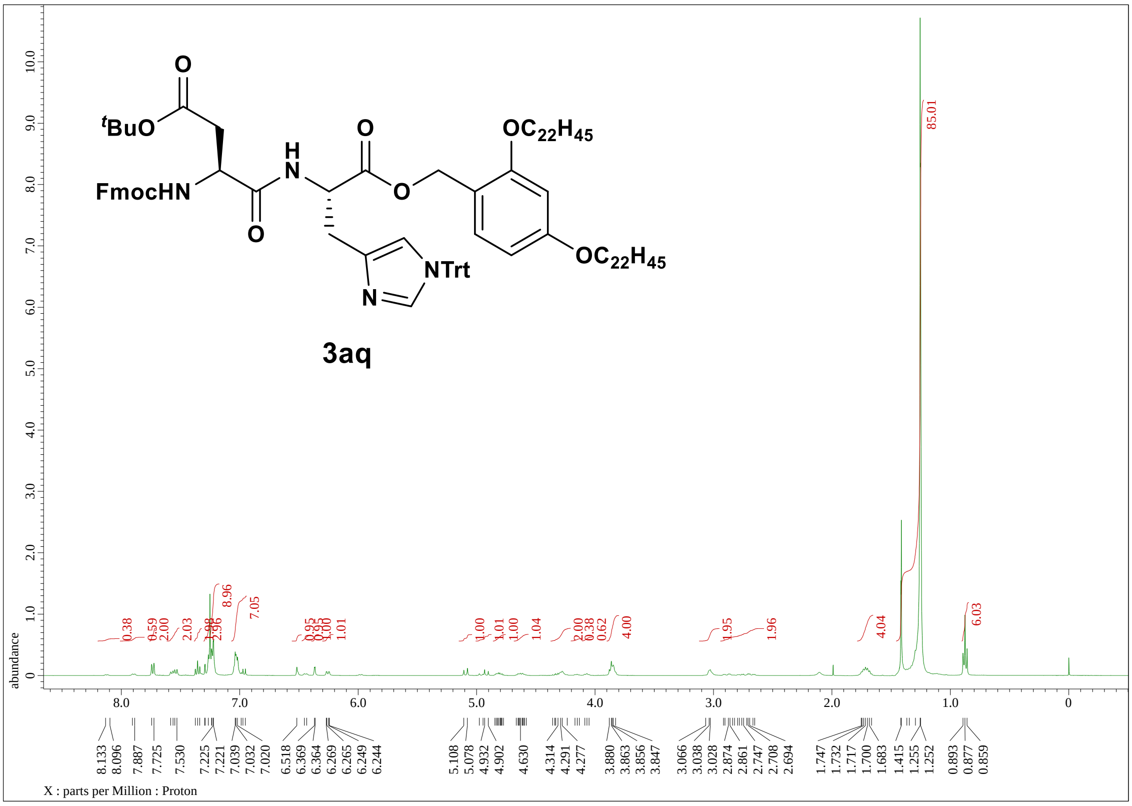
3aq
X : parts per Million : Proton
abundance

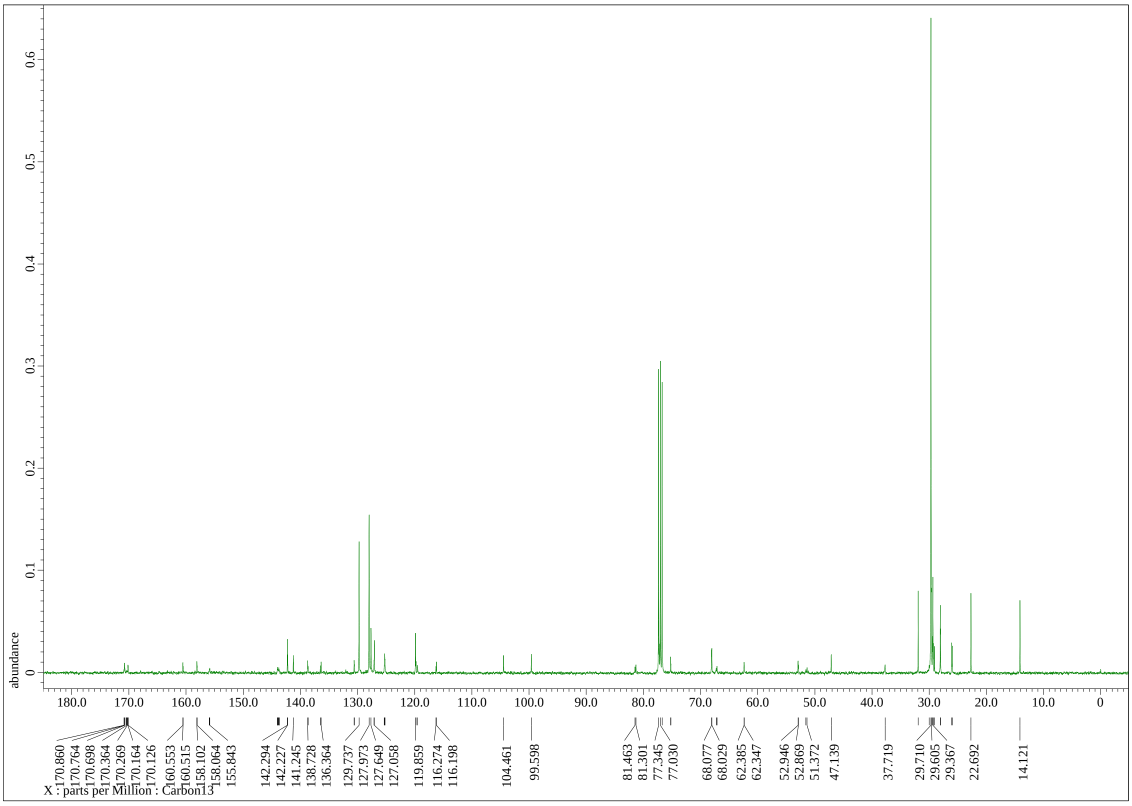
X : parts per Million : Carbon13
abundance

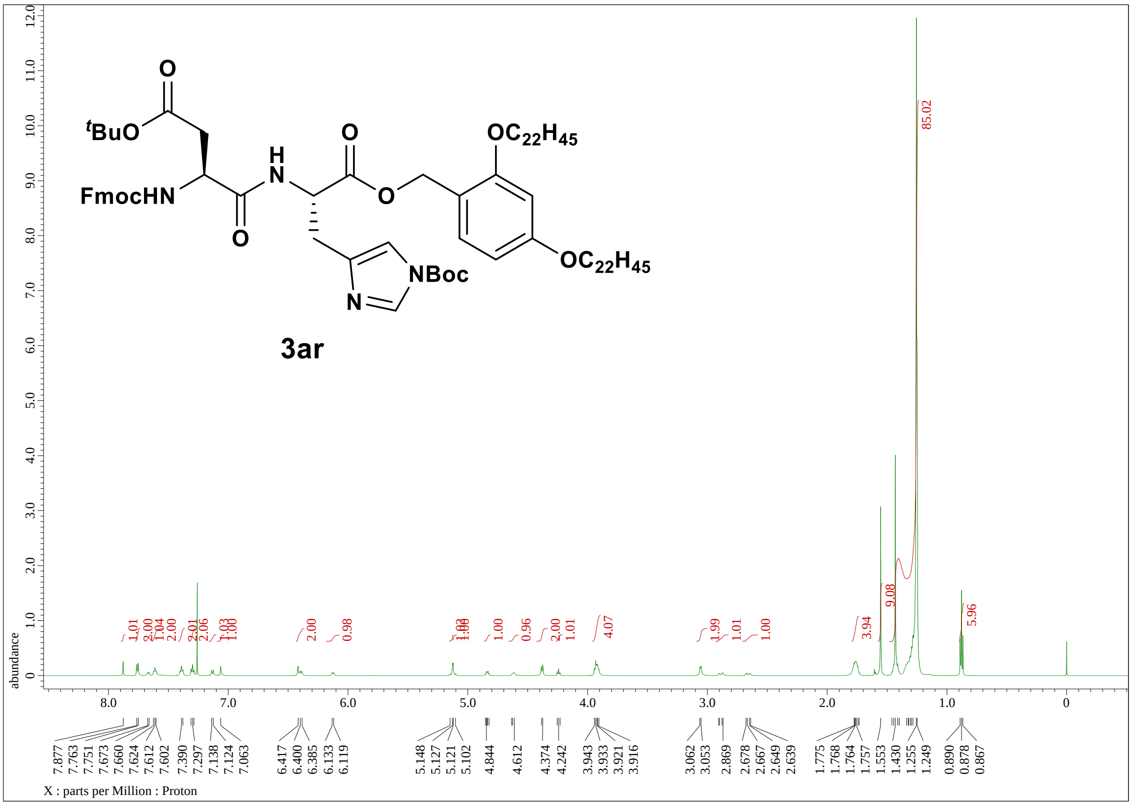

tBuO
FmocHN
OC22H45
NBoc
3ar
X : parts per Million : Proton

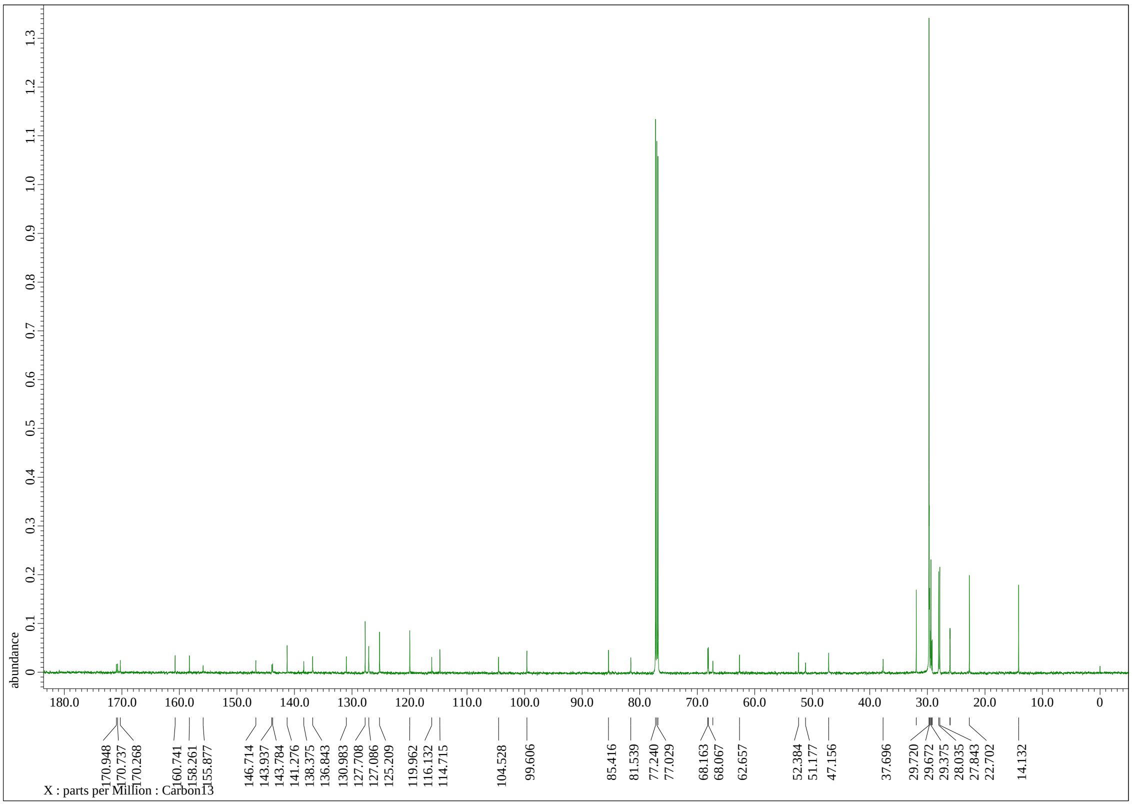

X : parts per Million : Carbon13

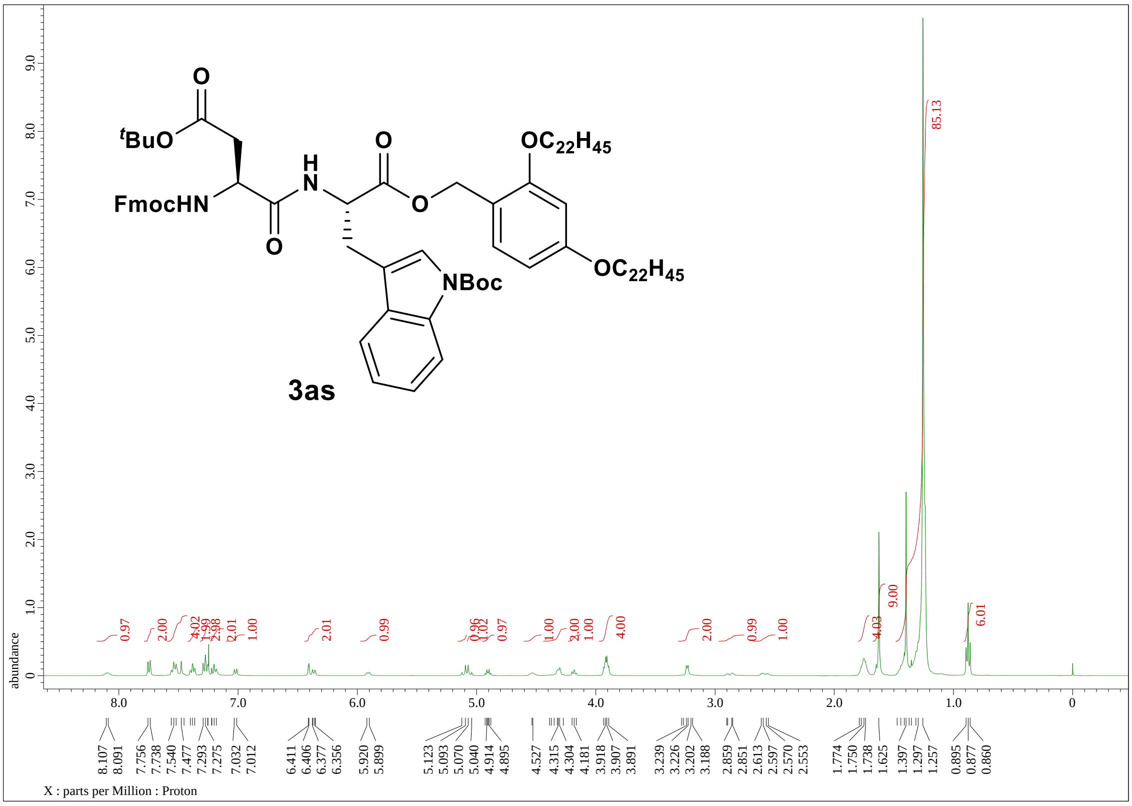

tBuO
FmocHN
NBoc
OC22H45
3as
abundance
X : parts per Million : Proton

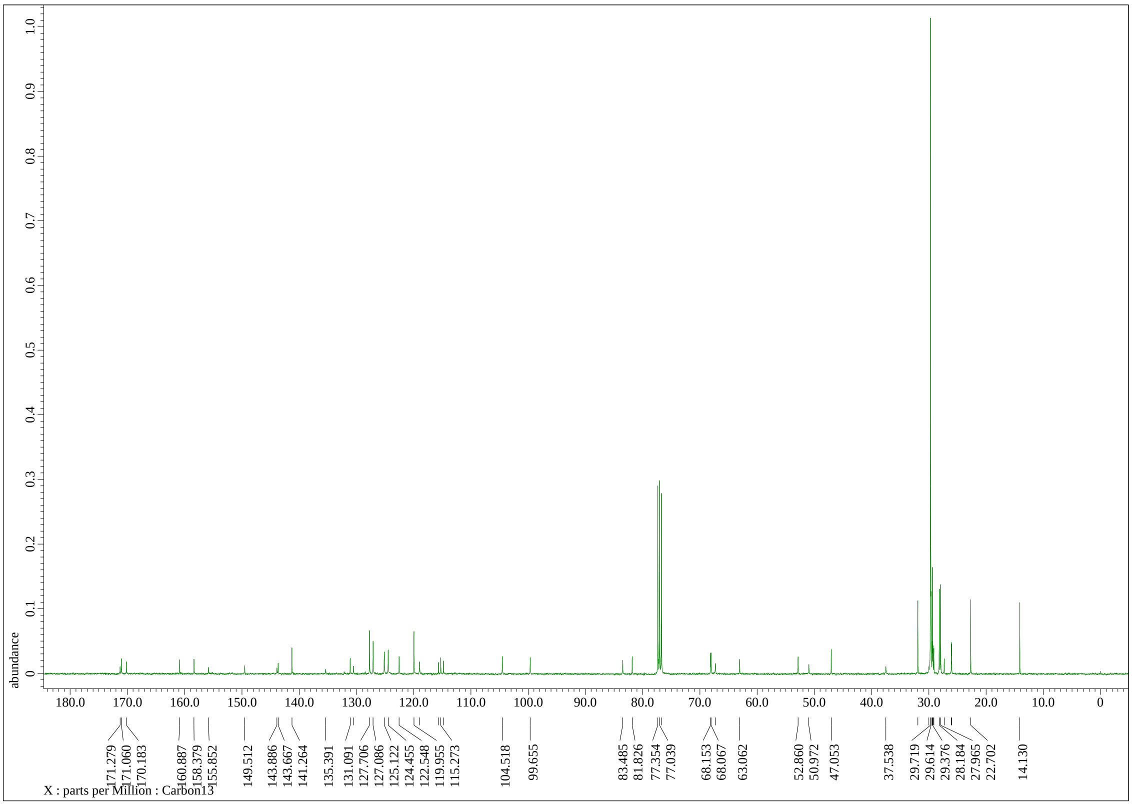

abundance
X : parts per Million : Carbon13

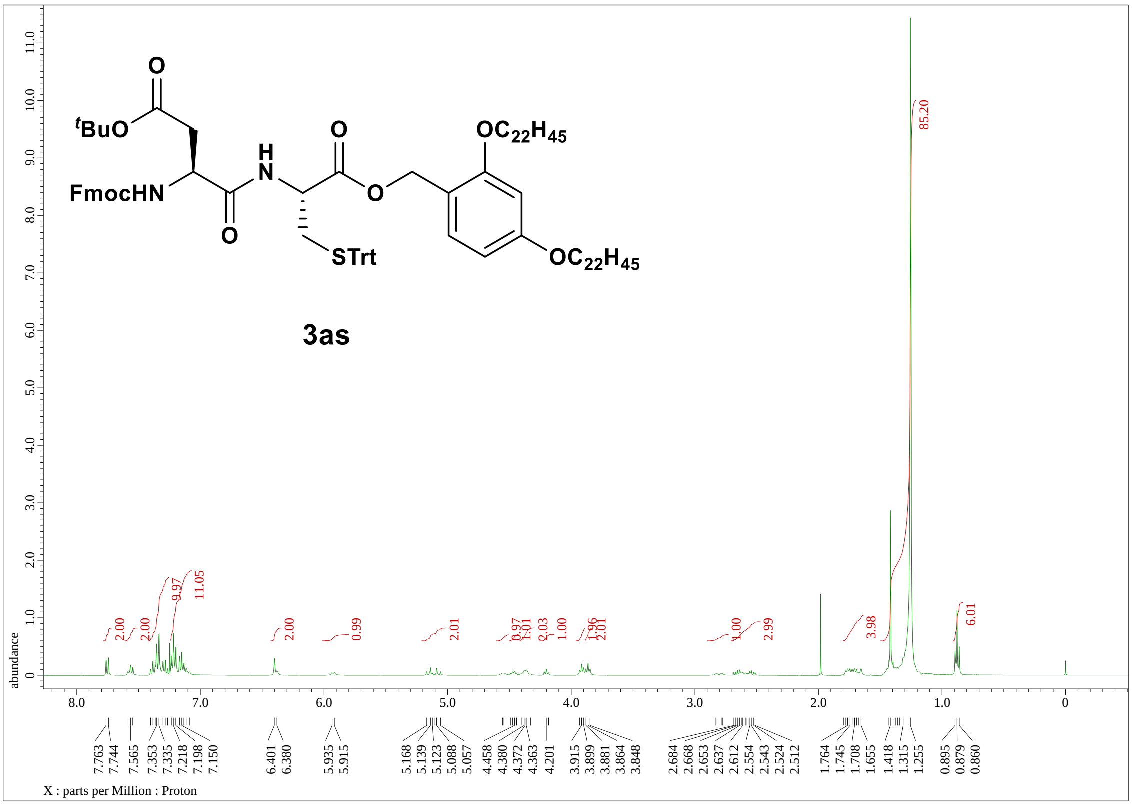
O
tBuO
FmocHN
H
N
O
O
OC22H45
STrt
OC22H45
3as
abundance
X : parts per Million : Proton
7.763
7.744
7.565
7.353
7.335
7.218
7.198
7.150
6.401
6.380
5.935
5.915
5.168
5.139
5.123
5.088
5.057
4.458
4.380
4.372
4.363
4.201
3.915
3.899
3.881
3.864
3.848
2.684
2.668
2.653
2.637
2.612
2.554
2.543
2.524
2.512
1.764
1.745
1.708
1.655
1.418
1.315
1.255
0.895
0.879
0.860
2.00
2.00
2.00
9.97
11.05
2.00
0.99
2.01
0.97
1.01
2.03
1.00
1.96
2.01
1.00
2.99
3.98
85.20
6.01

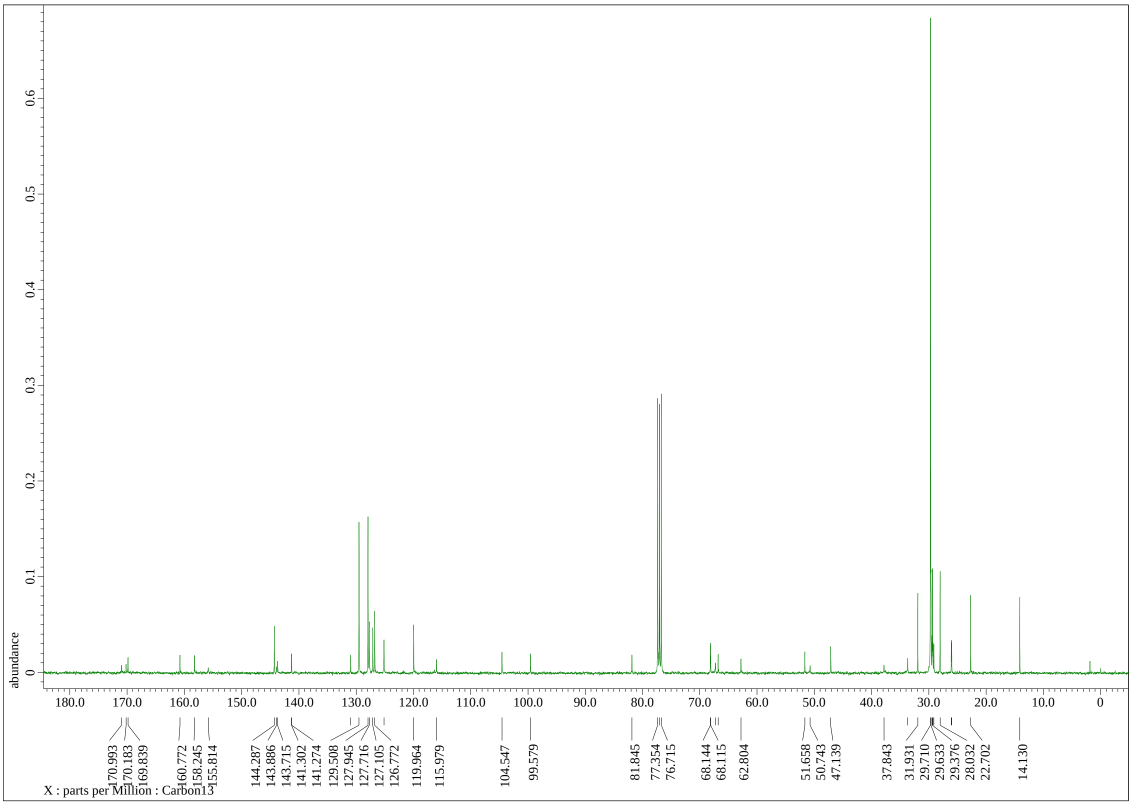
abundance
X : parts per Million : Carbon13
170.993
170.183
169.839
160.772
158.245
155.814
144.287
143.886
143.715
141.302
141.274
129.508
127.945
127.716
127.105
126.772
119.964
115.979
104.547
99.579
81.845
77.354
76.715
68.144
68.115
62.804
51.658
50.743
47.139
37.843
31.931
29.710
29.633
29.376
28.032
22.702
14.130

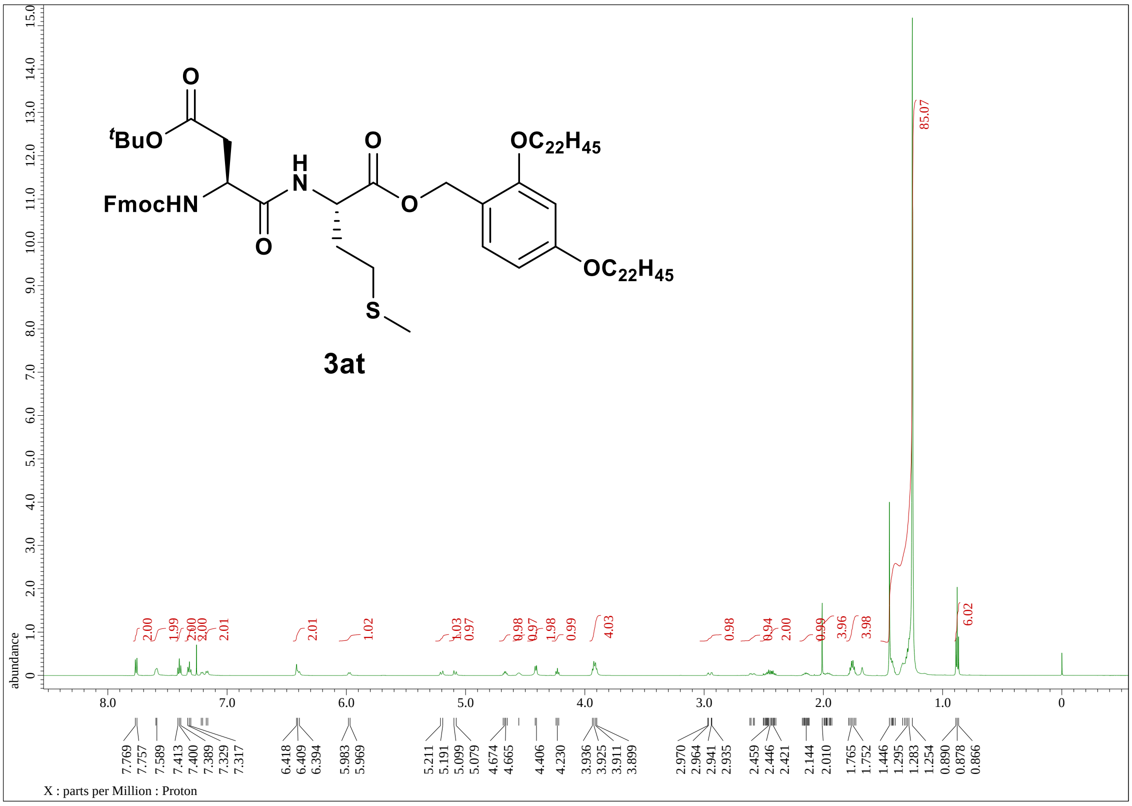
O
tBuO
FmocHN
H
N
O
O
O
OC22H45
OC22H45
S
3at
abundance
2.00 1.99 2.00 2.00 2.01 2.01 1.02 1.03 0.97 0.98 0.97 1.98 0.99 4.03 0.98 0.94 2.00 0.99 3.96 3.98 85.07 6.02
7.769 7.757 7.589 7.413 7.400 7.389 7.329 7.317 6.418 6.409 6.394 5.983 5.969 5.211 5.191 5.099 5.079 4.674 4.665 4.406 4.230 3.936 3.925 3.911 3.899 2.970 2.964 2.941 2.935 2.459 2.446 2.421 2.144 2.010 1.765 1.752 1.446 1.295 1.283 1.254 0.890 0.878 0.866
X : parts per Million : Proton

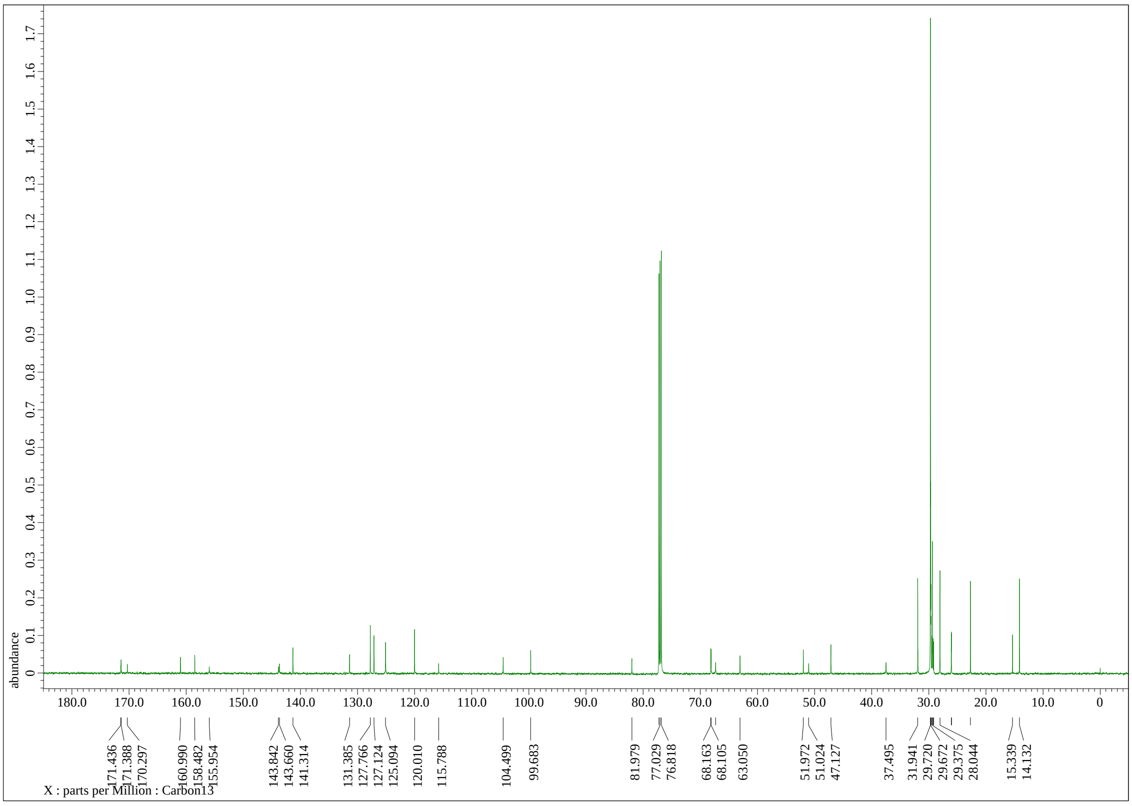
abundance
171.436 171.388 170.297 160.990 158.482 155.954 143.842 143.660 141.314 131.385 127.766 127.124 125.094 120.010 115.788 104.499 99.683 81.979 77.029 76.818 68.163 68.105 63.050 51.972 51.024 47.127 37.495 31.941 29.720 29.672 29.375 28.044 15.339 14.132
X : parts per Million : Carbon13

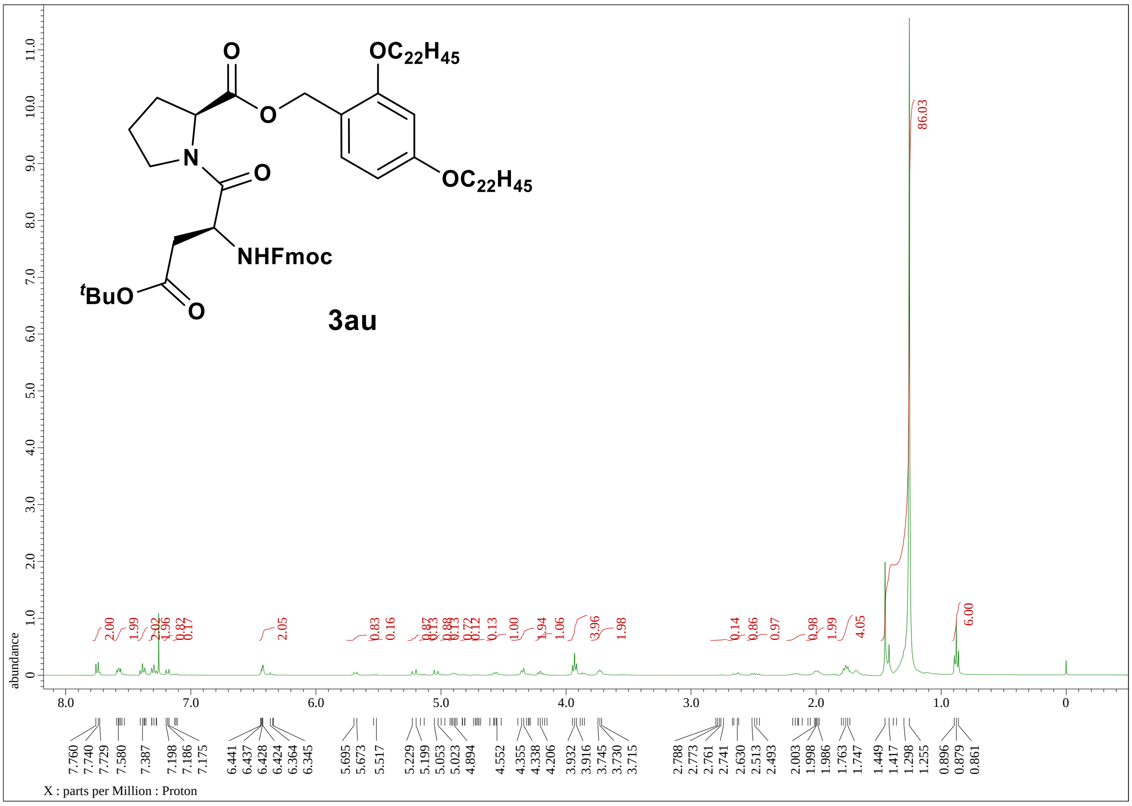

3au
X : parts per Million : Proton
abundance

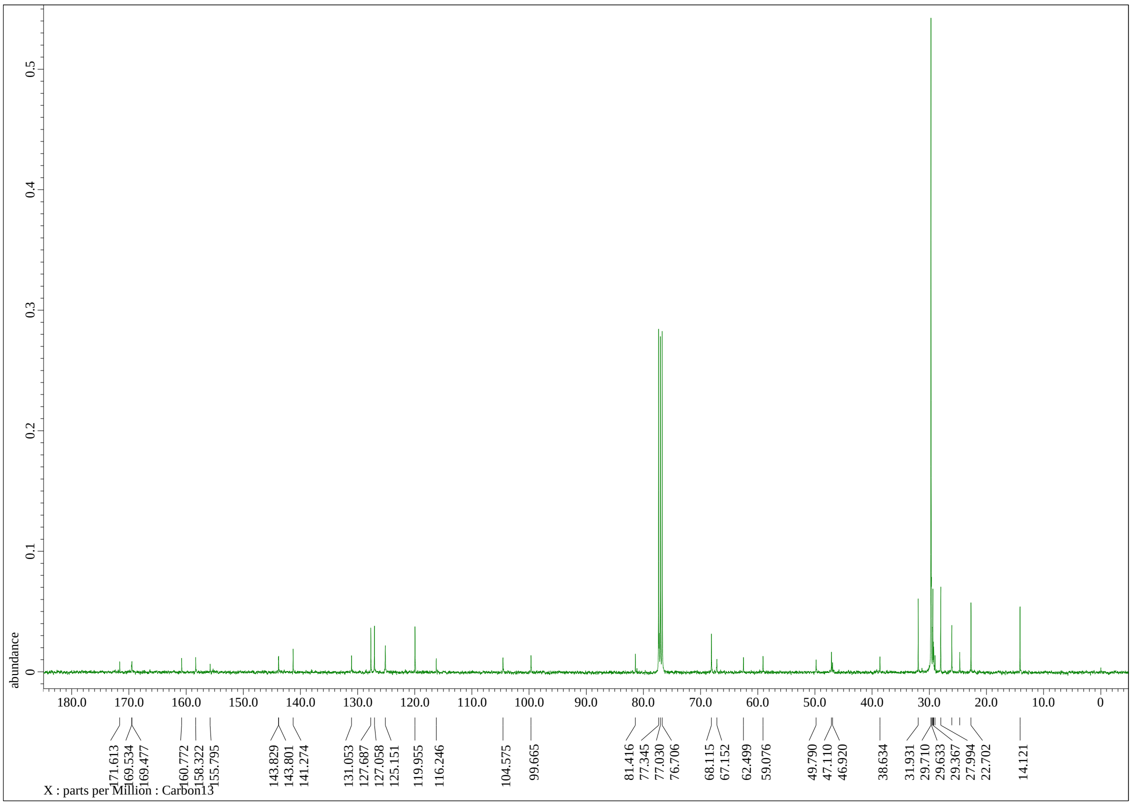

X : parts per Million : Carbon13
abundance

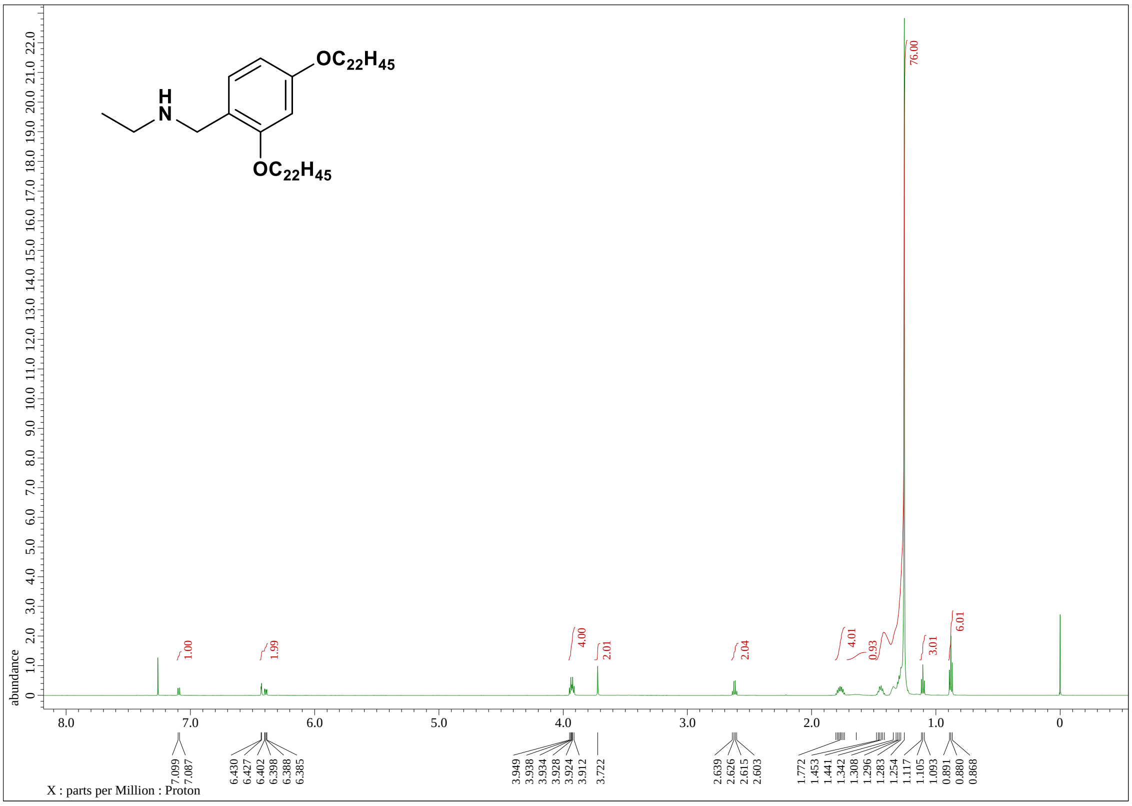
OC22H45
H
N
OC22H45
abundance
76.00
1.00
1.99
4.00
2.01
2.04
4.01
0.93
3.01
6.01
7.099
7.087
6.430
6.427
6.402
6.398
6.388
6.385
3.949
3.938
3.934
3.928
3.924
3.912
3.722
2.639
2.626
2.615
2.603
1.772
1.453
1.441
1.342
1.308
1.296
1.283
1.254
1.117
1.105
1.093
0.891
0.880
0.868
X : parts per Million : Proton

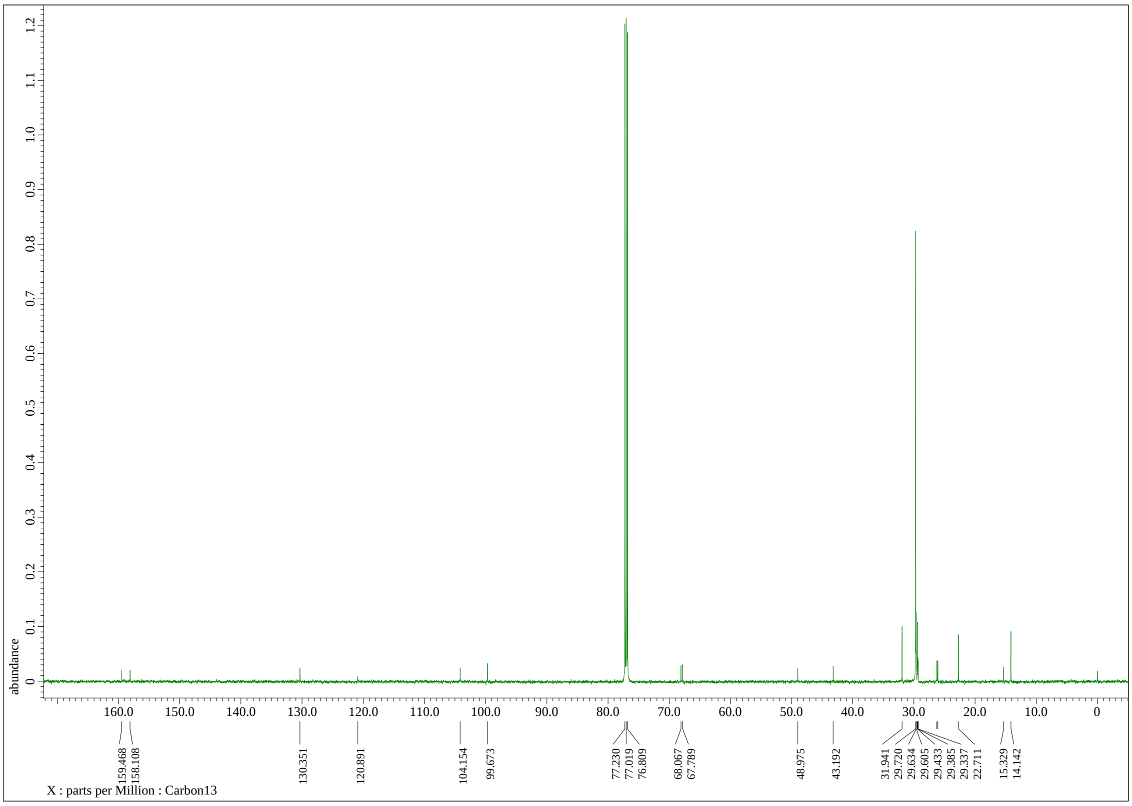
abundance
159.468
158.108
130.351
120.891
104.154
99.673
77.230
77.019
76.809
68.067
67.789
48.975
43.192
31.941
29.720
29.634
29.605
29.433
29.385
29.337
22.711
15.329
14.142
X : parts per Million : Carbon13

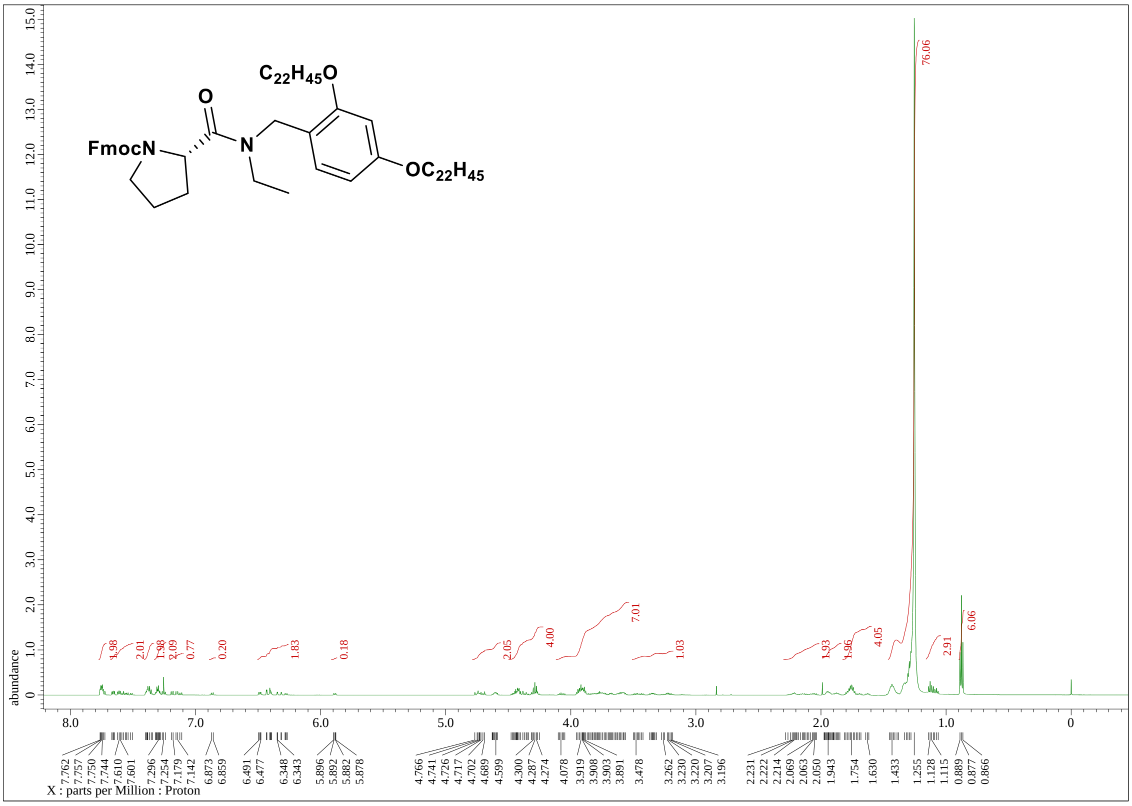

C22H45O
O
FmocN
N
OC22H45
X : parts per Million : Proton
abundance

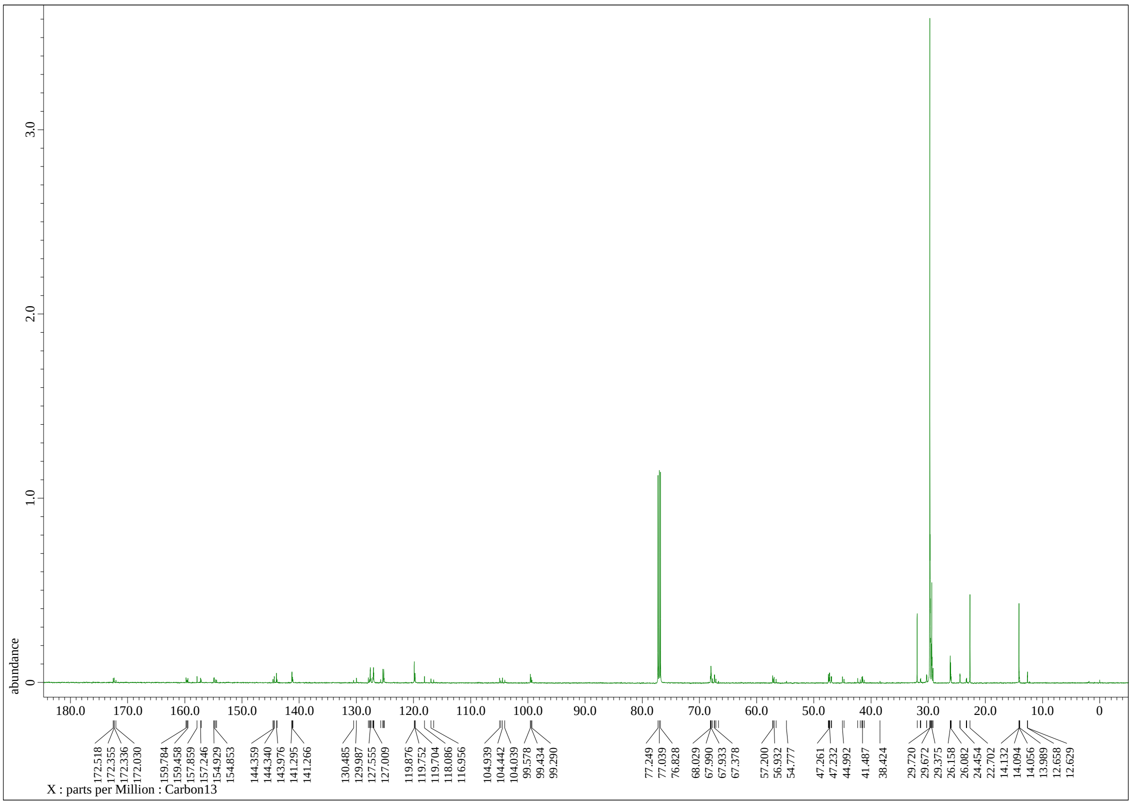

X : parts per Million : Carbon13
abundance
